# Catalytic Undirected Meta‐Selective C–H Borylation of Metallocenes

**DOI:** 10.1002/advs.202304672

**Published:** 2023-08-26

**Authors:** Hao Zheng, Chang‐Hui Liu, Xiao‐Yu Wang, Yan Liu, Bing‐Zhi Chen, Yan‐Cheng Hu, Qing‐An Chen

**Affiliations:** ^1^ Dalian Institute of Chemical Physics Chinese Academy of Sciences Dalian 116023 P. R. China; ^2^ University of Chinese Academy of Sciences Beijing 100049 P. R. China; ^3^ School of Chemical Engineering and Technology China University of Mining and Technology Xuzhou Jiangsu 221116 P. R. China

**Keywords:** C–H borylation, directing group free, ferrocene, iridium catalysis, meta‐selective

## Abstract

Metallocenes are privileged backbones in the fields of synthetic chemistry, catalysis, polymer science, etc. Direct C–H functionalization is undoubtedly the simplest approach for tuning the properties of metallocenes. However, owing to the presence of multiple identical C(sp^2^)‐H sites, this protocol often suffers from low reactivity and selectivity issues, especially for the regioselective synthesis of 1,3‐difunctionalized metallocenes. Herein, an efficient iridium‐catalyzed meta‐selective C–H borylation of metallocenes is reported. With no need of preinstalled directing groups, this approach enables a rapid synthesis of various boronic esters based on benzoferrocenes, ferrocenes, ruthenocene, and related half sandwich complex. A broad range of electron‐deficient and ‐rich functional groups are all compatible with the process. Notably, C–H borylation of benzoferrocenes takes place exclusively at the benzene ring, which is likely ascribed to the shielding effect of pentamethylcyclopentadiene. The synthetic utility is further demonstrated by easy scalability to gram quantities, the conversion of boron to heteroatoms including N_3_, SePh, and OAc, as well as diverse cross‐coupling reactions.

## Graphical abstract

With Ir(I) catalyst and dinitrogen ligands, a meta‐selective C–H borylation of metallocenes enabled by steric control strategy is herein realized. With no need of directing groups, this practical method allows for a rapid synthesis of various useful boronic esters based on benzoferrocenes, ferrocenes, ruthenocene, and related half sandwich complex.

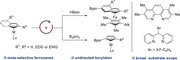

## 1 Introduction

**Figure 1:**
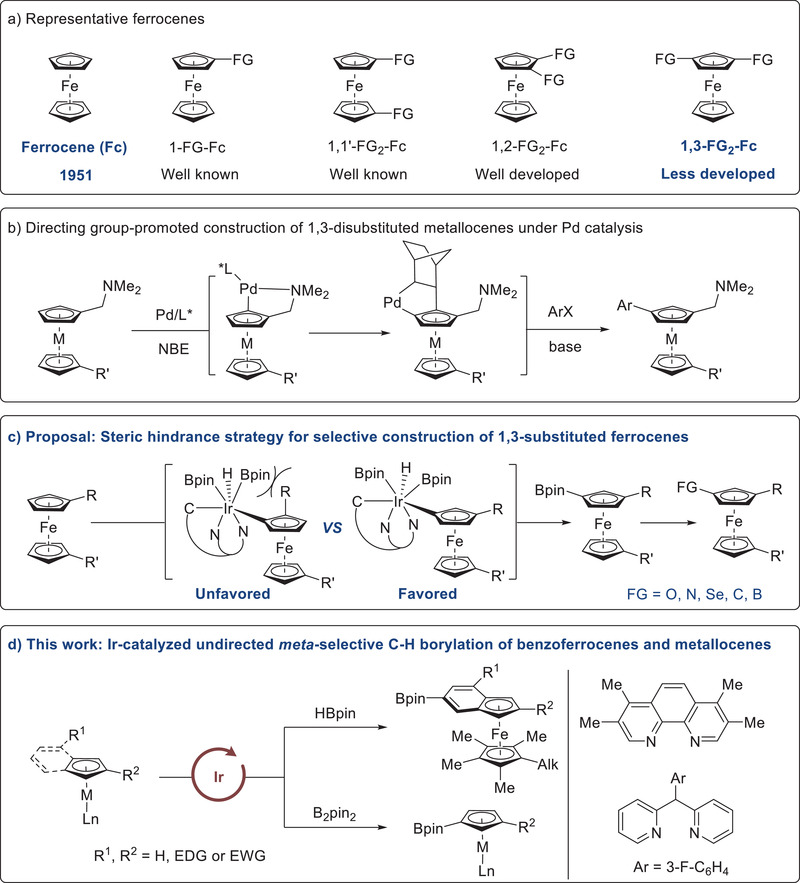
Synthesis of substituted metallocenes.

Since their serendipitous discovery and determination of the sandwich‐type structure in the early 1950s,^[^ ^1^ ^]^ ferrocenes and their derivatives have found extensive applications in various areas including homogeneous catalysis,^[^ ^2^ ^]^ polymer science,^[^ ^3^ ^]^ and medicinal chemistry.^[^ ^4^ ^]^ Consequently, the development of new functionalization of ferrocenes has always been a hot research spot. The synthetic approaches towards mono‐functionalized,^[^ ^5^ ^]^ 1,1′‐^[^ ^6^ ^]^ and 1,2‐bisfunctionalized ferrocenes^[^ ^7^ ^]^ are well developed. In contrast, the selective construction of 1,3‐bisfunctionalized ferrocenes remains rarely studied (**Figure** **1**a). From the viewpoint of synthetic efficiency, direct C–H functionalization is arguably the simplest strategy. However, the presence of multiple identical C(sp^2^)‐H sites makes regioselectivity control a great challenge. More recently, by taking advantage of pre‐installed amino group, Yu and Zhou^[^ ^8^ ^]^ et al accomplished an elegant asymmetric meta‐selective C–H arylation of ferrocenes via Pd‐catalyzed Catellani process (Figure 1b). The directing group is crucial for achieving the high regioselectivity. While applicable, it would be more appealing to exploit an undirected meta‐selective C–H functionalization of ferrocenes, which can further improve atom‐ and step‐economy.

From the perspective of molecular diversification, organoboron compounds are versatile precursors for the creation of new C–C^[^ ^9^ ^]^ and C–heteroatom bonds.^[^ ^10^ ^]^ In this regard, the selective incorporation of boron into metallocenes is of great significance for the rapid assembly of metallocene library, which can in turn promote the advancement of related fields. In 2004, Plenio^[^ ^11^ ^]^ and co‐workers pioneered Ir‐catalyzed C–H borylation of ferrocenes with dtbbpy ligand, in which ferrocenes containing electron‐donor groups were not suitable. The recent years have witnessed impressive progress^[^ ^12^ ^]^ in iridium‐catalyzed C–H borylation of alkanes,^[^ ^13^ ^]^ heteroarenes,^[^ ^14^ ^]^ and electron‐biased arenes.^[^ ^15^ ^]^ With the aid of dinitrogen ligands, steric interactions can effectively block proximal sites, thus enabling selective activation of remote C–H sites. Prompted by this steric control strategy and our continuous interest in metallocenes modification, we envisioned that owing to the sterically crowded nature of the active Ir catalyst, uncrowded meta‐C–H bond of substituted ferrocenes might be preferentially activated by oxidative addition, thus finally leading to the formation of meta‐functionalized products (Figure 1c). Just as anticipated, when using tetramethylphenanthroline or 2,2′‐dipyridylarylmethane as ligand, Ir‐catalyzed borylation of metallocenes takes place exclusively at meta C–H site (Figure 1d). Without directing group, this general approach allows for a facile synthesis of diverse 1,3‐disubstituted benzoferrocenes, ferrocenes, ruthenocene and related half sandwich complex. Notably, C–H borylation of benzoferrocenes preferably proceeds at the benzene ring, likely due to the shielding effect of pentamethylcyclopentadiene. Additional features include wide functional group tolerance, easy scalability, and useful synthetic transformations. Herein, we demonstrate these preliminary results.

## 2 Results and Discussion

Compared with the progress achieved in the chemistry of simple ferrocene, the research in benzoferrocene lags far behind, mainly due to a lack of general functionalization methodology. Given this fact, C–H borylation of benzoferrocene was first investigated. Benzoferrocene **1a** and pinacolborane **2a** were selected as model substrates to optimize the reaction conditions (**Table** **1**). With a combination of [Ir(cod)OMe]_2_ and 3,4,7,8‐tetramethyl‐1,10‐phenanthroline (**L2**), the benzene ring of benzoferrocene **1a** was selectively borylated in methylcyclohexane (MeCy) at 100 °C and product **3a** was isolated in up to 95% yield (entry 1). The naked phenanthroline **L1** and mono‐substituted phenanthrolines (**L3**, **L4**) sharply decreased the reaction efficiency (entries 2−4). Besides, other bidentate dinitrogen ligands with distinct backbone were also tested. 4,4′‐Di‐*tert*‐butyl‐2,2′‐bipyridine (dtbbpy) **L5** afforded **3a** in only 18% yield (entry 5), while qunioline‐oxazoline ligand **L6** and thiophene‐pyridine ligand **L7** completely inhibited the reaction (entries 6–7). It is worthwhile to mention that 2,2′‐dipyridylphenylmethane ligand **L8** could also promote the process but with a moderate yield (entry 8). Other catalytic systems such as [Cp*IrCl_2_]_2_ or Ni(cod)_2_/ICy proved to be inefficient and a very low yield was obtained with [Ir(cod)Cl]_2_ as catalyst precursor (entries 9−11). Reducing the temperature resulted in a decline in the yield (entry 12). Finally, tetrahydrofuran (THF) and 1,4‐dioxane could also be used as the solvents for this transformation but with slightly decreased yields (entries 13−14).

**Table 1:** Optimization of the reaction conditions

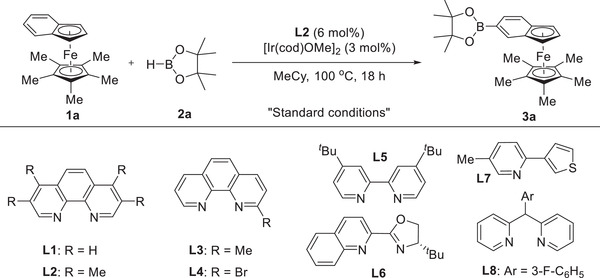		
Entry^a)^	Deviation from standard conditions	3a Yield [%]
1	None	99% (95%)^b)^
2	**L1** instead of **L2**	31%
3	**L3** instead of **L2**	35%
4	**L4** instead of **L2**	N. D.
5	**L5** instead of **L2**	18%
6	**L6** instead of **L2**	N. D.
7	**L7** instead of **L2**	N. D.
8	**L8** instead of **L2**	56%
9	Ni(cod)_2_ and ICy instead of [Ir(cod)OMe]_2_ and **L2**	N. D.
10	[Cp*IrCl_2_]_2_ instead of [Ir(cod)OMe]_2_ and **L2**	N. D.
11	[Ir(cod)Cl]_2_ instead of [Ir(cod)OMe]_2_	10%
12	80 °C instead of 100 ^o^C	82%
13	THF instead of MeCy	87%
14	Dioxane instead of MeCy	81%

^a)^ Conditions: **1a** (0.20 mmol), **2a** (1.0 mmol), [Ir(cod)OMe]_2_ (0.006 mmol), **L** (0.012 mmol), solvent (1.0 mL), 100 °C, under N_2_, 18 h. Yields were determined by ^1^H NMR spectroscopy using 1,3,5‐trimethoxybenzene as the internal standard;

^b)^ Isolated yield.

**Figure 2:**
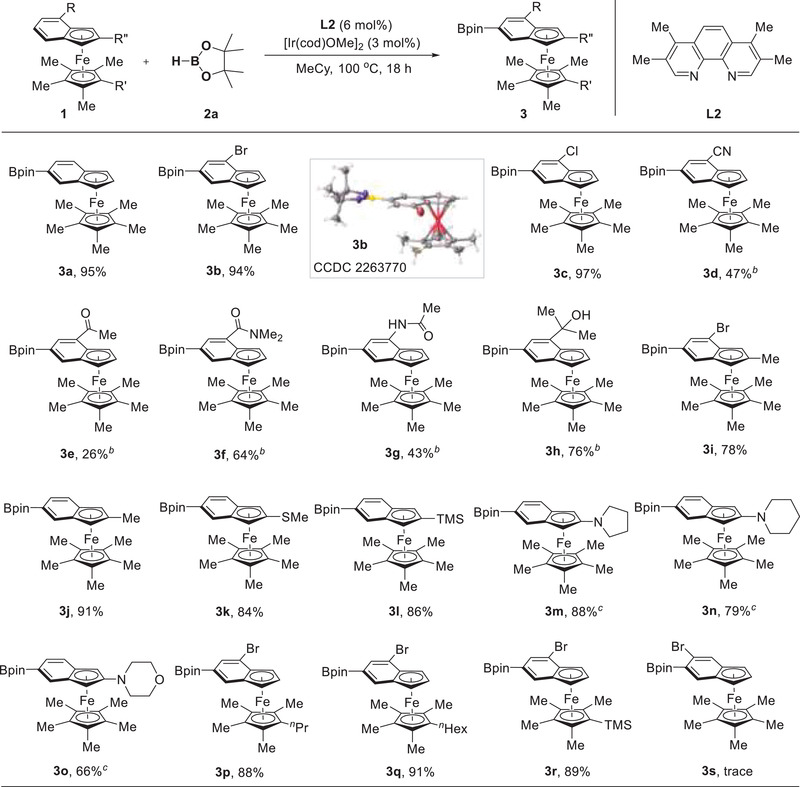
Substrate scope for benzoferrocenes. *^a^* Conditions: **1** (0.20 mmol), **2a** (1.0 mmol), [Ir(cod)OMe]_2_ (0.006 mmol), **L2** (0.012 mmol), MeCy (1.0 mL), 100 °C, under N_2_, 18 h. *^b^* B_2_pin_2_ as boron source (0.3 mmol). *^c^* 120 °C.

With the optimized conditions in hand, we then evaluated the substrate scope for this iridium‐catalyzed undirected meta‐C–H borylation of substituted benzoferrocenes. As shown in **Figure** **2**, all substrates underwent borylation exclusively at the *β*‐position of phenyl ring. More importantly, competitive C–H borylation at *α*‐position of phenyl ring or Cp (cyclopentadienyl) ring was not observed. The halide groups on the phenyl ring of benzoferrocenes were well tolerated and the boronic esters (**3b**, **3c**) were afforded in high yields. The regioselectivity was unambiguously confirmed by X‐ray analysis of **3b** (CCDC 2 263 770, see the Supporting Information for detail). The strong electron‐withdrawing substituents such as cyano and carbonyl were also applicable to this transformation (**3d**, **3e**), and relatively decreased yields resulted from the facile decomposition of these substrates. In the case of benzoferrocenes possessing amides and free hydroxyl group, the borylation gave the target products in moderate yields because these substrates could not be totally consumed (**3f–3 h**). The nature of the substituents on the Cp ring of benzoferrocenes exerted no significant impact on the outcome. For instance, methyl, sulfide and silyl were all compatible with the process, leading to the corresponding products **3i–3l** in good yields. Remarkably, this protocol could be successfully extended to borylation of heterocycle‐derived substrates but an elevated temperature was required (**3m**–**3o**). Furthermore, when replacing methyl group of the Cp* ring with other substituents such as propyl, hexyl, and TMS, the desired borylation also occurred with high efficiencies (**3p–3r**). Benzoferrocene bearing bromo at the *β*‐position of phenyl ring was an ineffective substrate, likely due to the steric hindrance.

**Figure 3:**
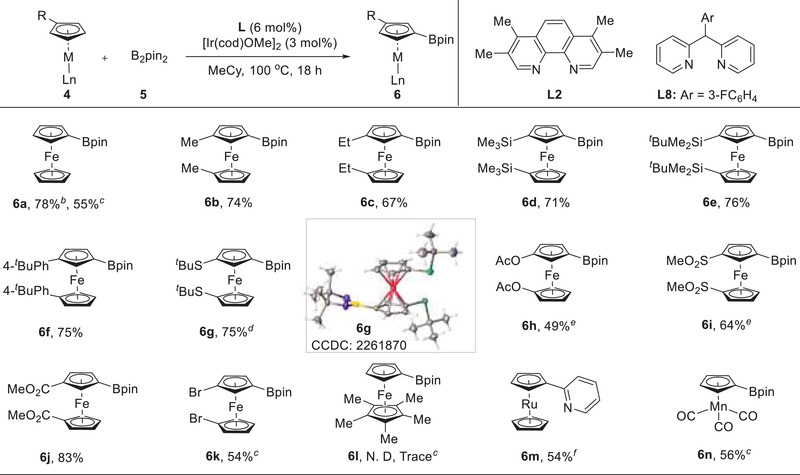
*^a^* Conditions: **4** (0.20 mmol), **5** (0.30 mmol), [Ir(cod)OMe]_2_ (0.006 mmol), **L8** (0.012 mmol), MeCy (1.0 mL), 100 °C, under N_2_, 18 h. *^b^* B_2_pin_2_ (0.10 mmol). *^c^* **L2** as ligand and HBpin as boron source. *^d^* B_2_pin_2_ (0.20 mmol). *^e^* THF as solvent. *^f^* B_2_pin_2_ (0.20 mmol) with **L2**, then treated with 2‐bromopyridine in the presence of Pd(PPh_3_)_4_.

Subsequently, C–H borylation of simple metallocenes and related half‐sandwich compounds was investigated (**Figure** **3**). The coupling between simple ferrocene and HBpin indeed took place under the standard conditions but the only 55% yield was obtained for the bisborylation. After further screening the ligand and boron source, we were delighted to find that when using B_2_pin_2_ as partner and 2,2′‐dipyridylphenylmethane **L8** as ligand, the desired mono‐borylated product **6a** was delivered in 78% yield while the bisborylation was observed. Under this modified condition, 1,1′‐dialkyl ferrocenes underwent meta‐C–H borylation smoothly, providing the target products (**6b**, **6c**) in decent yields. 1,1′‐Diphenyl ferrocene participated in the process as well and 75% yield of **6f** was achieved. Besides, the silyl and sulfide units were all tolerated, resulting in the formation of the corresponding boronic esters in good yields (**6d**, **6e**, **6g**). When using THF to increase the substrate solubility, the ferrocenes bearing ester and sulfone groups could be readily transformed into their borylated products (**6** **h**, **6i**). The electron‐withdrawing groups ‐Br and ‐CO_2_Me were also applicable to this borylation (**6j**, **6k**). Unfortunately, C–H borylation of pentamethylferrocene was completely inhibited, likely because its five C─H bonds of Cp ring were shielded by the crowded methyl groups of Cp* ring (**6l**). The reactivity of ruthenocene was further studied. Since its boronic ester is prone to undergo undesired protodeborylation process, a tandem sequence consisting of Ir‐catalyzed C–H borylation of ruthenocene and Pd‐catalyzed Suzuki coupling was established (**6m**) to install a pyridyl group on ruthenocene. Notably, subjecting half sandwich complex CpMn(CO)_3_ to the standard conditions gave rise to borylated product **6n** in 56% yield.

**Figure 4:**
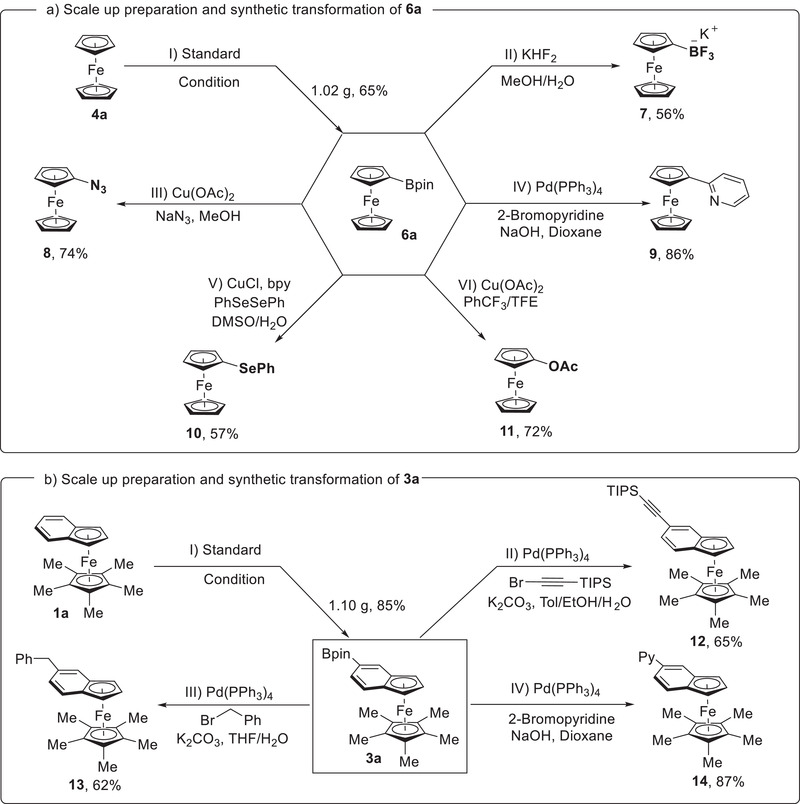
Scale up preparations and synthetic transformations.

To further demonstrate the utility of this method, scale‐up experiments along with derivatizations of boronic esters **3a** and **6a** were performed (**Figure** **4**). Under the established conditions, C–H borylation of benzoferrocene **1a** and ferrocene **4a** could be successfully conducted on 3.0 mmol or 5.0 mmol scale without obvious loss of yield and selectivity. Then, the decoration of the resulting borylated ferrocenes was evaluated. The boronic ester **6a** can be transformed into the potassium trifluoroborate salt **7** with KHF_2_ in 56% yield.^[^ ^16^ ^]^ In the presence of Cu(OAc)_2_, **6a** reacted with sodium azide smoothly to furnish product **8** in 74% yield.^[^ ^7s^ ^]^ Palladium‐catalyzed Suzuki coupling between **6a** and 2‐bromopyridrine led to the formation of compound **9** in 86% yield. With the assistance of 2,2′‐bipyridine and CuCl, a treatment **6a** with diphenyl diselenide easily provided **10** in 57% yield.^[^ ^15d^ ^]^ In addition, **6a** could be acetoxylated to obtain **11** efficiently when using cupric acetate as reactant. Moreover, by adopting Pd‐catalyzed Suzuki coupling strategy, the alkynyl, benzyl and pyridyl groups could be selectively incorporated into benzoferrocene (**12**–**14**).

## 3 Conclusion

In conclusion, we have developed an undirected meta‐selective C–H borylation of metallocenes enabled by steric control strategy. With the help of [Ir(cod)OMe]_2_ and tetramethylphenanthroline or 2,2′‐dipyridylarylmethane ligands, a series of boronic esters based on benzoferrocenes, ferrocenes, ruthenocene and related half sandwich complex were easily accessed through this general approach. Of particular note was C–H borylation of benzoferrocenes, which proceeded exclusively at the benzene ring. This phenomenon mainly results from the shielding effect of bulky pentamethylcyclopentadiene which lowers the reactivity of Cp ring. The salient features include excellent regioselectivity, good functional‐group tolerance, high step‐economy, easy scalability, and diverse synthetic elaborations. We anticipate that this practical diversification protocol will open new avenues for construction of 1,3‐disubstituted metallocenes.

## Conflict of Interest

The authors declare no conflict of interest.

## Supporting information

Supporting Information

## Data Availability

The data that support the findings of this study are available in the supplementary material of this article.
