# Supplementary material for: Catalytic Undirected Meta‐Selective C–H Borylation of Metallocenes
Source: Adv Sci (Weinh). 2023 Aug 26;10(31):2304672. doi: 10.1002/advs.202304672 (PMC10625117; doi:10.1002/advs.202304672)
Supplement: Supplementary file 1 — Supporting Information [file ADVS-10-2304672-s001.pdf]

ADVANCED SCIENCE
Open Access

Supporting Information

for *Adv. Sci.*, DOI 10.1002/advs.202304672

Catalytic Undirected Meta-Selective C–H Borylation of Metallocenes

Hao Zheng, Chang-Hui Liu, Xiao-Yu Wang, Yan Liu, Bing-Zhi Chen, Yan-Cheng Hu and Qing-An Chen**

Supporting Information For:

Catalytic Undirected *meta*-Selective C–H Borylation of Metallocenes

Hao Zheng,[†,‡] Chang-Hui Liu,[†,‡] Xiao-Yu Wang,[†,‡] Yan Liu,[†,‡] Bing-Zhi Chen,[†,‡] Yan-Cheng Hu[†,¶,*] and Qing-An Chen[†,‡,*]

[†]*Dalian Institute of Chemical Physics, Chinese Academy of Sciences, Dalian 116023, People's Republic of China*

[‡]*University of Chinese Academy of Sciences, Beijing 100049, People's Republic of China*

[¶]*School of Chemical Engineering and Technology, China University of Mining and Technology, Xuzhou 221116, Jiangsu, People's Republic of China*

* E-mail: ychu@cumt.edu.cn; qachen@dicp.ac.cn, Web: www.lbcs.dicp.ac.cn

Table of Contents

1. General experimental details S1

2. General procedure for the synthesis of substrates S2

3. Typical procedure for iridium-catalyzed C-H borylation S10

4. Derivatizations and scale-up synthesis S16

5. X-ray Crystal Structures S20

6. Copies of NMR spectra S22

7. References S82

1. General experimental details

Commercially available reagents were used without further purification. Solvents were treated prior to use according to the standard methods. Unless otherwise stated, all reactions were conducted under inert atmosphere using standard Schlenk techniques or in a nitrogen-filled glove-box. ^{1}H NMR, ^{13}C NMR and ^{11}B NMR spectra were recorded at room temperature in $CDCl_3$ or C_6D_6 on 400 MHz or 700MHz instrument with tetramethylsilane (TMS) as internal standard. Flash column chromatography was performed on silica gel (200-300 mesh) or neutral aluminum oxide (200-300 mesh). All reactions were monitored by TLC or NMR analysis. HRMS data was obtained with Agilent 8890-7250 or Agilent 6540 Accurate-MS spectrometer (Q-TOF). Ferrocene **4a**, 1,1'-dimethylferrocene **4b**, 1,1'-diethylferrocene **4c**, 1,1'-dibromoferrocene **4k**, ruthenocene **4m** and $CpMn(CO)_3$ **4n** are commercially available. Benzoferrocenes **1a**, **1b**, **1i** and **1j** were prepared according to literature procedures[1]. The **L8** was prepared according to literature procedure[2].

2. General procedure for the synthesis of substrates

2.1. Synthesis of indenes and cyclopentadienes

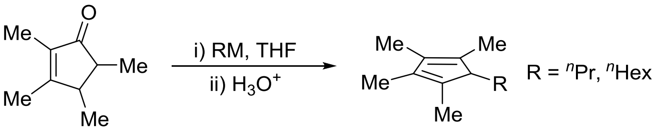

A modification of synthetic protocol from the literature[3]: A solution of the corresponding Grignard reagent (1.5 equiv.) in THF was added to a stirred solution of 2,3,4,5-tetramethyl-2-cyclopentenone (1.0 equiv.) in anhydrous THF. The mixture was refluxed for 24 h, then cooled to 0 ℃ and quenched with HCl. This solution was warmed to room temperature and stirred for 2 h. The mixture was diluted with ethyl acetate and washed with water, and the obtained organic layer was dried over Na_2SO_4 and filtered. The obtained mixture was concentrated under reduced pressure without further purification.

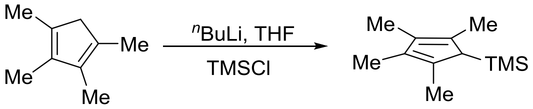

A modification of synthetic protocol from the literature[4]: *n*-BuLi (2.5 M in hexane, 1 equiv.) was added to a stirred solution of 1,2,3,4-tetramethylcyclopenta-1,3-diene (1.0 equiv.) in anhydrous THF at -78 ^oC, were warmed to rt and stirred for 2 h, then cooled to 0 ℃ and quenched with chlorotrimethylsilane (1.5 equiv.). This solution was warmed to room temperature and stirred for 16 h. The mixture was diluted with ethyl acetate and washed with saturated NaCl and water, then the obtained organic layer was dried over Na_2SO_4 and filtered. The obtained mixtures were concentrated under reduced pressure without further purification.

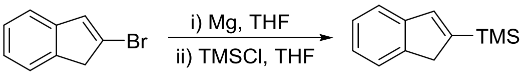

A modification of synthetic protocol from the literature[5]. Magnesium turnings (1.2 equiv.) were placed in a 100 mL flask equipped with an addition funnel and cooled with a water bath. Dry THF (30 mL) was added. In a separate flask, 2-bromoindene (1.0 equiv.) was dissolved in 20 mL THF, and the resulting solution was transferred to the addition funnel. 2 mL of the 2-bromoindene solution was added to the magnesium suspension, and then a small amount of iodine was added to initiate the reaction. Within 15 min, the reaction mixture become cloudy. At this point the remaining 2-bromoindene solution was added dropwise over half an hour. After stirring for an additional 4 h, the unreacted magnesium was filtered off and the Grignard solution was transferred to a second 100 mL flask and cooled with a water-bath. Chlorotrimethylsilane (1.5 equiv.) and THF (10 mL) were then placed in the addition funnel, then this solution was added dropwise over about 30 minutes. The solution was then stirred overnight at room temperature. water (25 mL) was added, followed by ethyl acetate (20 mL), and the obtained organic layer was dried over Na_2SO_4 and filtered. The obtained mixtures were concentrated under reduced pressure without further

purification.

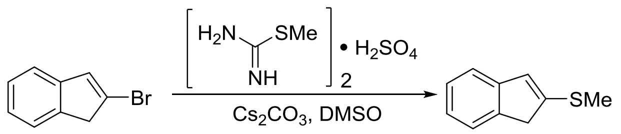

A modification of synthetic protocol from the literature[6]: A mixture of 2-bromoindene (1.95 g, 10 mmol), S-methylisothiourea salts (2.78 g, 10 mmol), cesium carbonate (9.77 g, 30 mmol) and DMSO (20 mL) was reacted in the range of room temperature to 80 ℃ for 5 h. After completion of the reaction, the mixture was poured into water and extracted with dichloromethane (3 ×15 mL). The combined organic layers were dried over anhydrous Na_2SO_4 and filtered. After the removal of the solvent, the residue was purified by flash column chromatography on silica gel using petroleum ether as the eluent to afford the target products, white solid, 1.37 g, 85% yield.

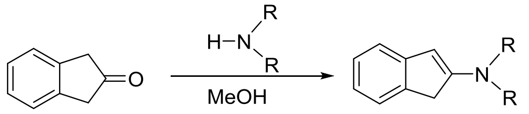

A modification of synthetic protocol from the literature[7]: To a stirred and pre-cooled to 0 °C solution of indan-2-one kept under argon (1.0 equiv.) in dry methanol (25 mL), the corresponding *sec*-amine (1.1 equiv.) as a solution in dry methanol (5.0 mL) was slowly syringed via the septum, the reaction mixture was allowed to warm up slowly to r.t, followed by cooling down to -20 °C in a fridge overnight. The precipitated products were filtered, and washed with 2 portions (10 mL each) of pre-cooled methanol, then filtered and dried under dynamic vacuum.

2.2. Synthesis of benzoferrocenes and ferrocenes

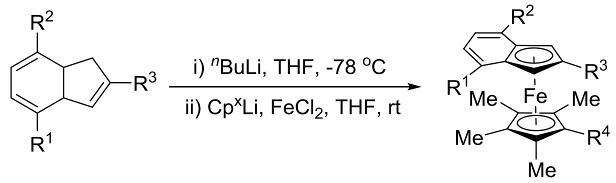

A modification of synthetic protocol from the literature[1]: To a solution of indene (1 equiv.) in THF at -78 °C, *n*-BuLi (2.5 M in hexane, 1.5 equiv.) was added via syringe in a dropwise manner. After addition, the solution was stirred for 1 h -78 °C, warmed to rt slowly and stirred for 2 h. Pentamethylcyclopentadiene (Cp*–H, 1 equiv.) in THF in another round-bottomed flask was treated with *n*-BuLi (2.5 M in hexane, 1.5 equiv.) at -78 °C, the solution was warmed to rt, giving a white suspension after 2 h. This white suspension was added to a suspension of anhydrous $FeCl_2$ (1 equiv.) in THF with vigorous stirring via a cannula. After stirring at rt for 1 h, the solution of lithiated indene was added dropwise via a cannula, and the resulting deep purple solution was stirred at rt. After 2 h, TLC (SiO_2, petroleum ether) indicated quantitative conversion. THF was removed under reduced pressure on a rotary evaporator, the deep purple residue was partitioned between 10% K_2CO_3 and petroleum ether and the organic layer was further washed with 10% K_2CO_3 and water. After drying over anhydrous Na_2SO_4, the deep purple solution was concentrated. The crude product mixture was purified by flash column chromatography (neutral Al_2O_3, petroleum ether) to afford product.

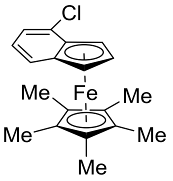

4-Chloroindenyl-pentamethylcyclopentadienyliron (1c): Red solid, mp 126.6-127.5 °C, 2.54 g, 50% yield. **^{1}H NMR** (400 MHz, C_6D_6) δ 7.09 – 6.98 (m, 1H), 6.97 – 6.83 (m, 1H), 6.71 – 6.49 (m, 1H), 4.70 – 4.47 (m, 1H), 4.26 – 4.03 (m, 1H), 3.75 – 3.54 (m, 1H), 1.58 (s, 15H). **^{13}C NMR** (100 MHz, C_6D_6) δ 133.9, 126.5, 122.4, 120.8, 90.7, 88.5, 78.3, 76.8, 67.1, 65.3, 10.0. **HRMS** calculated for $C_{19}H_{22}ClFe$ $[M+H]^+$ 341.0754, found 341.0751.

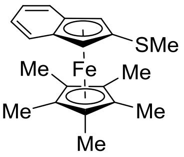

1-Methylthioindenyl-pentamethylcyclopentadienyliron (1k): Red solid, mp 81.1-82.9 °C, 1.17 g, 33% yield. **^{1}H NMR** (400 MHz, $CDCl_3$) δ 7.33 – 7.24 (m, 2H), 7.08 – 6.89 (m, 2H), 4.52 – 4.29 (m, 2H), 2.16 (s, 3H), 1.58 (s, 15H). **^{13}C NMR** (175 MHz, $CDCl_3$) δ 126.7, 123.5, 89.6, 87.0, 78.8, 68.5, 20.9, 9.7. **HRMS** calculated for $C_{20}H_{25}FeS$ $[M+H]^+$ 353.1021, found 353.1017.

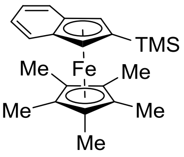

1-Trimethylsilylindenyl-pentamethylcyclopentadienyliron (1l): Purple gum, 1.57 g, 42% yield. **^{1}H NMR** (700 MHz, C_6D_6) δ 7.27 – 7.19 (m, 2H), 7.00 – 6.90 (m, 2H), 4.27 – 4.11 (m, 2H), 1.58 (s, 15H), 0.28 (s, 9H). **^{13}C NMR** (175 MHz, C_6D_6) δ 127.9, 123.4, 91.9, 79.9, 77.3, 69.9, 10.6, 0.4. **HRMS** calculated for $C_{22}H_{31}FeSi$ $[M+H]^+$ 379.1539, found 379.1535.

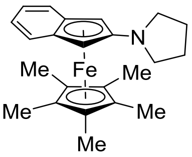

1-(Pyrrolidin-1-yl)indenyl-pentamethylcyclopentadienyliron (1m): Purple solid, mp 128.1-128.9 °C, 2.44 g, 65% yield. **^{1}H NMR** (700 MHz, C_6D_6) δ 7.32 – 7.27 (m, 2H), 7.01 – 6.96 (m, 2H), 3.83 – 3.77 (m, 2H), 2.90 – 2.84 (m, 4H), 1.67 (s, 15H), 1.62 – 1.57 (m, 4H). **^{13}C NMR** (175 MHz, C_6D_6) δ 129.0, 121.8, 116.2, 85.9, 76.5, 51.9, 49.1, 25.6, 10.6. **HRMS** calculated for $C_{23}H_{30}FeN$ $[M+H]^+$ 376.1722, found 376.1726.

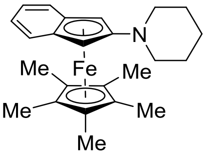

1-(Piperidin-1-yl)indenyl-pentamethylcyclopentadienyliron (1n): Purple solid, mp 137.5-139.2 °C, 2.03 g, 52% yield. **^{1}H NMR** (700 MHz, C_6D_6) δ 7.30 – 7.20 (m, 2H), 7.01 – 6.90 (m, 2H), 4.02 – 3.88 (m, 2H), 2.75 – 2.57 (m, 4H), 1.67 (s, 15H), 1.52 – 1.41 (m, 4H), 1.35 – 1.27 (m, 2H). **^{13}C NMR** (175 MHz, C_6D_6) δ 128.6, 122.2, 118.1, 86.1, 76.8, 53.7, 51.4, 26.0, 24.7, 10.2. **HRMS** calculated for $C_{24}H_{32}FeN$ $[M+H]^+$ 390.1879, found 390.1873.

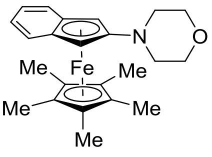

1-Morpholinoindenyl-pentamethylcyclopentadienyliron (1o): Purple solid, mp 108.5-109.8 °C, 2.16 g, 55% yield. **^{1}H NMR** (700 MHz, C_6D_6) δ 7.30 – 7.19 (m, 2H), 7.02 – 6.88 (m, 2H), 3.96 – 3.77 (m, 2H), 3.71 – 3.49 (m, 4H), 2.63 – 2.46 (m, 4H), 1.63 (s, 15H). **^{13}C NMR** (175 MHz, C_6D_6) δ 128.4, 122.5, 117.14, 86.0, 76.9, 66.8, 53.4, 50.7, 10.2. **HRMS** calculated for $C_{23}H_{30}FeNO$ $[M+H]^+$ 392.1671, found 392.1668.

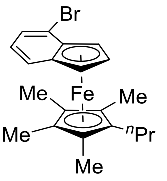

4-Bromoindenyl-1,2,3,4-tetramethyl-5-propylcyclopenta-1,3-dienyliron (1p): Purple liquid, 1.42 g, 33% yield. **^{1}H NMR** (700 MHz, $CDCl_3$) δ 7.30 – 7.05 (m, 2H), 6.90 – 6.61 (m, 1H), 4.69 – 4.48 (m, 1H), 4.48 – 4.31 (m, 1H), 4.00 – 3.71

(m, 1H), 2.36 – 2.10 (m, 2H), 1.66 (s, 2H), 1.31 – 1.19 (m, 12H), 0.96 – 0.78 (m, 3H). **^{13}C NMR** (175 MHz, $CDCl_3$) δ 127.1, 124.2, 123.0, 122.4, 90.0, 89.7, 82.8, 78.6, 78.25, 78.18, 76.4, 67.0, 66.5, 28.0, 24.1, 14.3, 10.2, 10.1, 10.0. **HRMS** calculated for $C_{21}H_{26}BrFe$ $[M+H]^+$ 413.0562, found 413.0562.

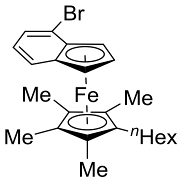

4-Bromoindenyl-5-hexyl-1,2,3,4-tetramethylcyclopenta-1,3-dienyliron (1q): Purple liquid, 1.38 g, 30% yield. **^{1}H NMR** (400 MHz, $CDCl_3$) δ 7.29 (d, *J* = 8.6 Hz, 1H), 7.22 – 7.17 (m, 1H), 6.82 – 6.75 (m, 1H), 4.55 – 4.50 (m, 1H), 4.42 – 4.37 (m, 1H), 3.87 – 3.81 (m, 1H), 2.28 – 2.18 (m, 2H), 1.73 – 1.60 (m, 12H), 1.29 – 1.22 (m, 6H), 1.21 – 1.15 (m, 2H), 0.91 – 0.86 (m, 3H). **^{13}C NMR** (100 MHz, $CDCl_3$) δ 127.1, 124.2, 123.0, 122.4, 90.0, 89.7, 83.1, 78.6, 78.14, 78.10, 76.4, 67.0, 66.5, 31.9, 31.1, 29.5, 26.0, 22.8, 14.2, 10.14, 10.10, 10.0. **HRMS** calculated for $C_{24}H_{32}BrFe$ $[M+H]^+$ 455.1031, found 455.1036.

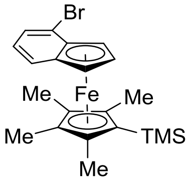

4-Bromoindenyl-1,2,3,4-tetramethyl-5-trimethylsilylcyclopenta-1,3-dienyliron (1r): Purple gum, 1.00 g, 22% yield. **^{1}H NMR** (400 MHz, C_6D_6) δ 7.12 – 7.05 (m, 2H), 6.50 (t, *J* = 7.7 Hz, 1H), 4.73 – 4.66 (m, 1H), 4.46 – 4.39 (m, 1H), 3.82 – 3.75 (m, 1H), 1.78 (s, 3H), 1.54 (s, 3H), 1.51 (s, 3H), 1.45 (s, 3H), 0.40 (s, 9H). **^{13}C NMR** (100 MHz, C_6D_6) δ 127.4, 125.0, 123.4, 123.0, 90.7, 90.0, 84.0, 83.1, 82.9, 75.6, 66.5, 66.1, 65.4, 13.3, 12.5, 9.8, 9.4, 2.3. **HRMS** calculated for $C_{21}H_{27}BrFeSi$ $[M]^+$ 442.0415, found 442.0418.

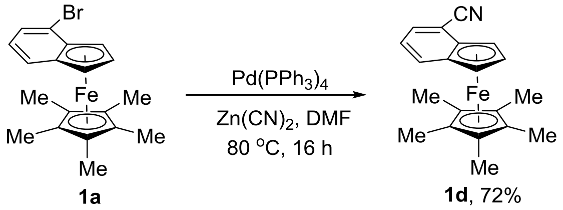

4-Cyanoindenyl-pentamethylcyclopentadienyliron (1d)[8]: 1a (385.1 mg, 1.0 mmol), $Pd(PPh_3)_4$ (115.6 mg, 0.10 mmol), $Zn(CN)_2$ (117.4 mg, 0.10 mmol) and DMF (5 mL) was added to a 15 mL sealed tube. The mixture was stirred at 80 °C for 16 h. After completion of the reaction, the mixture was cooled to rt and poured into water and extracted with ethyl acetate (3 ×10 mL). The combined organic layers were dried over anhydrous Na_2SO_4 and filtered. After the removal of the solvent, the residue was purified by flash column chromatography (silica gel, petroleum ether:ethyl acetate = 100:1) to afford product as a purple solid, mp 140.3-142.1 °C, 237.3 mg, 87% yield. **^{1}H NMR** (700 MHz, C_6D_6) δ 7.17 – 7.10 (m, 1H), 7.05 – 6.94 (m, 1H), 6.50 – 6.38 (m, 1H), 4.70 – 4.55 (m, 1H), 4.18 – 4.02 (m, 1H), 3.73 – 3.55 (m, 1H), 1.49 (s, 15H). **^{13}C NMR** (175 MHz, C_6D_6) δ 135.0, 130.3, 120.8, 118.7, 110.6, 88.2, 87.7, 78.5, 78.1, 67.1, 65.8, 10.0. **HRMS** calculated for $C_{20}H_{22}FeN$ $[M+H]^+$ 332.1096, found 332.1082.

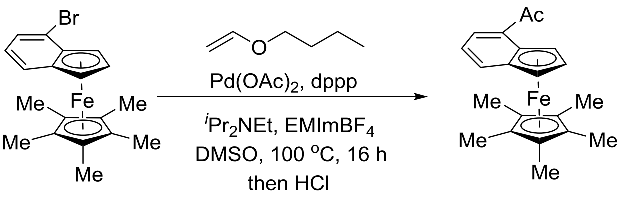

4-Acetylindenyl-pentamethylcyclopentadienyliron (1e)[9]: To a 15 mL sealed tube was sequentially added **1a** (385.1 mg, 1.0 mmol), $Pd(OAc)_2$ (11.2 mg, 0.05 mmol), dppp (41.2 mg, 0.04 mmol), DMSO (5.0 mL), $EMImBF_4$ (0.5 mL) and EtN^iPr_2 (258.5 mg, 2.0 mmol). The resulting mixture was stirred at 100 °C for 16 h. the mixture was cooled to rt, the 2 M HCl (5 mL, 10.0 mmol) solution was added to the mixture and stirred for 30 min, then poured into water and extracted with ethyl acetate (3 ×10 mL). The combined organic layers were dried over anhydrous Na_2SO_4 and filtered. After the removal of the solvent, the residue was purified by flash column chromatography (silica gel, petroleum ether:ethyl acetate = 20:1) to afford product as a green solid, mp 97.1-98.9 °C, 536.1 mg, 77% yield. **^{1}H NMR** (400 MHz, $CDCl_3$) δ 7.87 – 7.71 (m, 1H), 7.69 – 7.58 (m, 1H), 7.12 – 6.94 (m, 1H), 5.57 – 5.36 (m, 1H), 4.50 – 4.25 (m, 1H), 4.09 – 3.84 (m, 1H), 2.65 (s, 3H), 1.57 (s, 15H). **^{13}C NMR** (100 MHz, $CDCl_3$) δ 198.7, 137.3, 135.4, 129.3, 120.4, 88.6, 86.0, 78.8, 78.2, 68.8, 66.2, 27.1, 10.1. **HRMS** calculated for $C_{21}H_{25}FeO$ $[M+H]^+$ 349.1249, found 349.1241.

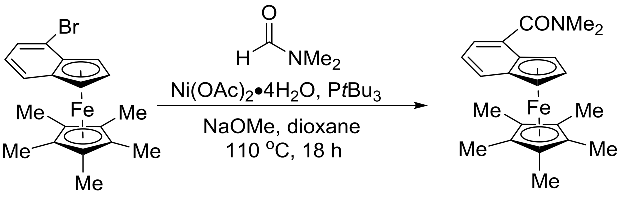

4-Dimethylcarbamoylindenyl-pentamethylcyclopentadienyliron (1f)[10]: To a 15 mL sealed tube was sequentially added, $Ni(OAc)_2 \cdot 4H_2O$ (24.9 mg, 0.10 mmol), P^tBu_3 (20.2 mg, 0.10 mmol) and dioxane (5.0 mL), the mixture was stirred for 30 min and then **1a** (385.1 mg, 1.0 mmol), DMF (1.0 mL) and NaOMe (216.1 mg, 4.0 mmol) were introduced. The resulting mixture was stirred at 110 °C for 18 h. the mixture was cooled to rt, then poured into water and extracted with ethyl acetate (3 ×10 mL). The combined organic layers were dried over anhydrous Na_2SO_4 and filtered. After the removal of the solvent, the residue was purified by flash column chromatography (silica gel, petroleum ether:ethyl acetate = 10:1) to afford product as a purple solid, mp 83.2-84.3 °C, 252.4 mg, 67% yield. **^{1}H NMR** (400 MHz, C_6D_6) δ 7.27 (d, J = 8.6 Hz, 1H), 7.14 – 7.10 (m, 1H), 6.86 – 6.78 (m, 1H), 4.44 – 4.37 (m, 1H), 4.25 – 4.18 (m, 1H), 3.79 – 3.70 (m, 1H), 2.56 (s, 6H), 1.70 (s, 15H). **^{13}C NMR** (100 MHz, C_6D_6) δ 170.1, 135.0, 129.5, 121.9, 121.3, 88.1, 87.5, 78.1, 76.7, 65.6, 64.9, 36.7, 9.7. **HRMS** calculated for $C_{22}H_{28}FeNO$ $[M+H]^+$ 378.1515, found 378.1510.

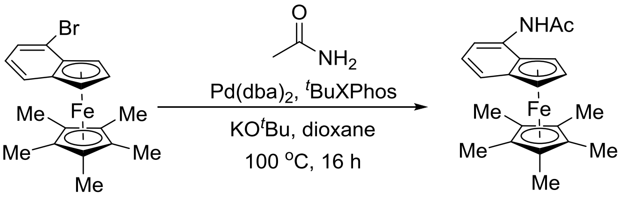

4-Acetamidoindenyl-pentamethylcyclopentadienyliron (1g): To a 15 mL sealed tube was sequentially added, Pd(dba)$_2$ (28.7 mg, 0.05 mmol), ^{t}BuXPhos (42.4 mg, 0.10 mmol) and dioxane (5.0 mL), the mixture was stirred for 30 min and then **1a** (385.1 mg, 1.0 mmol) and KO^{t}Bu (336.6 mg, 3.0 mmol) were introduced. The resulting mixture was stirred at 100 °C for 16 h. The mixture was cooled to rt, then poured into water and extracted with ethyl acetate (3 ×10 mL). The combined organic layers were dried over anhydrous Na_2SO_4 and filtered. After the removal of the solvent, the residue was purified by flash column chromatography (neutral Al_2O_3, petroleum ether:ethyl acetate = 5:1) to afford product as a purple solid, mp 181.4-182.8 °C (decomp.), 197.3 mg, 54% yield. **^{1}H NMR** (700 MHz, $CDCl_3$) δ 7.47 (s, 1H), 7.20 – 7.05 (m, 1H), 6.96 – 6.75 (m, 1H), 6.74 – 6.50 (m, 1H), 4.71 – 4.27 (m, 2H), 3.98 – 3.64 (m, 1H), 2.25 (s, 3H), 1.46 (s, 15H). **^{13}C NMR** (175 MHz, $CDCl_3$) δ 168.1, 136.1, 123.8, 122.9, 111.6, 90.7, 87.8, 83.2, 78.8, 76.4, 66.1, 60.4, 24.9, 9.9. **HRMS** calculated for $C_{21}H_{26}FeNO$ $[M+H]^+$ 364.1358, found 364.1358.

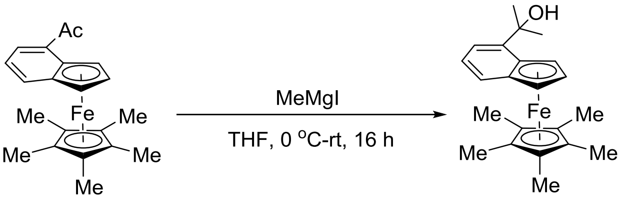

4-(2-Hydroxypropan-2-yl)indenyl-pentamethylcyclopentadienyliron (1h): To a solution of **1e** (1 equiv.) in THF at 0 °C, *n*-BuLi (2.5 M in hexane, 1.5 equiv.) was added via syringe in a dropwise manner. After addition, the solution was stirred for 1 h at -78 °C, warmed to rt slowly and stirred for 16 h. The products were concentrated under reduced pressure and the residue was purified by flash column chromatography (silica gel, petroleum ether:ethyl acetate = 50:1) to afford product as a red solid, mp 75.3-76.1 °C, 0.67 g, 48% yield. **^{1}H NMR** (400 MHz, C_6D_6) δ 7.21 – 7.09 (m, 1H), 6.93 – 6.79 (m, 2H), 4.93 – 4.80 (m, 1H), 4.24 – 4.12 (m, 1H), 3.69 – 3.59 (m, 1H), 2.29 (s, 1H), 1.64 (s, 3H), 1.57 (s, 18H). **^{13}C NMR** (100 MHz, C_6D_6) δ 146.1, 127.0, 122.0, 117.4, 89.1, 87.2, 77.3, 76.3, 73.5, 66.5, 66.2, 30.8, 29.9, 10.0. **HRMS** calculated for $C_{22}H_{29}FeO$ $[M+H]^+$ 365.1562, found 365.1562.

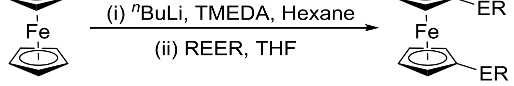

A modification of general procedure from the literature[11]: TMEDA (2.5 equiv.) and ^{n}BuLi (2.5 equiv., 2.5 M) were added dropwise to a stirred solution of **1a** (1.0 equiv.) in hexane under a dry N_2 atmosphere at rt. The solution was stirred at room temperature overnight. The orange slurry was allowed to settle and the hexane layer was removed by a syringe. The remaining orange

powder was washed with dry hexane and dissolved in dry THF. Electrophile was added to the orange solution at -78 °C. The solution was slowly warmed to room temperature until the reaction was complete as indicated by TLC and the reaction was quenched after the given time by the addition of water. The reaction mixture was diluted with ethyl acetate. The layers were separated and the aqueous was extracted thoroughly. The combined organic fractions were dried over Na_2SO_4 and evaporated to give the crude product, and the residue was purified by flash column chromatography (silica gel, petroleum ether:ethyl acetate) to afford respective disubstituted ferrocenes.

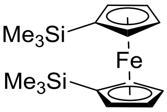

1,1'-Bis(trimethylsilyl)ferrocene (4d): known compound[11], red oil, 2.43 g, 71% yield. **^{1}H NMR** (400 MHz, $CDCl_3$) δ 4.29 (t, J = 1.7 Hz, 4H), 4.07 (t, J = 1.7 Hz, 4H), 0.23 (s, 18H).

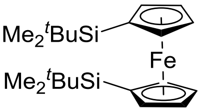

1,1'-Bis(*tert*-butyldimethylsilyl)ferrocene (4e): Yellow solid, mp 91.5-92.3 °C, 1.79 g, 43% yield. **^{1}H NMR** (400 MHz, $CDCl_3$) δ 4.28 – 4.26 (m, 4H), 4.04 – 4.01 (m, 4H), 0.79 (s, 12H), 0.25 (s, 18H). **^{13}C NMR** (175 MHz, $CDCl_3$) δ 73.7 71.3, 69.4 26.5, 17.2, -5.5. **HRMS** calculated for $C_{22}H_{38}FeSi_2$ $[M]^+$ 414.1861, found 414.1861.

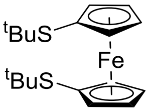

1,1'-Bis(*tert*-butylthio)ferrocene (4g): Yellow solid, mp 85.6-87.3 °C, 0.43 g, 12% yield. **^{1}H NMR** (700 MHz, $CDCl_3$) δ 4.32 – 4.29 (m, 4H), 4.27 – 4.24 (m, 4H), 1.17 (s, 18H). **^{13}C NMR** (175 MHz, $CDCl_3$) δ 77.5, 77.0, 71.6, 45.2, 30.8. **HRMS** calculated for $C_{18}H_{27}FeS_2$ $[M+H]^+$ 363.0898, found 363.0897.

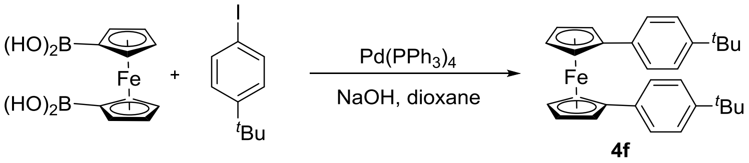

1,1'-Bis((4''-*tert*-butyl)phenyl)ferrocene (4f): A mixture of boronic acid (1.09 g, 4 mmol), $Pd(PPh_3)_4$ (290.3 mg, 0.40 mmol), dioxane (30 mL), ArI (3.12 g, 12 mmol) and 3 M NaOH (6 mL). The mixture was stirred at 105 °C for 48 h. The mixture was cooled to rt and diluted with ethyl acetate. The layers were separated and the aqueous layer was extracted with ethyl acetate. The combined organic layers were washed with saturated NaCl, dried with Na_2SO_4 and concentrated under vacuum. The residue was purified by flash column chromatography (silica gel, petroleum ether: ethyl acetate = 200:1) to afford product as an orange red gum, 397.4 mg, 22% yield. **^{1}H NMR** (400 MHz, $CDCl_3$) δ 7.29 – 7.27 (m, 3H), 7.27 – 7.25 (m, 3H), 7.25 – 7.23 (m, 1H), 7.21 – 7.19 (m, 1H), 4.45 – 4.42 (m, 4H), 4.16 – 4.12 (m, 4H), 1.33 (s, 18H). **^{13}C NMR** (100 MHz, $CDCl_3$) δ 148.8, 135.5, 128.3, 126.1, 126.0, 125.9, 125.21, 125.19, 70.7, 70.6, 70.4, 67.9, 67.84, 67.80, 34.6, 31.5. **HRMS** calculated for $C_{30}H_{35}Fe$ $[M+H]^+$ 451.2083, found 451.2079.

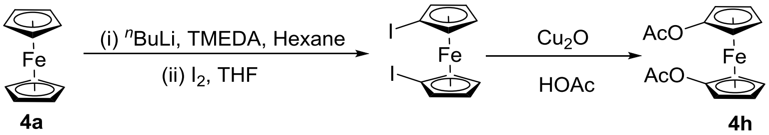

1,1'-Diacetoxyferrocene (4h)[12]: TMEDA (2.5 equiv.) and ^{n}BuLi (2.5 equiv., 2.5 M) were added dropwise to a stirred solution of **1a** (1.0 equiv.) in hexane under a dry N_2 atmosphere at rt. The solution was stirred at rt overnight. The orange slurry was allowed to settle and the hexane layer was removed by a syringe. The remaining orange powder was washed with dry hexane and dissolved in dry THF. I_2 was added to the orange solution at -78 °C. The solution was slowly warmed to room temperature until the reaction was complete as indicated by TLC and the reaction was quenched after the given time by the addition of water. The reaction mixture was diluted with ethyl acetate. The layers were separated and the aqueous was extracted thoroughly. The combined organic fractions were dried over Na_2SO_4 and evaporated to give the crude product without further purification. To a 100 mL sealed tube was sequentially added, 1,1-diiodoferrocene, Cu_2O (12.7 g), HOAc (2.5 mL), MeCN (60 mL) and PhH (10 mL). The mixture was stirred at 90 °C for 36 h. After cooling down to room temperature, the reaction mixture was concentrated under reduced pressure. Water was added and the mixture was extracted with EtOAc. The combined organic layers were dried over anhydrous Na_2SO_4, filtered, and concentrated under reduced pressure. The residue was purified by flash column chromatography (silica gel, petroleum ether:ethyl acetate = 50:1) to afford product as a yellow solid, mp 51.2-52.4 °C, 0.27 g, 9% yield. **^{1}H NMR** (400 MHz, $CDCl_3$) δ 4.60 – 4.35 (m, 4H), 4.12 – 3.90 (m, 4H), 2.17 (s, 6H). **^{13}C NMR** (100 MHz, $CDCl_3$) δ 169.1, 116.1, 64.6, 62.1, 21.3. **HRMS** calculated for $C_{14}H_{15}FeO_4$ $[M+H]^+$ 303.0314, found 303.0318.

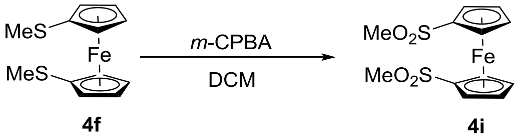

1,1'-Bis(methylsulfonyl)ferrocene (4i): To a solution of **4f** (834.6 mg, 3.0 mmol) in DCM (20 mL) at 0 °C, *m*-CPBA (2.07 g, 12.0 mmol) was added in portions. After addition, the solution was slowly warmed to rt and stirred for 16 h, saturated NaOH solution was added to above mixture and the organic layer was further washed with 2 M HCl and saturate $NaHCO_3$. The combined organic layers were dried over anhydrous Na_2SO_4, filtered, and concentrated under reduced pressure. The residue was purified by flash column chromatography (silica gel, dichloromethane:ethyl acetate = 6:1) to afford product as a yellow solid. 748.1 mg, 73% yield. **^{1}H NMR** (400 MHz, $CDCl_3$) δ 4.89 (t, J = 2.0 Hz, 4H), 4.82 (t, J = 2.0 Hz, 4H), 3.00 (s, 6H). **^{13}C NMR** (100 MHz, $CDCl_3$) δ 91.0, 74.2, 71.5, 45.1. **HRMS** calculated for $C_{12}H_{15}FeO_4S_2$ $[M+H]^+$ 342.9756, found 342.9758.

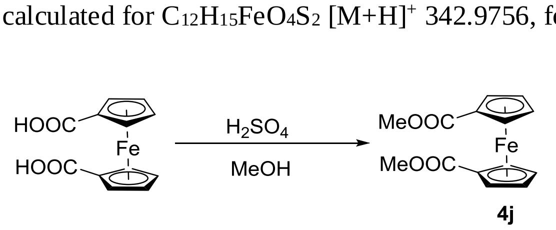

1,1'-Bis(methoxycarbonyl) ferrocene (4j)[13]: To a solution of ferrocenecarboxylic acid (618.1 mg, 2.5 mmol) in MeOH (25 mL), concentrated H_2SO_4 (0.5 mL) was added in dropwise manner. After addition, the solution was refluexd warmed to rt and stirred for 16 h, After cooled to rt, saturate $NaHCO_3$ solution was added to above mixture and the organic layer was further washed with saturate NaCl solution. The combined organic layers were dried over anhydrous Na_2SO_4, filtered, and concentrated under reduced pressure. The residue was purified by flash column

chromatography (silica gel, petroleum ether:ethyl acetate = 50:1) to afford product as a yellow solid, known compound, 401.2 mg, 53%. **^{1}H NMR** (400 MHz, $CDCl_3$) δ 4.87 – 4.76 (m, 4H), 4.46 – 4.33 (m, 4H), 3.80 (s, 6H). **^{13}C NMR** (100 MHz, $CDCl_3$) δ 170.8, 72.9, 72.6, 71.6, 51.7.

3. Typical procedure for iridium-catalyzed C-H borylation

3.1. Typical procedure for iridium-catalyzed C-H borylation of benzoferrocenes

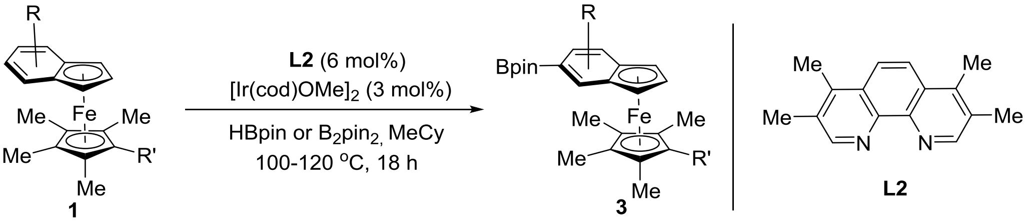

To a 5 mL sealed tube was sequentially added, **1** (0.20 mmol, 1.0 equiv.), $[Ir(cod)OMe]_2$ (4.0 mg, 0.006 mmol), **L2** (2.8 mg, 0.012 mmol), MeCy (1.0 mL) and HBpin (128.0 mg, 1.0 mmol) or B_2pin_2 (76.2 mg, 0.30 mmol). The resulting mixture was stirred at 100 °C or 120 °C for 18 h. The mixture was cooled to rt. After the removal of the solvent, the residue was purified by chromatography (petroleum ether: ethyl acetate = 30:1) to afford product.

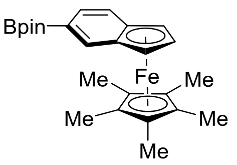

5-(4,4,5,5-Tetramethyl-1,3,2-dioxaborolan-2-yl)indenyl-pentamethyl cyclopentadienyliron (3a): Purple solid, mp 110.0-111.7 °C, 81.8 mg, 95%. **^{1}H NMR** (400 MHz, C_6D_6) δ 8.35 (s, 1H), 7.76 (d, *J* = 8.7 Hz, 1H), 7.26 (d, *J* = 8.7 Hz, 1H), 4.24 (d, *J* = 2.4 Hz, 2H), 3.71 (t, *J* = 2.5 Hz, 1H), 1.56 (s, 15H), 1.20 (s, 12H). **^{13}C NMR** (100 MHz, $CDCl_3$) δ 139.3, 126.6, 125.6, 90.1, 87.2, 83.5, 78.4, 66.5, 66.4, 25.1, 24.8, 10.1. **^{11}B NMR** (128 MHz, $CDCl_3$) δ 30.8. **HRMS** calculated for $C_{25}H_{34}BFeO_2$ $[M+H]^+$ 433.1996, found 433.1994.

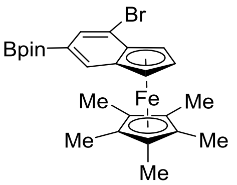

4-Bromo-6-(4,4,5,5-tetramethyl-1,3,2-dioxaborolan-2-yl)indenyl-pentamethylcyclopentadienyl iron (3b): Purple solid, mp 148.9-149.5 °C, 96.5 mg, 94% yield. **^{1}H NMR** (400 MHz, $CDCl_3$) δ 7.93 (s, 1H), 7.52 (s, 1H), 4.64 – 4.50 (m, 1H), 4.48 – 4.30 (m, 1H), 4.01 – 3.84 (m, 1H), 1.65 (s, 15H), 1.36 (s, 12H). **^{13}C NMR** (100 MHz, $CDCl_3$) δ 138.8, 127.8, 121.6, 91.2, 88.3, 83.8, 78.6, 77.5, 67.7, 67.5, 25.1, 24.7, 10.0. **^{11}B NMR** (128 MHz, $CDCl_3$) δ 30.0. **HRMS** calculated for $C_{25}H_{33}BBrFeO_2$ $[M+H]^+$ 511.1101, found 511.1098.

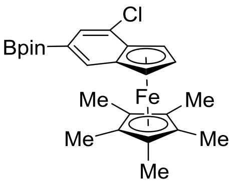

4-Chloro-6-(4,4,5,5-tetramethyl-1,3,2-dioxaborolan-2-yl)indenyl-pentamethylcyclopentadienyliron (3c): Red solid, mp 149.3-150.7 °C, 90.2 mg, 97% yield. **^{1}H NMR** (400 MHz, $CDCl_3$) δ 7.86 (s, 1H), 7.30 (s, 1H), 4.68 – 4.52 (m, 1H), 4.45 – 4.30 (m, 1H), 3.98 – 3.85 (m, 1H), 1.62 (s, 15H), 1.35 (s, 12H). **^{13}C NMR** (100 MHz, $CDCl_3$) δ 138.0, 132.1, 124.2, 89.6, 88.6, 83.8, 78.6, 77.5, 67.5, 65.8, 25.1, 24.7, 9.9. **^{11}B NMR** (128 MHz, $CDCl_3$) δ 31.3. **HRMS** calculated for $C_{25}H_{33}BClFeO_2$ $[M+H]^+$ 467.1606, found 467.1607.

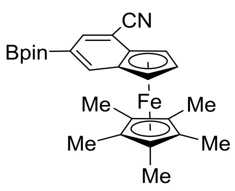

4-Cyano-6-(4,4,5,5-tetramethyl-1,3,2-dioxaborolan-2-yl)indenyl-pentamethylcyclopentadienyliron (3d): Purple solid, mp 195.7-197.4 °C (decomp.), 43.1 mg, 47% yield. **^{1}H NMR** (700 MHz, C_6D_6) δ 8.36 (s, 1H), 8.06 (s, 1H), 4.73 – 4.64 (m, 1H), 4.10 (d, *J* = 2.2 Hz, 1H), 3.68 (t, *J* = 2.4 Hz, 1H), 1.46 (s, 15H), 1.15 (s, 12H). **^{13}C NMR** (175 MHz, C_6D_6) δ 146.4, 135.2, 118.9, 109.4, 89.2, 86.5, 83.9, 79.0, 78.8, 67.8, 66.8, 25.0, 24.8, 9.9. **^{11}B NMR** (128 MHz, C_6D_6) δ 31.2. **HRMS** calculated for $C_{26}H_{33}BFeNO_2$ $[M+H]^+$ 458.1948, found 458.1945.

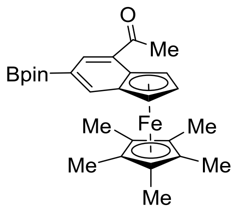

4-Acetyl-6-(4,4,5,5-tetramethyl-1,3,2-dioxaborolan-2-yl)indenyl-pentamethylcyclopentadienyliron (3e): Green solid, mp 201.2-202.8 °C, 24.8 mg, 26% yield. **^{1}H NMR** (700 MHz, C_6D_6) δ 8.53 (s, 1H), 8.32 (s, 1H), 5.96 – 5.92 (m, 1H), 4.21 – 4.18 (m, 1H), 3.88 (t, *J* = 2.5 Hz, 1H), 2.34 (s, 3H), 1.51 (s, 15H), 1.21 (s, 12H). **^{13}C NMR** (175 MHz, C_6D_6) δ 197.5, 148.2, 134.4, 133.1, 87.5, 87.3, 83.4, 79.7, 77.9, 70.3, 66.7, 26.1, 24.7, 24.5, 9.7. **^{11}B NMR** (128 MHz, C_6D_6) δ 30.6. **HRMS** calculated for $C_{27}H_{36}BFeO_3$ $[M+H]^+$ 475.2101, found 475.2106.

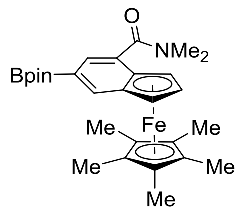

4-Dimethylcarbamoyl-6-(4,4,5,5-tetramethyl-1,3,2-dioxaborolan-2-yl)-7-bromoindenyl-pentamethylcyclopentadienyliron (3f): Red gum, 65.1 mg, 64% yield. **^{1}H NMR** (700 MHz, C_6D_6) δ 8.41 (s, 1H), 7.89 (s, 1H), 4.58 – 4.53 (m, 1H), 4.27 – 4.21 (m, 1H), 3.80 (t, *J* = 2.4 Hz, 1H), 3.08 – 2.56 (m, 3H), 2.53 – 2.27 (m, 3H), 1.69 (s, 15H), 1.19 (s, 12H). **^{13}C NMR** (175 MHz, C_6D_6) δ 170.7, 142.3, 133.4, 126.5, 89.5, 87.1, 83.6, 78.7, 78.1, 66.7, 66.3, 38.1, 35.4, 25.0, 24.9, 10.0. **^{11}B NMR** (128 MHz, C_6D_6) δ 32.5. **HRMS** calculated for $C_{28}H_{39}BFeNO_3$ $[M+H]^+$ 504.2367, found 504.2364.

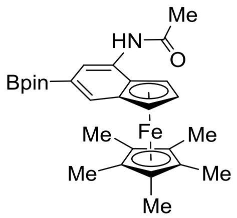

4-Acetamidoindenyl-6-(4,4,5,5-tetramethyl-1,3,2-dioxaborolan-2-yl)indenyl-pentamethylcyclopentadienyliron (3g): Purple solid, mp 187.1-190.0 °C (decomposed), 43.2 mg, 43% yield (accompanied by a small amount of ethyl acetate, the yield of the product has been adjusted accordingly). **^{1}H NMR** (400 MHz, C_6D_6) δ 8.72 (s, 1H), 8.05 (s, 1H), 7.49 (s, 1H), 4.47 – 4.35 (m, 1H), 4.32 – 4.21 (m, 1H), 3.80 – 3.67 (m, 1H), 1.99 (s, 3H), 1.58 (s, 15H), 1.16 (s, 6H), 1.16 (s, 6H). **^{13}C NMR** (175 MHz, $CDCl_3$) δ 168.0, 135.2, 134.5, 123.3, 115.6, 89.0, 85.0, 83.5, 81.4, 78.7, 66.8, 61.8, 25.0, 24.8, 24.7, 9.8. **^{11}B NMR** (128 MHz, C_6D_6) δ 32.5. **HRMS** calculated for $C_{27}H_{37}BFeNO_3$. $[M+H]^+$ 490.2210, found 490.2216.

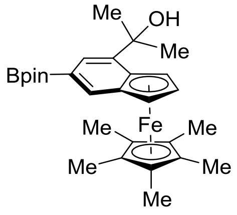

4-(2-Hydroxypropan-2-yl)-6-(4,4,5,5-tetramethyl-1,3,2-dioxaborolan-2-yl)indenyl-pentamethylcyclopentadienyliron (3h): Red gum, 77.9 mg, 76% yield (accompanied by a small amount of ethyl acetate, the yield of the product has been adjusted accordingly). **^{1}H NMR** (400 MHz, C_6D_6) δ 8.30 (s, 1H), 7.73 (s, 1H), 4.99 – 4.93 (m, 1H), 4.24 – 4.19 (m, 1H), 3.72 (t, *J* = 2.4 Hz, 1H), 2.14 (s, 1H), 1.68 (s, 3H), 1.57 (s, 15H), 1.56 (s, 3H), 1.21

(s, 6H), 1.20 (s, 6H). **^{13}C NMR** (100 MHz, C_6D_6) δ 144.6, 140.1, 121.6, 89.3, 88.1, 83.4, 77.9, 77.7, 74.0, 67.8, 67.4, 31.1, 30.2, 25.2, 24.9, 10.3. **^{11}B NMR** (128 MHz, C_6D_6) δ 32.1. **HRMS** calculated for $C_{28}H_{40}BFeO_3$ $[M+H]^+$ 491.2414, found 491.2415.

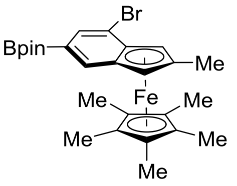

4-Bromo-1-methyl-6-(4,4,5,5-tetramethyl-1,3,2-dioxaborolan-2-yl)indenyl-pentamethylcyclopentadienyliron (3i): Purple solid, mp 157.5-158.8 °C, 81.7 mg, 78% yield. **^{1}H NMR** (700 MHz, C_6D_6) δ 8.20 (s, 1H), 8.03 (s, 1H), 4.40 (s, 1H), 3.99 (s, 1H), 1.72 (s, 3H), 1.55 (s, 15H), 1.16 (s, 12H). **^{13}C NMR** (175 MHz, C_6D_6) δ 139.5, 128.5, 122.2, 92.0, 89.7, 89.1, 83.6, 78.0, 67.9, 67.9, 25.1, 24.8, 13.4, 9.7. **^{11}B NMR** (128 MHz, C_6D_6) δ 33.7. **HRMS** calculated for $C_{26}H_{35}BBrFeO_2$ $[M+H]^+$ 525.1257, found 525.1254.

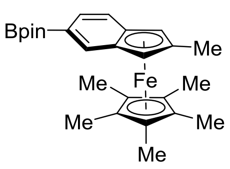

1-Methyl-5-(4,4,5,5-tetramethyl-1,3,2-dioxaborolan-2-yl)indenyl-pentamethylcyclopentadienyliron (3j): Purple solid, mp 87.9-89.2 °C, 81.1 mg, 91% yield. **^{1}H NMR** (400 MHz, $CDCl_3$) δ 7.73 (s, 1H), 7.09 (d, J = 8.3 Hz, 1H), 7.00 (d, J = 9.0 Hz, 1H), 4.60 – 4.30 (m, 2H), 1.80 (s, 3H), 1.38 (s, 27H). **^{13}C NMR** (100 MHz, $CDCl_3$) δ 140.1, 126.7, 126.3, 92.0, 90.4, 88.4, 83.3, 77.9, 65.9, 25.0, 24.6, 13.3, 9.5. **^{11}B NMR** (128 MHz, $CDCl_3$) δ 30.6. **HRMS** calculated for $C_{26}H_{36}BFeO_2$ $[M+H]^+$ 447.2152, found 447.2149.

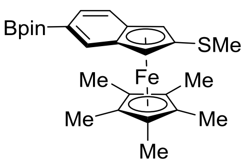

1-Methylthio-5-(4,4,5,5-tetramethyl-1,3,2-dioxaborolan-2-yl)indenyl-pentamethylcyclopentadienyliron (3k): Red gum, 80.1 mg, 84% yield. **^{1}H NMR** (700 MHz, $CDCl_3$) δ 7.94 (s, 1H), 7.43 – 7.31 (m, 1H), 7.30 – 7.16 (m, 1H), 4.44 (s, 2H), 2.22 (s, 3H), 1.62 (s, 15H), 1.40 (s, 12H). **^{13}C NMR** (175 MHz, $CDCl_3$) δ 138.6, 127.3, 125.0, 91.1, 88.0, 83.6, 78.9, 77.3, 69.0, 25.1, 24.8, 20.7, 9.6. **^{11}B NMR** (128 MHz, C_6D_6) δ 30.6. **HRMS** calculated for $C_{26}H_{36}BFeO_2S$ $[M+H]^+$ 479.1873, found 479.1869.

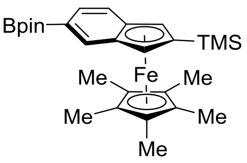

1-Trimethylsilyl-5-(4,4,5,5-tetramethyl-1,3,2-dioxaborolan-2-yl) indenyl-pentamethylcyclopentadienyliron (3l): Purple gum, 87.1 mg, 86% yield. **^{1}H NMR** (700 MHz, $CDCl_3$) δ 7.89 (s, 1H), 7.28 – 7.23 (m, 1H), 7.21 – 7.13 (m, 1H), 4.43 (s, 1H), 4.39 (s, 1H), 1.48 (s, 15H), 1.36 (s, 12H), 0.21 (s, 9H). **^{13}C NMR** (175 MHz, $CDCl_3$) δ 140.0, 127.2, 126.2, 93.4, 90.3, 83.5, 81.9, 77.7, 70.4, 70.1, 25.1, 24.8, 10.4, 0.2. **^{11}B NMR** (128 MHz, $CDCl_3$) δ 31.2. **HRMS** calculated for $C_{28}H_{42}BFeO_2Si$ $[M+H]^+$ 505.2391, found 505.2390.

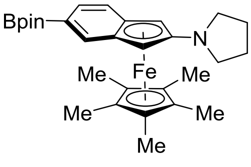

1-(Pyrrolidin-1-yl)-5-(4,4,5,5-tetramethyl-1,3,2-dioxaborolan-2-yl) indenyl-pentamethylcyclopentadienyliron (3m): Purple solid, mp 178.8-180.2 °C, 87.8 mg, 88% yield. **^{1}H NMR** (700 MHz, C_6D_6) δ 8.40 (s, 1H), 7.80 (d, J = 8.5 Hz, 1H), 7.32 (d, J = 8.5 Hz, 1H), 3.84 (s, 1H), 3.80 (s, 1H), 2.86 – 2.79 (m, 4H), 1.64 (s, 15H), 1.56 (s, 4H), 1.22 (s, 6H), 1.21 (s, 6H). **^{13}C NMR** (175 MHz, C_6D_6) δ 141.9, 127.4, 126.4, 117.1, 87.9, 84.6, 83.4, 76.8, 53.1, 52.1, 49.2, 25.8, 25.4, 25.1, 10.7. **^{11}B NMR** (128 MHz, C_6D_6) δ 33.2. **HRMS** calculated for $C_{29}H_{41}BFeNO_2$ $[M+H]^+$ 502.2574, found 502.2578.

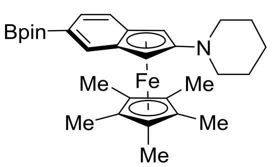

1-(Piperidin-1-yl)-5-(4,4,5,5-tetramethyl-1,3,2-dioxaborolan-2-yl) indenyl-pentamethylcyclopentadienyliron (3n): Purple solid, mp 149.8-150.6 °C, 81.5 mg, 79% yield. **^{1}H NMR** (700 MHz, C_6D_6) δ 8.35 (s, 1H), 7.77 (d, *J* = 8.6 Hz, 1H), 7.27 (d, *J* = 8.6 Hz, 1H), 3.99 (s, 1H), 3.97 (s, 1H), 2.65 – 2.61 (m, 4H), 1.64 (s, 15H), 1.47 – 1.41 (m, 4H), 1.32 – 1.27 (m, 2H), 1.21 (s, 6H), 1.20 (s, 6H). **^{13}C NMR** (175 MHz, C_6D_6) δ 141.1, 126.8, 126.7, 118.9, 87.9, 84.6, 83.3, 77.0, 54.6, 53.9, 51.1, 26.0, 25.1, 24.9, 24.7, 10.1. **^{11}B NMR** (128 MHz, C_6D_6) δ 32.1. **HRMS** calculated for $C_{30}H_{43}BFeNO_2$ $[M+H]^+$ 516.2731, found 516.2726.

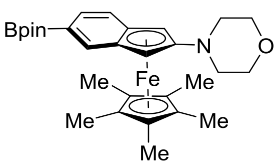

1-Morpholino-5-(4,4,5,5-tetramethyl-1,3,2-dioxaborolan-2-yl) indenyl-pentamethylcyclopentadienyliron (3o): Purple solid, mp 171.7-172.8 °C, 68.7 mg, 66% yield. **^{1}H NMR** (400 MHz, C_6D_6) δ 8.35 (s, 1H), 7.80 (d, *J* = 8.4 Hz, 1H), 7.26 (d, *J* = 8.4 Hz, 1H), 3.90 (s, 2H), 3.63 – 3.52 (m, 4H), 2.57 – 2.46 (m, 4H), 1.61 (s, 15H), 1.21 (s, 12H). **^{13}C NMR** (100 MHz, C_6D_6) δ 141.5, 127.4, 127.2, 118.5, 88.4, 85.1, 83.9, 77.6, 67.3, 54.7, 54.1, 50.9, 25.7, 25.5, 10.7. **^{11}B NMR** (128 MHz, C_6D_6) δ 30.7. **HRMS** calculated for $C_{29}H_{41}BFeNO_3$ $[M+H]^+$ 518.2523, found 518.2525.

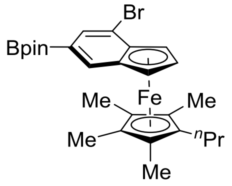

4-Bromo-6-(4,4,5,5-tetramethyl-1,3,2-dioxaborolan-2-yl)indenyl-1,2,3,4-tetramethyl-5-propylcyclopenta-1,3-dienyliron (3p): Purple gum, 94.5 mg, 88% yield. **^{1}H NMR** (400 MHz, $CDCl_3$) δ 7.92 (s, 1H), 7.51 (s, 1H), 4.71 – 4.48 (m, 1H), 4.45 – 4.25 (m, 1H), 4.00 – 3.74 (m, 1H), 2.31 – 1.98 (m, 2H), 1.67 (s, 6H), 1.62 (s, 6H), 1.36 (s, 12H), 1.25 – 1.09 (m, 3H), 0.84 (s, 4H). **^{13}C NMR** (100 MHz, $CDCl_3$) δ 138.8, 127.8, 121.7, 91.2, 88.3, 83.8, 82.9, 79.9, 78.8, 78.5, 78.36, 77.3, 67.6, 67.4, 28.0, 25.1, 24.7, 24.1, 14.3, 10.2, 10.0. **^{11}B NMR** (128 MHz, $CDCl_3$) δ 31.2. **HRMS** calculated for $C_{27}H_{37}BBrFeO_2$ $[M+H]^+$ 539.1414, found 539.1404.

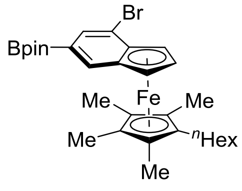

4-Bromo-6-(4,4,5,5-tetramethyl-1,3,2-dioxaborolan-2-yl)indenyl-5-hexyl-1,2,3,4-tetramethylcyclopenta-1,3-dienyliron (3q): Purple gum, 106.1 mg, 91% yield. **^{1}H NMR** (400 MHz, $CDCl_3$) δ 7.92 (s, 1H), 7.51 (s, 1H), 4.55 (d, *J* = 2.4 Hz, 1H), 4.39 (d, *J* = 2.5 Hz, 1H), 3.91 (t, *J* = 2.8 Hz, 1H), 2.24 – 2.08 (m, 2H), 1.67 (s, 3H), 1.63 (s, 3H), 1.62 (s, 3H), 1.61 (s, 3H), 1.36 (s, 6H), 1.35 (s, 6H), 1.27 – 1.21 (m, 6H), 1.17 – 1.10 (m, 2H), 0.92 – 0.80 (m, 3H). **^{13}C NMR** (100 MHz, $CDCl_3$) δ 138.8, 127.8, 121.7, 91.2, 88.3, 83.7, 83.2, 78.9, 78.8, 78.4, 78.3, 77.3, 67.6, 67.4, 31.8, 31.0, 29.6, 25.9, 25.1, 24.7, 22.8, 14.2, 10.1, 10.0, 9.97, 9.9. **^{11}B NMR** (128 MHz, $CDCl_3$) δ 31.4. **HRMS** calculated for $C_{30}H_{43}BBrFeO_2$ $[M+H]^+$ 581.1883, found 581.1886.

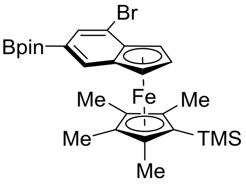

4-Bromo-6-(4,4,5,5-tetramethyl-1,3,2-dioxaborolan-2-yl)indenyl-1,2,3,4-tetramethyl-5-trimethylsilylcyclopenta-1,3-dienyliron (3r): Purple solid, mp 97.2-97.9 °C, 89% yield. **^{1}H NMR** (400 MHz, $CDCl_3$) δ 7.96 (s, 1H), 7.54 (s, 1H), 4.78 – 4.66 (m, 1H), 4.66 – 4.58 (m, 1H), 4.07 – 3.97 (m, 1H), 1.81 (s, 3H), 1.56 (s, 3H), 1.53 (s, 6H), 1.36 (s, 12H), 0.38 (s, 9H). **^{13}C NMR** (100 MHz, $CDCl_3$) δ 138.9, 128.4, 121.6, 91.5, 88.3, 84.33, 84.30, 83.8, 83.3,

83.1, 76.3, 66.8, 66.6, 65.6, 25.1, 24.7, 13.1, 12.2, 9.6, 9.5, 2.3. **^{11}B NMR** (128 MHz, $CDCl_3$) δ 29.8. **HRMS** calculated for $C_{27}H_{39}BBrFeO_2Si$ $[M+H]^+$ 569.1340, found 569.1333.

3.2. Typical procedure for iridium-catalyzed C-H borylation of metallocenes

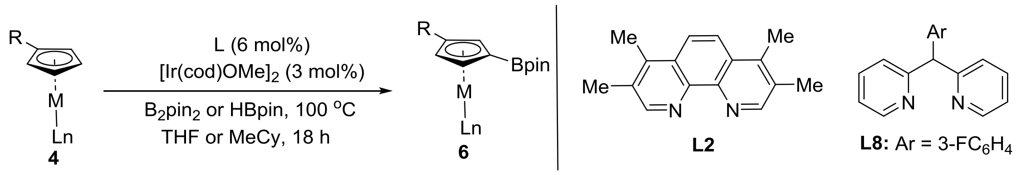

To a 5 mL sealed tube was sequentially added, **4** (0.20 mmol, 1.0 equiv.), $[Ir(cod)OMe]_2$ (4.0 mg, 0.006 mmol), **L2** (2.8 mg, 0.012 mmol) or **L8** (3.2 mg, 0.012 mmol), MeCy or THF (1.0 mL) and HBpin (128.0 mg, 1.0 mmol) or B_2pin_2 (0.10-0.30 mmol). The resulting mixture was stirred at 100 °C for 18 h, the mixture was cooled to rt. After the removal of the solvent, the residue was purified by chromatography (petroleum ether: ethyl acetate =30:1-4:1) to afford product.

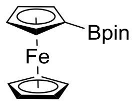

(4,4,5,5-Tetramethyl-1,3,2-dioxaborolan-2-yl)ferrocene (6a): Known compound[14], yellow solid, mp 115.8-116.4 °C, 48.4 mg, 78% yield. **^{1}H NMR** (400 MHz, $CDCl_3$) δ 4.46 – 4.41 (m, 2H), 4.41 – 4.36 (m, 2H), 4.15 (s, 5H), 1.35 (s, 12H). **^{13}C NMR** (100 MHz, $CDCl_3$) δ 83.3, 73.8, 72.1, 68.6, 25.0. **^{11}B NMR** (128 MHz, $CDCl_3$) δ 32.9.

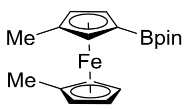

1,1'-Dimethyl-3-(4,4,5,5-tetramethyl-1,3,2-dioxaborolan-2-yl)ferrocene (6b): Yellow solid, mp 45.1-45.8 °C, 50.2 mg, 74% yield. **^{1}H NMR** (400 MHz, $CDCl_3$) δ 4.26 – 4.23 (m, 1H), 4.23 – 4.22 (m, 1H), 4.21 – 4.19 (m, 1H), 4.04 – 4.01 (m, 1H), 3.97 – 3.94 (m, 2H), 3.94 – 3.91 (m, 1H), 2.00 (s, 3H), 1.97 (s, 3H), 1.34 (s, 12H). **^{13}C NMR** (100 MHz, $CDCl_3$) δ 88.4, 84.5, 83.0, 75.5, 74.3, 73.8, 70.5, 70.3, 68.6, 68.1, 25.0, 24.9, 14.4, 14.2. **^{11}B NMR** (128 MHz, $CDCl_3$) δ 33.2. **HRMS** calculated for $C_{18}H_{26}BFeO_2$ $[M+H]^+$ 341.1370, found 341.1365.

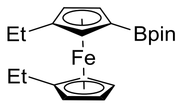

1,1'-Diethyl-3-(4,4,5,5-tetramethyl-1,3,2-dioxaborolan-2-yl)ferrocene (6c): Yellow gum, 49.0 mg, 67% yield. **^{1}H NMR** (400 MHz, $CDCl_3$) δ 4.28 – 4.24 (m, 2H), 4.24 – 4.22 (m, 1H), 4.06 – 4.03 (m, 1H), 4.00 – 3.98 (m, 1H), 3.98 – 3.97 (m, 1H), 3.96 – 3.95 (m, 1H), 2.36 (dq, *J* = 10.6, 7.5 Hz, 4H), 1.34 (s, 12H), 1.20 – 1.12 (m, 6H). **^{13}C NMR** (100 MHz, $CDCl_3$) δ 95.4, 91.6, 83.1, 73.7, 73.6, 72.7, 68.8, 68.6, 68.3, 67.9, 25.1, 22.3, 22.0, 15.1, 15.0. **^{11}B NMR** (128 MHz, $CDCl_3$) δ 33.5. **HRMS** calculated for $C_{20}H_{30}BFeO_2$ $[M+H]^+$ 369.1683, found 369.1683.

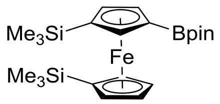

1,1'-Bis(trimethylsilane)-3-(4,4,5,5-tetramethyl-1,3,2-dioxaborolan-2-yl)ferrocene (6d): Yellow solid, mp 88.7-89.4 °C, 64.6 mg, 71% yield. **^{1}H NMR** (700 MHz, $CDCl_3$) δ 4.57 – 4.54 (m, 1H), 4.41 – 4.38 (m, 1H), 4.34 – 4.32 (m, 1H), 4.32 – 4.30 (m, 1H), 4.30 – 4.27 (m, 1H), 4.07 – 4.04 (m, 1H), 4.03 – 4.00 (m, 1H), 1.35 (s, 12H), 0.25 (s, 9H), 0.23 (s, 9H). **^{13}C NMR** (175 MHz, $CDCl_3$) δ 83.3, 78.9, 77.0, 76.9, 73.4, 73.0, 72.2, 71.2, 25.1, 0.0, -0.1. **^{11}B NMR** (128 MHz, $CDCl_3$) δ 32.5. **HRMS** calculated for $C_{22}H_{38}BFeO_2Si_2$ $[M+H]^+$ 457.1847, found 457.1852.

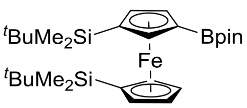

1,1'-Bis(*tert*-butyldimethylsilane)-3-(4,4,5,5-tetramethyl-1,3,2-dioxaborolan-2-yl)ferrocene (6e): Orange red solid, mp 125.1-125.9 °C, 81.9 mg, 76% yield. **^{1}H NMR** (400 MHz, $CDCl_3$) δ 4.50 (dd, *J* = 2.1, 0.9 Hz, 1H), 4.35 – 4.33 (m, 1H), 4.32 – 4.30 (m, 1H), 4.28 – 4.25 (m, 1H), 4.22 (dd, *J* = 2.1, 1.1 Hz, 1H), 4.01 – 3.97 (m, 1H), 3.95 – 3.92 (m, 1H), 1.35 (s, 12H), 0.79 (s, 9H), 0.78 (s, 9H), 0.32 (s, 3H), 0.265 (s, 3H), 0.258 (s, 3H), 0.25 (s, 3H). **^{13}C NMR** (100 MHz, $CDCl_3$) δ 83.2, 79.5, 77.5, 77.1, 74.02, 73.96, 73.5, 72.4, 70.9, 70.4, 26.4, 25.1, 25.0, 24.9, 17.11, 17.07, -5.48, -5.49, -5.6, -5.7. **^{11}B NMR** (128 MHz, $CDCl_3$) δ 33.8. **HRMS** calculated for $C_{28}H_{50}BFeO_2Si_2$ $[M+H]^+$ 541.2786, found 541.2785.

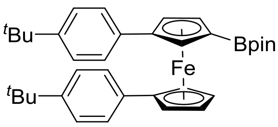

1,1'-Bis((4''-*tert*-butyl)phenyl)-3-(4,4,5,5-tetramethyl-1,3,2-dioxaborolan-2-yl)ferrocene (6f): Yellow gum, 86.5 mg, 75% yield. **^{1}H NMR** (700 MHz, $CDCl_3$) δ 7.23 (s, 4H), 7.21 (s, 4H), 4.72 – 4.70 (m, 1H), 4.63 – 4.60 (m, 1H), 4.44 – 4.41 (m, 1H), 4.38 – 4.36 (m, 1H), 4.35 – 4.33 (m, 1H), 4.31 – 4.28 (m, 1H), 4.14 – 4.11 (m, 1H), 1.36 (s, 12H), 1.34 (s, 9H), 1.33 (s, 9H). ^{13}C NMR (175 MHz, $CDCl_3$) δ 149.0, 148.98, 134.9, 134.8, 126.2, 126.0, 125.19, 125.16, 90.6, 87.4, 83.4, 76.5, 73.0, 72.8, 71.1, 70.2, 70.1, 69.2, 68.3, 34.6, 31.5, 25.12, 25.08. **^{11}B NMR** (128 MHz, $CDCl_3$) δ 33.6. **HRMS** calculated for $C_{36}H_{46}BFeO_2$ $[M+H]^+$ 577.2935, found 577.2939.

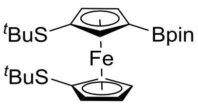

1,1'-Bis(*tert*-butylthio)-3-(4,4,5,5-tetramethyl-1,3,2-dioxaborolan-2-yl)ferrocene (6g): Yellow solid, mp 107.8-108.7 °C, 58.1 mg, 75% yield. **^{1}H NMR** (400 MHz, $CDCl_3$) δ 4.54 – 4.51 (m, 1H), 4.49 – 4.47 (m, 1H), 4.46 (dd, *J* = 2.3, 1.1 Hz, 1H), 4.27 – 4.21 (m, 4H), 1.35 (s, 12H), 1.18 (s, 9H), 1.15 (s, 9H). **^{13}C NMR** (100 MHz, $CDCl_3$) δ 83.6, 82.9, 81.6, 80.8, 78.3, 78.0, 77.8, 76.9, 71.9, 71.2, 45.3, 45.0, 30.82, 30.78, 25.2, 25.0. **^{11}B NMR** (128 MHz, $CDCl_3$) δ 34.3. **HRMS** calculated for $C_{24}H_{38}BFeO_2S_2$ $[M+H]^+$ 489.1750, found 489.1755.

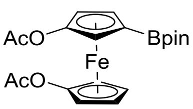

1,1'-Diacetoxy-3-(4,4,5,5-tetramethyl-1,3,2-dioxaborolan-2-yl) ferrocene (6h): Yellow solid, mp 83.4-84.5 °C, 48.6 mg, 49% yield. **^{1}H NMR** (400 MHz, $CDCl_3$) δ 4.73 – 4.64 (m, 2H), 4.51 – 4.45 (m, 1H), 4.45 – 4.40 (m, 1H), 4.30 – 4.24 (m, 1H), 4.05 – 3.99 (m, 1H), 3.99 – 3.93 (m, 1H), 2.16 (s, 6H), 1.31 (s, 12H). **^{13}C NMR** (100 MHz, $CDCl_3$) δ 169.0, 168.9, 118.6, 116.2, 83.4, 70.3, 66.9, 66.4, 65.1, 64.7, 62.7, 62.4, 24.9, 24.8, 21.3. **^{11}B NMR** (128 MHz, $CDCl_3$) δ 32.4 **HRMS** calculated for $C_{20}H_{26}BFeO_6$ $[M+H]^+$ 429.1166, found 429.1168.

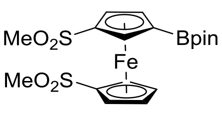

1,1'-Bis(methylsulfonyl)-3-(4,4,5,5-tetramethyl-1,3,2-dioxaborolan-2-yl) ferrocene (6i): Yellow solid, mp 148.8-149.6 °C, 30.3 mg, 64% yield. **^{1}H NMR** (400 MHz, $CDCl_3$) δ 5.12 – 5.08 (m, 1H), 5.02 (s, 1H), 5.01 – 4.98 (m, 1H), 4.95 – 4.93 (m, 1H), 4.87 – 4.83 (m, 1H), 4.74 – 4.71 (m, 1H), 4.66 – 4.63 (m, 1H), 3.03 (s, 3H), 3.00 (s, 3H), 1.35 (s, 12H). **^{13}C NMR** (100 MHz, $CDCl_3$) δ 94.0, 92.1, 84.4, 79.1, 76.7, 74.47, 74.45, 73.3, 71.9, 71.5, 44.9, 25.03, 24.99. **^{11}B NMR** (128 MHz, $CDCl_3$) δ 31.6. **HRMS** calculated for $C_{18}H_{29}BFeNO_6S_2$ $[M+NH_4]^+$ 486.0873, found 486.0876.

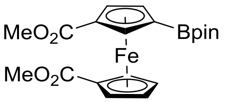

1,1’-Bis(methoxycarbonyl)-3-(4,4,5,5-tetramethyl-1,3,2-dioxaborolan-2-yl) ferrocene (6j): Known compound[14], orange red solid, mp 145.7-146.9 °C, 71.2 mg, 83% yield. **^{1}H NMR** (400 MHz, $CDCl_3$) δ 5.08 (t, *J* = 1.2 Hz, 1H), 4.97 (dd, *J* = 2.6, 1.2 Hz, 1H), 4.79 (dt, *J* = 2.5, 1.2 Hz, 1H), 4.77 (dt, *J* = 2.5, 1.2 Hz, 1H), 4.59 (dd, *J* = 2.5, 1.3 Hz, 1H), 4.41 (td, *J* = 2.5, 1.4 Hz, 1H), 4.33 (td, *J* = 2.5, 1.4 Hz, 1H), 3.81 (s, 6H), 1.35 (s, 12H). **^{13}C NMR** (100 MHz, $CDCl_3$) δ 170.4, 170.3, 83.9, 78.0, 77.7, 76.7, 75.2, 73.5, 73.2, 72.6, 72.3, 72.1, 51.9, 25.1, 25.0. **^{11}B NMR** (128 MHz, $CDCl_3$) δ 32.6.

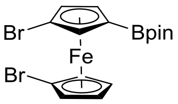

1,1’-Di(bromo)-3-(4,4,5,5-tetramethyl-1,3,2-dioxaborolan-2-yl)ferrocene (6k): Known compound[14], yellow solid, mp 60.1-60.6 °C, 54.6 mg, 54% yield. **^{1}H NMR** (400 MHz, $CDCl_3$) δ 4.63 (t, *J* = 1.2 Hz, 1H), 4.59 (dd, *J* = 2.4, 1.2 Hz, 1H), 4.39 (dd, *J* = 2.4, 1.3 Hz, 1H), 4.37 (dt, *J* = 2.5, 1.2 Hz, 1H), 4.34 (dt, *J* = 2.4, 1.2 Hz, 1H), 4.19 (td, *J* = 2.6, 1.4 Hz, 1H), 4.13 (td, *J* = 2.6, 1.4 Hz, 1H), 1.34 (s, 12H). **^{13}C NMR** (100 MHz, $CDCl_3$) δ 83.8, 80.8, 78.8, 77.9, 75.6, 73.79, 73.0, 70.5, 69.4, 25.1, 25.0. **^{11}B NMR** (128 MHz, $CDCl_3$) δ 32.2.

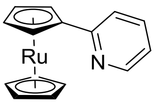

(Pyridin-2-yl)ruthenocene (6m): Known compound[15], light yellow solid, mp 129.6-130.1 °C, 33.3 mg, 54% yield. **^{1}H NMR** (400 MHz, $CDCl_3$) δ 8.43 (d, *J* = 4.4 Hz, 1H), 7.55 – 7.48 (m, 1H), 7.33 (d, *J* = 8.0 Hz, 1H), 7.06 – 6.99 (m, 1H), 5.32 – 5.27 (m, 2H), 4.76 – 4.71 (m, 2H), 4.46 (s, 5H).

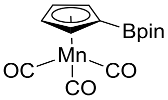

(4,4,5,5-Tetramethyl-1,3,2-dioxaborolan-2-yl)cymantrene (6n): Known compound[14], white solid, 36.9 mg, 56% yield. **^{1}H NMR** (400 MHz, $CDCl_3$) δ 5.15 – 5.10 (m, 2H), 4.87 – 4.81 (m, 2H), 1.30 (s, 12H). **^{13}C NMR** (100 MHz, $CDCl_3$) δ 91.7, 85.2, 84.2, 24.7. **^{11}B NMR** (128 MHz, $CDCl_3$) δ 30.2.

4. Derivatizations and scale-up synthesis

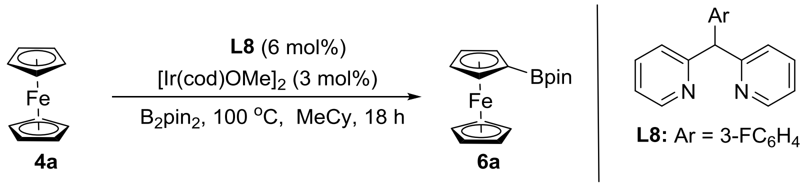

To a 100 mL sealed tube was sequentially added, **4a** (930.1 mg, 5.0 mmol), $[Ir(cod)OMe]_2$ (99.4 mg, 0.15 mmol), **L8** (79.3 mg, 0.30 mmol), B_2pin_2 (888.8 mg, 3.5 mmol) and MeCy (25 mL). The resulting mixture was stirred at 100 °C for 18 h. the mixture was cooled to rt. After the removal of the solvent, the residue was purified by flash column chromatography (silica gel, petroleum ether:ethyl acetate = 30:1) to afford product as a yellow solid, 1.02 g, 65%.

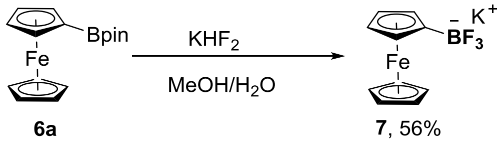

Ferrocenly trifluoroboric acid potassium salt (7): To a solution of **6a** (80.8 mg, 0.20 mmol) in methanol (1.0 mL) was added aqueous potassium hydrogen fluoride (0.1 mL, 10 M, 1.0 mmol). The resulting mixture was stirred at room temperature for 5 h, concentrated in vacuo. The crude product was washed with water (5 mL ×3), the residue was dried in vacuo to give a yellow solid, known compound[16], 32.9 mg, 56% yield. ^{1}**H NMR** (400 MHz, DMSO) δ 3.98 (s, 5H), 3.90 (s, 4H). ^{19}**F NMR** (376 MHz, DMSO) δ -133.4.

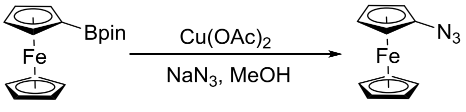

6a **8**, 74%

1-Azidoferrocene (8): A mixture of **6a** (62.4 mg, 0.20 mmol), NaN_3 (26.0 mg, 0.40 mmol), $Cu(OAc)_2$ (7.3 mg, 0.04 mmol) and MeOH (1.0 mL) was stirred at 55 ℃ under air atmosphere for 6 hours. After cooling down to room temperature, the residue was purified by flash column chromatography (silica gel, petroleum ether) to afford product as a yellow oil, known compound[17], 33.7 mg, 74% yield. ^{1}**H NMR** (400 MHz, $CDCl_3$) δ 4.28 (s, 5H), 4.27 – 4.25 (m, 2H), 4.06 – 4.02 (m, 2H).

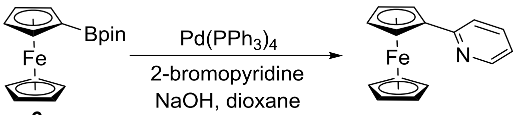

6a **9**, 86%

(Pyridin-2-yl)ferrocene (9): A mixture of **6a** (62.4 mg, 0.20 mmol), $Pd(PPh_3)_4$ (11.5 mg, 0.01 mmol), dioxane (1.0 mL), 2-bromopyridine (47.4 mg, 0.30 mmol) and 3 M NaOH (0.5 mL). The mixture was stirred at 110 ℃ for 18 h. The mixture was cooled to rt and diluted with ethyl acetate. The layers were separated and the aqueous layer was extracted with ethyl acetate. The combined organic layers were washed with saturated NaCl, dried with Na_2SO_4 and concentrated under vacuum. The residue was purified by flash column chromatography (silica gel, petroleum ether: ethyl acetate = 20:1) to afford product as an orange red solid, known compound[18], mp 89.5-91.0 °C, 45.2 mg, 86% yield. ^{1}**H NMR** (700 MHz, $CDCl_3$) δ 8.50 (ddd, J = 4.8, 1.7, 0.8 Hz, 1H), 7.59 – 7.54 (m, 1H), 7.41 (dt, J = 8.0, 0.9 Hz, 1H), 7.06 (ddd, J = 7.4, 4.9, 1.1 Hz, 1H), 4.94 – 4.90 (m, 2H), 4.41 – 4.38 (m, 2H), 4.05 (s, 5H).

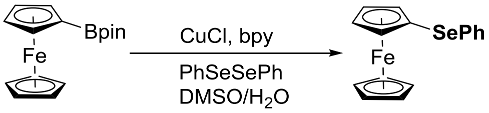

6a **10**, 57%

(Phenylseleno)ferrocene (10): A mixture of **6a** (62.4 mg, 0.20 mmol), diphenyl diselenide (124.8 mg, 0.40 mmol), CuCl (4.0 mg, 0.04 mmol), 2,2-bipyridine (6.2 mg, 0.04 mol) and DMSO (0.6 mL)/H_2O (0.3 mL) was stirred at 80 ℃ under air atmosphere for 16 h. The mixture was cooled to rt and diluted with ethyl acetate. The layers were separated and the aqueous layer was extracted with ethyl acetate. The combined organic layers were washed with saturated NaCl, dried with Na_2SO_4 and concentrated under vacuum. The residue was purified by flash column chromatography (silica gel, petroleum ether) to afford product as a yellow solid, known

compound[19], mp 118.9-119.7 °C, 45.2 mg, 38.6 mg, 57% yield. ^{1}**H NMR** (700 MHz, CDCl$_3$) δ 7.24 – 7.21 (m, 2H), 7.16 (t, *J* = 7.5 Hz, 2H), 7.13 – 7.09 (m, 1H), 4.51 – 4.43 (m, 2H), 4.39 – 4.33 (m, 2H), 4.28 (s, 5H).

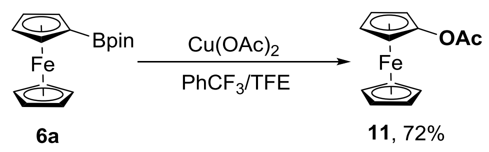

Ferrocenyl acetate (11): A mixture of **6a** (62.4mg, 0.20 mmol), Cu(OAc)$_2$ (72.7 mg, 0.40 mmol), TFE (0.50 mL) and PhCF$_3$ (0.50 mL) was stirred at 60 ℃ under air atmosphere for 12 h. After the mixture was cooled down to room temperature and concentrated under reduced pressure. The residue was purified by flash column chromatography (silica gel, petroleum ether: ethyl acetate = 30:1) to afford product as a yellow solid, known compound[20], mp 64.7-65.3 °C, 35.2 mg, 72% yield. ^{1}**H NMR** (400 MHz, CDCl$_3$) δ 4.41 (t, *J* = 1.8 Hz, 2H), 4.23 (s, 5H), 3.96 (t, *J* = 1.8 Hz, 2H), 2.17 (s, 3H). ^{13}**C NMR** (100 MHz, CDCl$_3$) δ 169.5, 115.8, 69.5, 63.4, 61.0, 21.4.

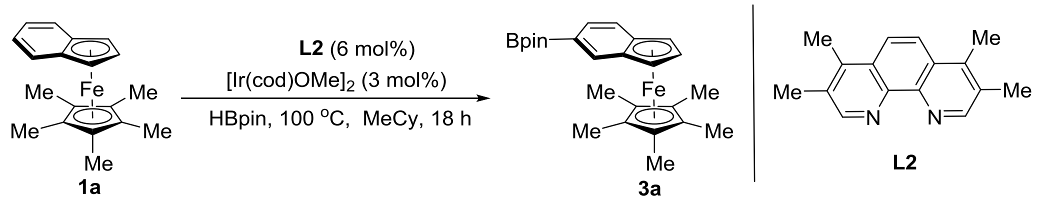

To a 100 mL sealed tube was sequentially added, **1a** (918.7 mg, 3.0 mmol), [Ir(cod)OMe]$_2$ (59.6 mg, 0.09 mmol), **L2** (42.5 mg, 0.18 mmol), MeCy (15 mL) and HBpin (1.15 g, 9.0 mmol). The resulting mixture was stirred at 100 °C for 18 h. The mixture was cooled to rt. After the removal of the solvent, the residue was purified by flash column chromatography (silica gel, petroleum ether:ethyl acetate = 30:1) to afford product as a purple solid, 1.10 g, 85%.

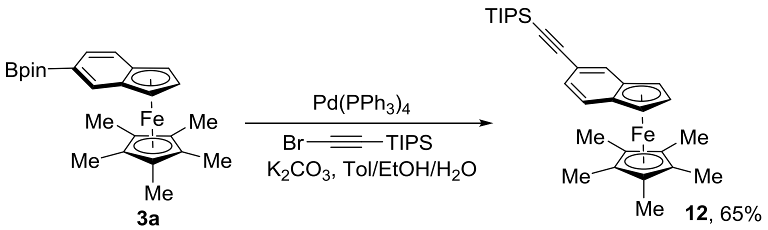

5-((Triisopropylsilyl)ethynyl)indenyl-pentamethylcyclopentadienyliron (12): A mixture of **3a** (86.4 mg, 0.20 mmol), Pd(PPh$_3$)$_4$ (11.5 mg, 0.01 mmol), K$_2$CO$_3$ (69.1 mg, 0.50 mmol), toluene/EtOH/H$_2$O (0.3 mL/0.3 mL/0.03 mL) and (bromoethynyl)triisopropylsilane (104.5 mg, 0.40 mmol). The mixture was stirred at 100 ℃ for 16 h. The mixture was cooled to rt and diluted with ethyl acetate. The layers were separated and the aqueous layer was extracted with ethyl acetate. The combined organic layers were washed with saturated NaCl, dried with Na$_2$SO$_4$ and concentrated under vacuum. The residue was purified by flash column chromatography (silica gel, petroleum ether: ethyl acetate = 50:1) to afford the product **12** as a purple gum, 63.0 mg, 65% yield. ^{1}**H NMR** (400 MHz, CDCl$_3$) δ 7.56 (s, 1H), 7.26 – 7.23 (m, 1H), 7.00 (d, *J* = 8.6 Hz, 1H), 4.42 – 4.34 (m, 1H), 4.34 – 4.29 (m, 1H), 3.92 – 3.84 (m, 1H), 1.65 (s, 15H), 1.15 (s, 21H). ^{13}**C**

NMR (100 MHz, $CDCl_3$) δ 133.5, 127.2, 125.4, 117.0, 109.4, 88.3, 87.5, 87.3, 78.5, 77.2, 66.6, 66.1, 18.9, 11.5, 10.2. **HRMS** calculated for $C_{30}H_{43}FeSi$ $[M+H]^+$ 487.2478, found 487.2478.

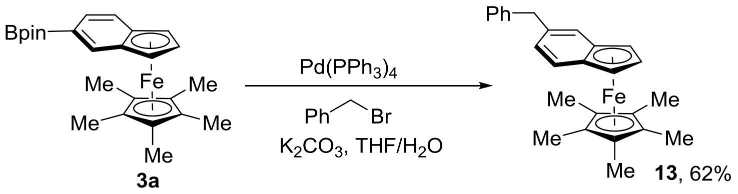

5-Benzylindenyl-pentamethylcyclopentadienyliron (13): A mixture of **3a** (86.4 mg, 0.20 mmol), $Pd(PPh_3)_4$ (23.1 mg, 0.02 mmol), K_2CO_3 (110.6 mg, 0.80 mmol), THF/H_2O (1.0 mL/0.1 mL) and benzylbromide (51.3 mg, 0.30 mmol). The mixture was stirred at 100 ℃ for 24 h. The mixture was cooled to rt and diluted with ethyl acetate. The layers were separated and the aqueous layer was extracted with ethyl acetate. The combined organic layers were washed with saturated NaCl, dried with Na_2SO_4 and concentrated under vacuum. The residue was purified by flash column chromatography (silica gel, petroleum ether: ethyl acetate = 50:1) to afford product as a red gum. 49.1 mg, 62% yield. **^{1}H NMR** (400 MHz, C_6D_6) δ 7.14 – 6.98 (m, 6H), 6.93 (s, 1H), 6.84 – 6.71 (m, 1H), 4.21 – 4.04 (m, 2H), 3.84 – 3.69 (m, 2H), 3.66 – 3.54 (m, 1H), 1.52 (s, 15H). **^{13}C NMR** (100 MHz, C_6D_6) δ 141.7, 136.0, 129.6, 128.7, 127.8, 126.3, 125.3, 125.2, 89.5, 87.5, 78.0, 75.9, 65.9, 65.7, 43.0, 10.4. **HRMS** calculated for $C_{26}H_{29}Fe$ $[M+H]^+$ 397.1613, found 397.1613.

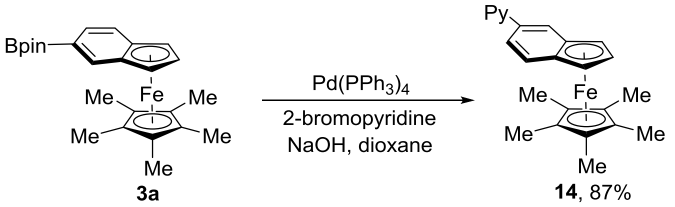

5-(Pyridin-2-yl)indenyl-pentamethylcyclopentadienyliron (14): A mixture of **3a** (86.4 mg, 0.20 mmol), $Pd(PPh_3)_4$ (11.5 mg, 0.01 mmol), dioxane (1.0 mL), 2-bromopyridine (47.4 mg, 0.30 mmol) and 3 M NaOH (0.5 mL, 1.5 mmol). The mixture was stirred at 110 ℃ for 18 h. The mixture was cooled to rt and diluted with ethyl acetate. The layers were separated and the aqueous layer was extracted with ethyl acetate. The combined organic layers were washed with saturated NaCl, dried with Na_2SO_4 and concentrated under vacuum. The residue was purified by flash column chromatography (silica gel, petroleum ether: ethyl acetate = 20:1) to afford the reddish brown product, 66.4 mg, 87% yield. **^{1}H NMR** (400 MHz, C_6D_6) δ 8.72 – 8.54 (m, 1H), 8.32 – 8.15 (m, 1H), 8.07 – 7.92 (m, 1H), 7.57 – 7.42 (m, 1H), 7.40 – 7.29 (m, 1H), 7.24 – 7.08 (m, 1H), 6.74 – 6.59 (m, 1H), 4.40 – 4.30 (m, 1H), 4.29 – 4.18 (m, 1H), 3.83 – 3.69 (m, 1H), 1.57 (s, 15H). **^{13}C NMR** (100 MHz, C_6D_6) δ 158.5, 150.0, 136.3, 134.5, 127.7, 127.6, 122.5, 121.2, 119.4, 89.0, 88.7, 78.3, 77.2, 66.9, 66.4, 10.4. **HRMS** calculated for $C_{24}H_{26}FeN$ $[M+H]^+$ 384.1409, found 384.1407.

5. X-ray Crystal Structures

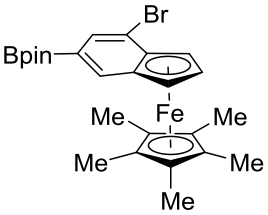

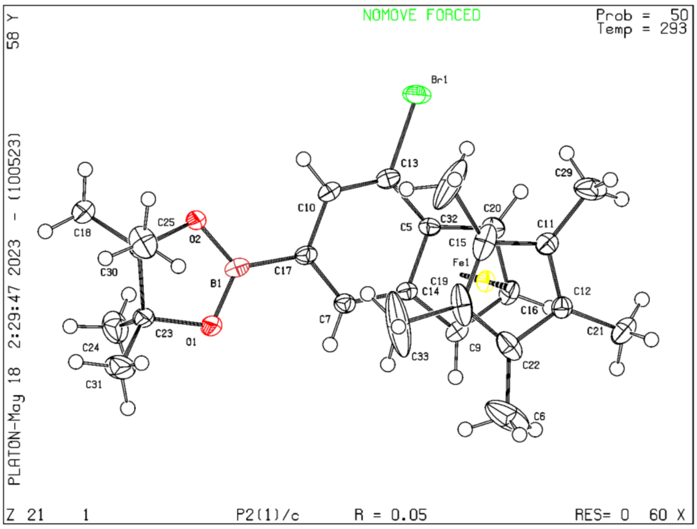

**Table 1 Crystal data and structure refinement for 3b**	
Identification code	**3b**
Empirical formula	$C_{25}H_{32}BO_2FeBr$
Formula weight	511.08
Temperature/K	293(2)
Crystal system	monoclinic
Space group	$P2_1/c$
a/Å	7.0433(4)
b/Å	24.3395(12)
c/Å	13.9919(9)
α/°	90.00
β/°	104.077(6)
γ/°	90.00
Volume/Å^3	2326.6(2)
Z	4
ρ_{calc}g/cm^3	1.459
μ/mm^{-1}	7.344
F(000)	1056.0
Crystal size/mm^3	0.19 × 0.16 × 0.12
Radiation	CuKα (λ = 1.54178)
2Θ range for data collection/°	7.26 to 134.4
Index ranges	$-6 \le h \le 8$, $-27 \le k \le 28$, $-16 \le l \le 14$
Reflections collected	9242
Independent reflections	3883 [R_{int} = 0.0433, R_{sigma} = 0.0414]
Data/restraints/parameters	3883/0/280
Goodness-of-fit on F^2	1.052
Final R indexes [I>=2σ (I)]	R_1 = 0.0501, wR_2 = 0.1276
Final R indexes [all data]	R_1 = 0.0608, wR_2 = 0.1336
Largest diff. peak/hole / e Å^{-3}	1.06/-0.69

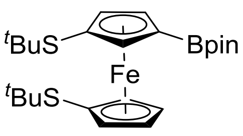

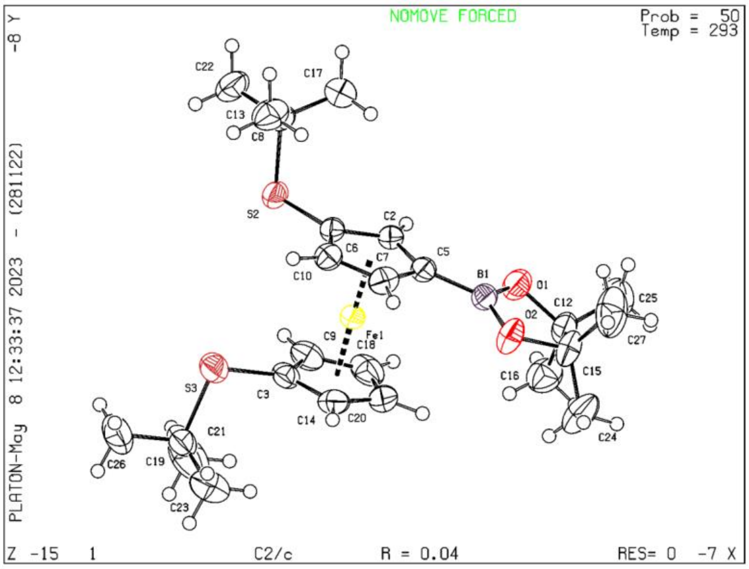

Table 2 Crystal data and structure refinement for 6h.	
Identification code	**6h**
Empirical formula	$C_{24}H_{37}BO_2S_2Fe$
Formula weight	488.32
Temperature/K	293(2)
Crystal system	monoclinic
Space group	C2/c
a/Å	21.341(2)
b/Å	10.8890(8)
c/Å	24.634(3)
α/°	90.00
β/°	115.323(12)
γ/°	90.00
Volume/Å^3	5174.4(9)
Z	8
ρ_{calc}g/cm^3	1.254
μ/mm^{-1}	0.761
F(000)	2080.0
Crystal size/mm^3	0.17 × 0.15 × 0.11
Radiation	MoKα (λ = 0.71073)
2Θ range for data collection/°	6.2 to 50.04
Index ranges	-25 ≤ h ≤ 24, -12 ≤ k ≤ 12, -29 ≤ l ≤ 29
Reflections collected	28191
Independent reflections	4559 [R_{int} = 0.0488, R_{sigma} = 0.0259]
Data/restraints/parameters	4559/0/281
Goodness-of-fit on F^2	1.081
Final R indexes [I>=2σ (I)]	R_1 = 0.0375, wR_2 = 0.0784
Final R indexes [all data]	R_1 = 0.0519, wR_2 = 0.0857
Largest diff. peak/hole / e Å^{-3}	0.38/-0.33

6. Copies of NMR spectra

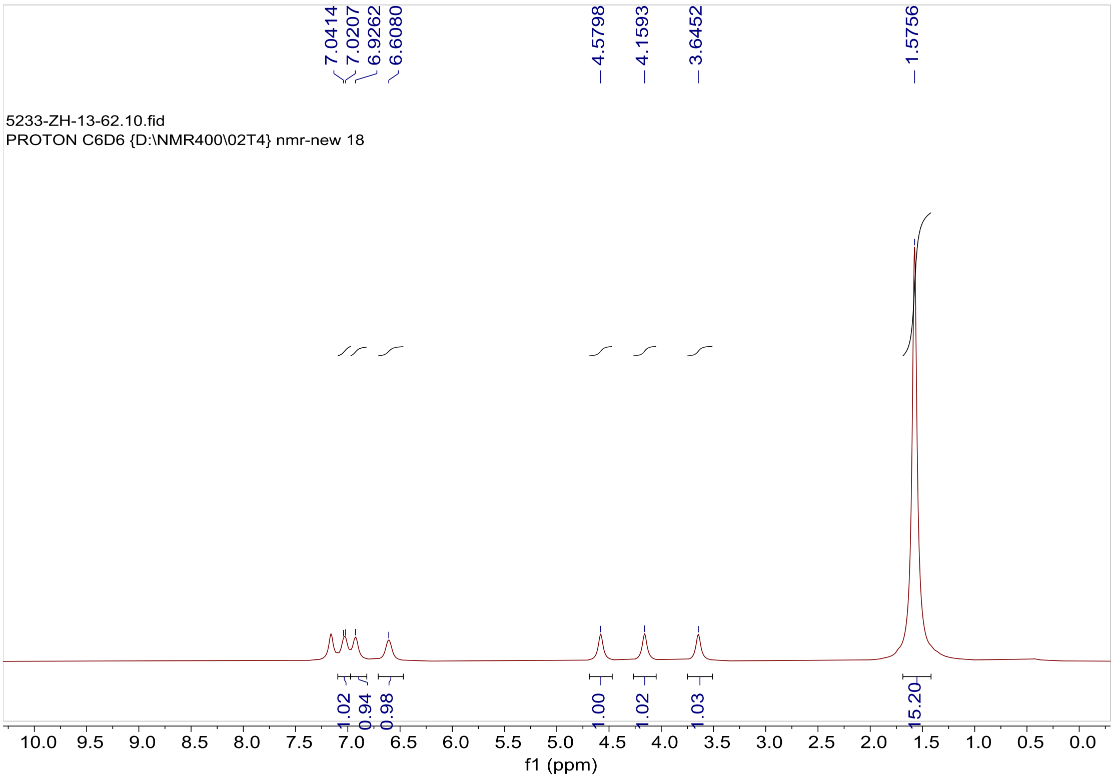

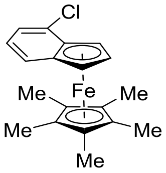

1c

^{1}H NMR (400 MHz, C_6D_6)
^{13}C NMR (100 MHz, C_6D_6)

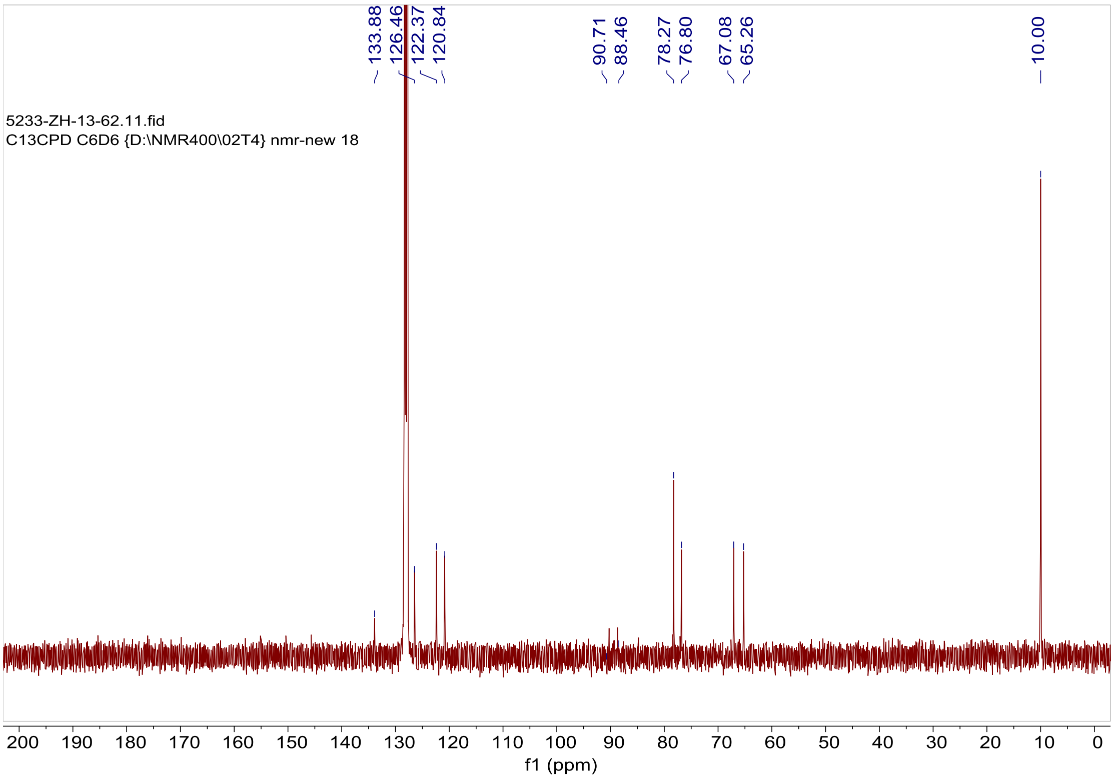

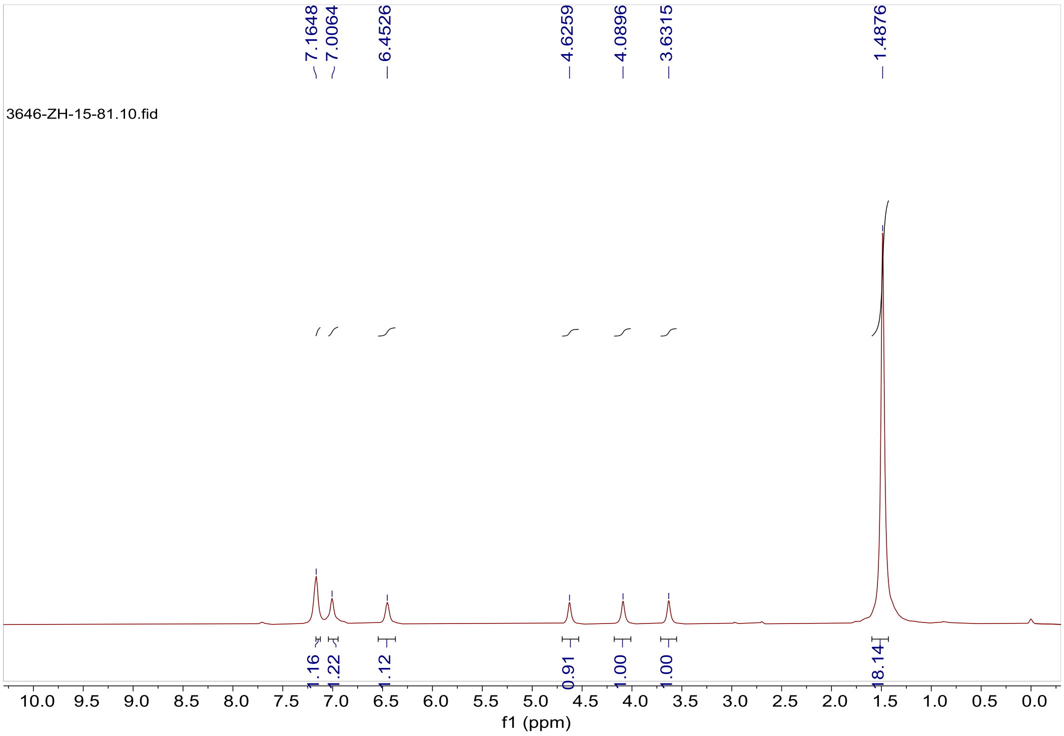

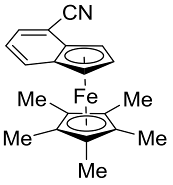

1d

^{1}H NMR (400 MHz, C_6D_6)
^{13}C NMR (100 MHz, C_6D_6)

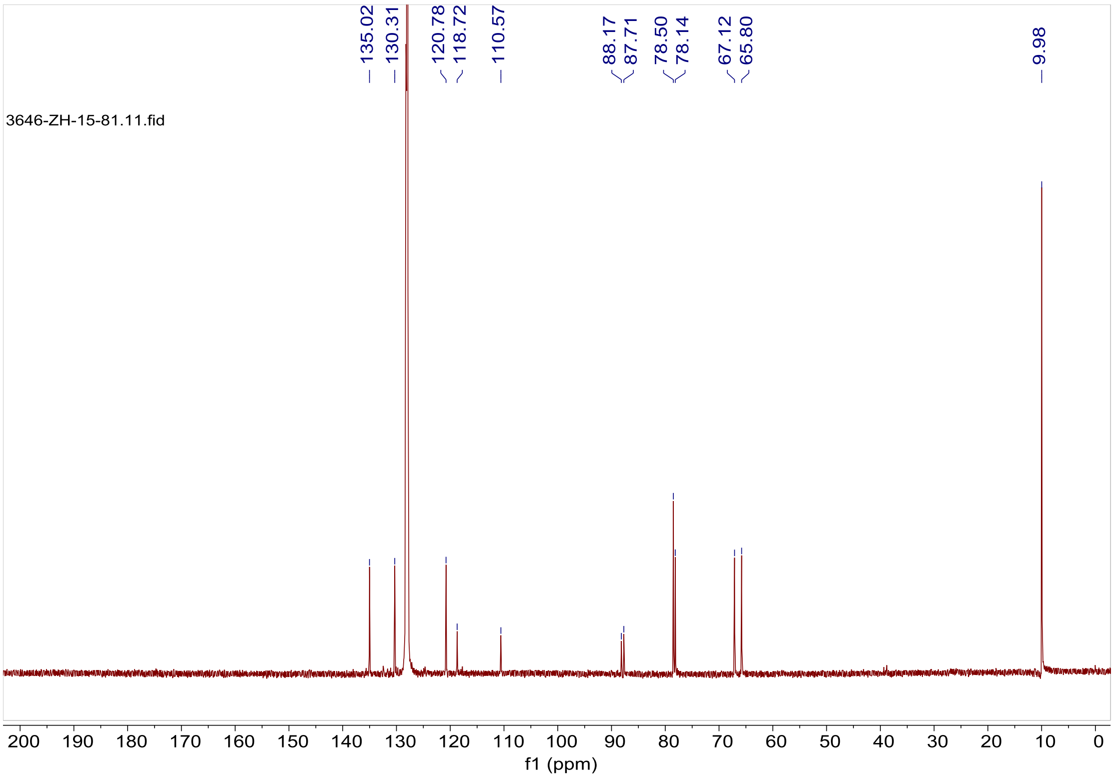

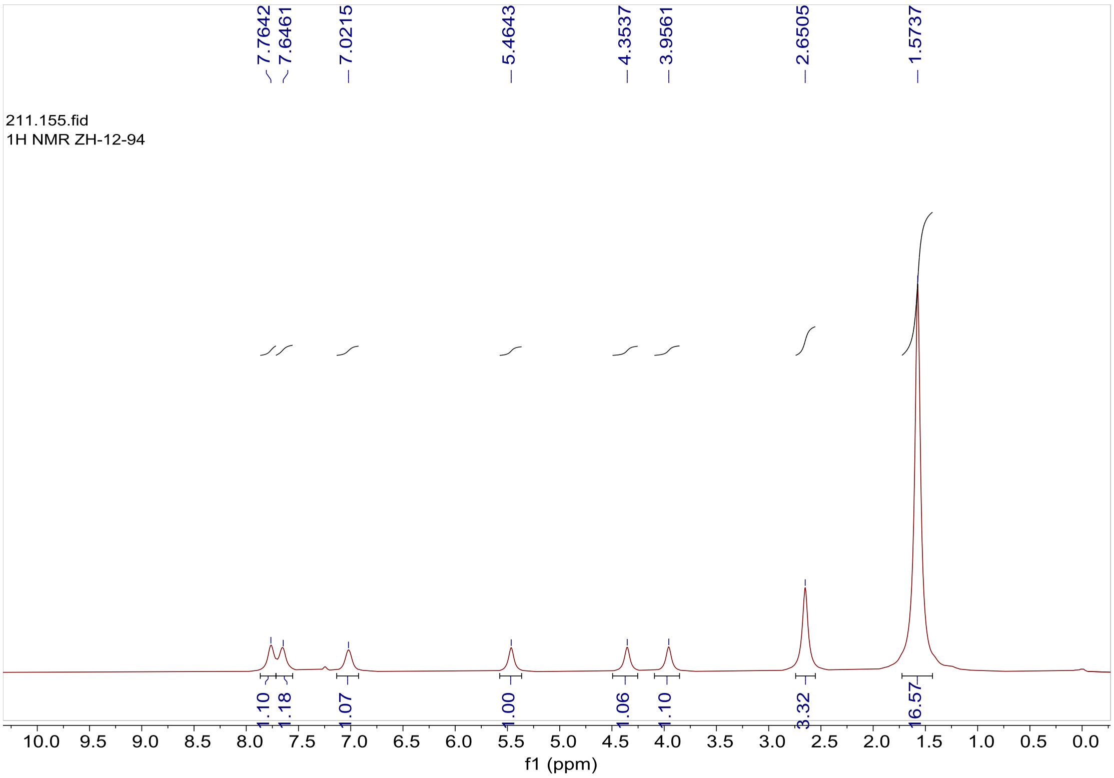

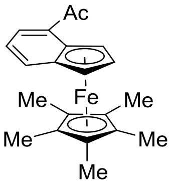

1e

^{1}H NMR (400 MHz, $CDCl_3$)

^{13}C NMR (100 MHz, $CDCl_3$)

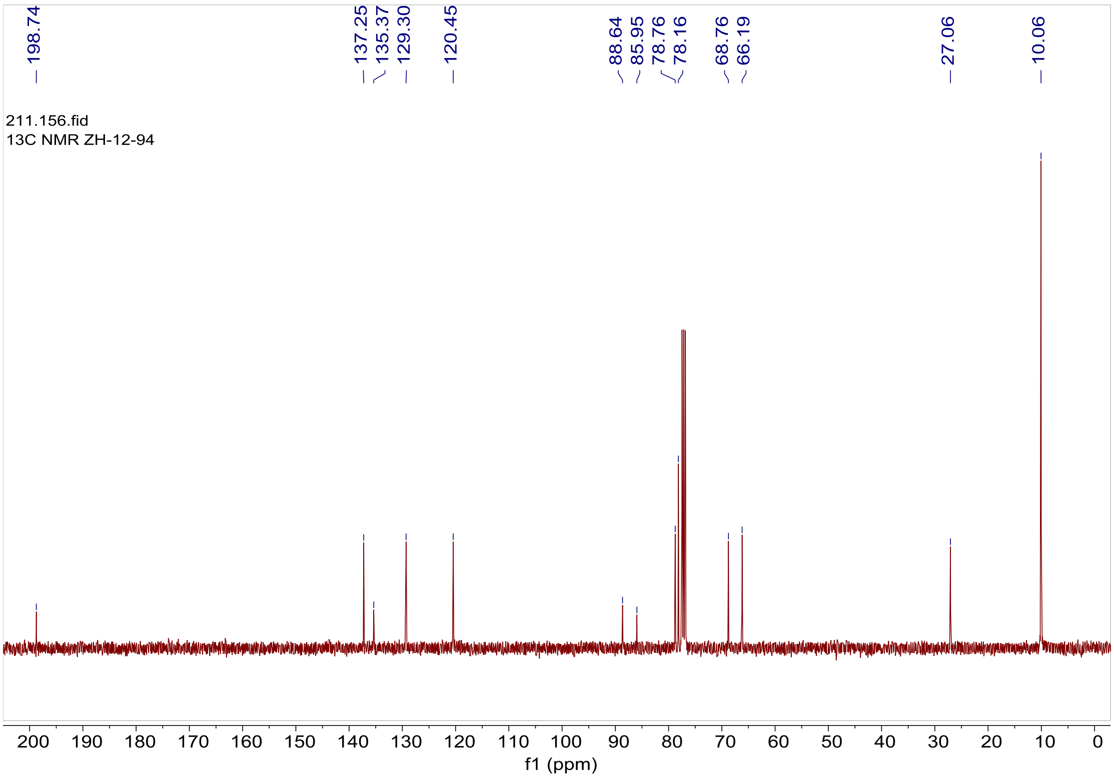

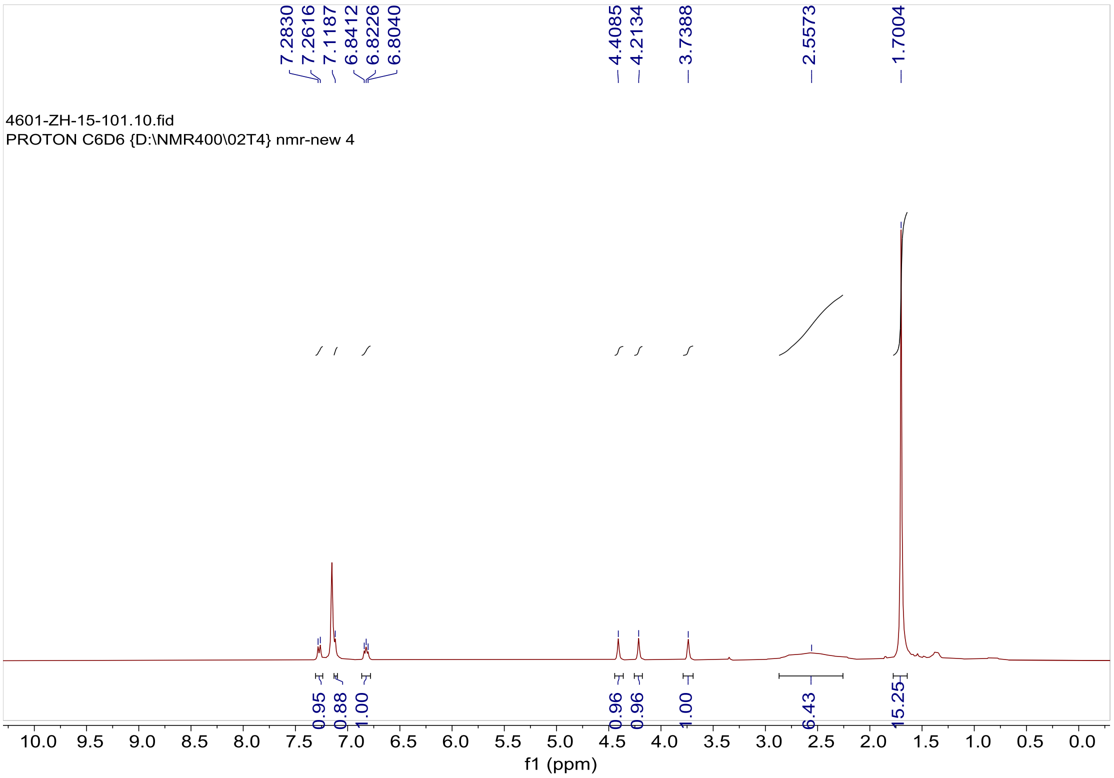

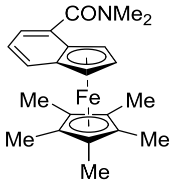

1f

^{1}H NMR (400 MHz, C_6D_6)
^{13}C NMR (100 MHz, C_6D_6)

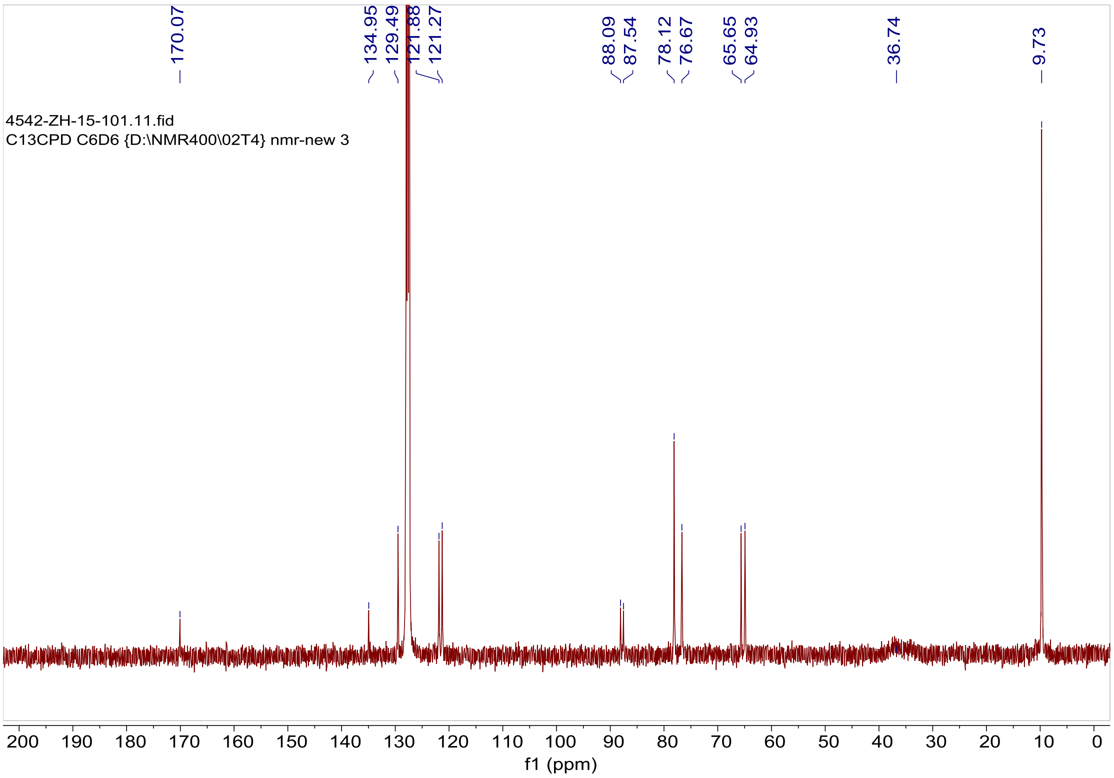

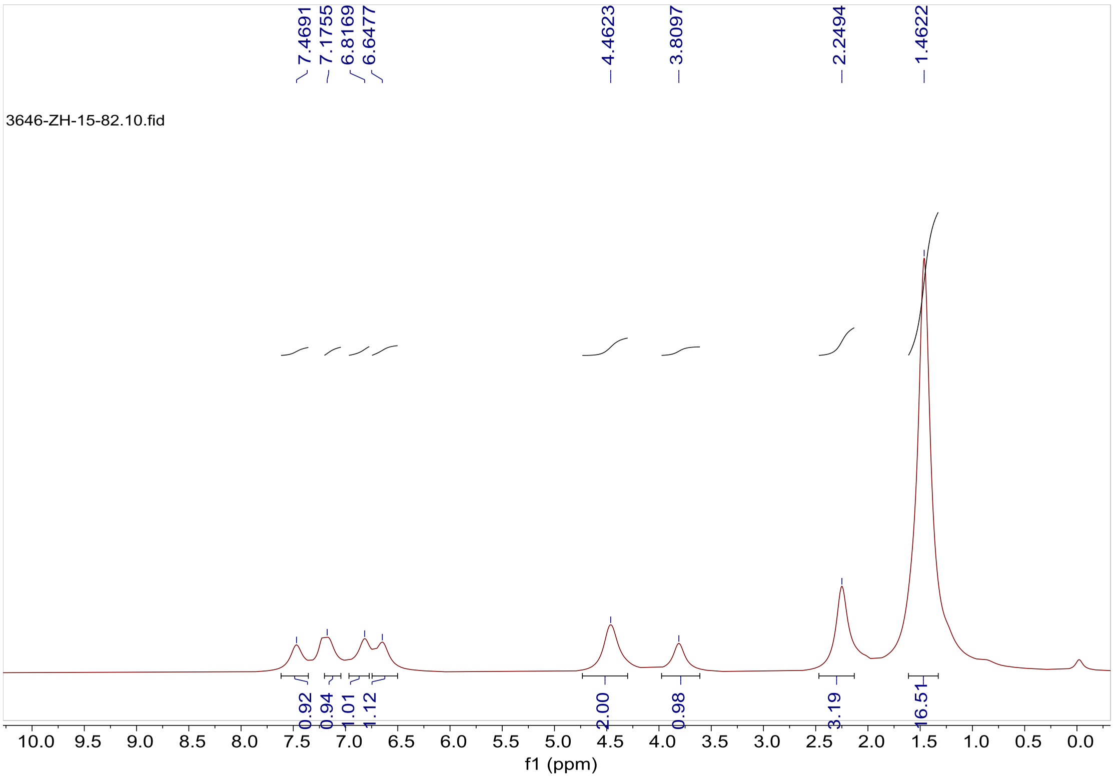

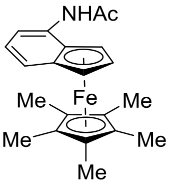

1g

^{1}H NMR (400 MHz, $CDCl_3$)
^{13}C NMR (100 MHz, $CDCl_3$)

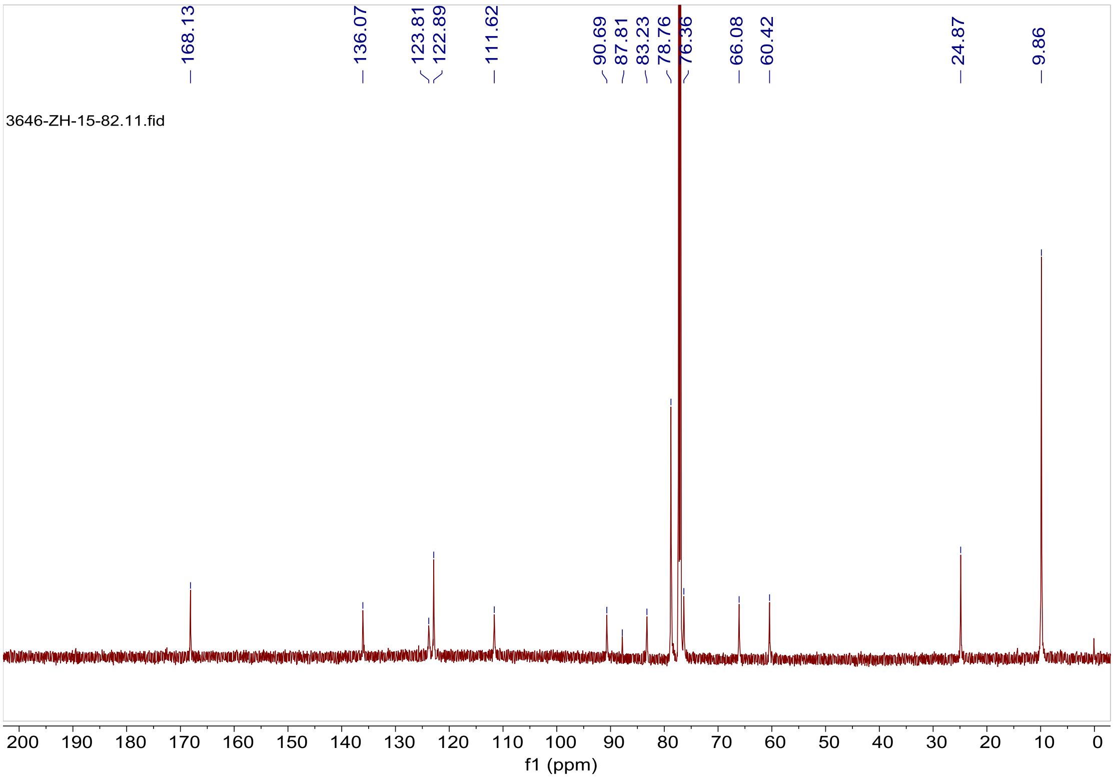

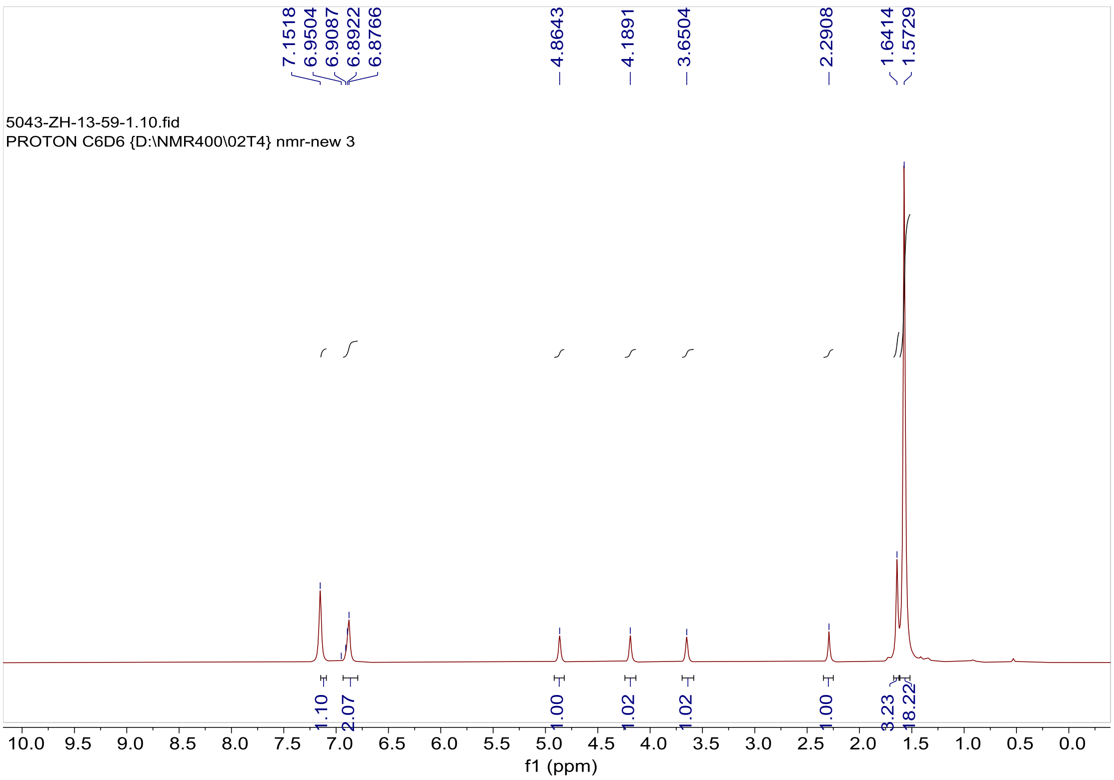

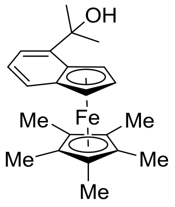

1h

^{1}H NMR (400 MHz, C_6D_6)

^{13}C NMR (100 MHz, C_6D_6)

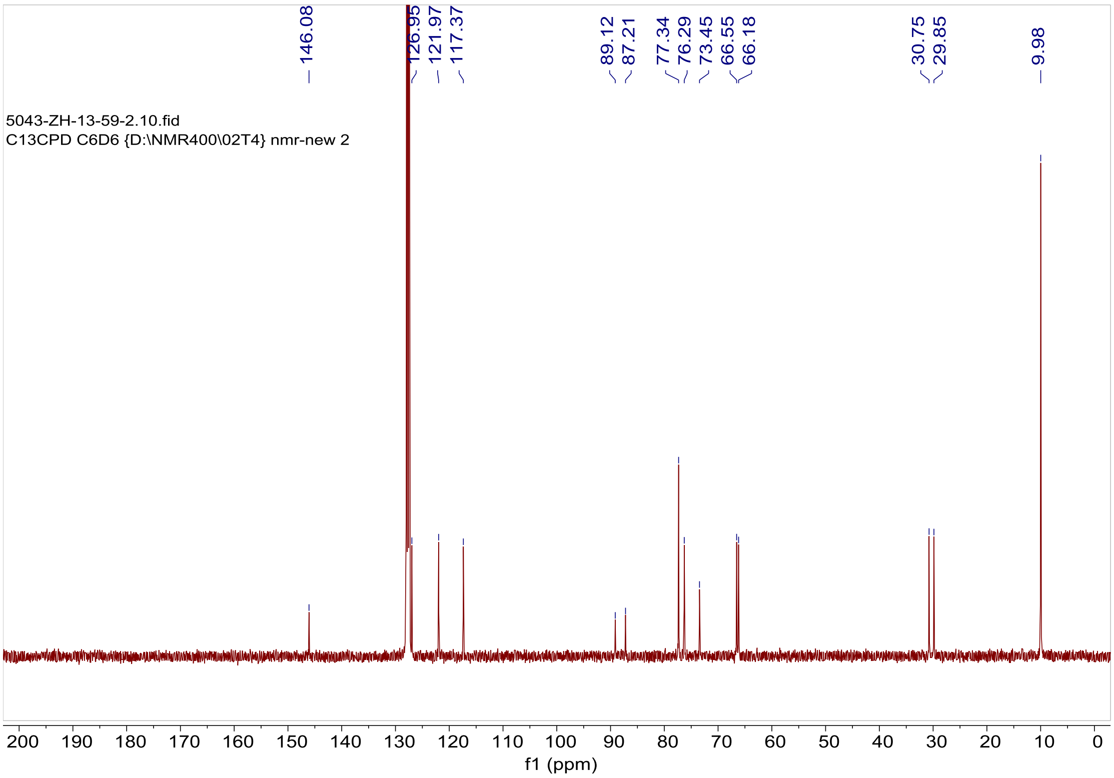

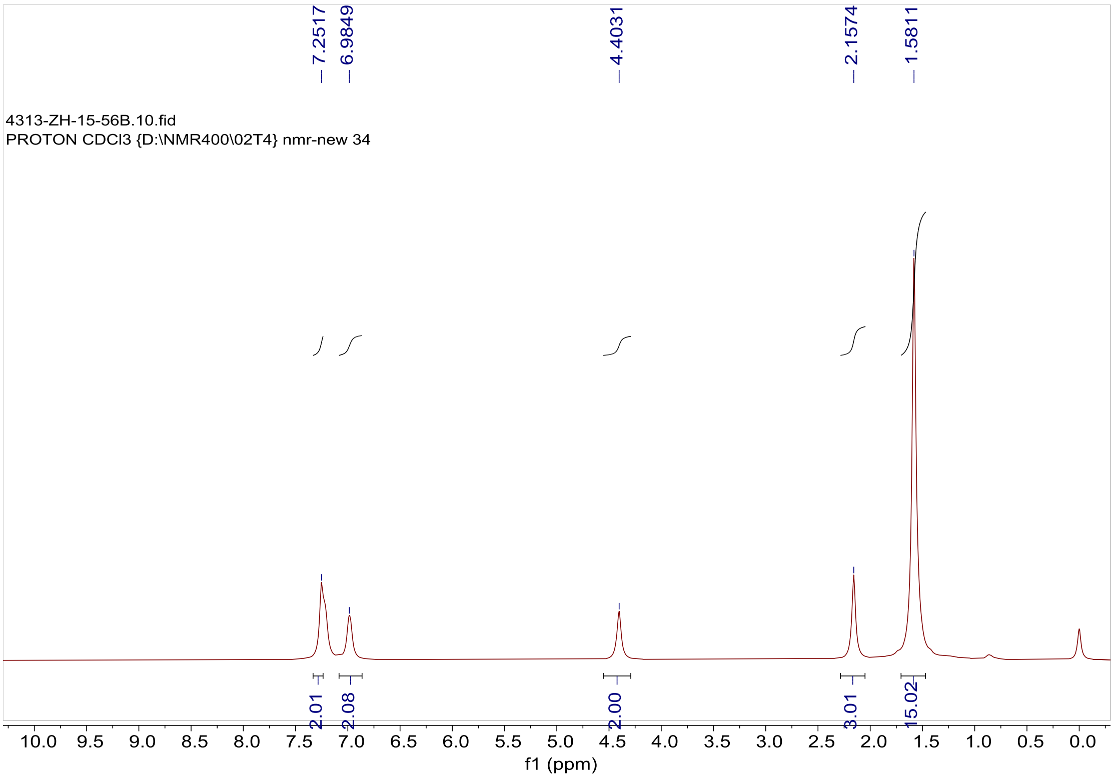

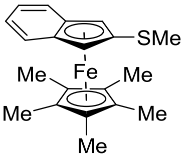

1k

^{1}H NMR (400 MHz, $CDCl_3$)

^{13}C NMR (100 MHz, $CDCl_3$)

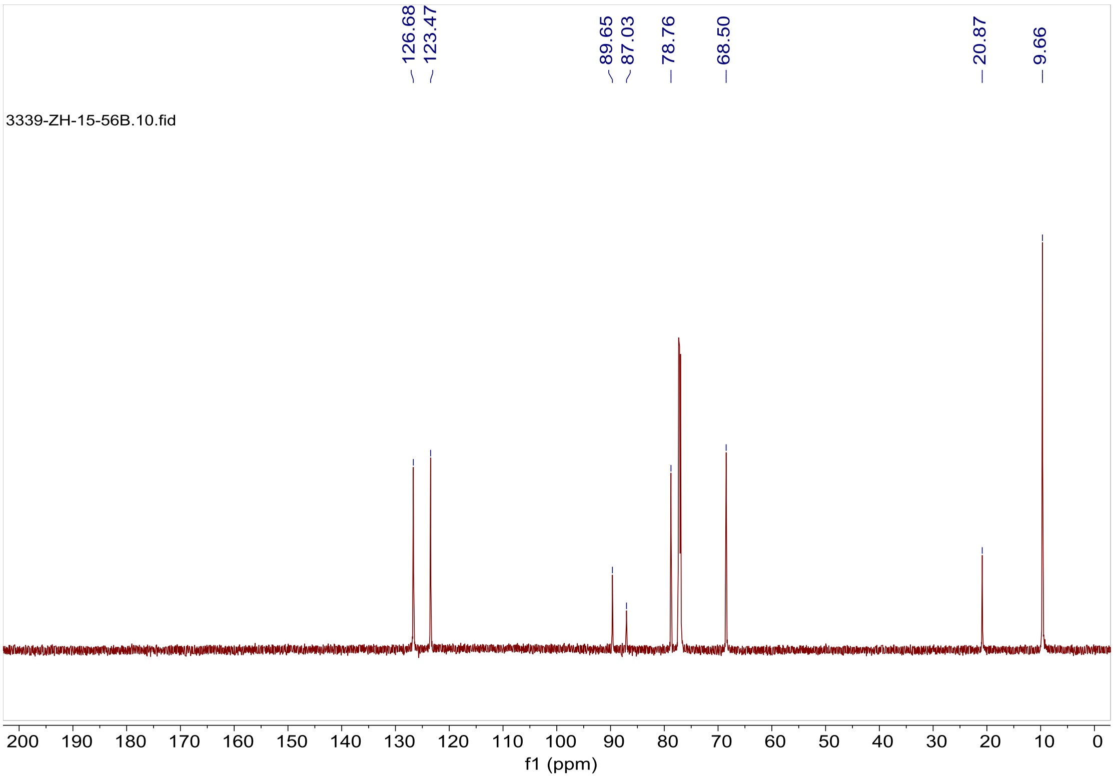

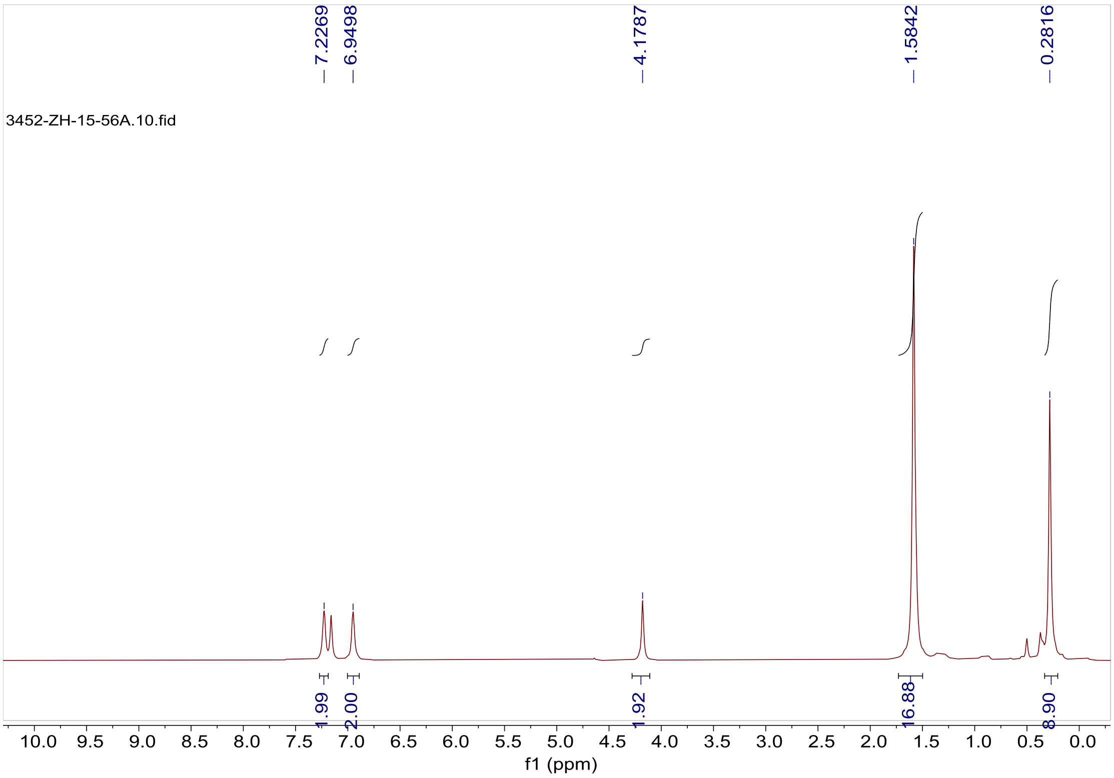

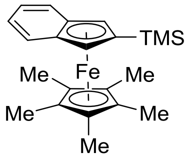

1l

^{1}H NMR (700 MHz, C_6D_6)
^{13}C NMR (175 MHz, C_6D_6)

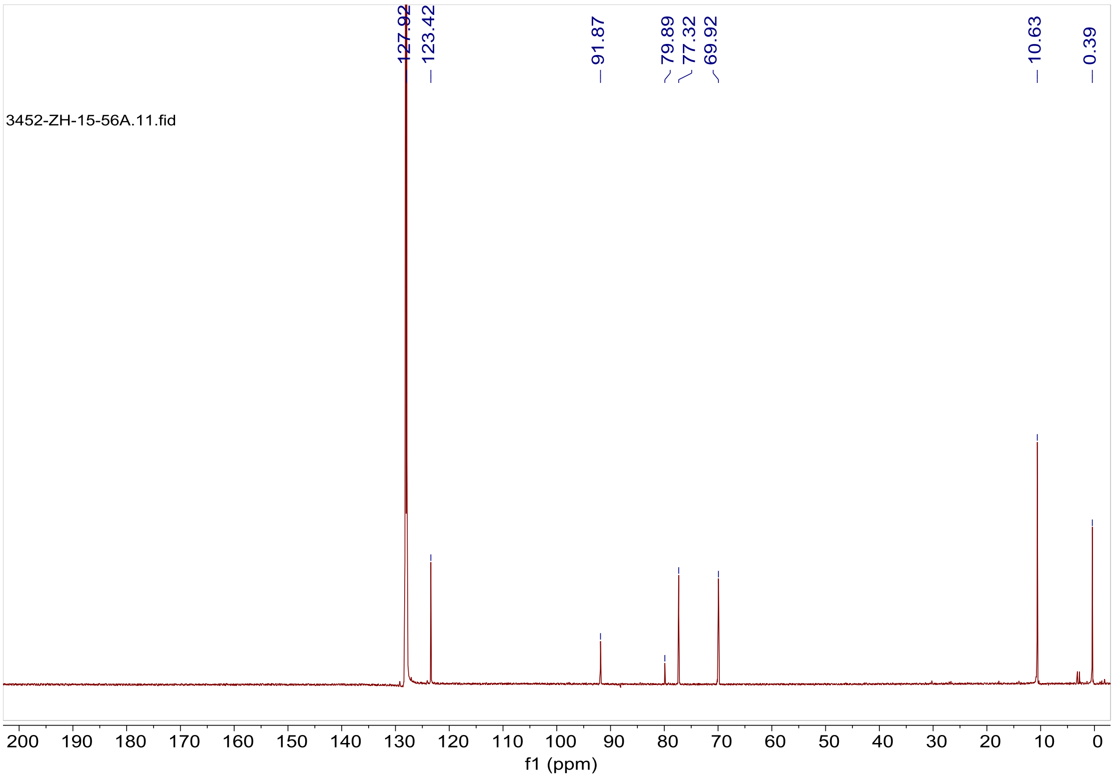

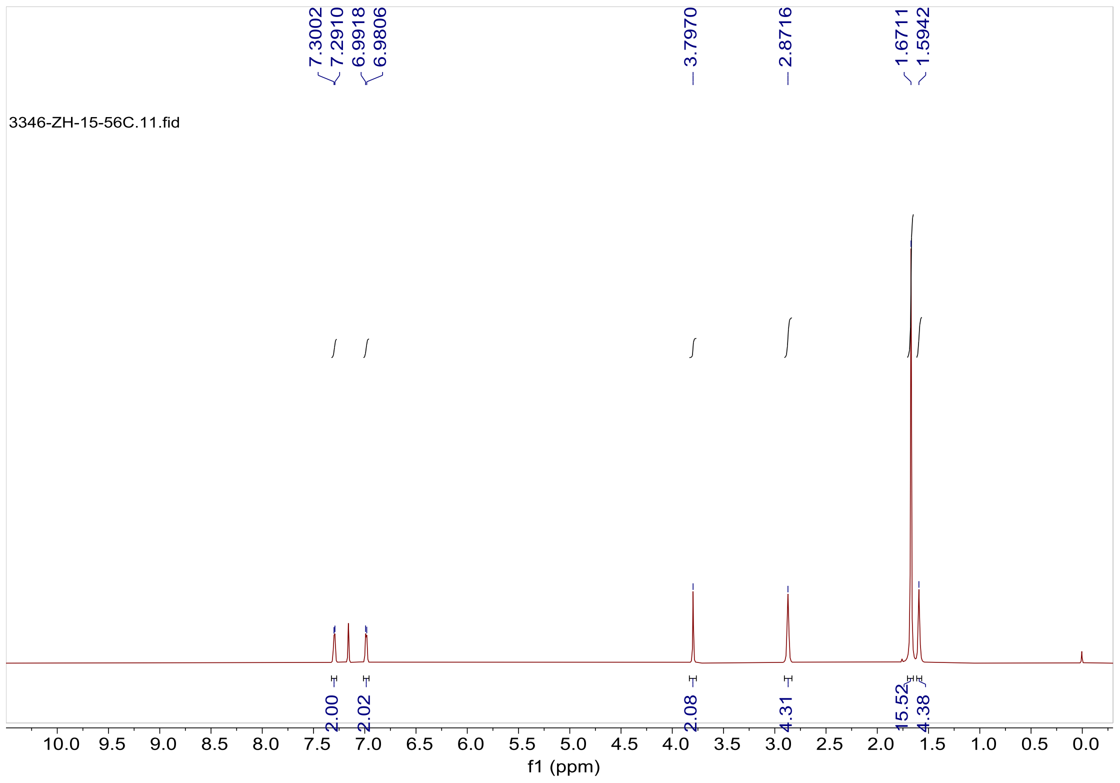

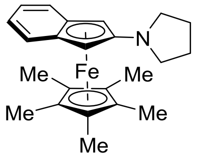

1m

^{1}H NMR (700 MHz, C_6D_6)

^{13}C NMR (175 MHz, C_6D_6)

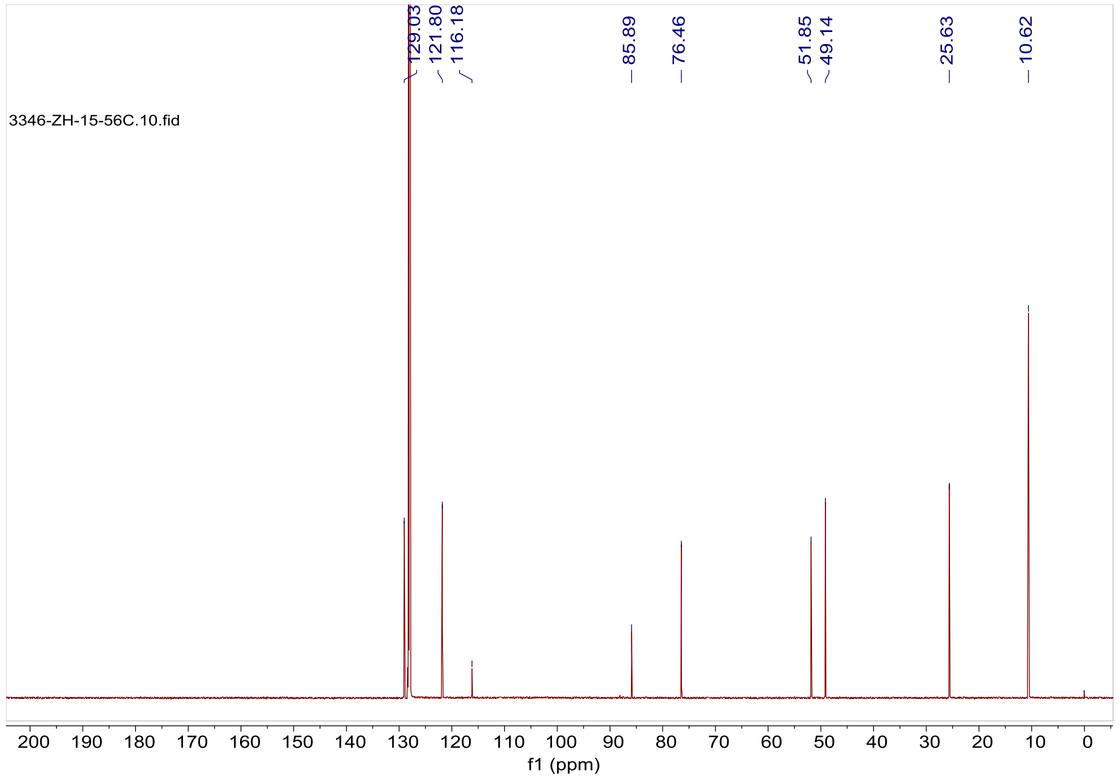

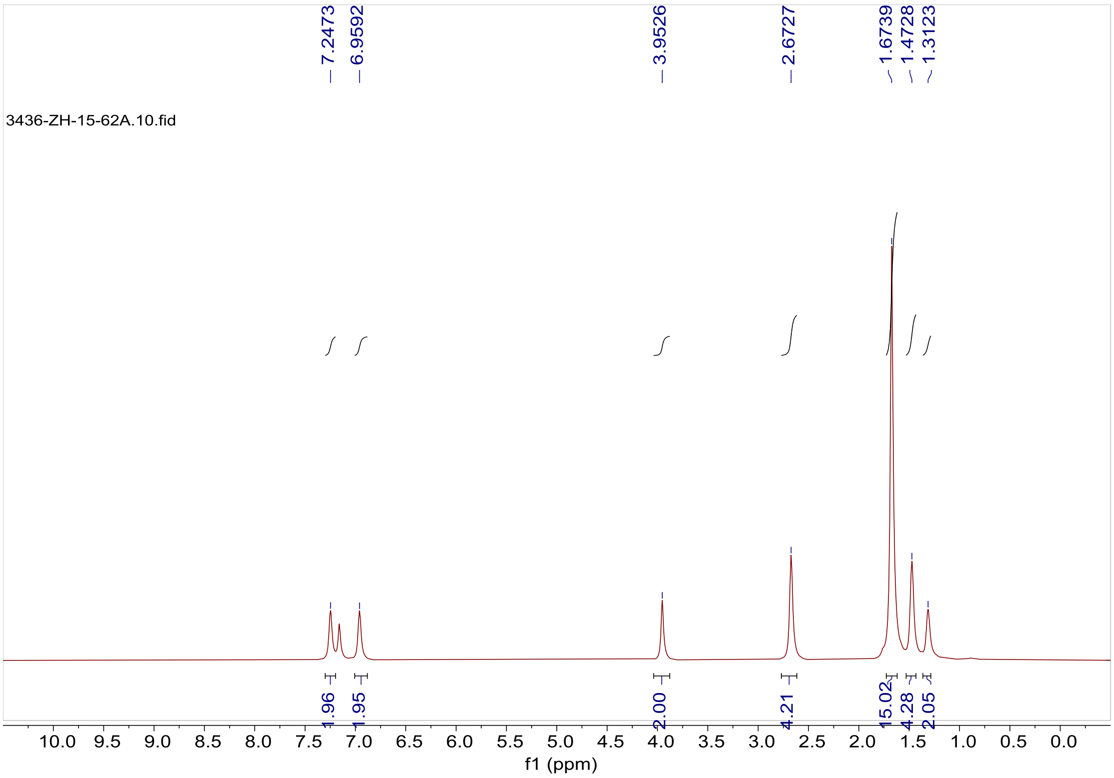

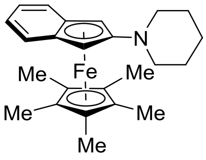

1n

^{1}H NMR (700 MHz, C_6D_6)

^{13}C NMR (175 MHz, C_6D_6)

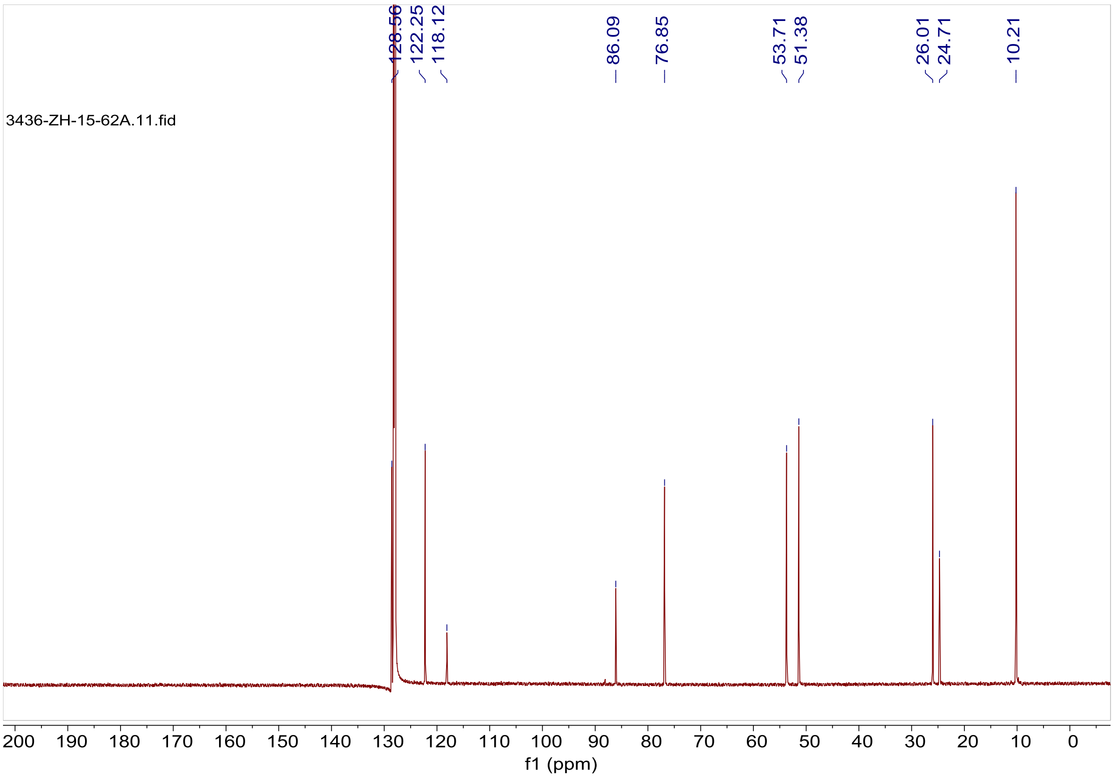

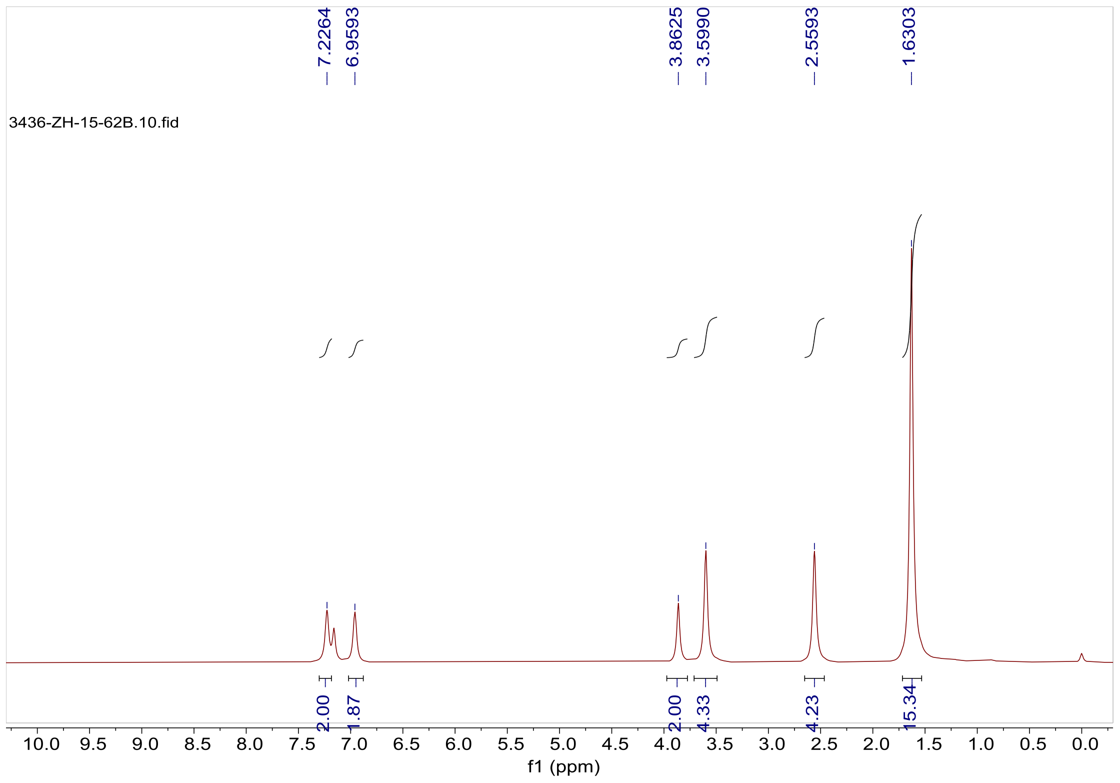

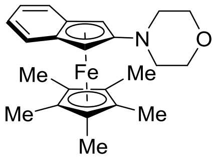

1o

^{1}H NMR (700 MHz, C_6D_6)
^{13}C NMR (175 MHz, C_6D_6)

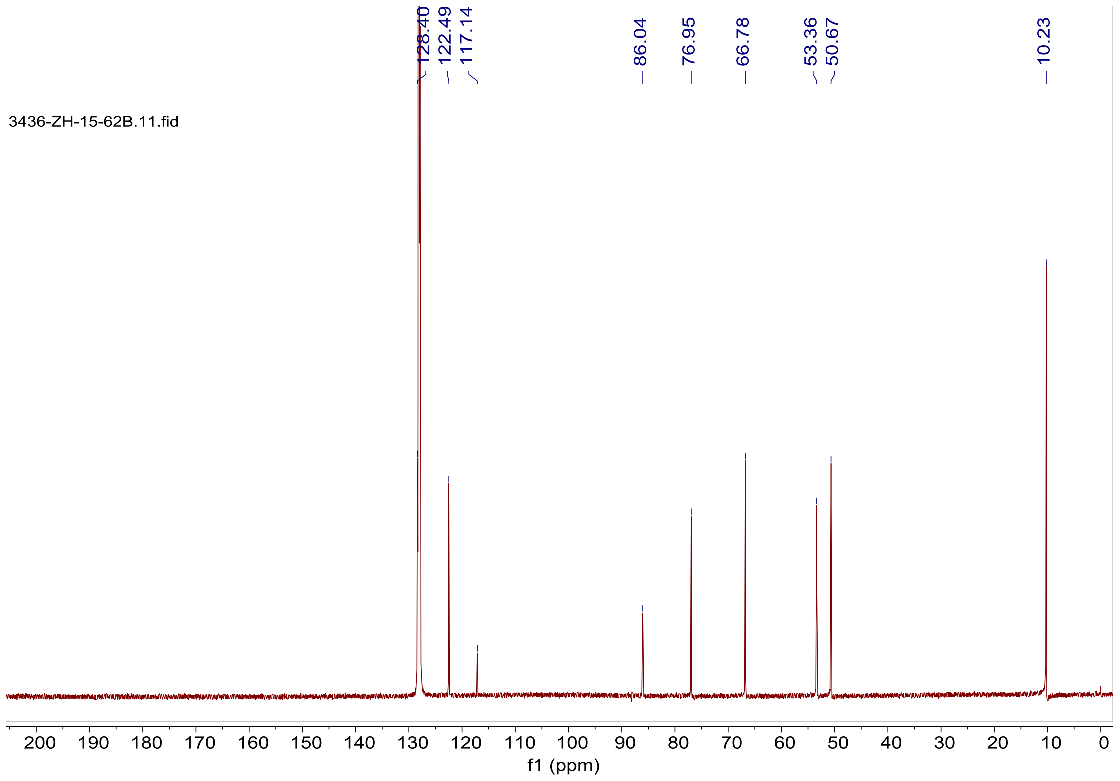

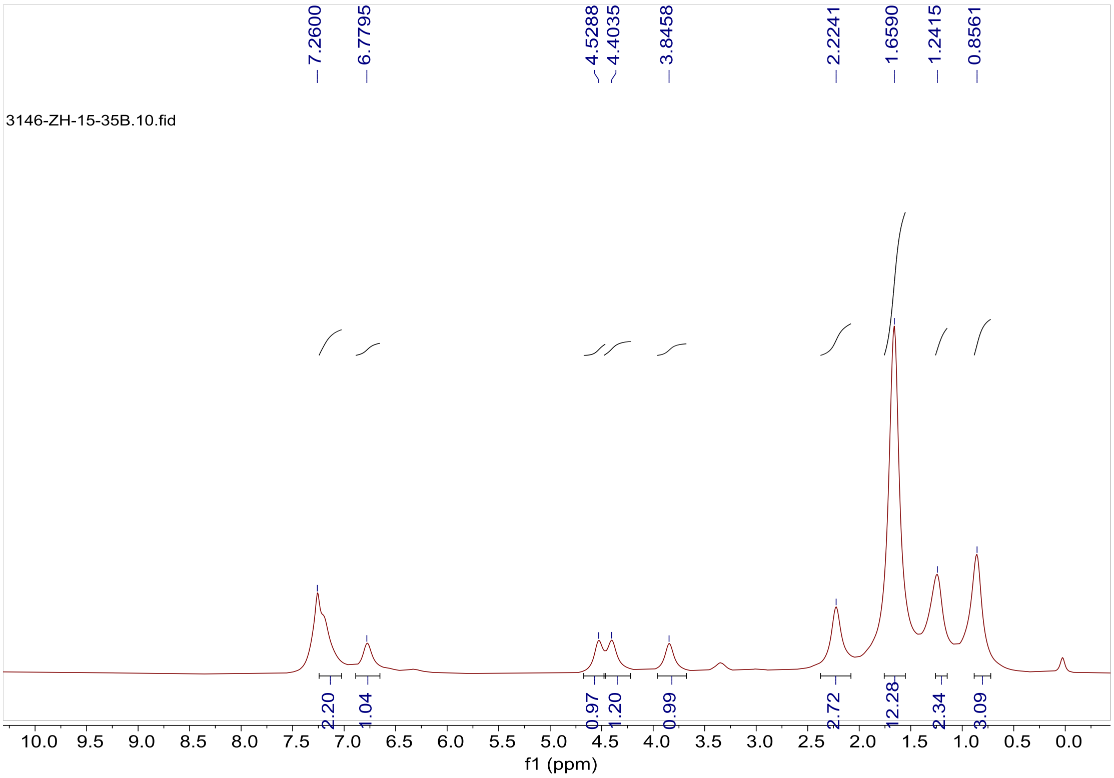

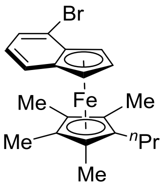

1p

^{1}H NMR (400 MHz, $CDCl_3$)
^{13}C NMR (100 MHz, $CDCl_3$)

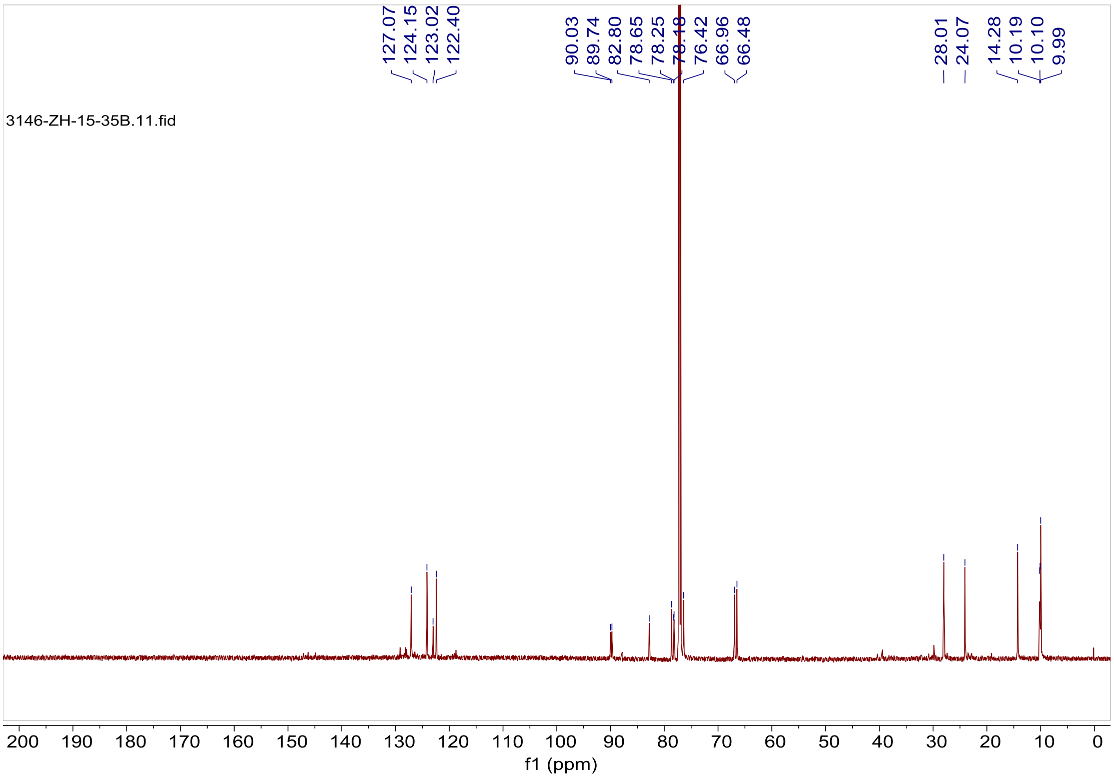

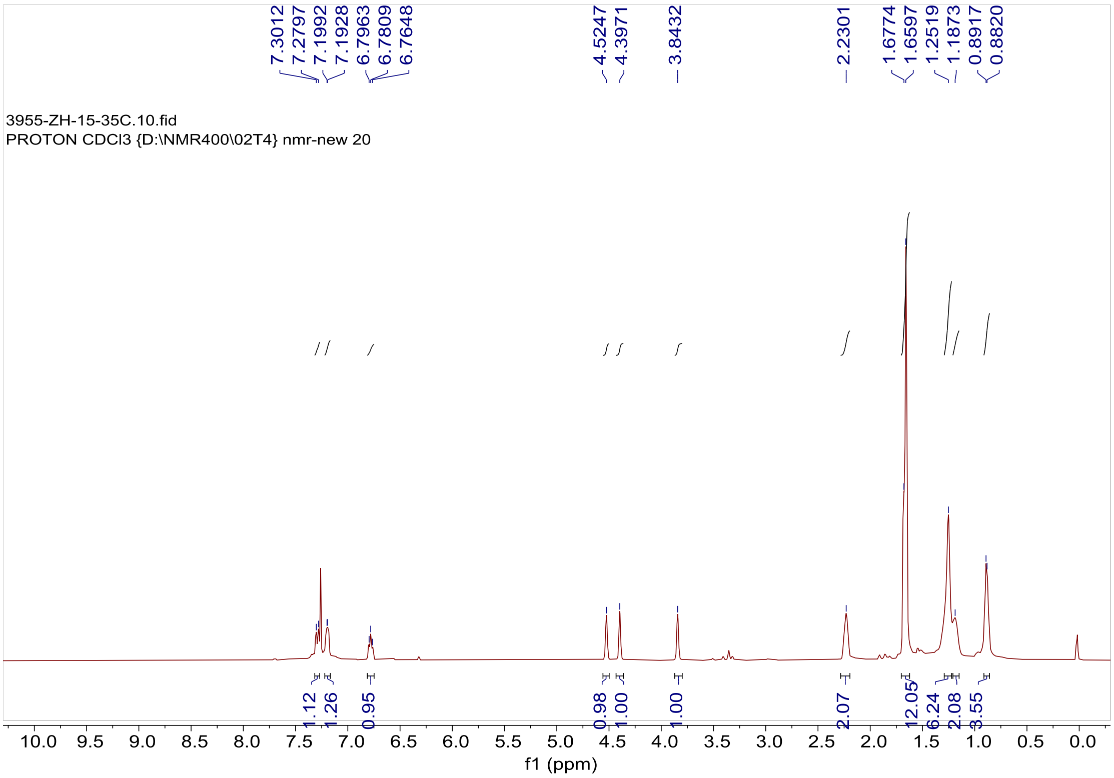

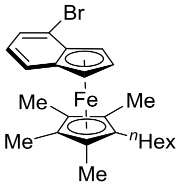

1q

^{1}H NMR (400 MHz, $CDCl_3$)
^{13}C NMR (100 MHz, $CDCl_3$)

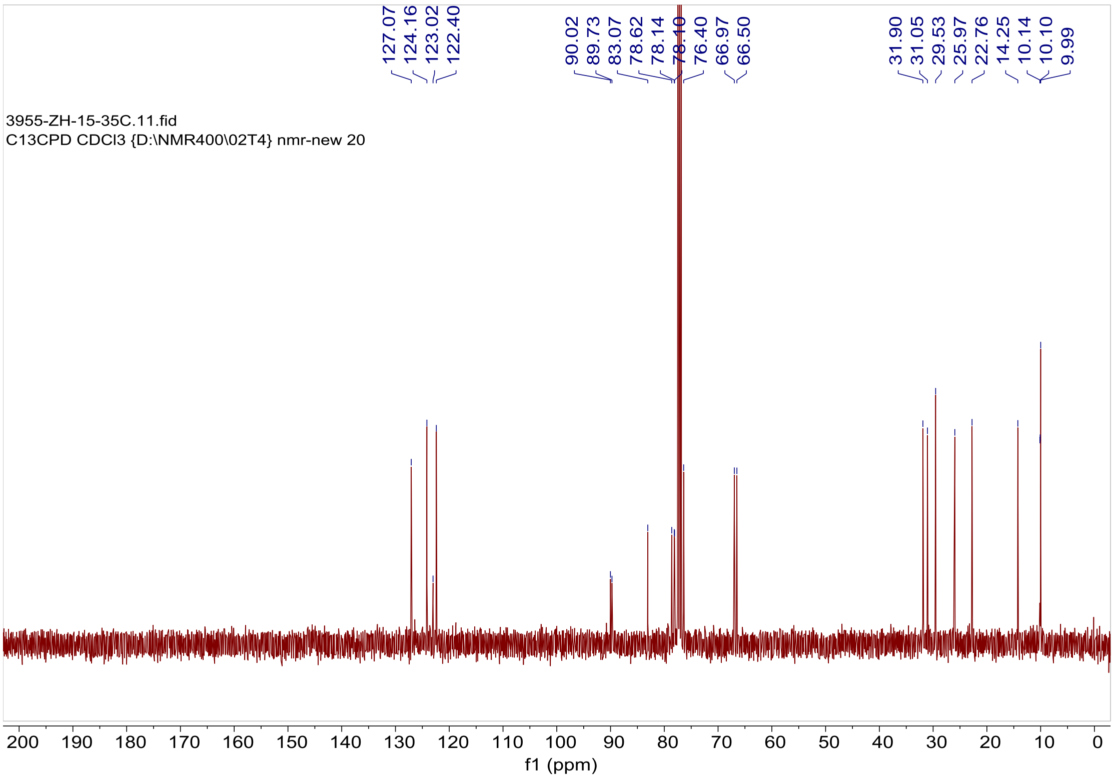

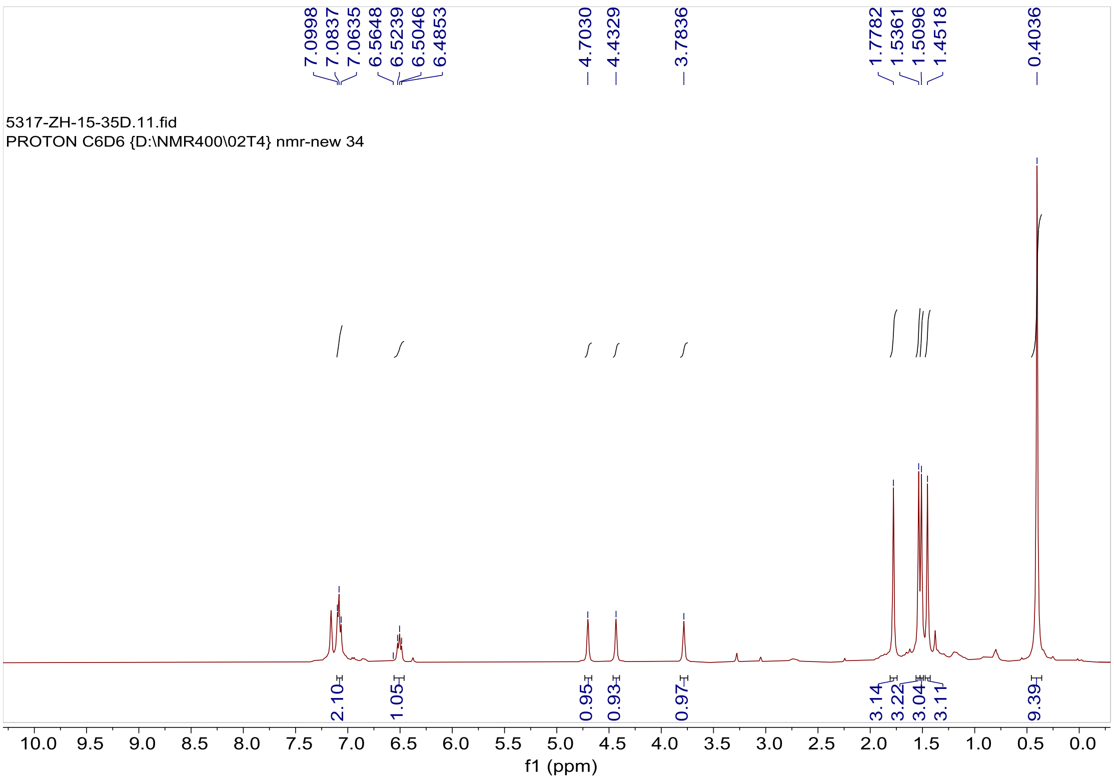

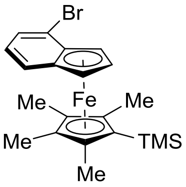

1r

^{1}H NMR (400 MHz, C_6D_6)

^{13}C NMR (100 MHz, C_6D_6)

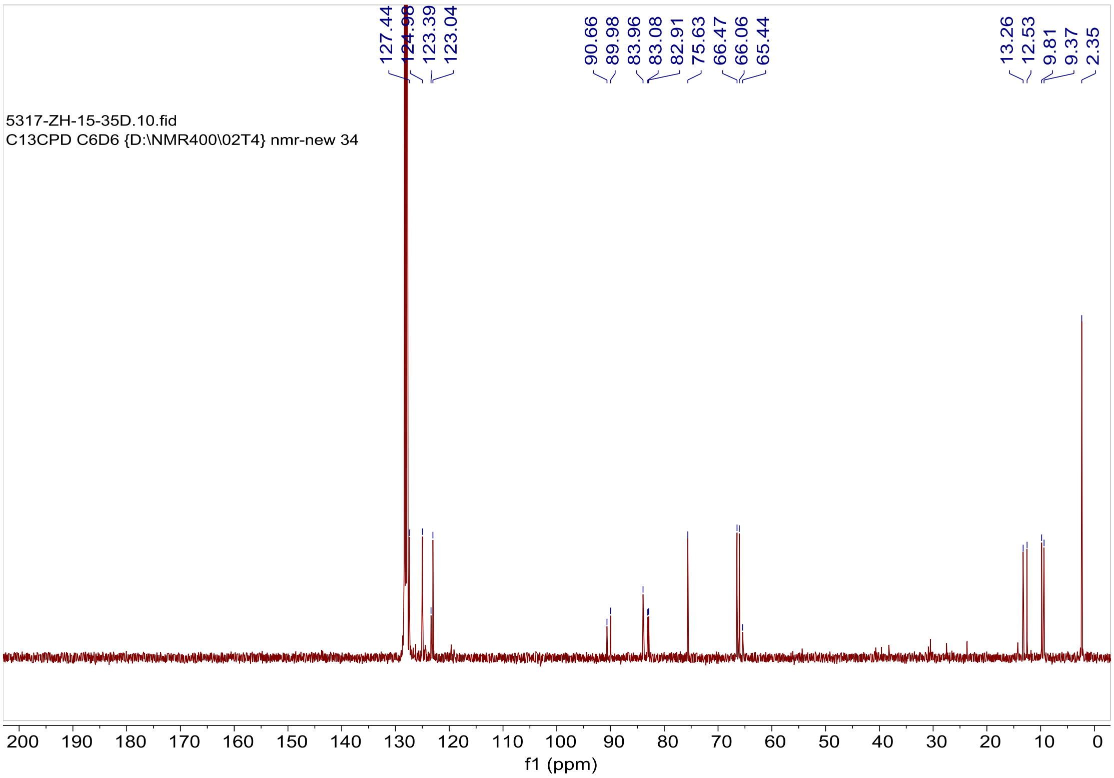

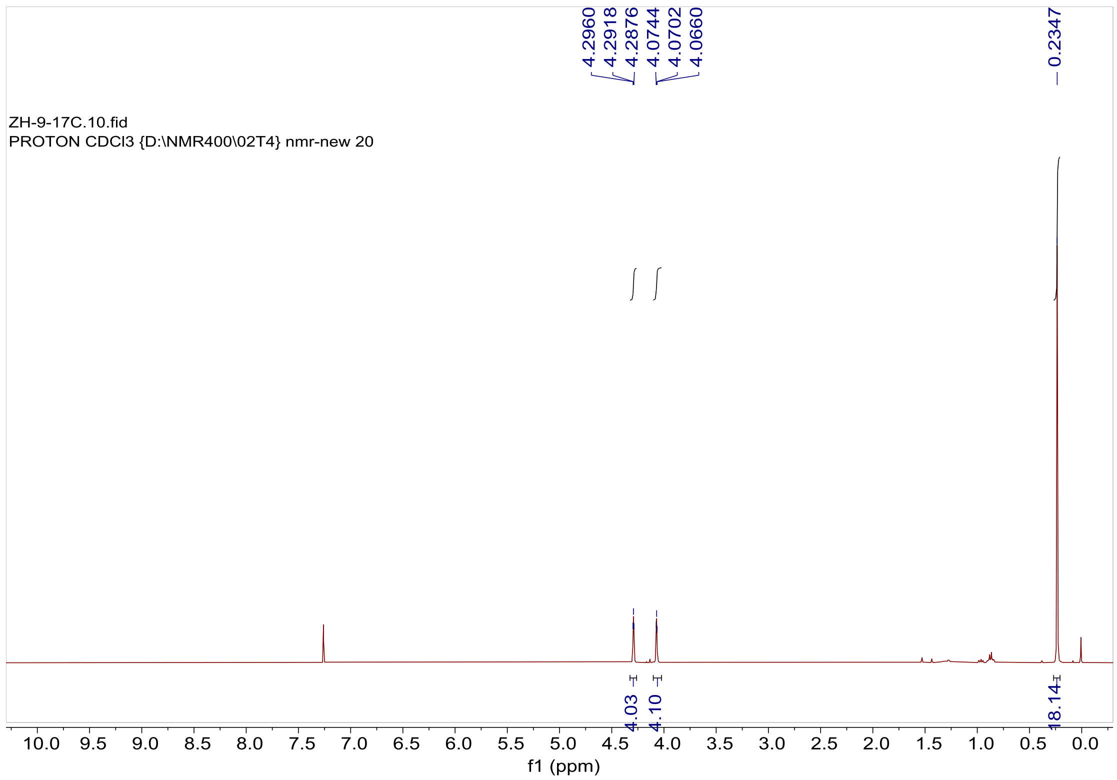

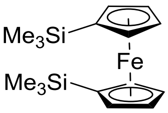

4d

^{1}H NMR (400 MHz, $CDCl_3$)

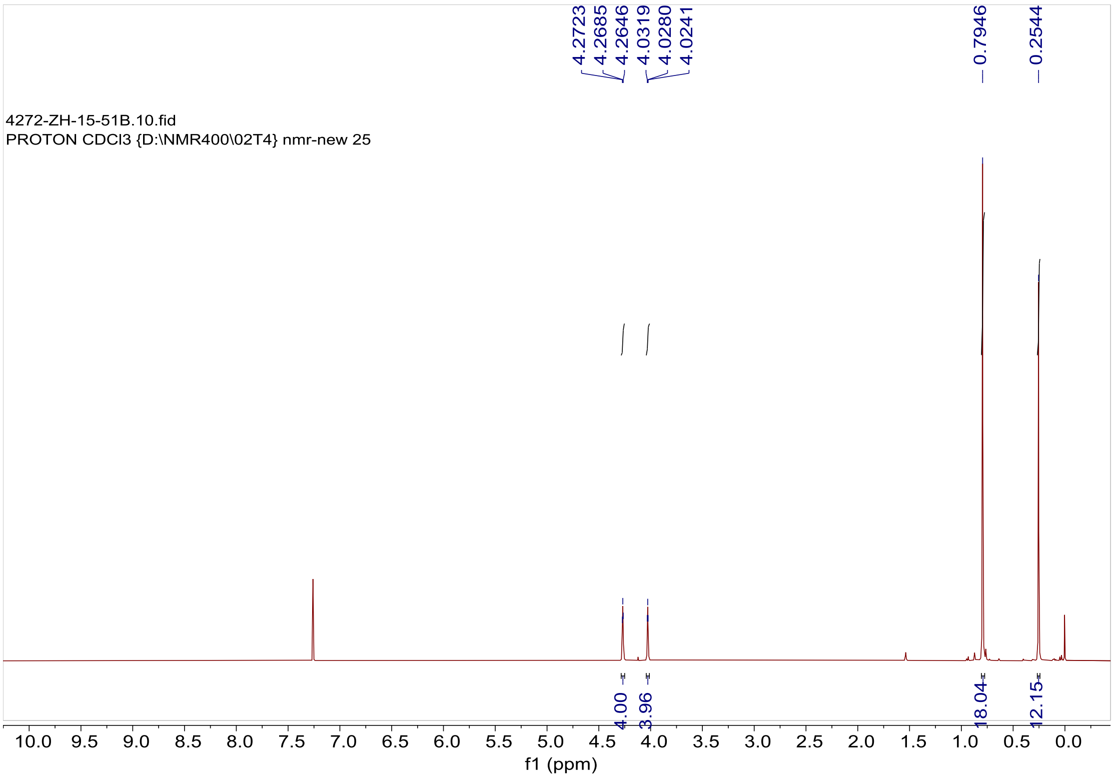

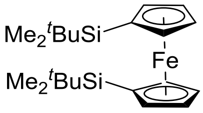

4e

^{1}H NMR (700 MHz, $CDCl_3$)
^{13}C NMR (175 MHz, $CDCl_3$)

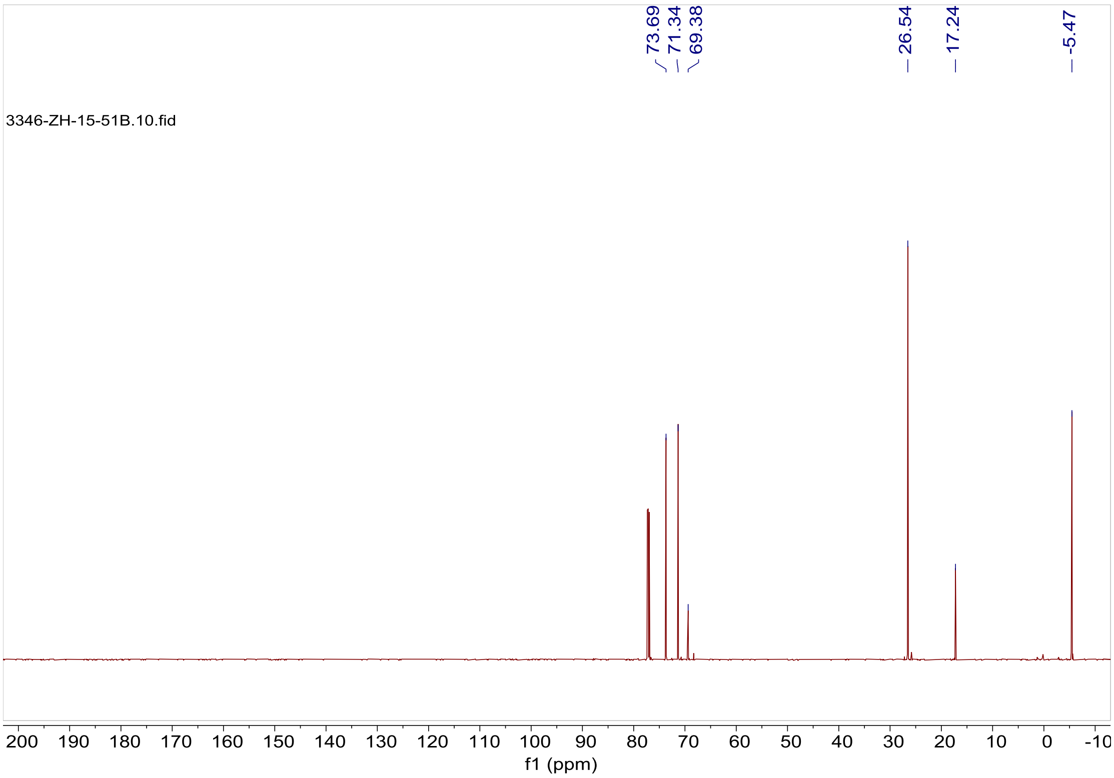

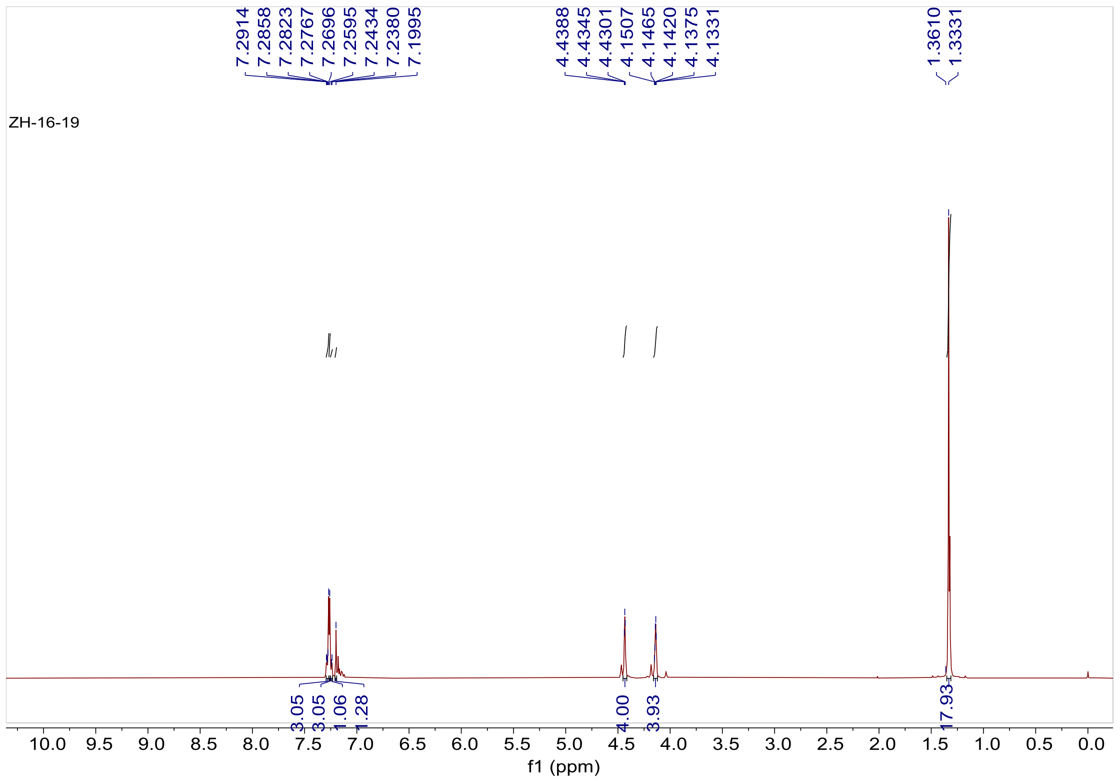

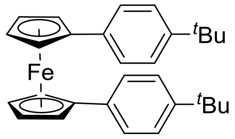

4f

^{1}H NMR (400 MHz, $CDCl_3$)

^{13}C NMR (100 MHz, $CDCl_3$)

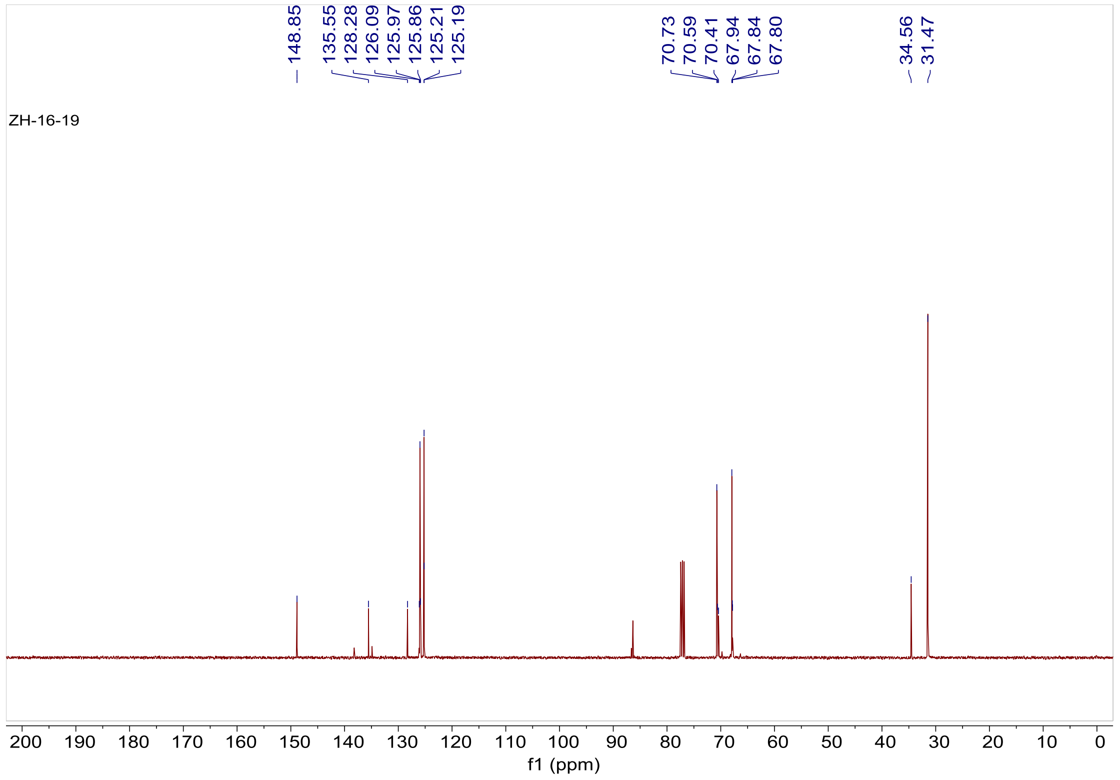

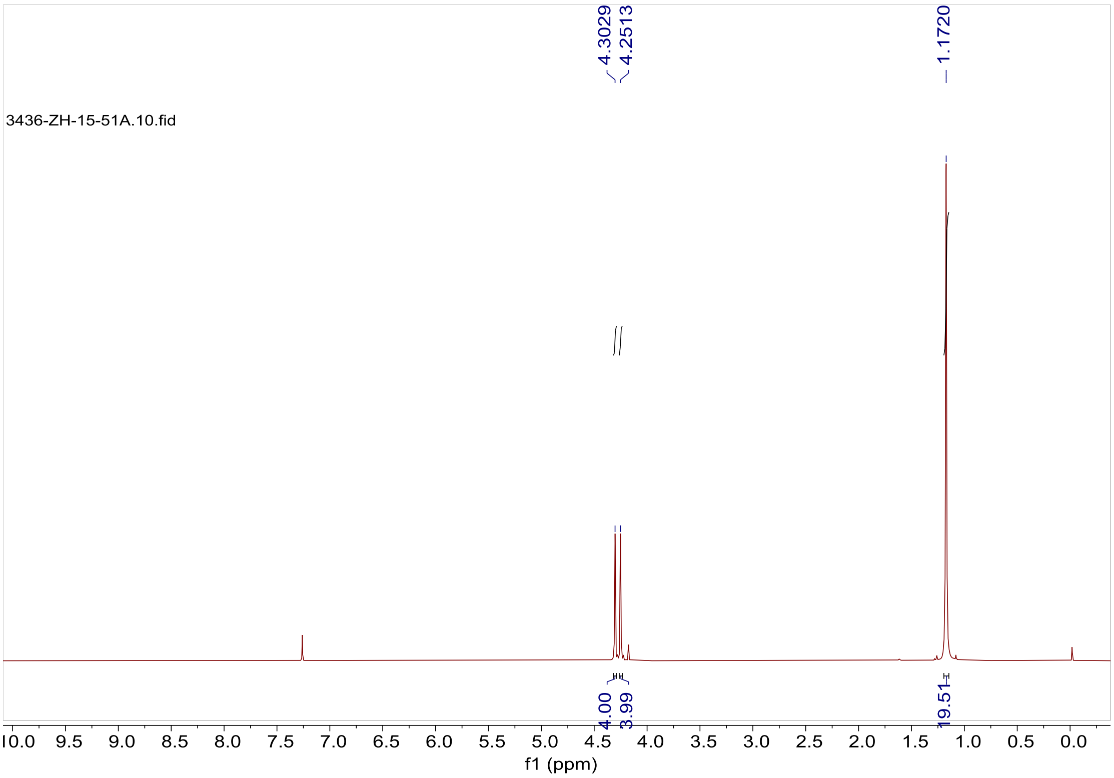

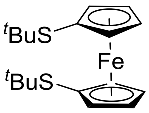

4g

^{1}H NMR (700 MHz, $CDCl_3$)
^{13}C NMR (175 MHz, $CDCl_3$)

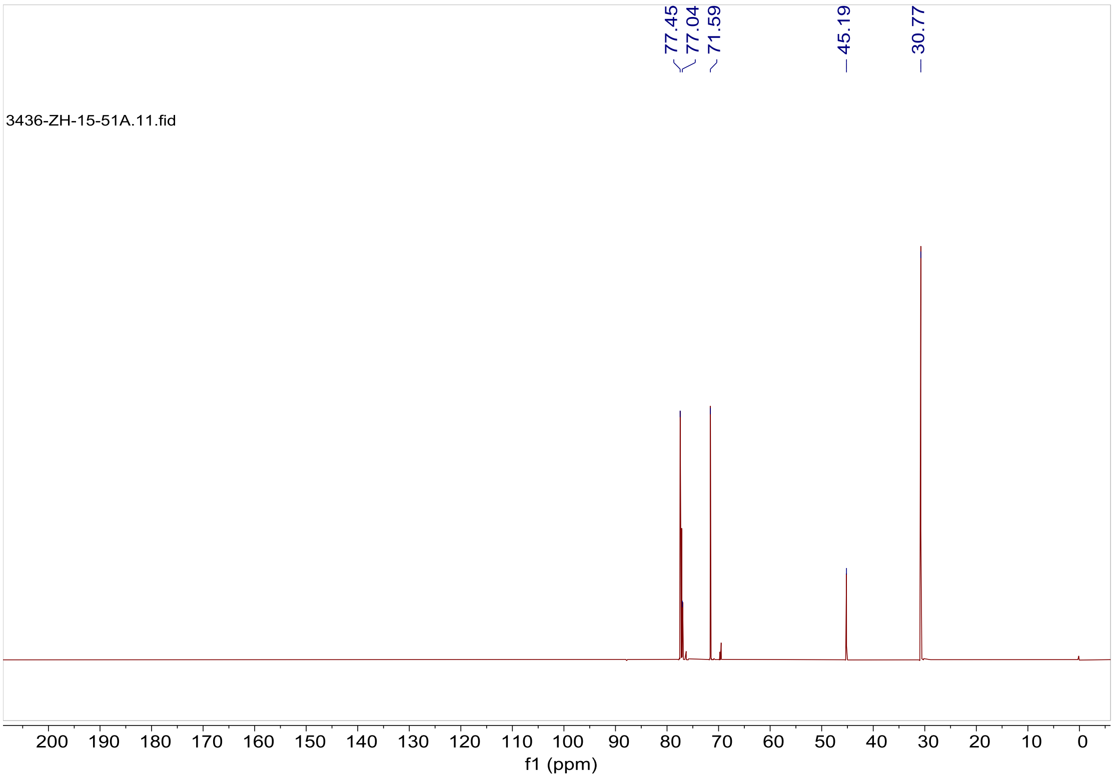

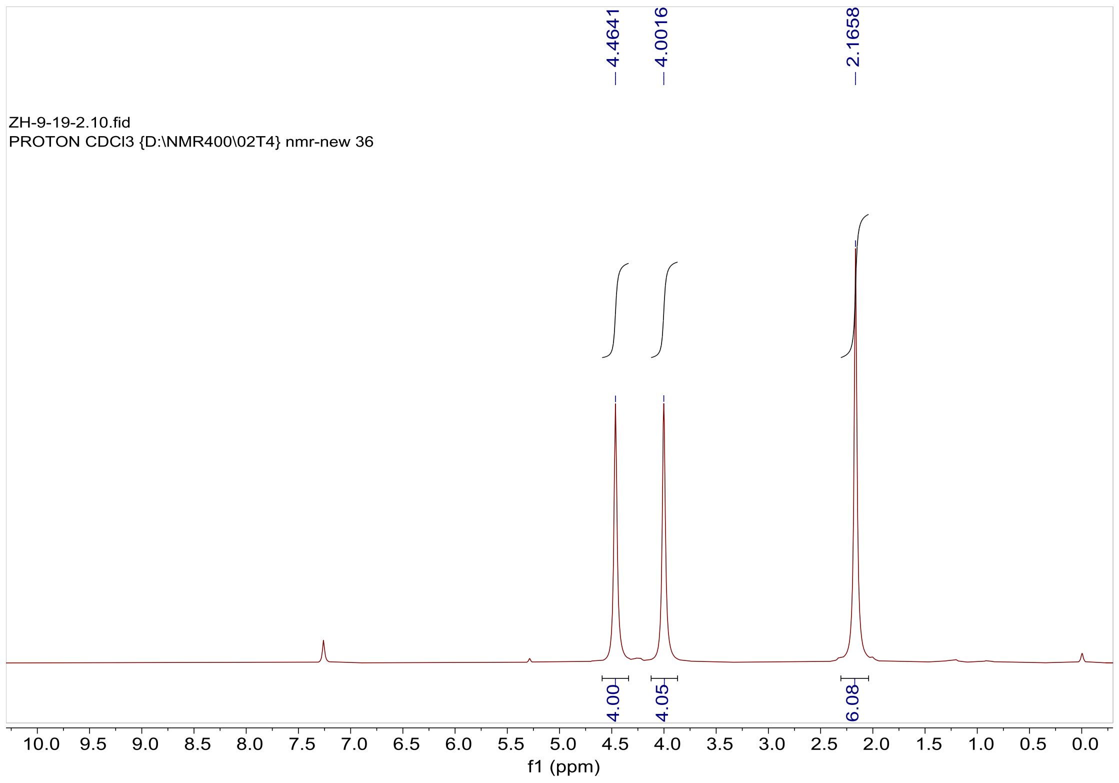

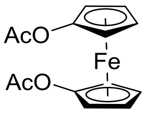

4h

^{1}H NMR (400 MHz, $CDCl_3$)

^{13}C NMR (100 MHz, $CDCl_3$)

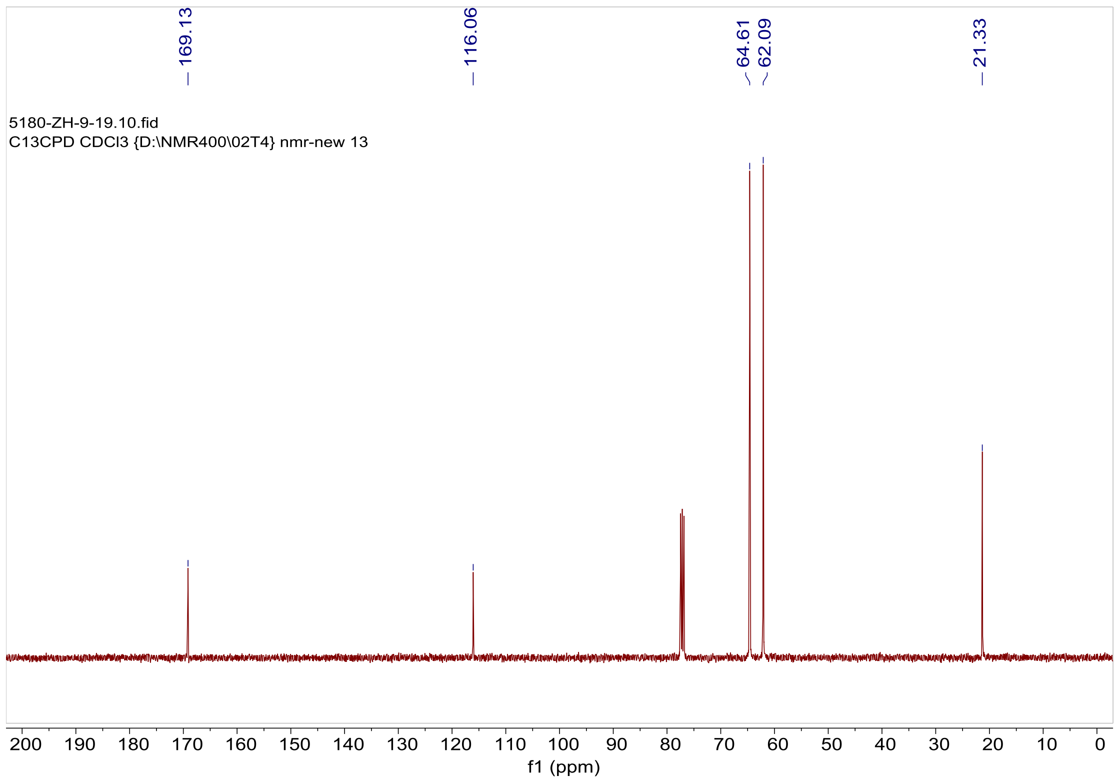

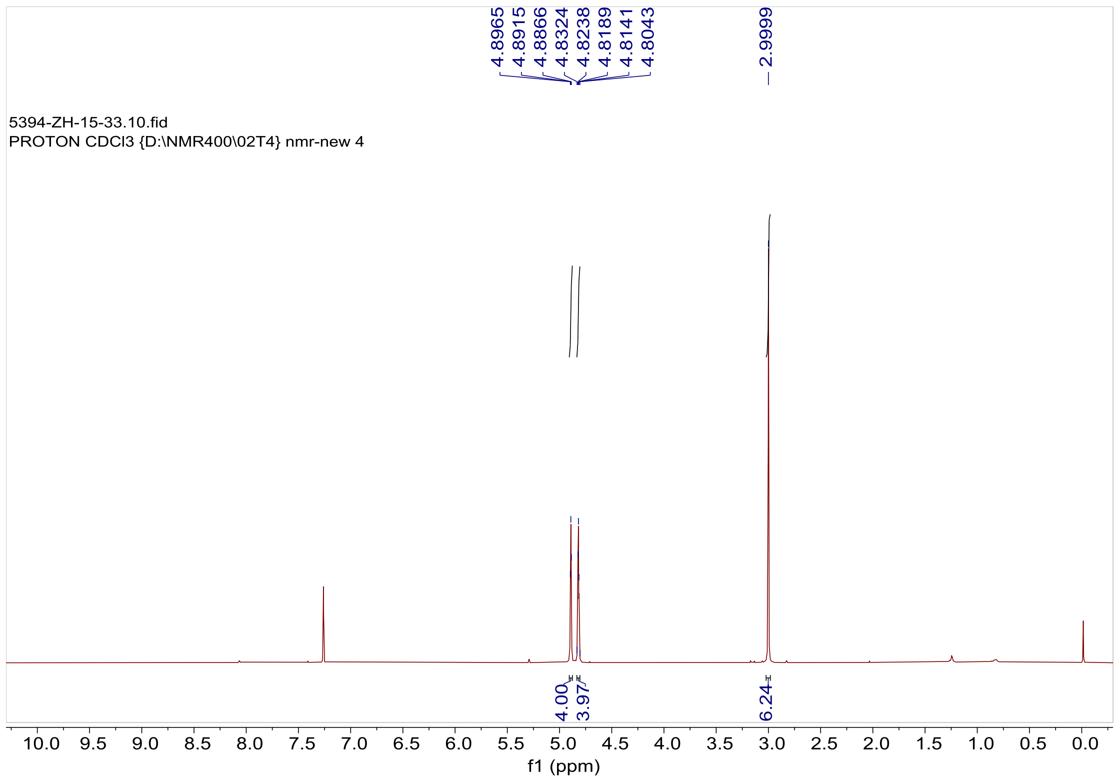

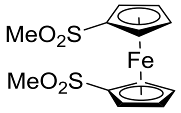

4i

^{1}H NMR (400 MHz, $CDCl_3$)

^{13}C NMR (100 MHz, $CDCl_3$)

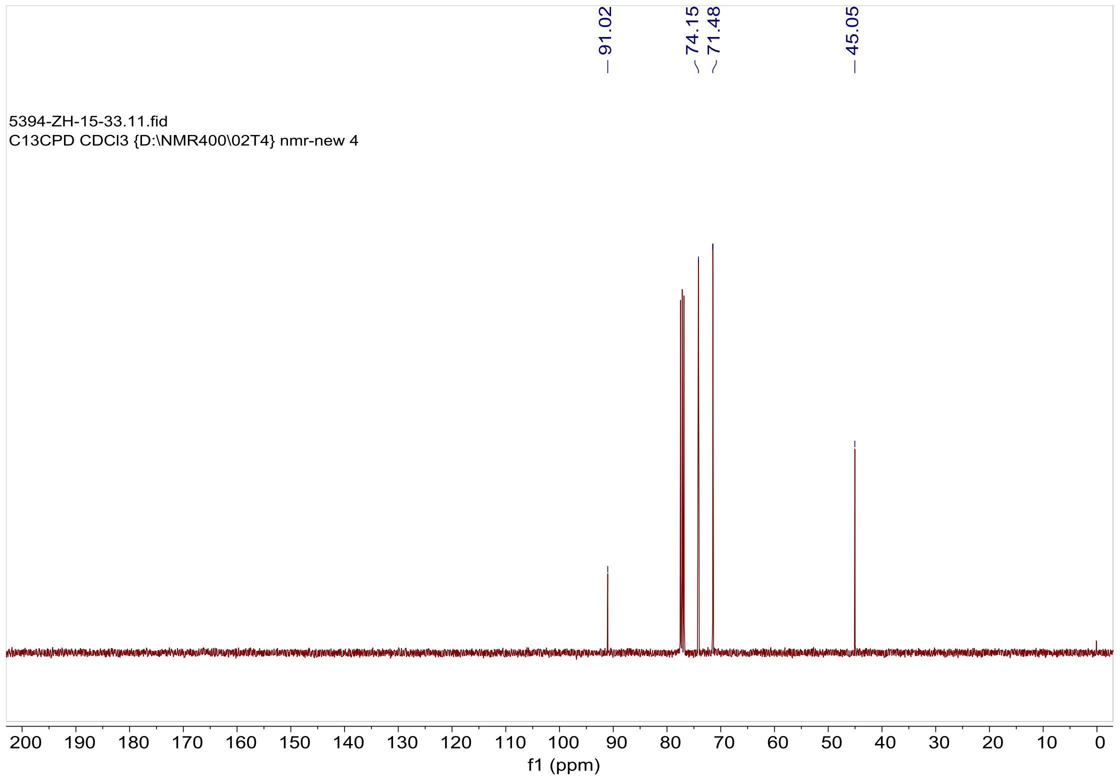

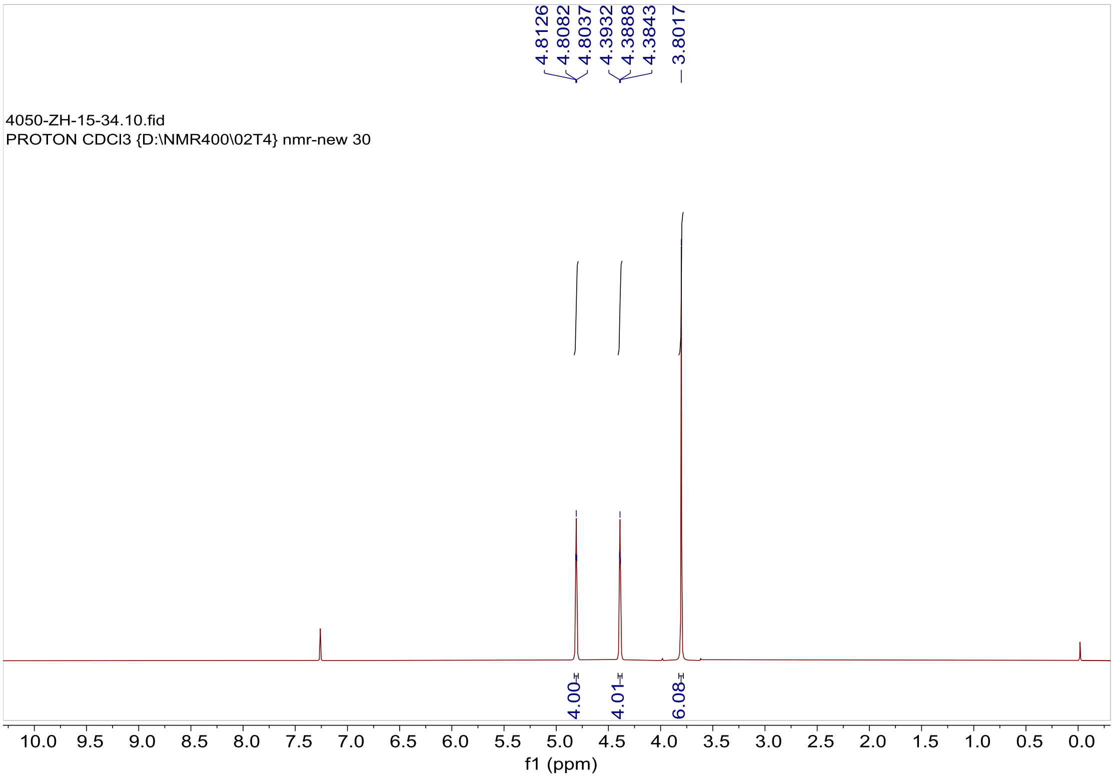

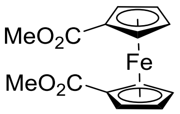

4j

^{1}H NMR (400 MHz, $CDCl_3$)
^{13}C NMR (100 MHz, $CDCl_3$)

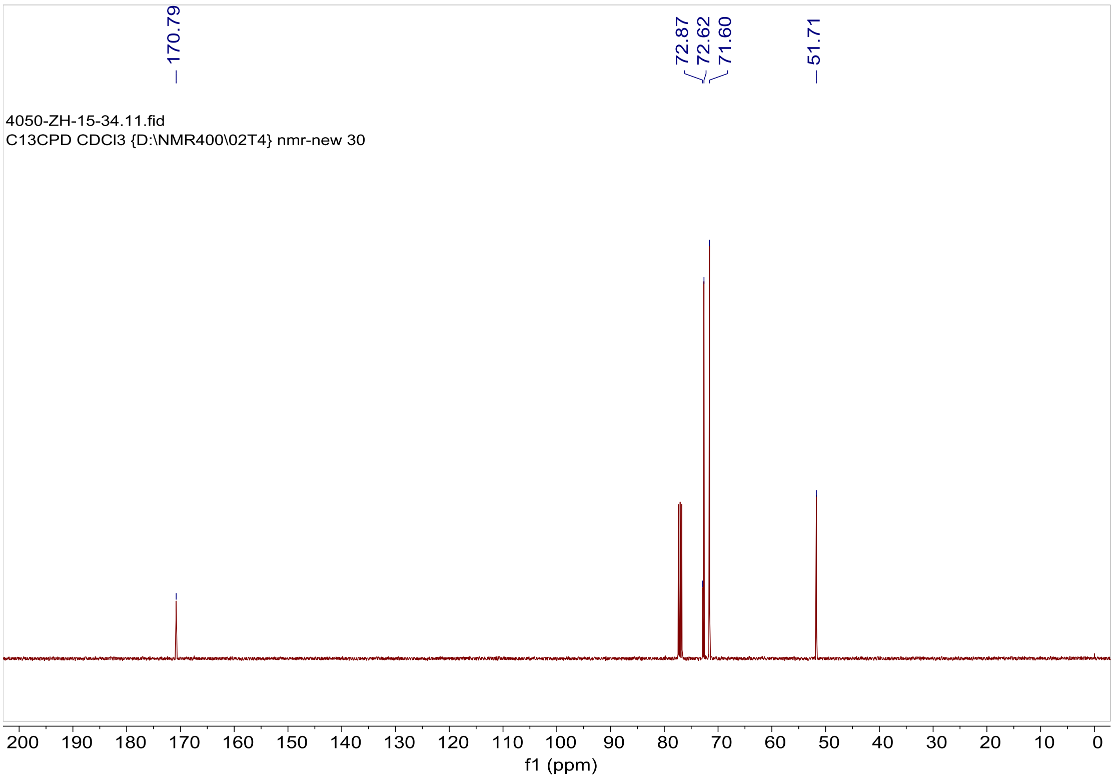

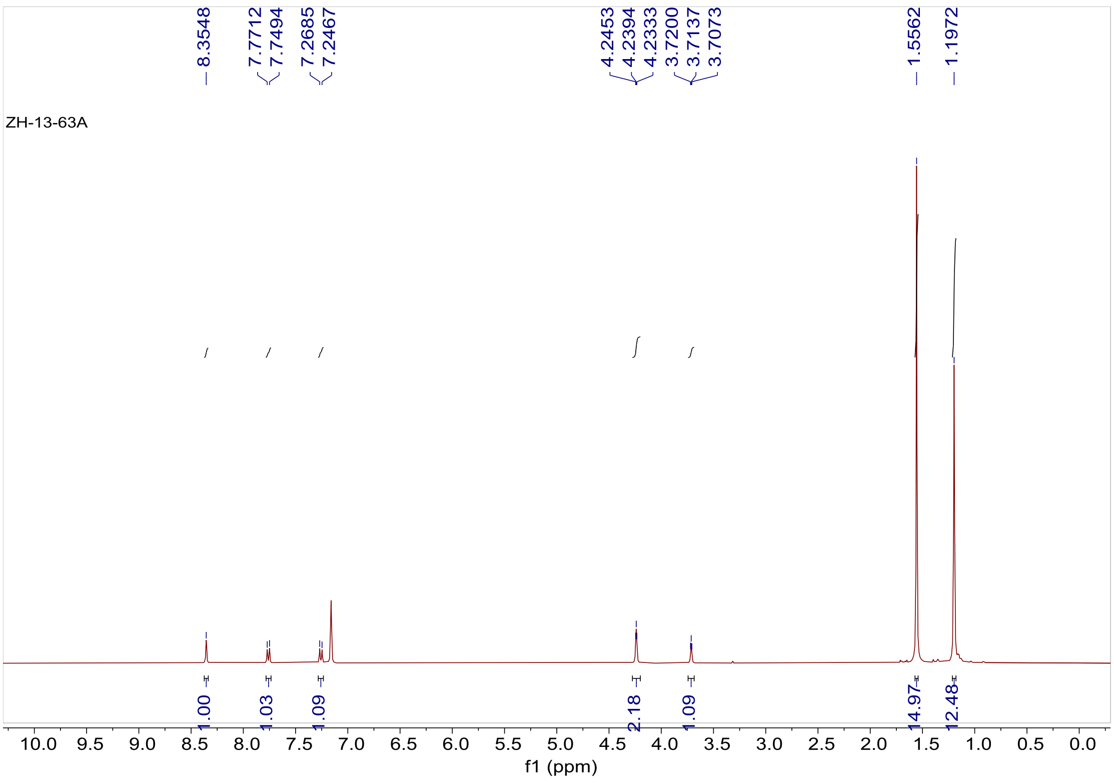

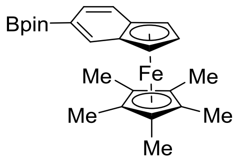

3a

^{1}H NMR (400 MHz, C_6D_6)

^{13}C NMR (100 MHz, $CDCl_3$)

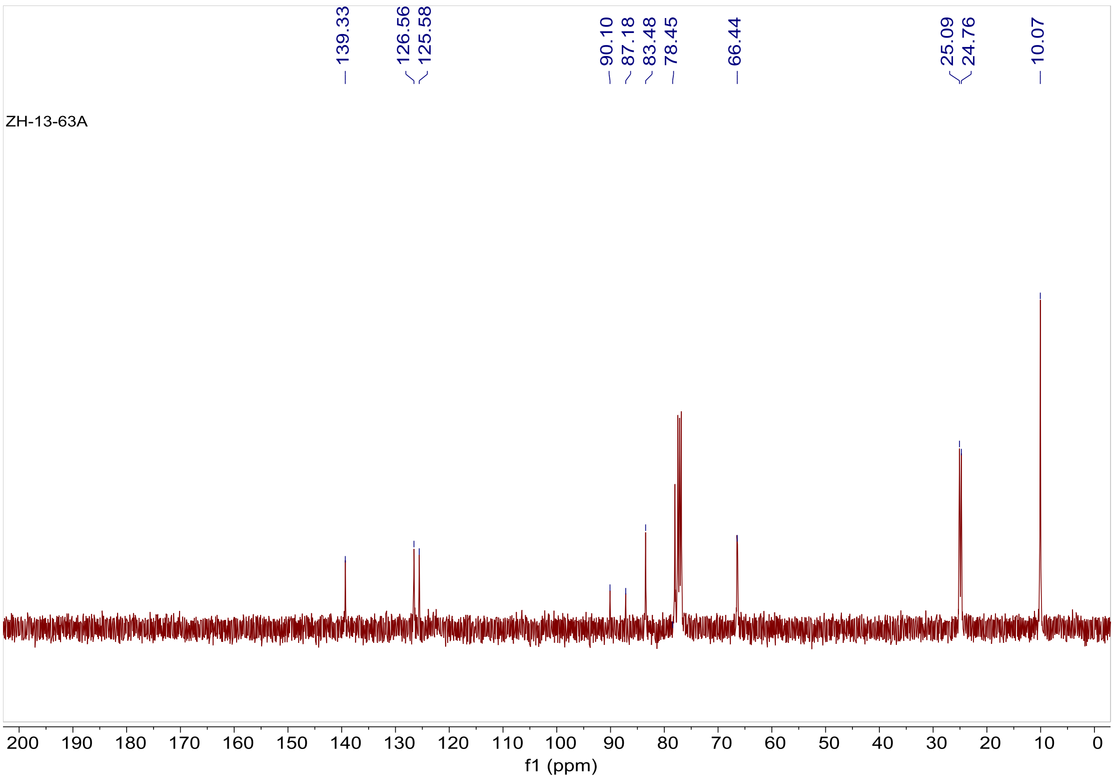

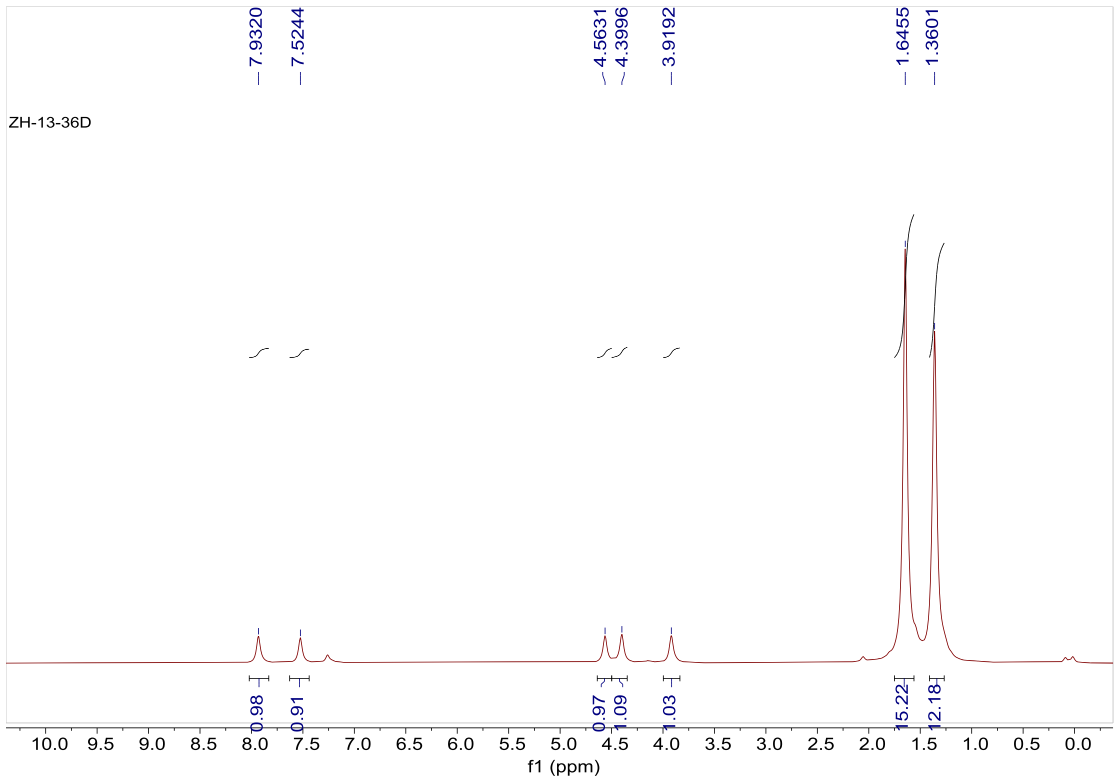

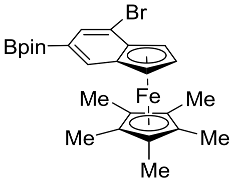

3b

^{1}H NMR (400 MHz, $CDCl_3$)
^{13}C NMR (100 MHz, $CDCl_3$)

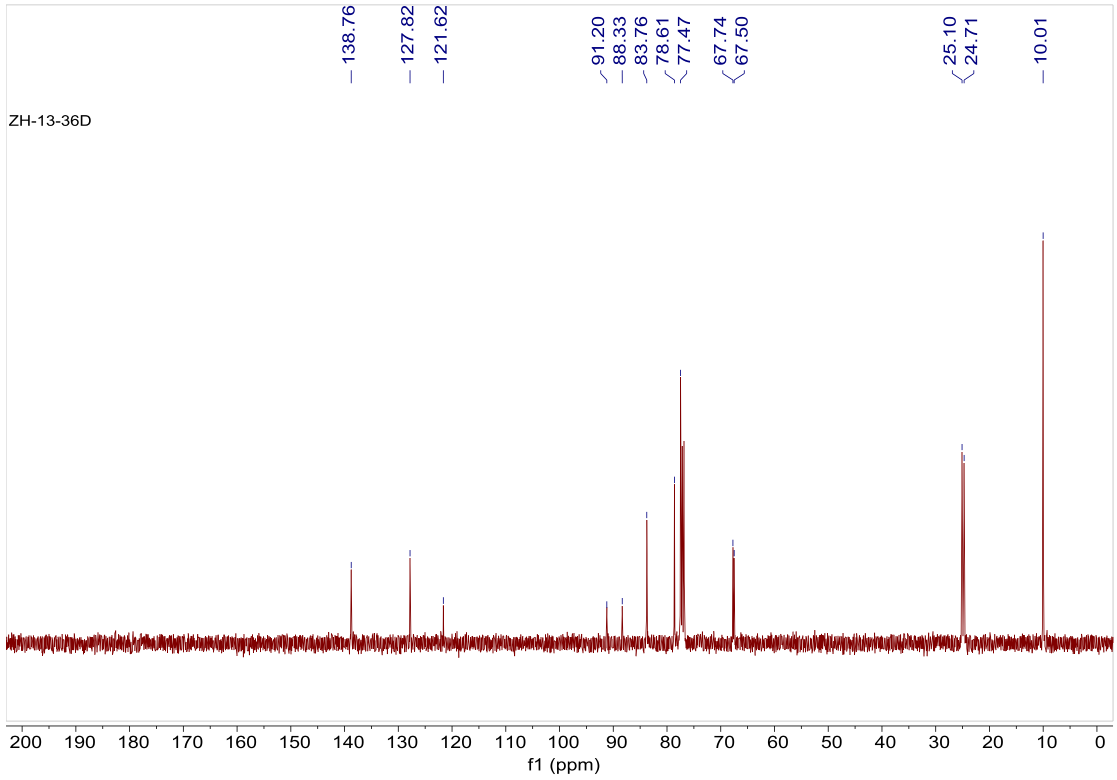

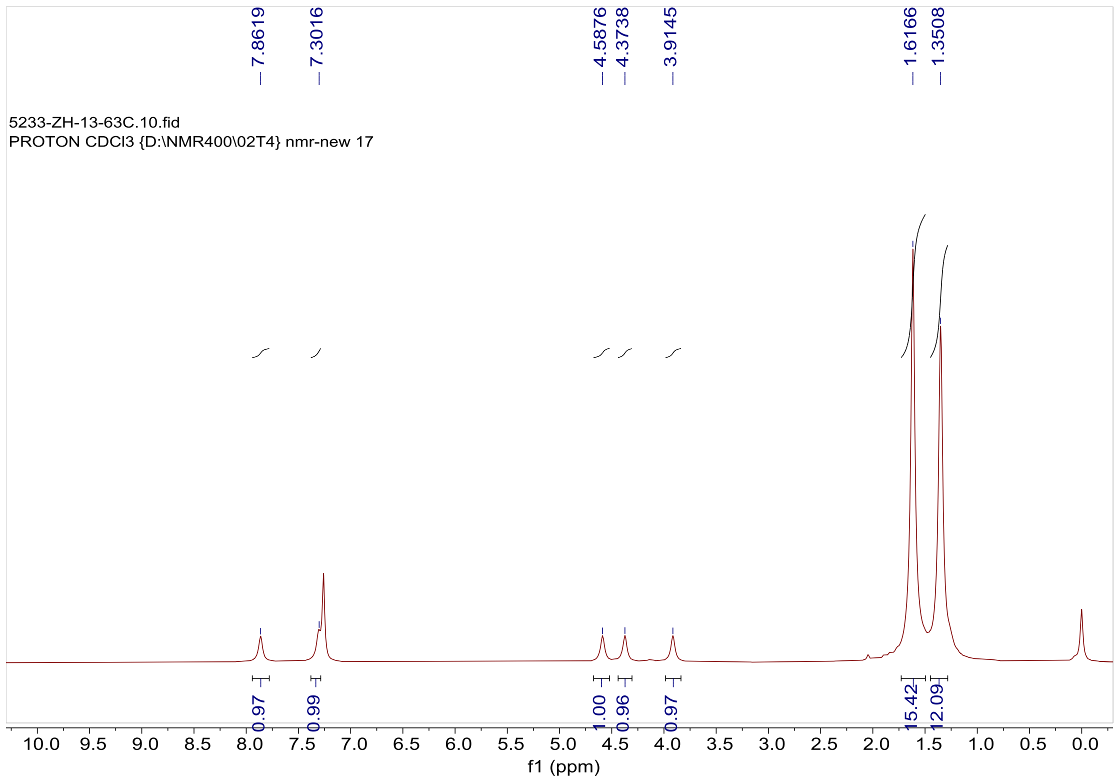

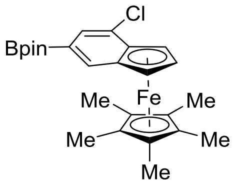

^{1}H NMR (400 MHz, $CDCl_3$)
^{13}C NMR (100 MHz, $CDCl_3$)

3c

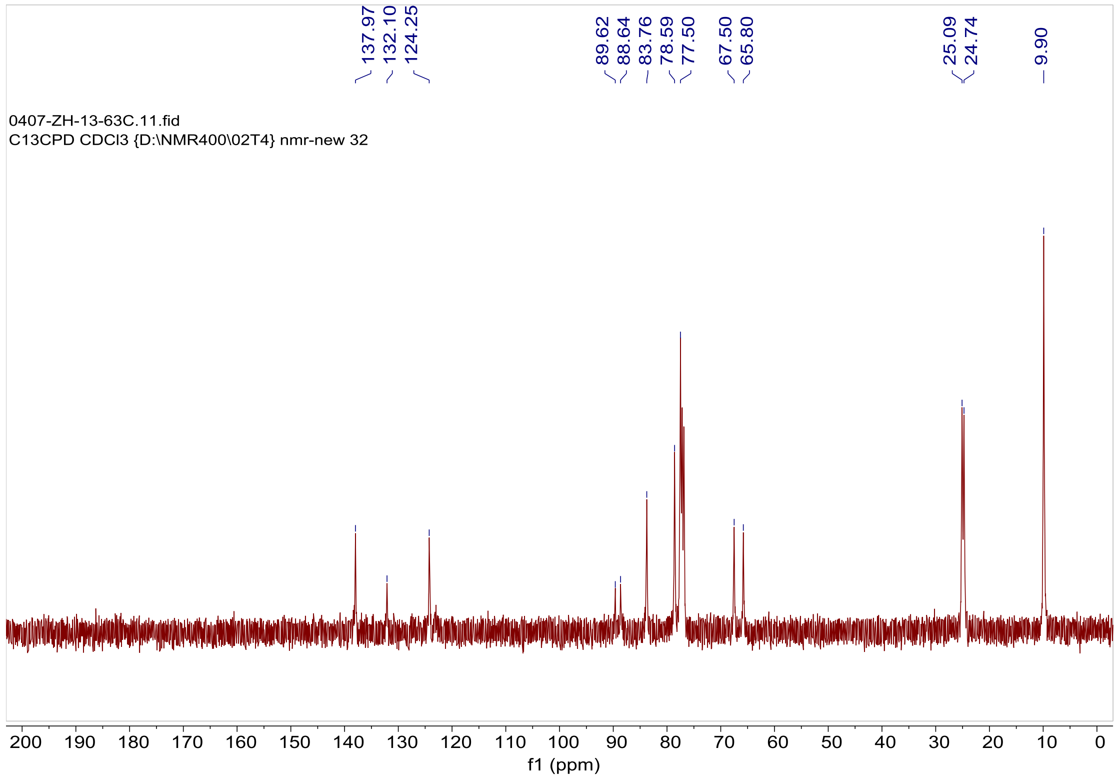

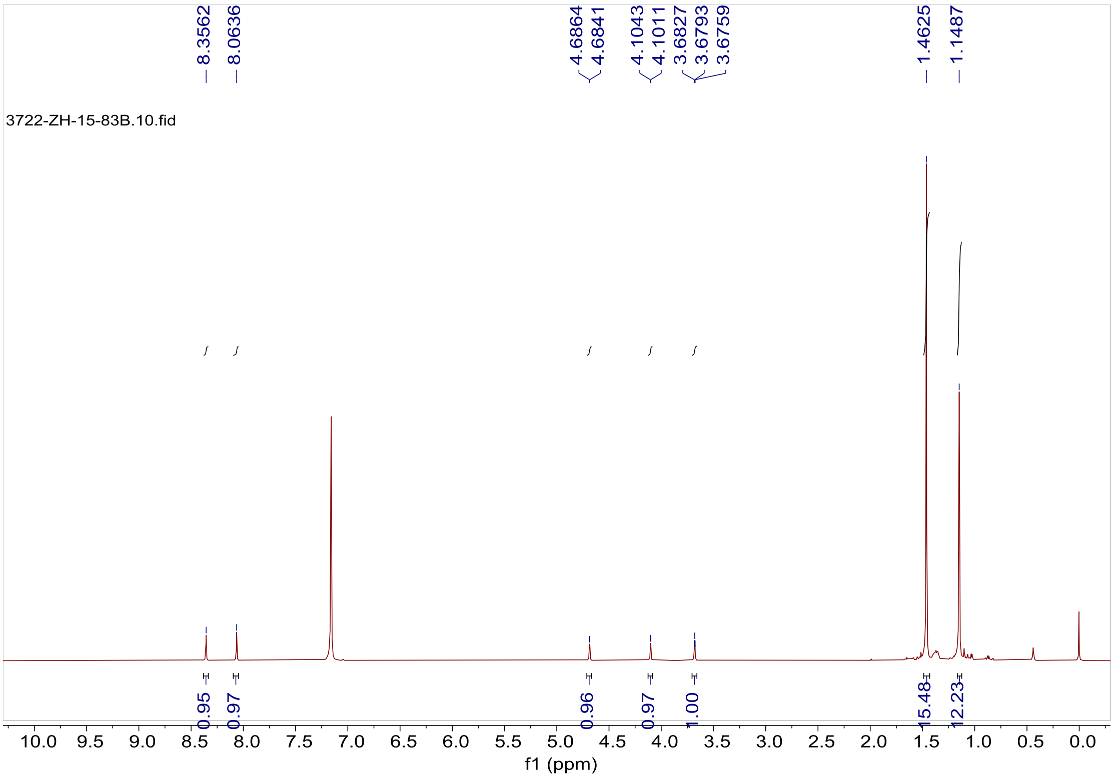

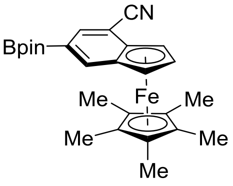

3d

^{1}H NMR (700 MHz, C_6D_6)

^{13}C NMR (175 MHz, C_6D_6)

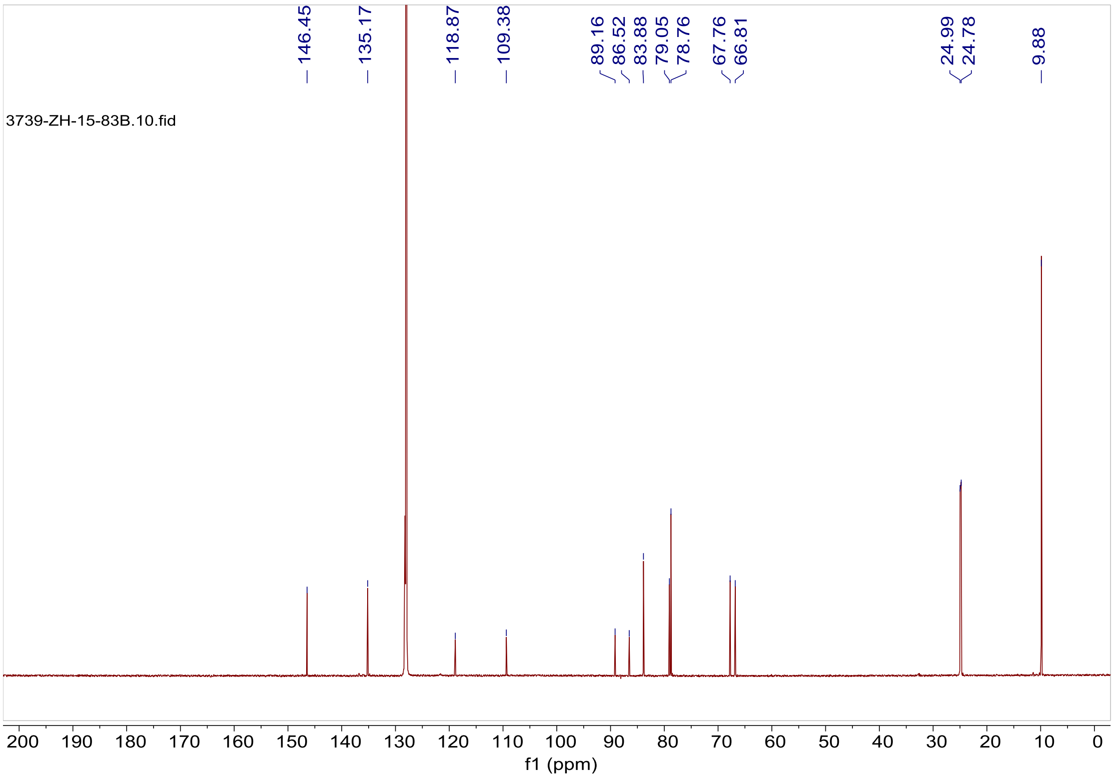

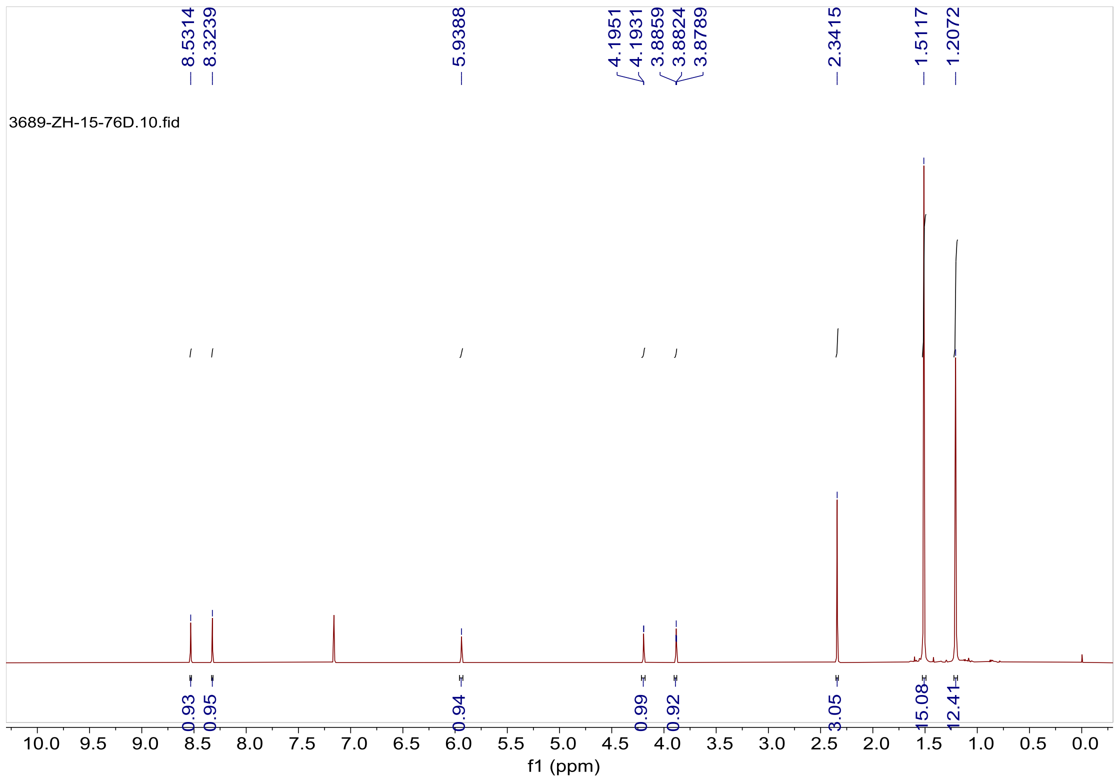

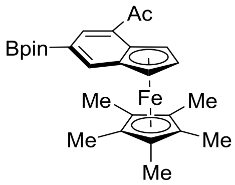

3e

^{1}H NMR (700 MHz, C_6D_6)
^{13}C NMR (175 MHz, C_6D_6)

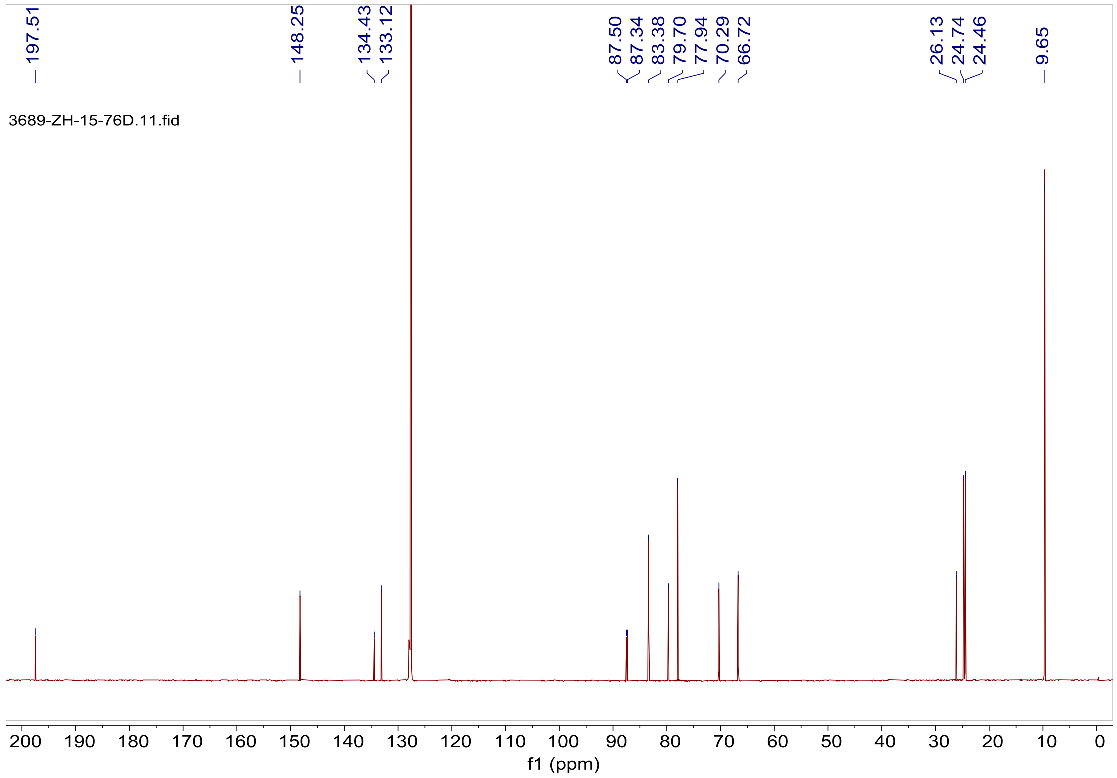

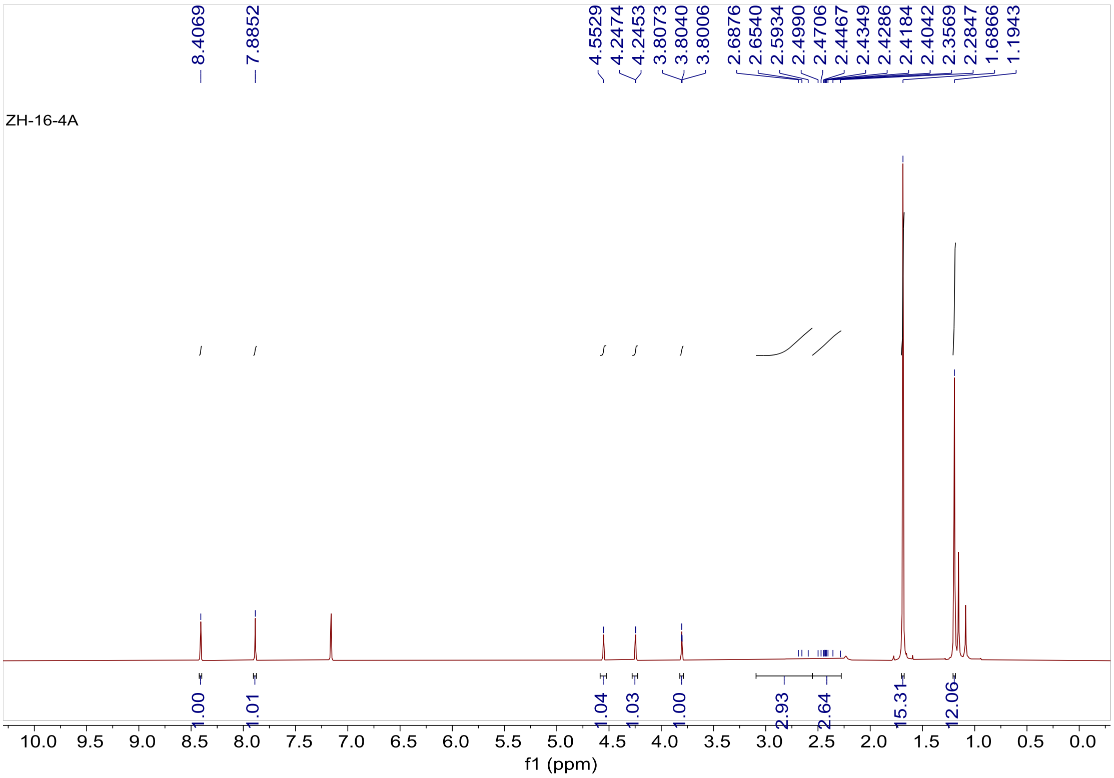

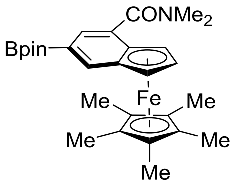

1f

^{1}H NMR (700 MHz, C_6D_6)

^{13}C NMR (175 MHz, C_6D_6)

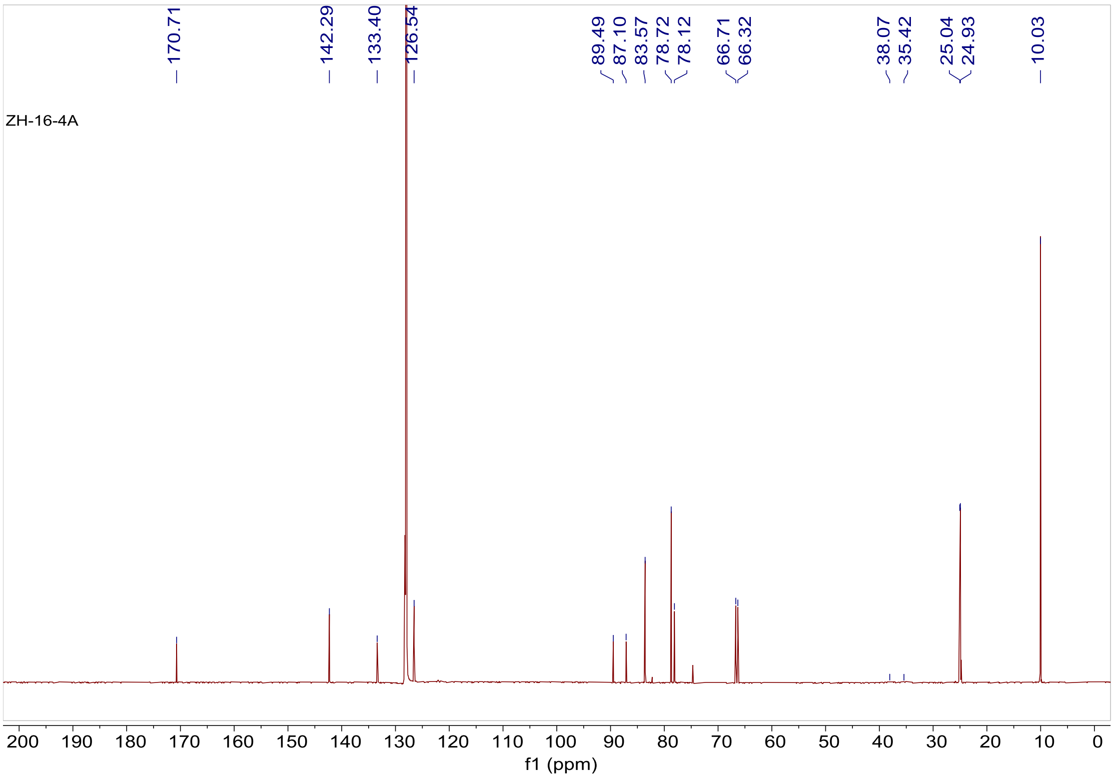

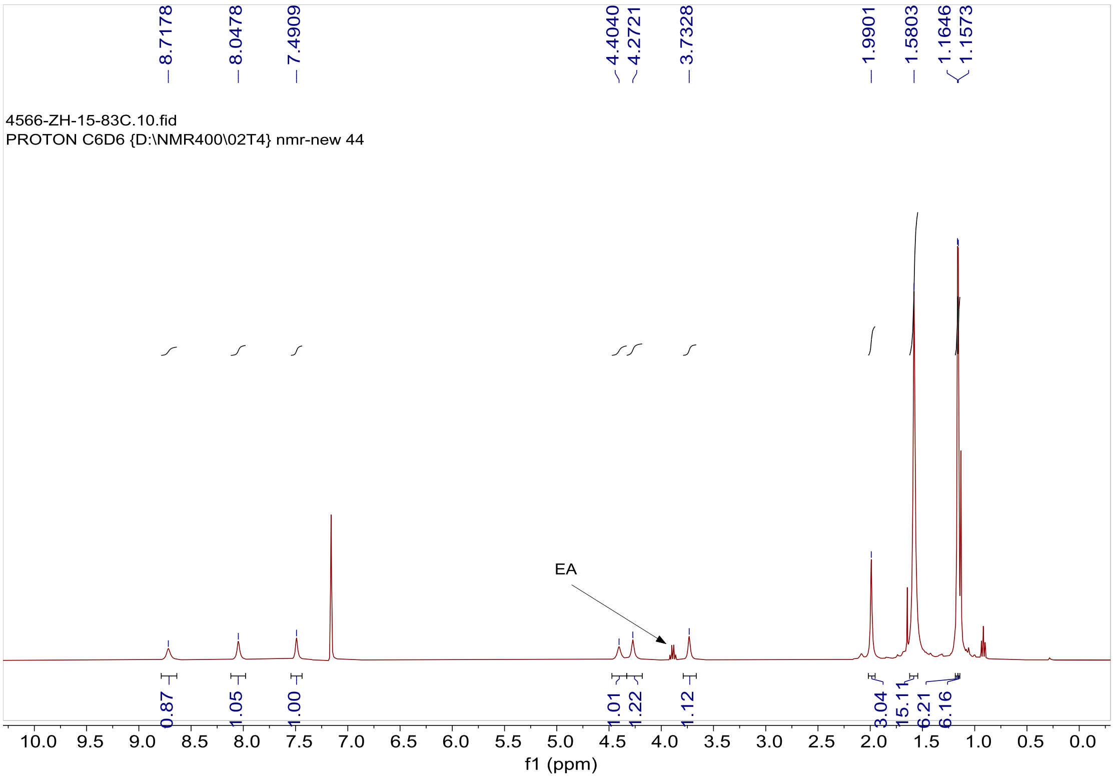

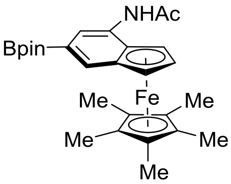

3g

^{1}H NMR (400 MHz, C_6D_6)

^{13}C NMR (100 MHz, $CDCl_3$)

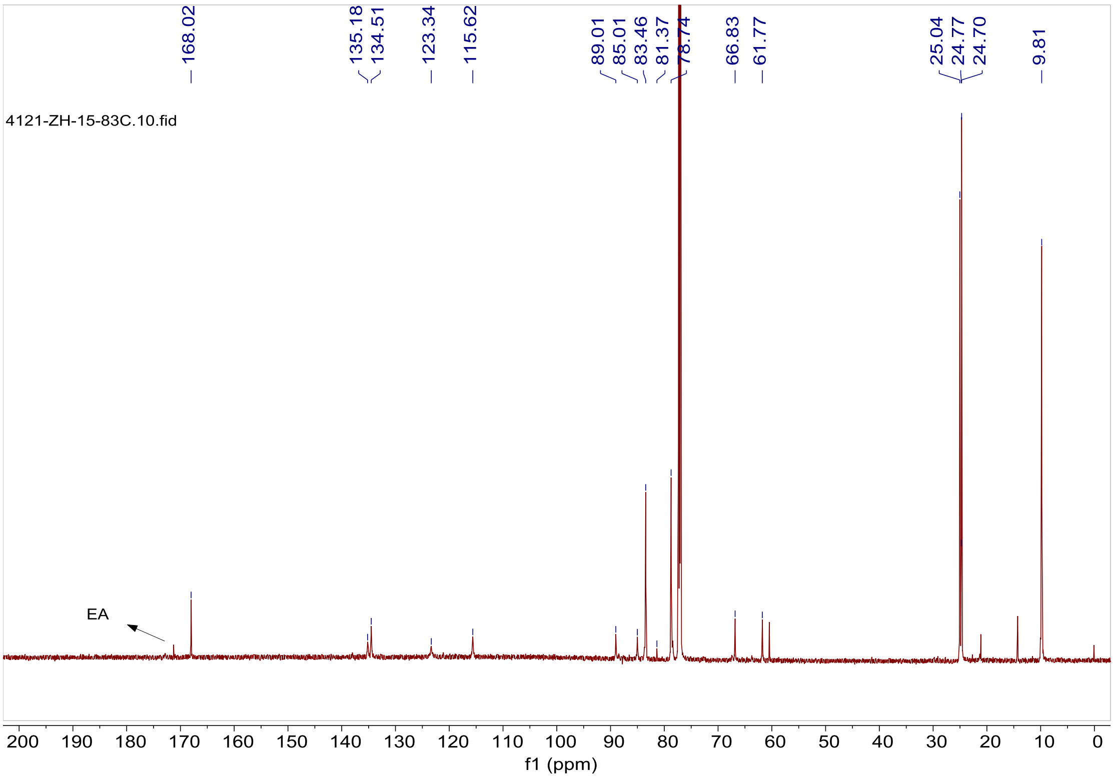

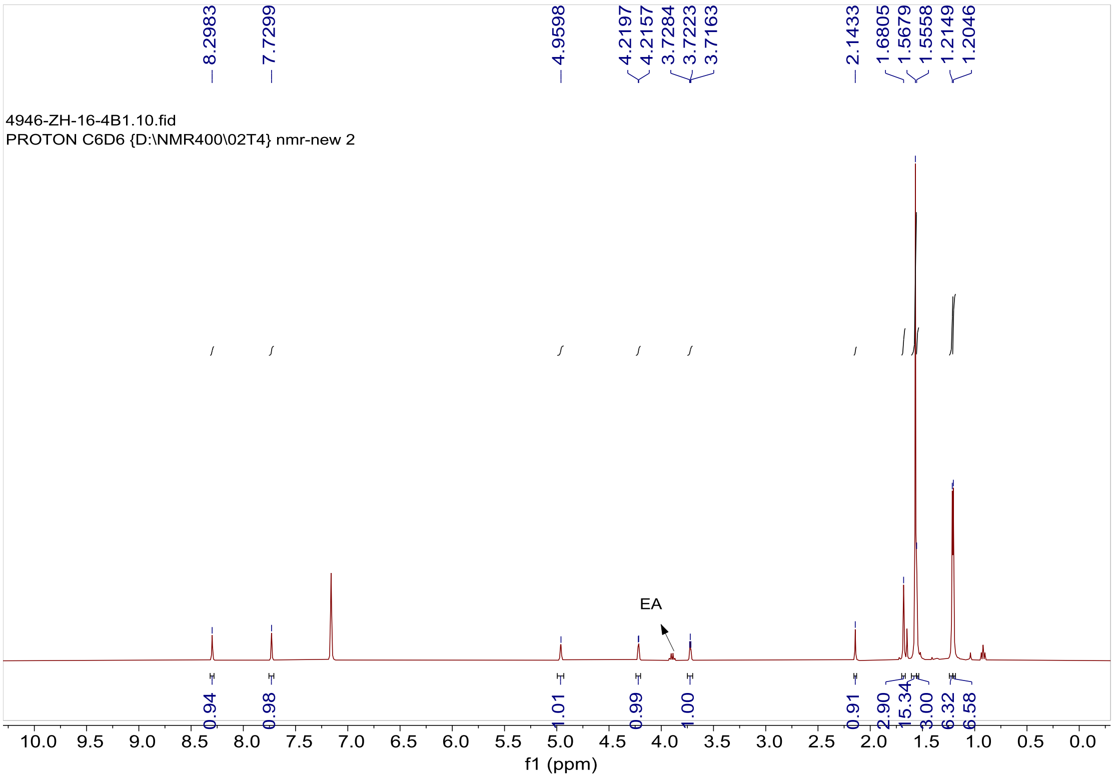

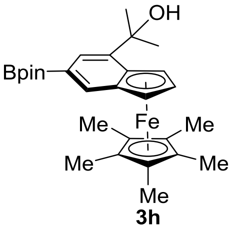

3h

^{1}H NMR (400 MHz, C_6D_6)
^{13}C NMR (100 MHz, C_6D_6)

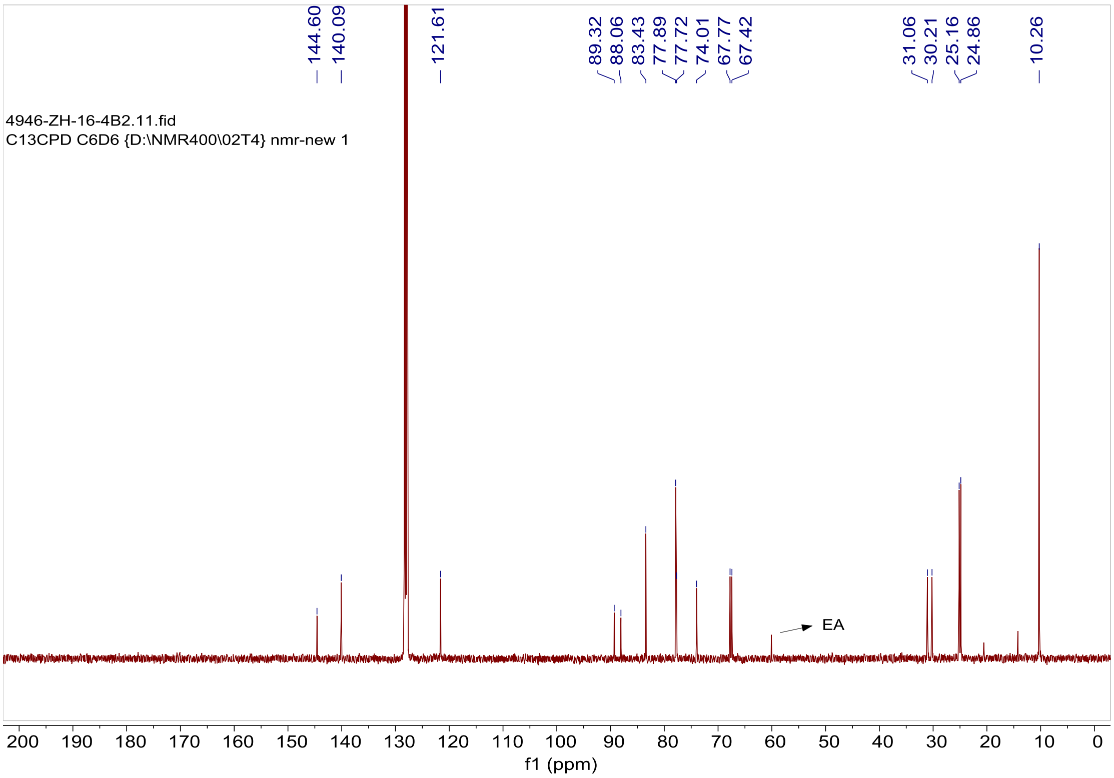

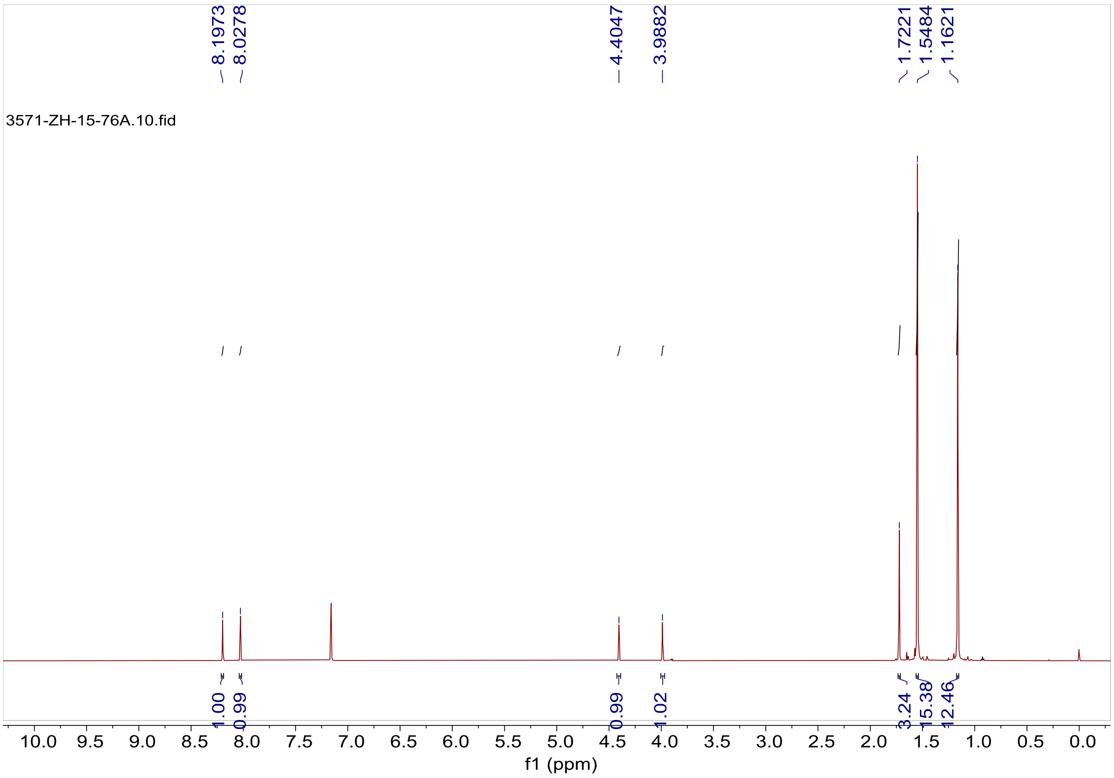

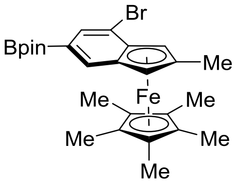

3i

^{1}H NMR (700 MHz, C_6D_6)
^{13}C NMR (175 MHz, C_6D_6)

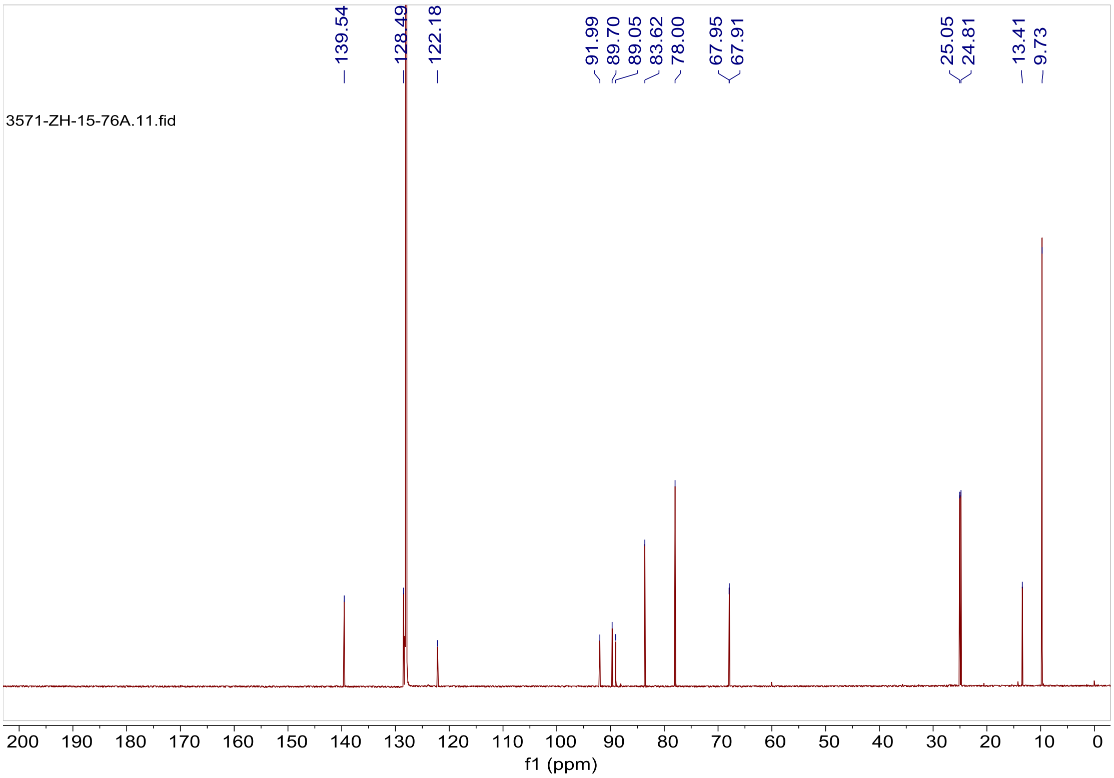

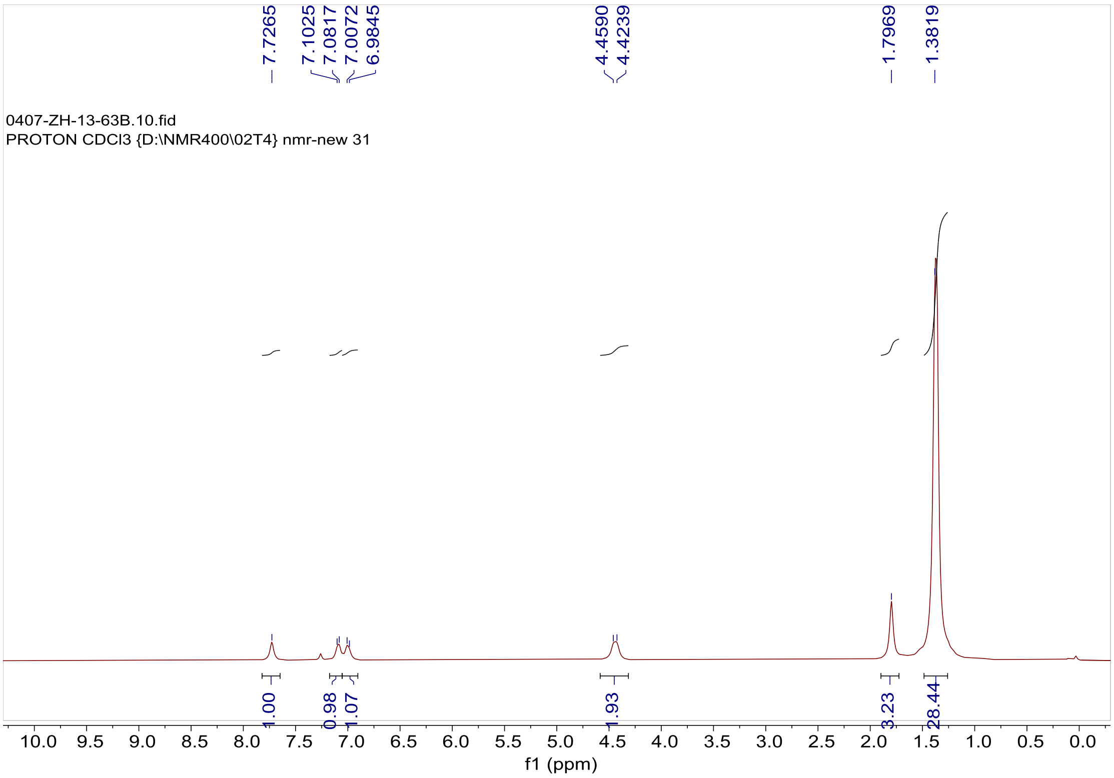

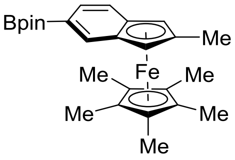

3j

^{1}H NMR (400 MHz, $CDCl_3$)
^{13}C NMR (100 MHz, $CDCl_3$)

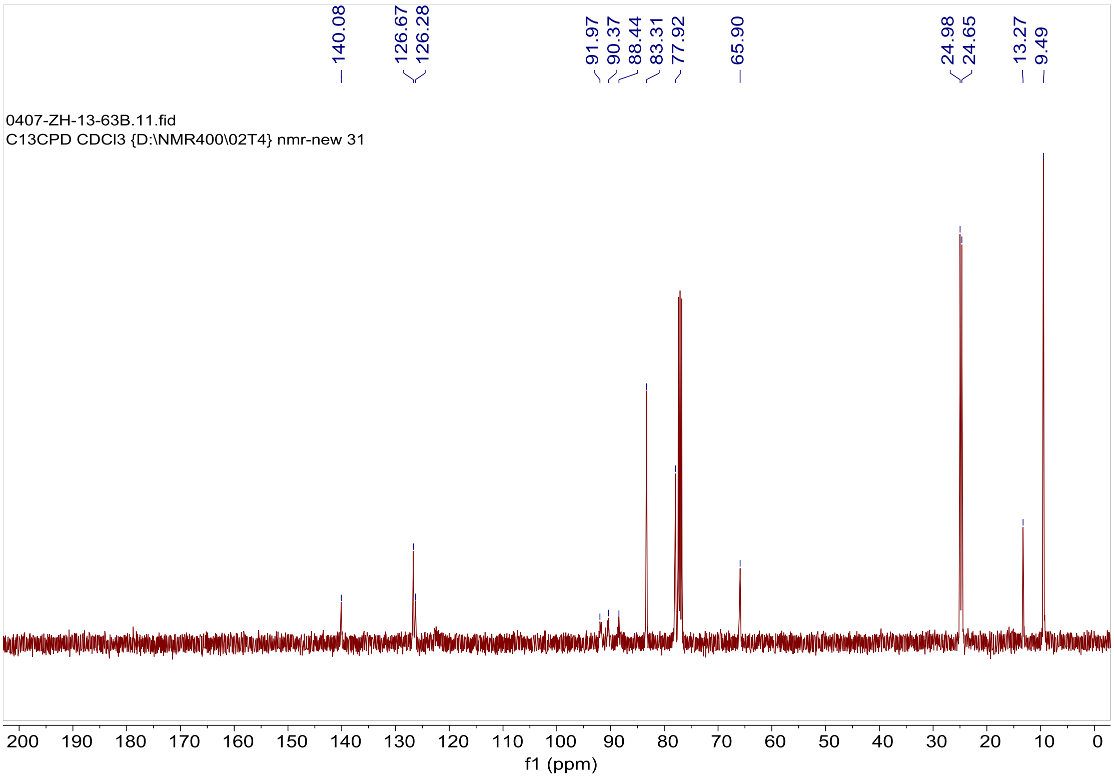

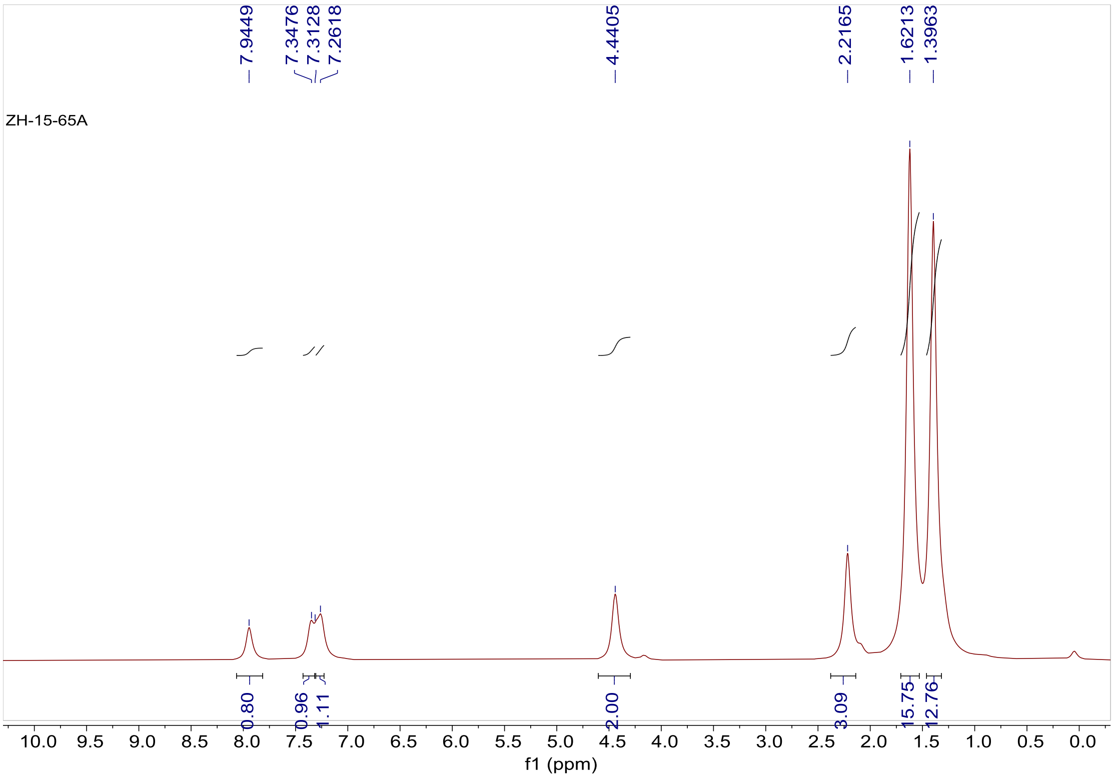

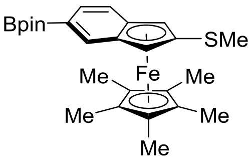

3k

^{1}H NMR (700 MHz, $CDCl_3$)
^{13}C NMR (175 MHz, $CDCl_3$)

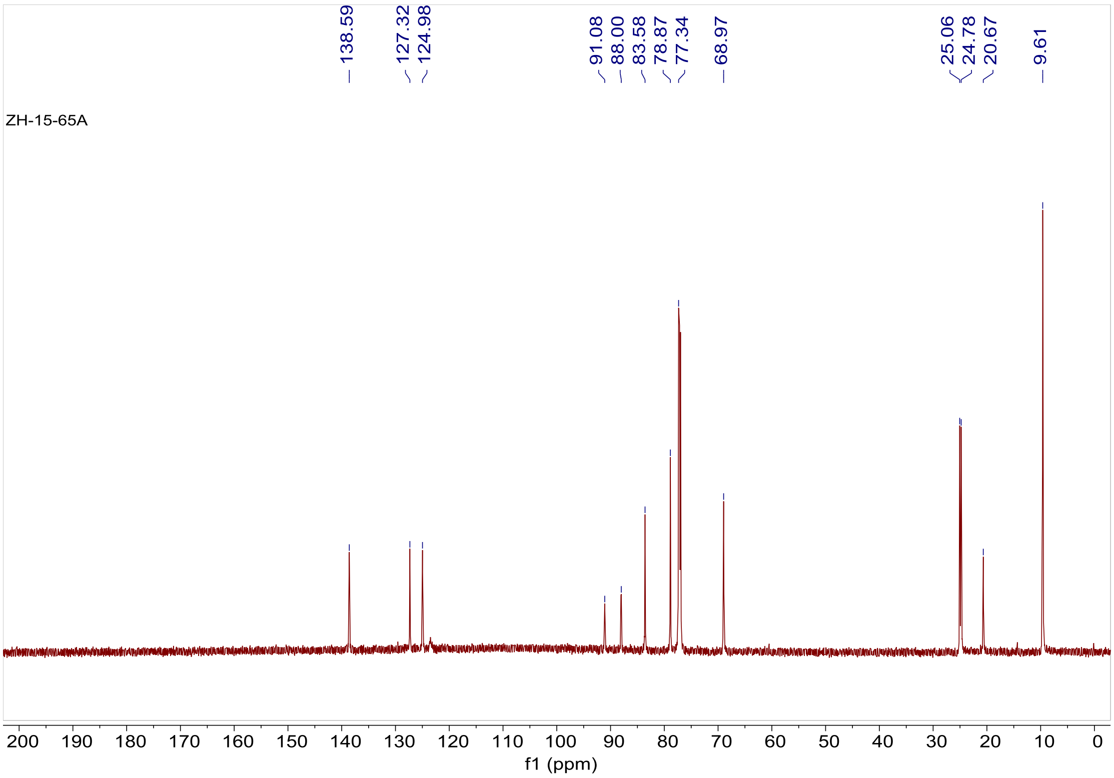

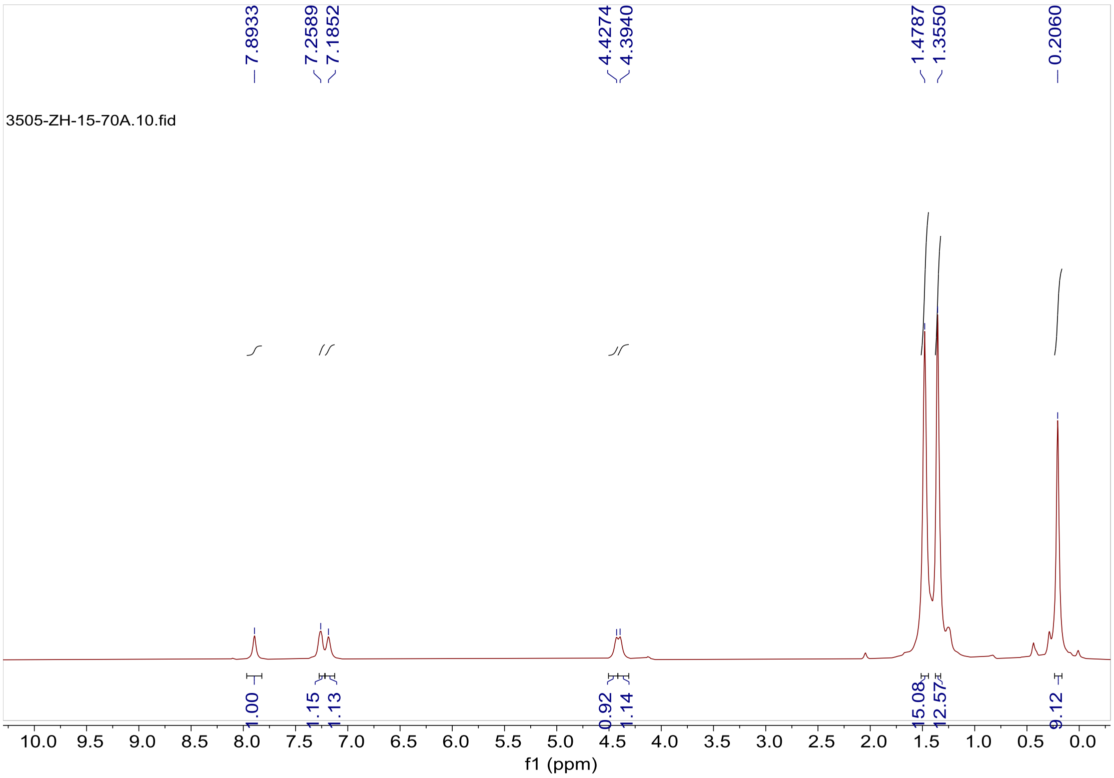

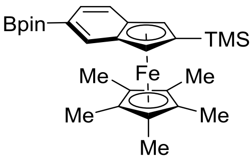

3l

^{1}H NMR (700 MHz, $CDCl_3$)
^{13}C NMR (175 MHz, $CDCl_3$)

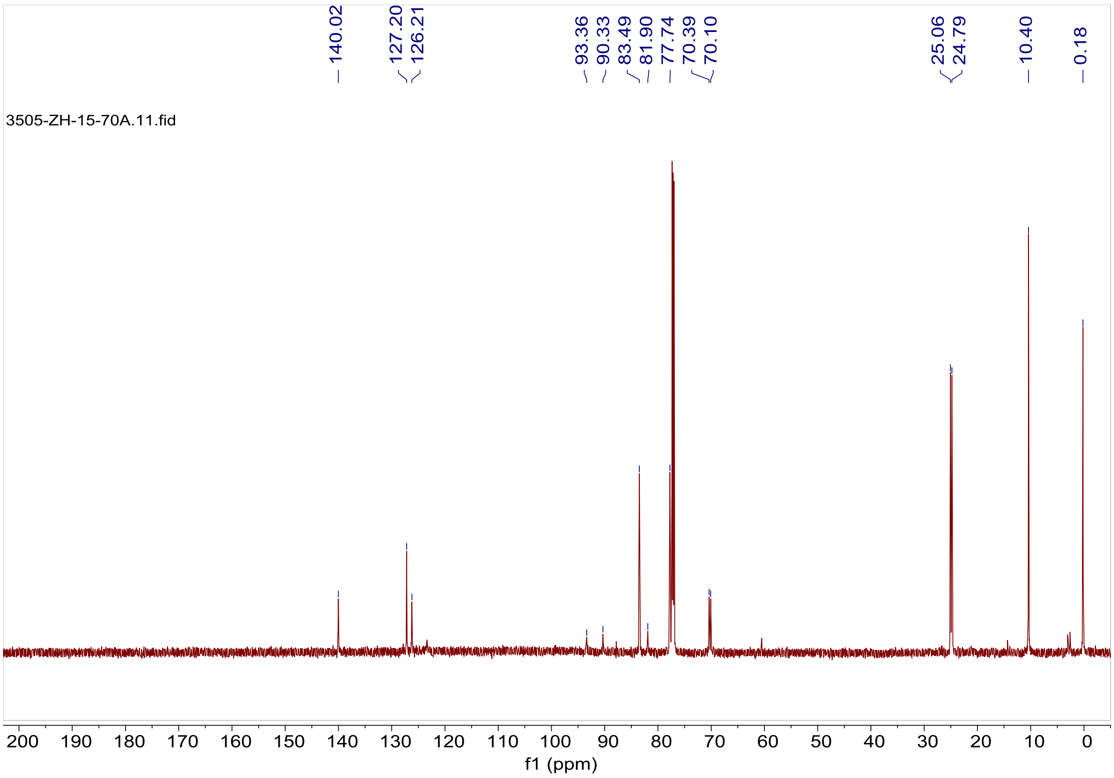

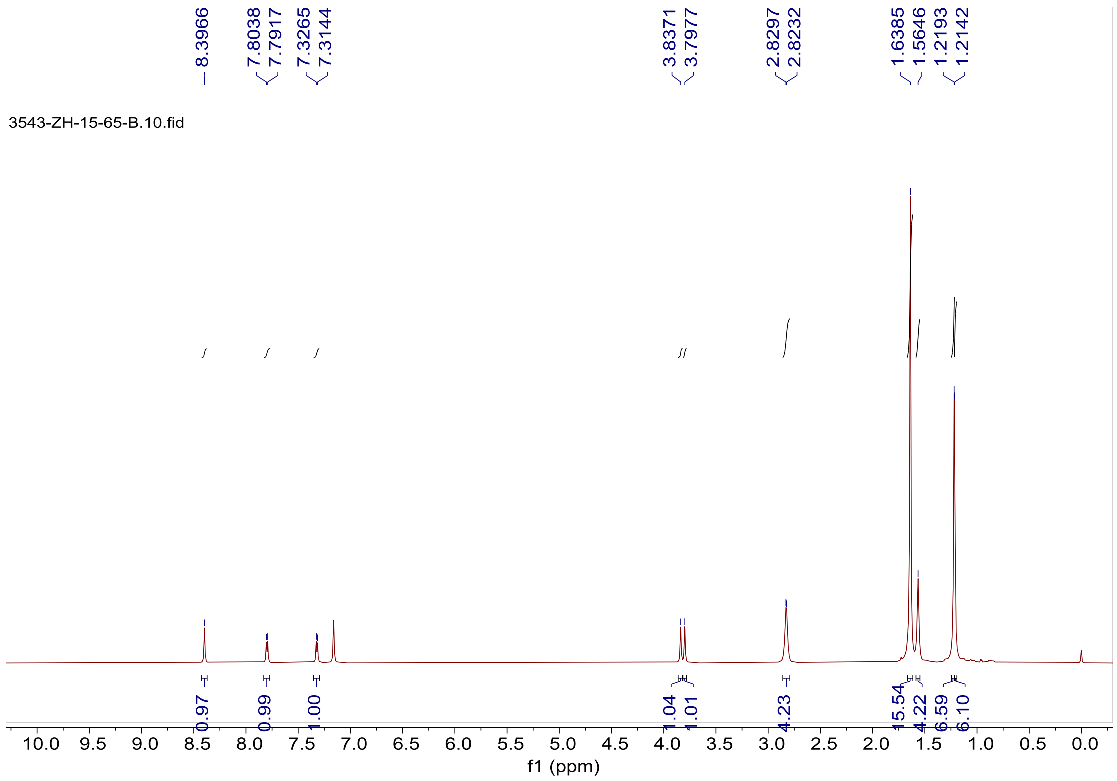

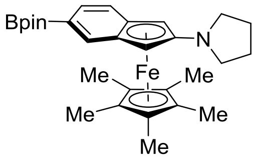

3m

^{1}H NMR (700 MHz, C_6D_6)

^{13}C NMR (175 MHz, C_6D_6)

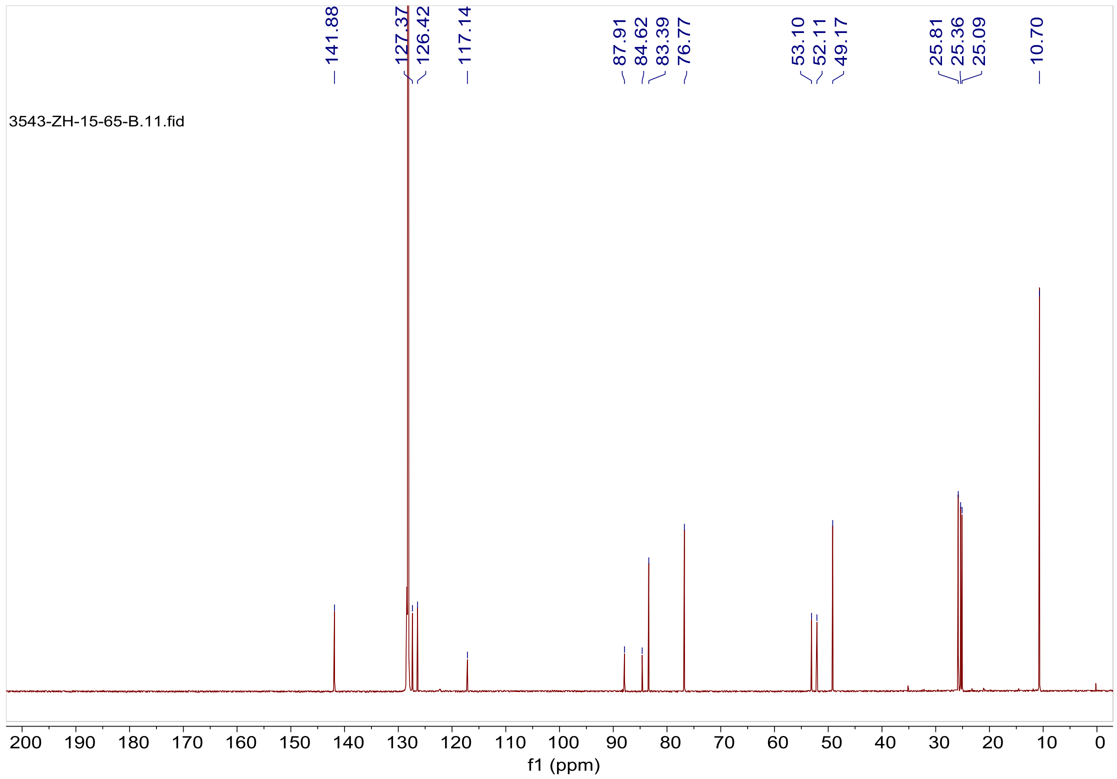

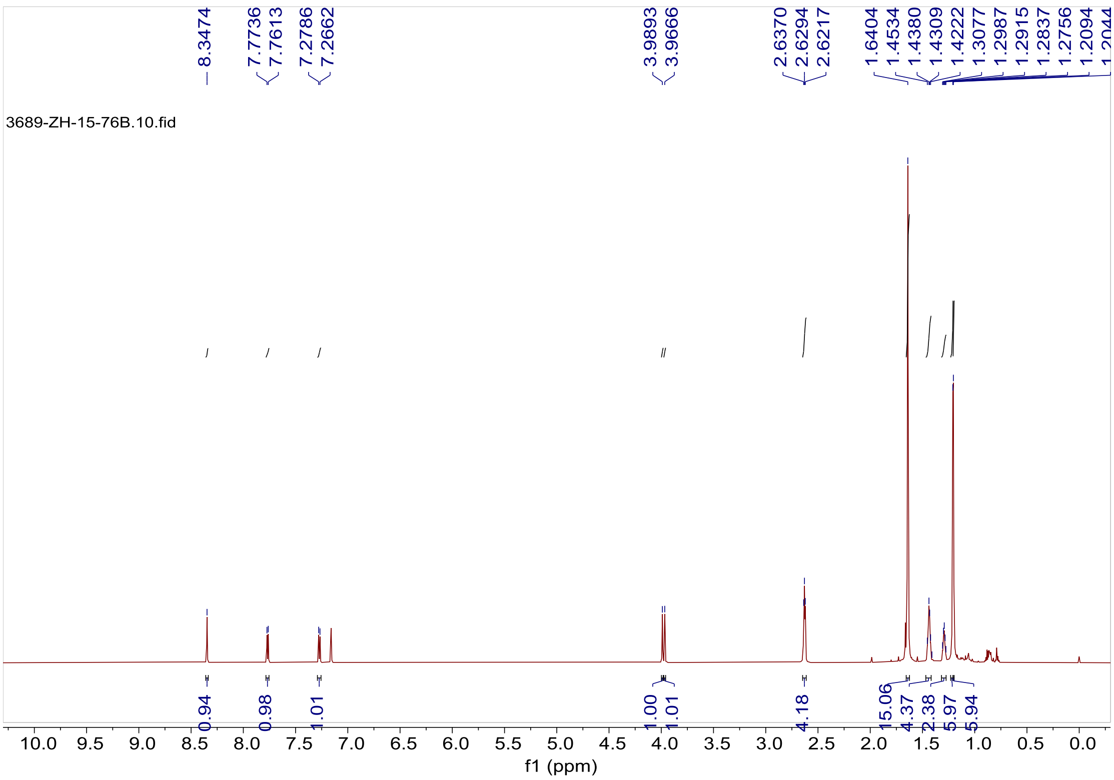

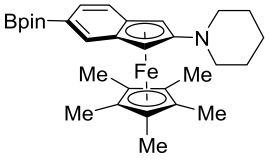

3n

^{1}H NMR (700 MHz, C_6D_6)

^{13}C NMR (175 MHz, C_6D_6)

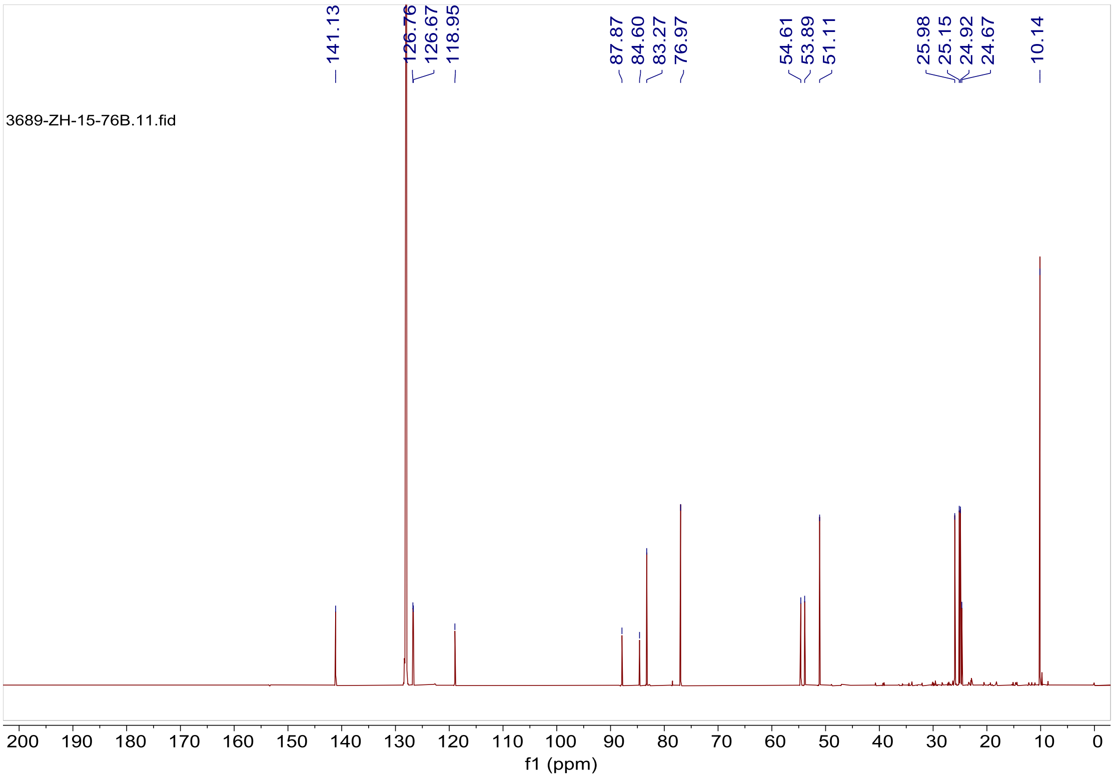

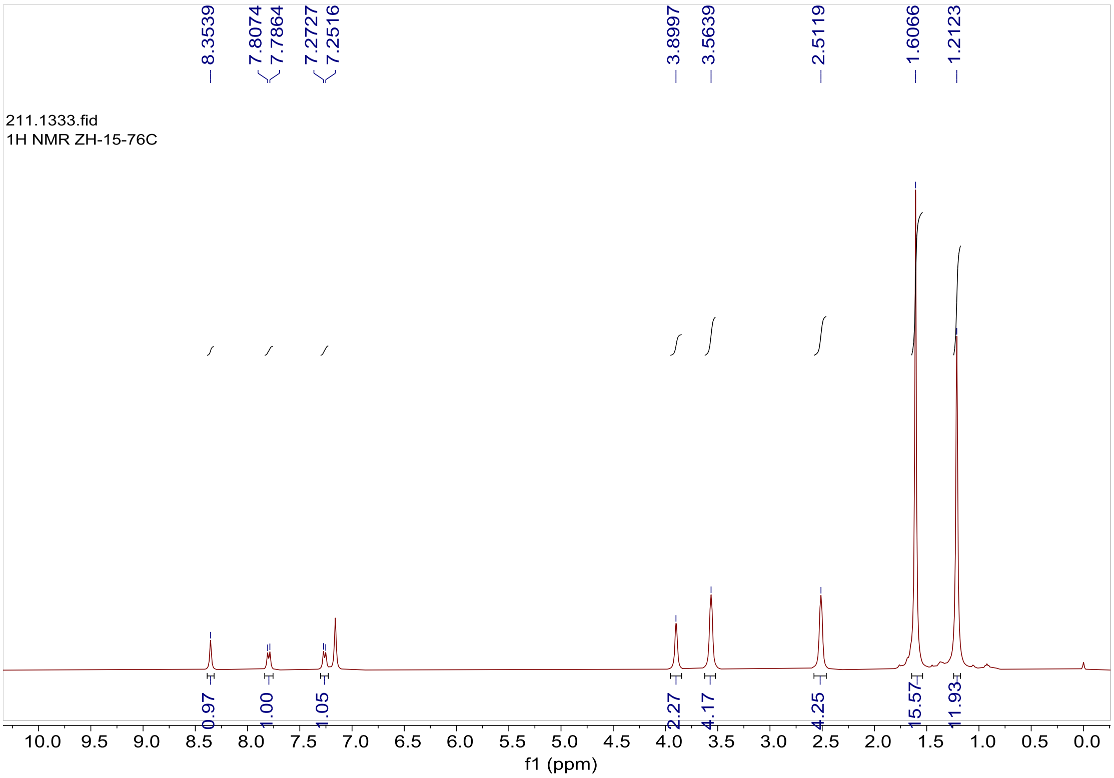

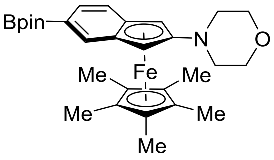

3o

^{1}H NMR (700 MHz, C_6D_6)

^{13}C NMR (175 MHz, C_6D_6)

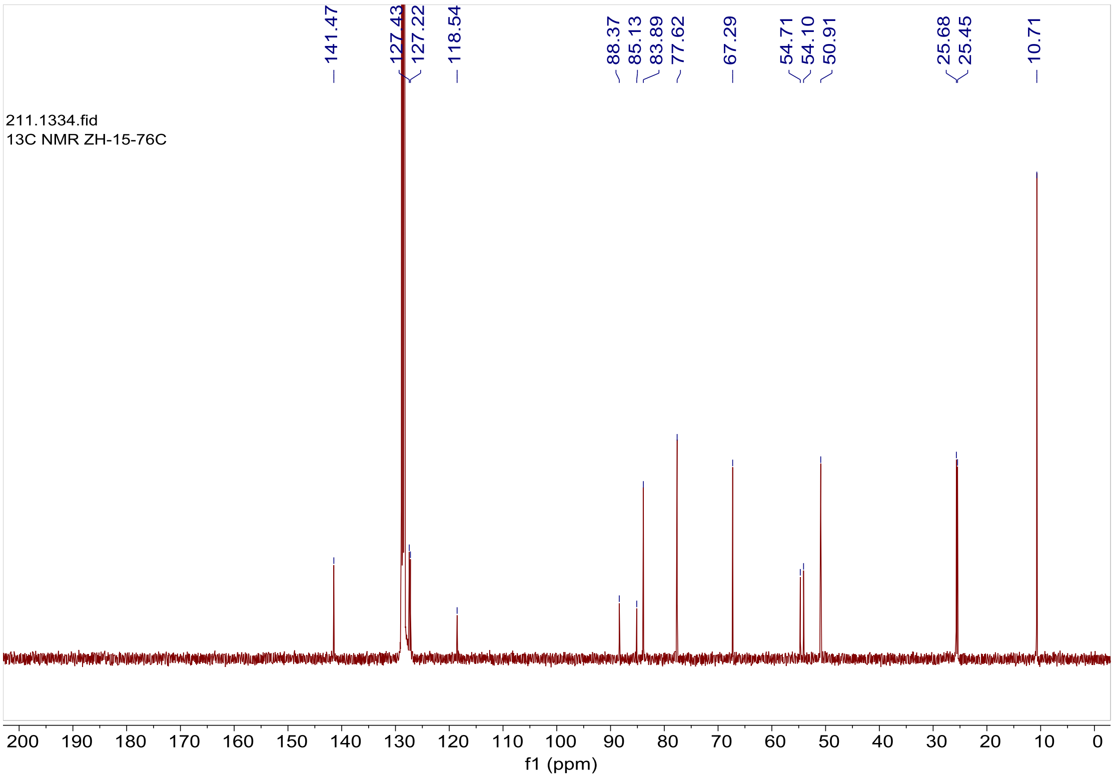

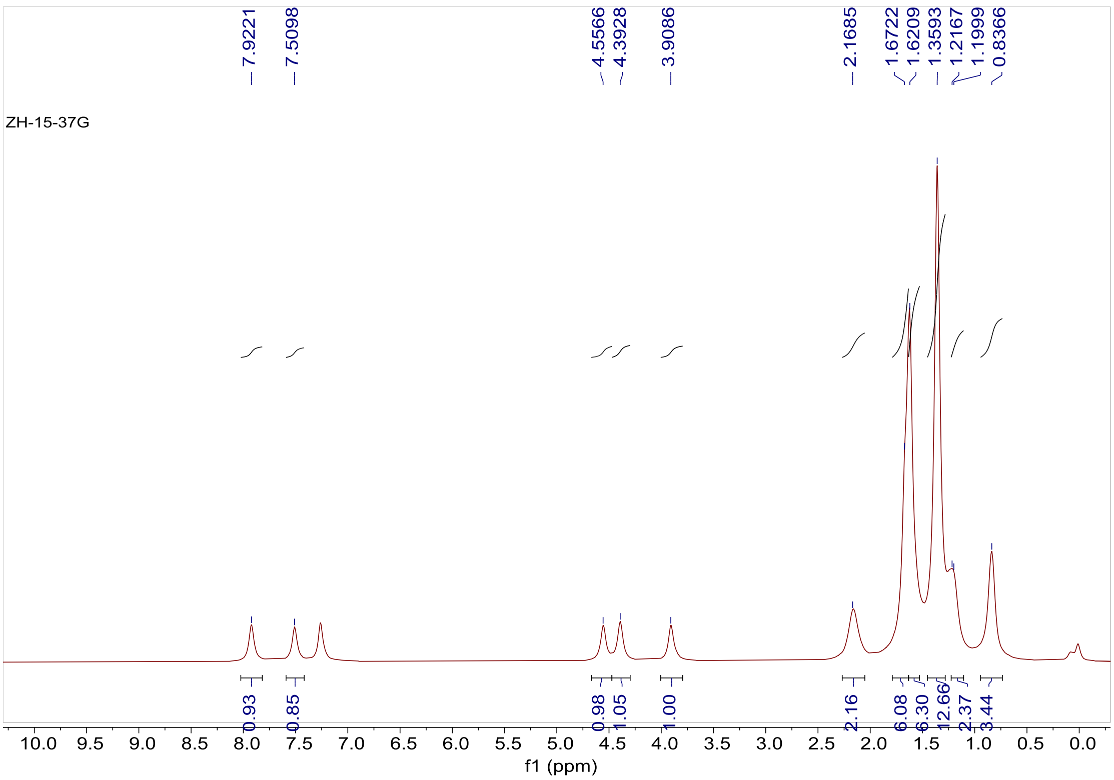

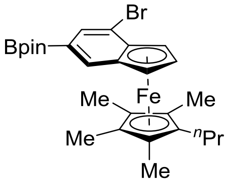

3p

^{1}H NMR (400 MHz, $CDCl_3$)

^{13}C NMR (100 MHz, $CDCl_3$)

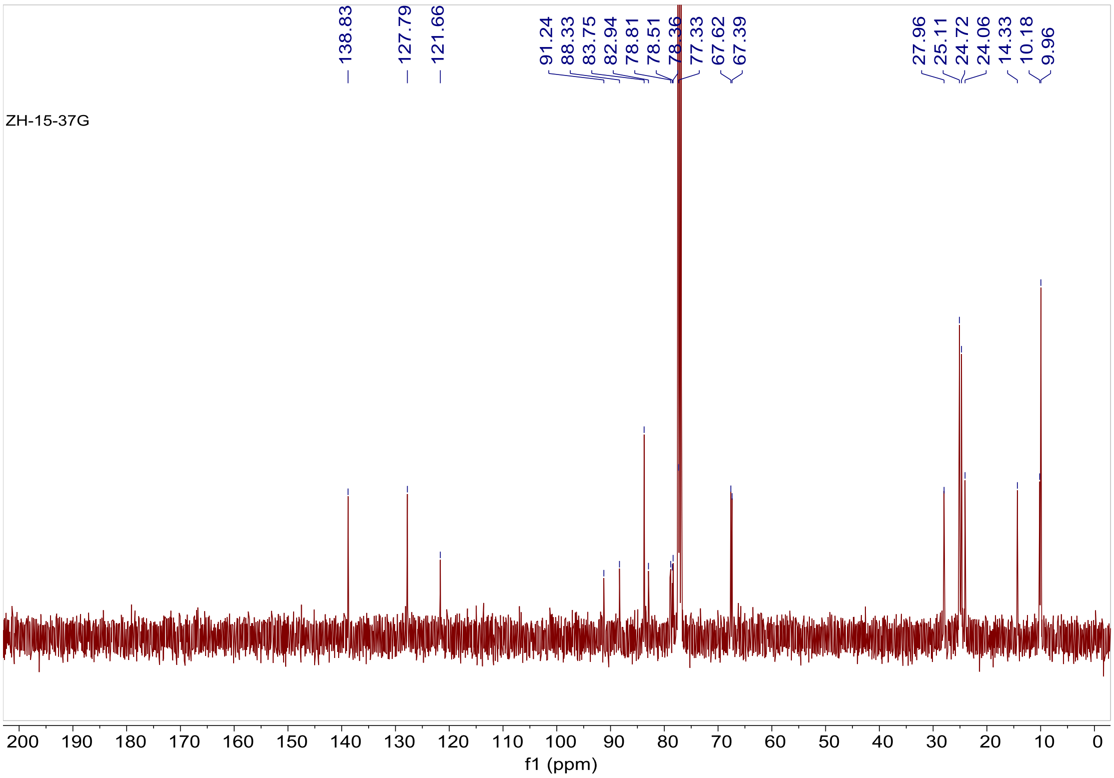

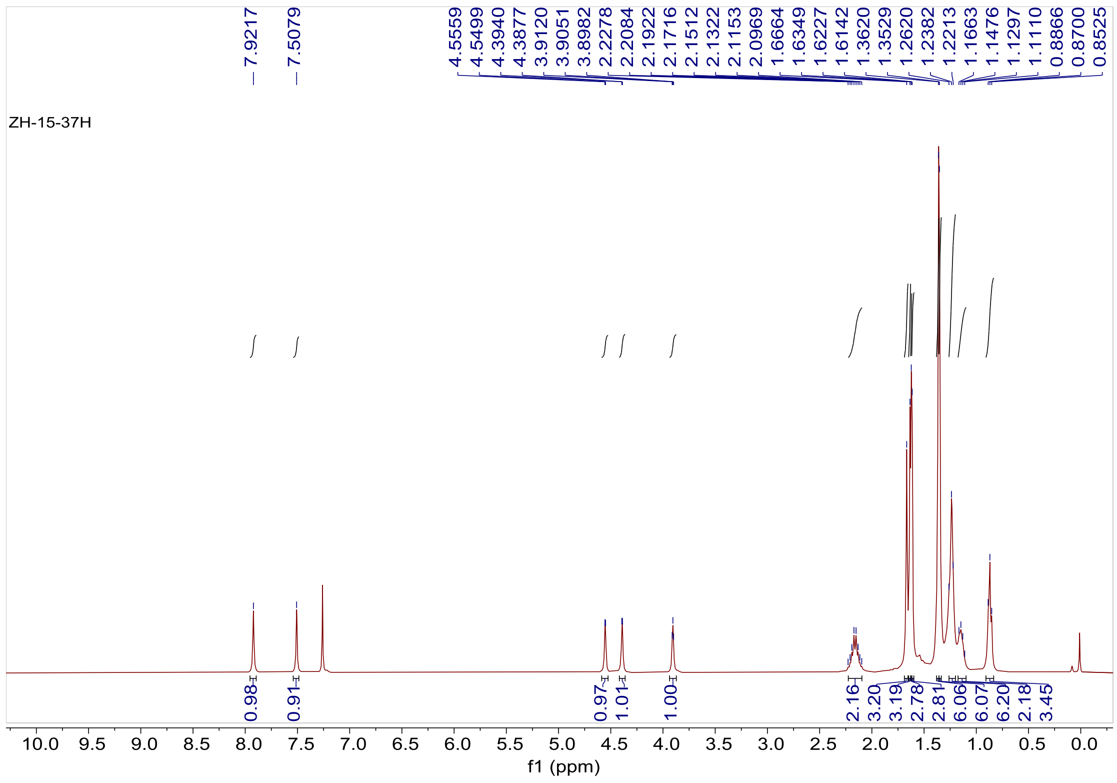

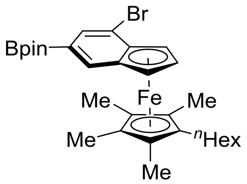

3q

^{1}H NMR (400 MHz, $CDCl_3$)

^{13}C NMR (100 MHz, $CDCl_3$)

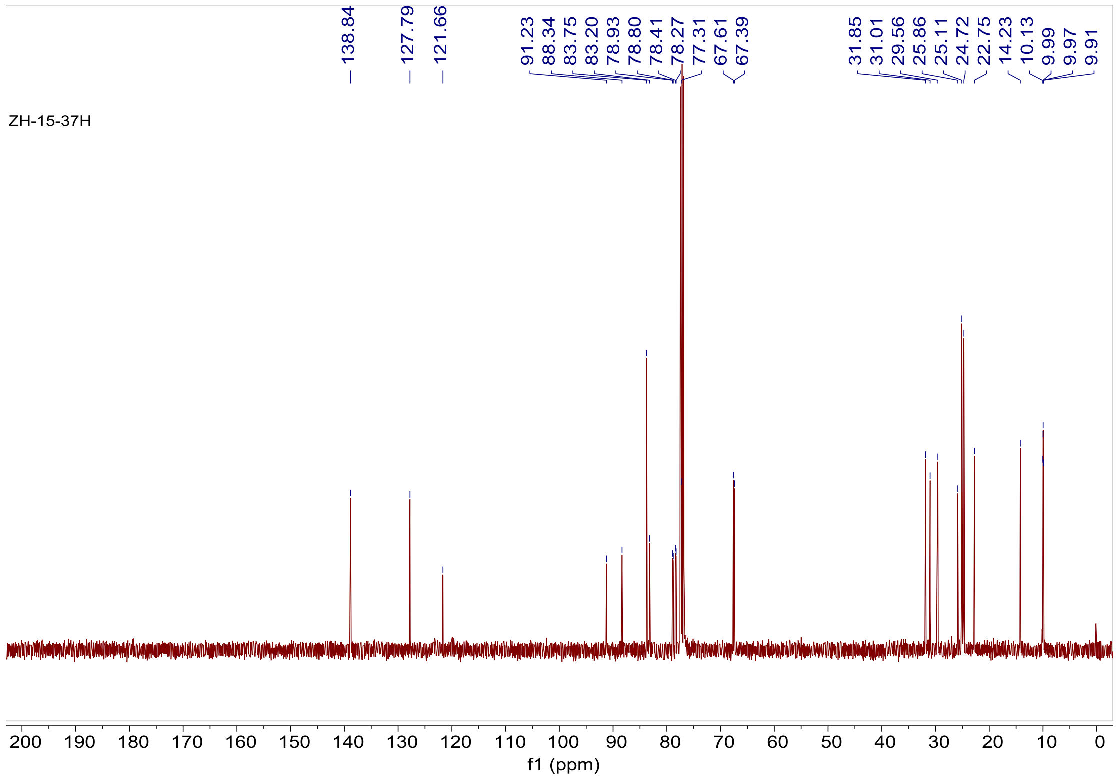

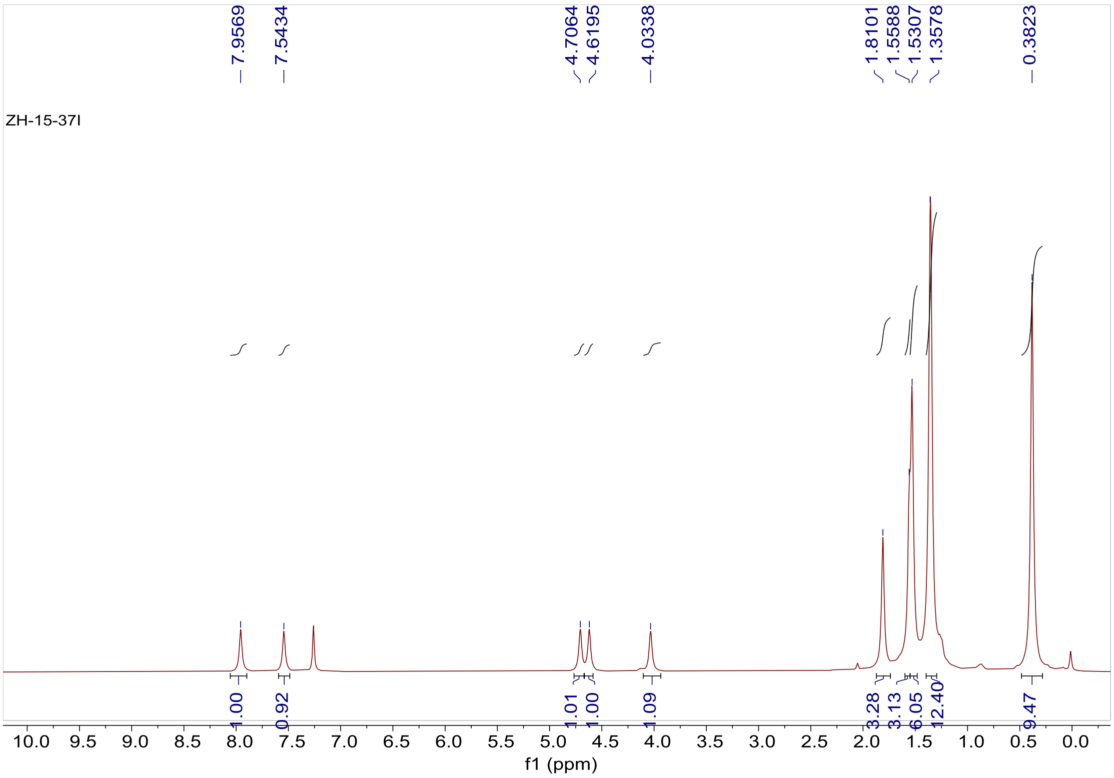

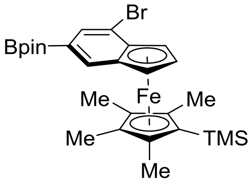

3r

^{1}H NMR (400 MHz, $CDCl_3$)
^{13}C NMR (100 MHz, $CDCl_3$)

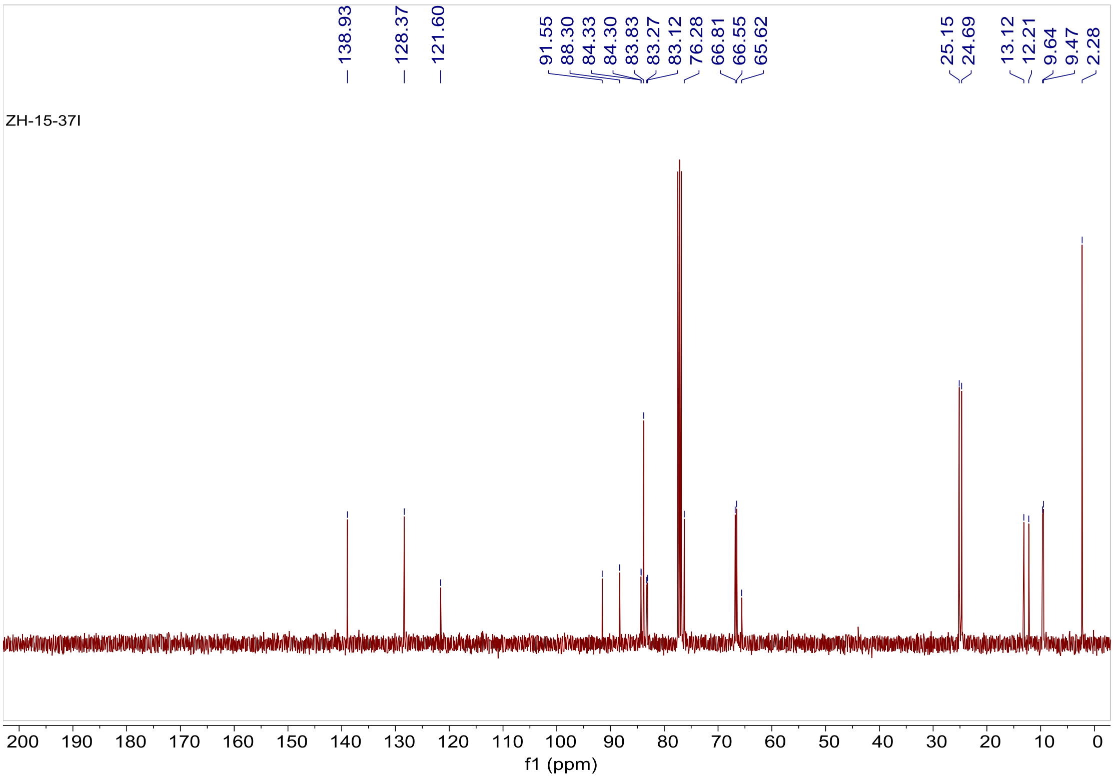

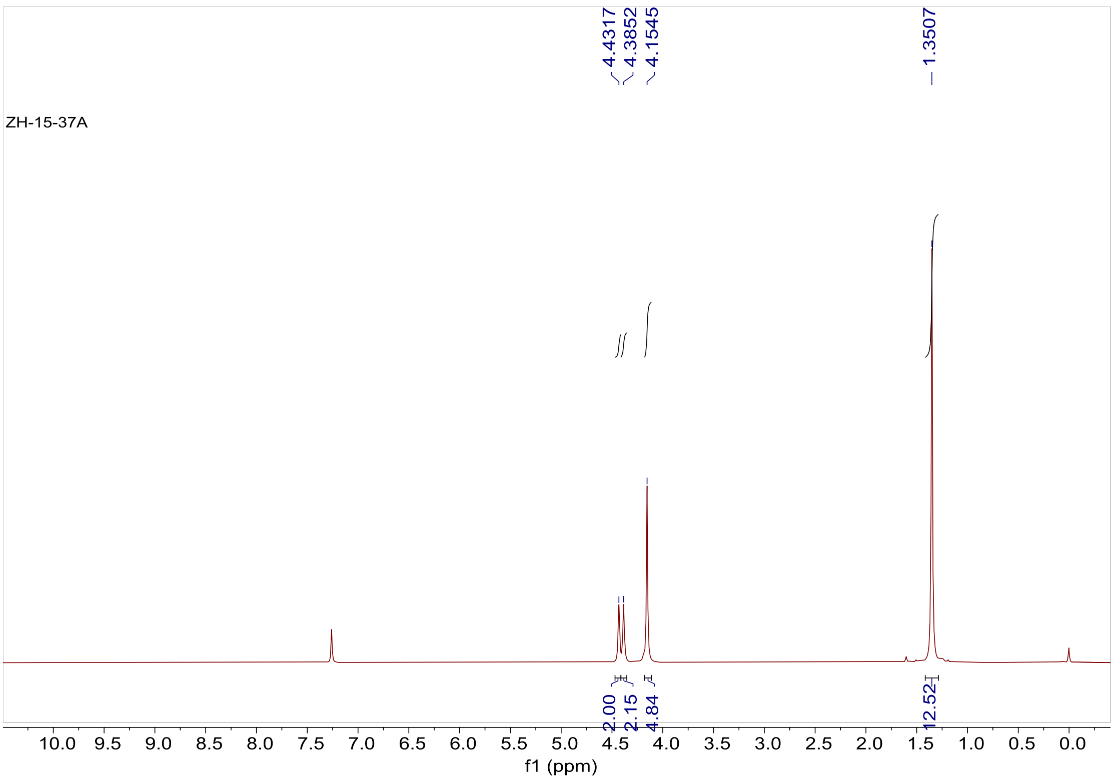

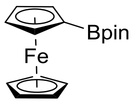

6a

^{1}H NMR (400 MHz, $CDCl_3$)
^{13}C NMR (100 MHz, $CDCl_3$)

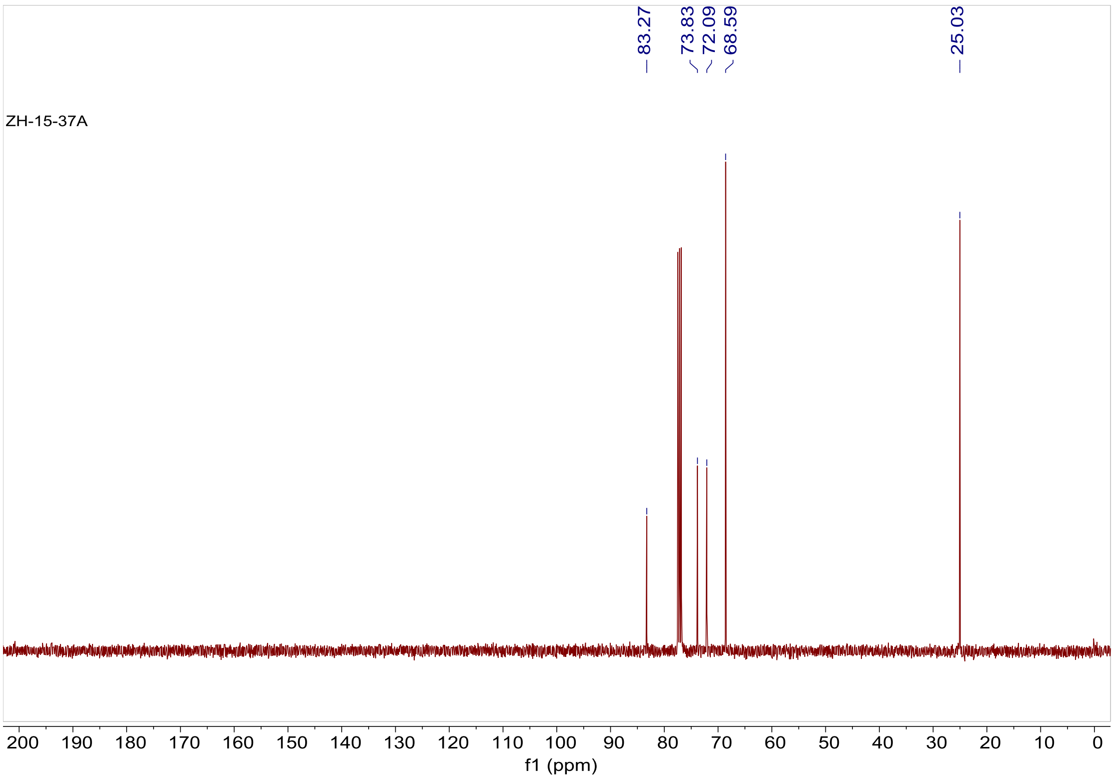

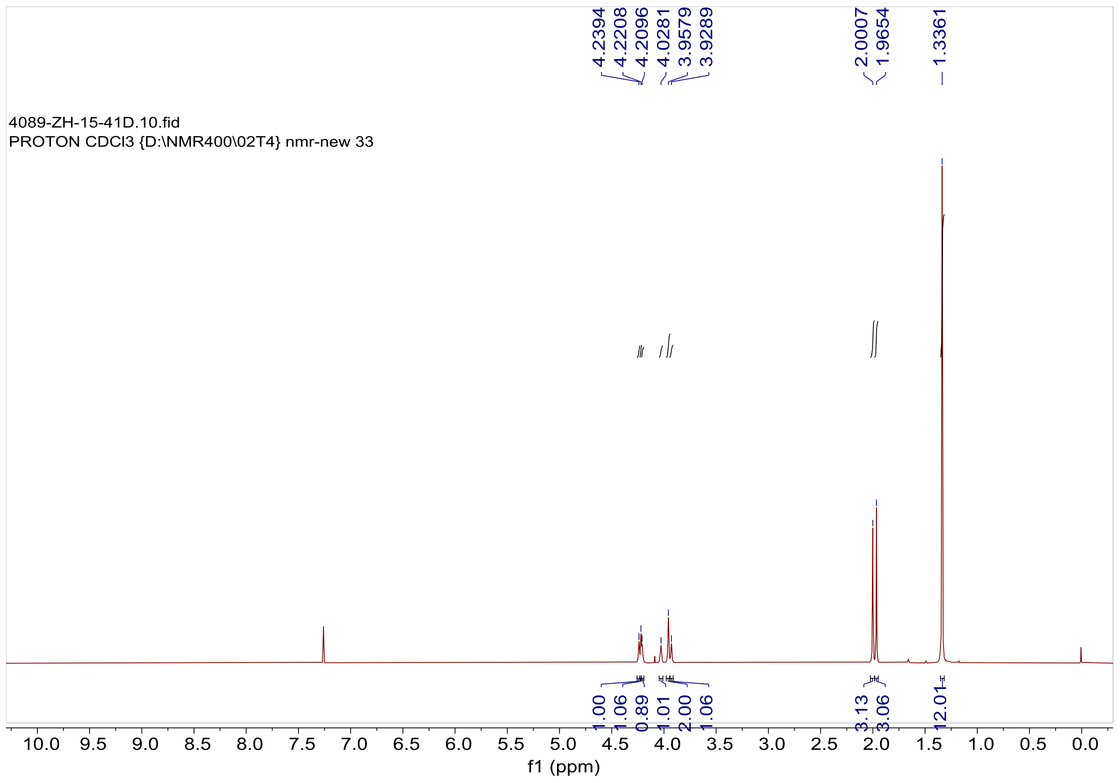

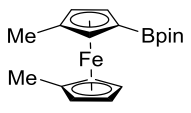

6b

^{1}H NMR (400 MHz, $CDCl_3$)

^{13}C NMR (100 MHz, $CDCl_3$)

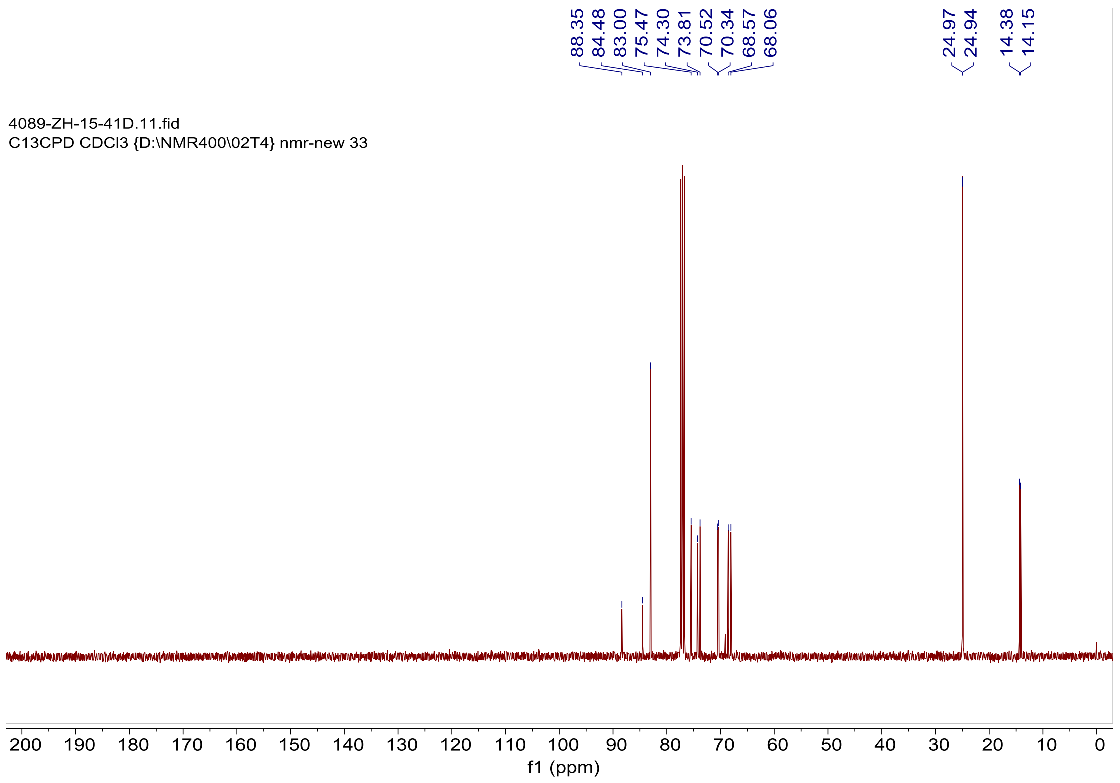

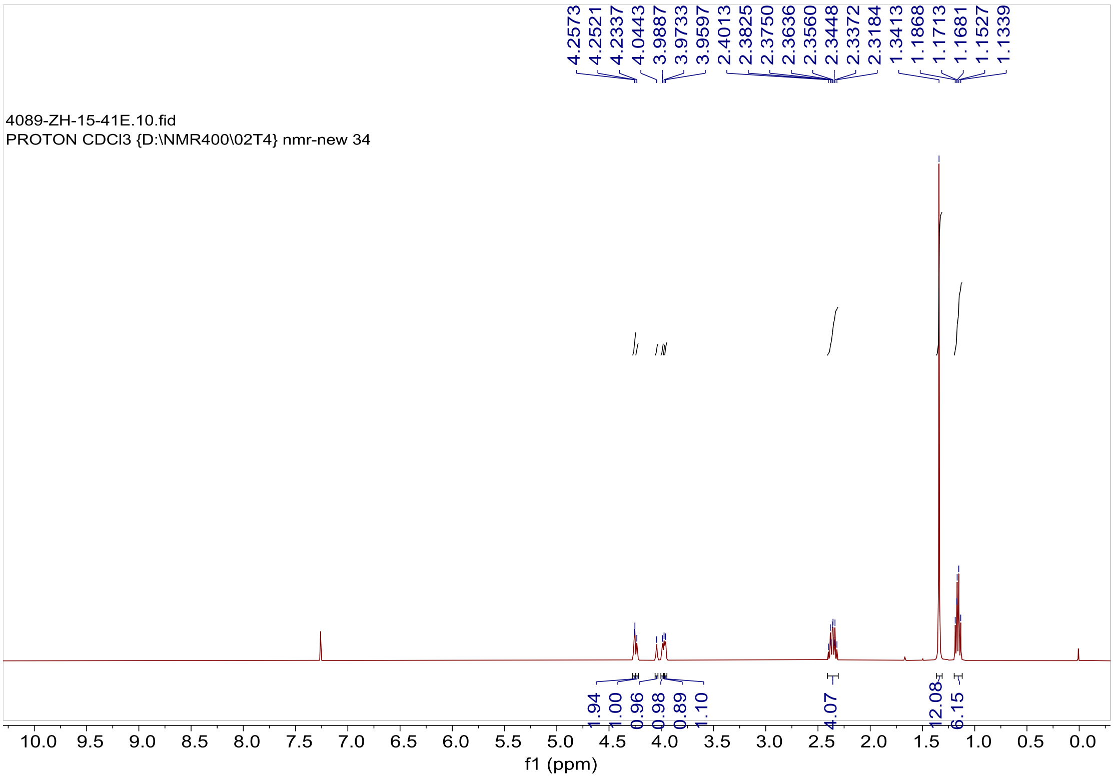

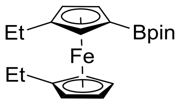

6c

^{1}H NMR (400 MHz, $CDCl_3$)
^{13}C NMR (100 MHz, $CDCl_3$)

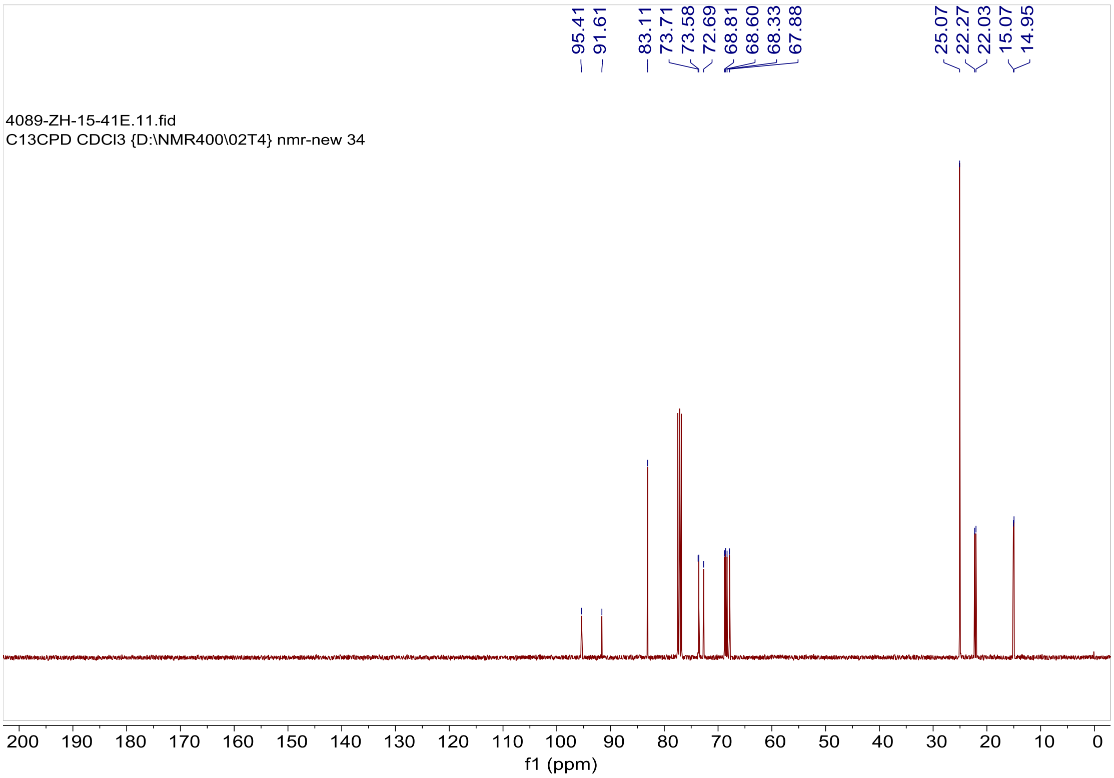

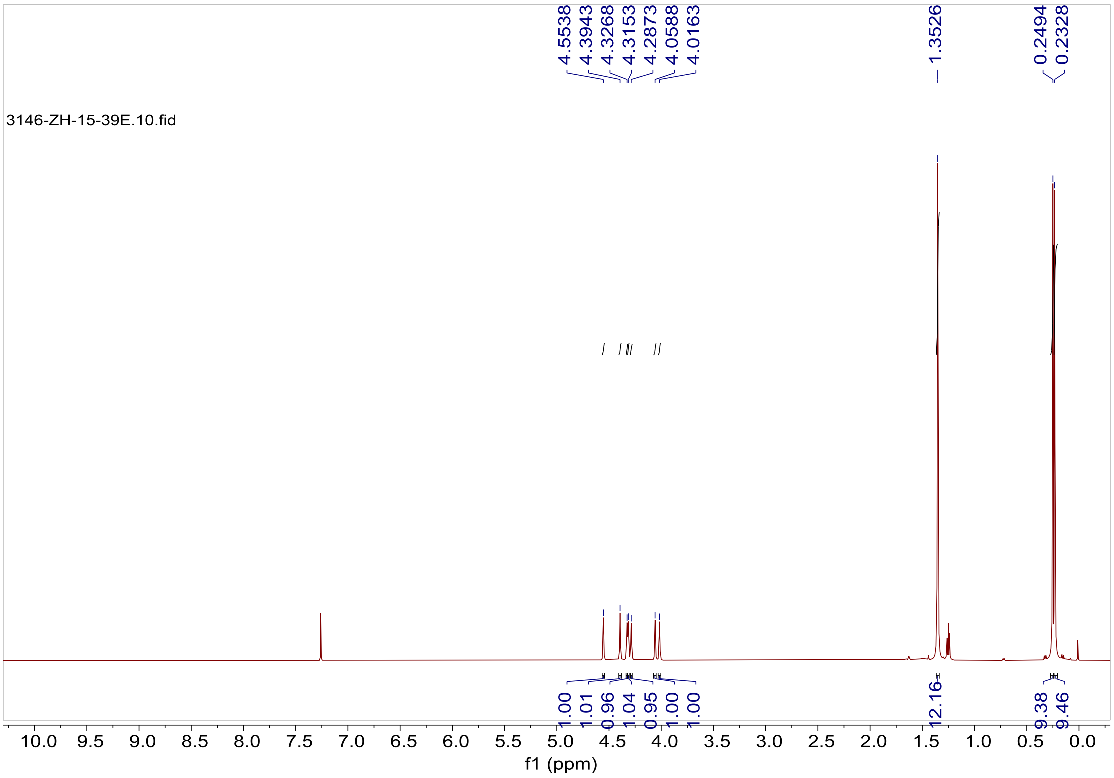

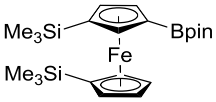

6d

^{1}H NMR (700 MHz, $CDCl_3$)
^{13}C NMR (175 MHz, $CDCl_3$)

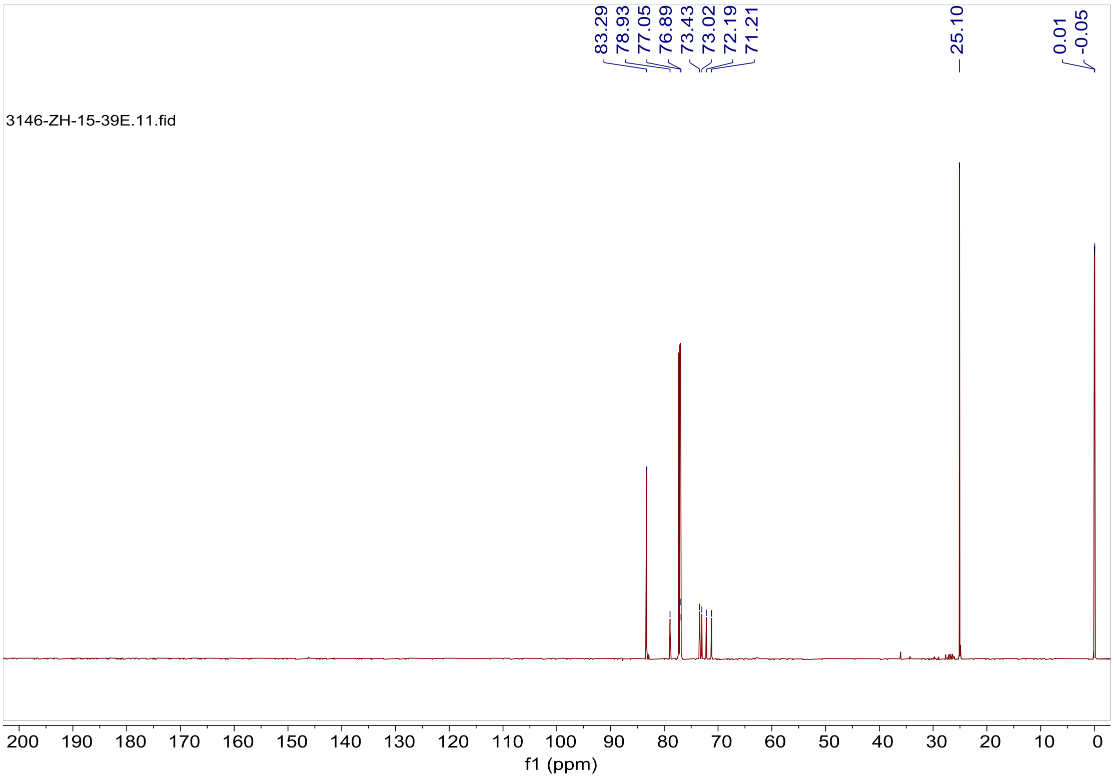

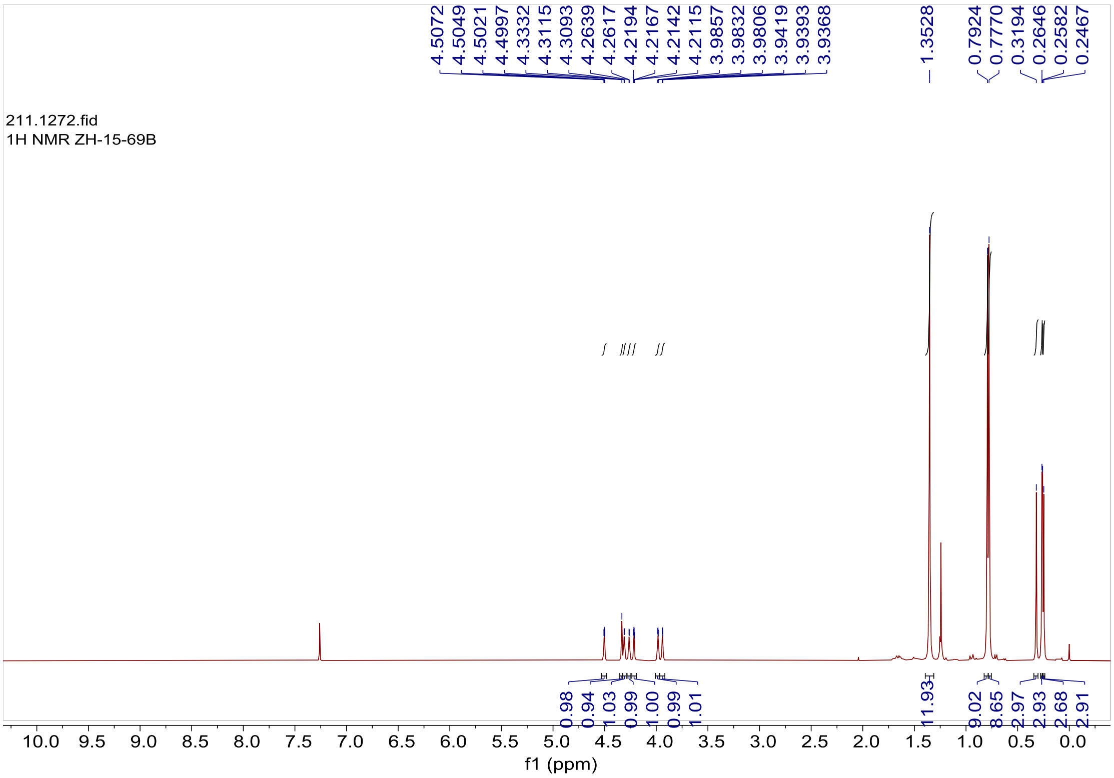

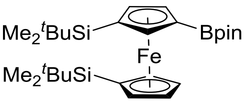

6e

^{1}H NMR (400 MHz, $CDCl_3$)
^{13}C NMR (100 MHz, $CDCl_3$)

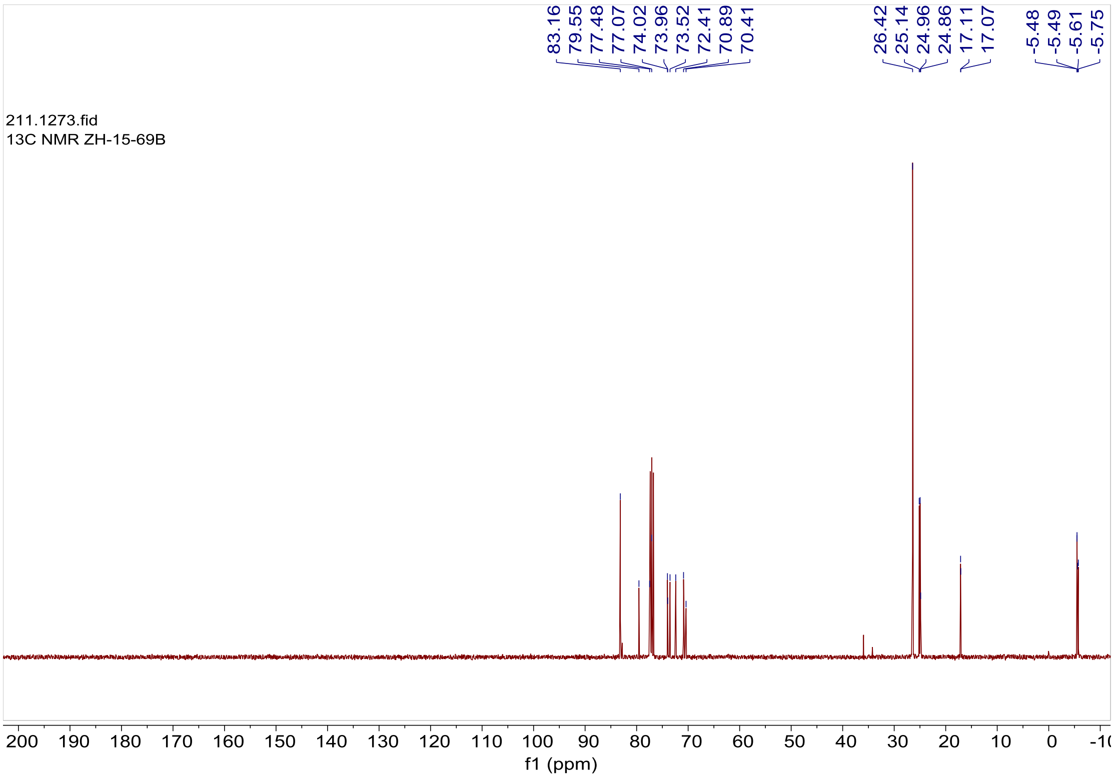

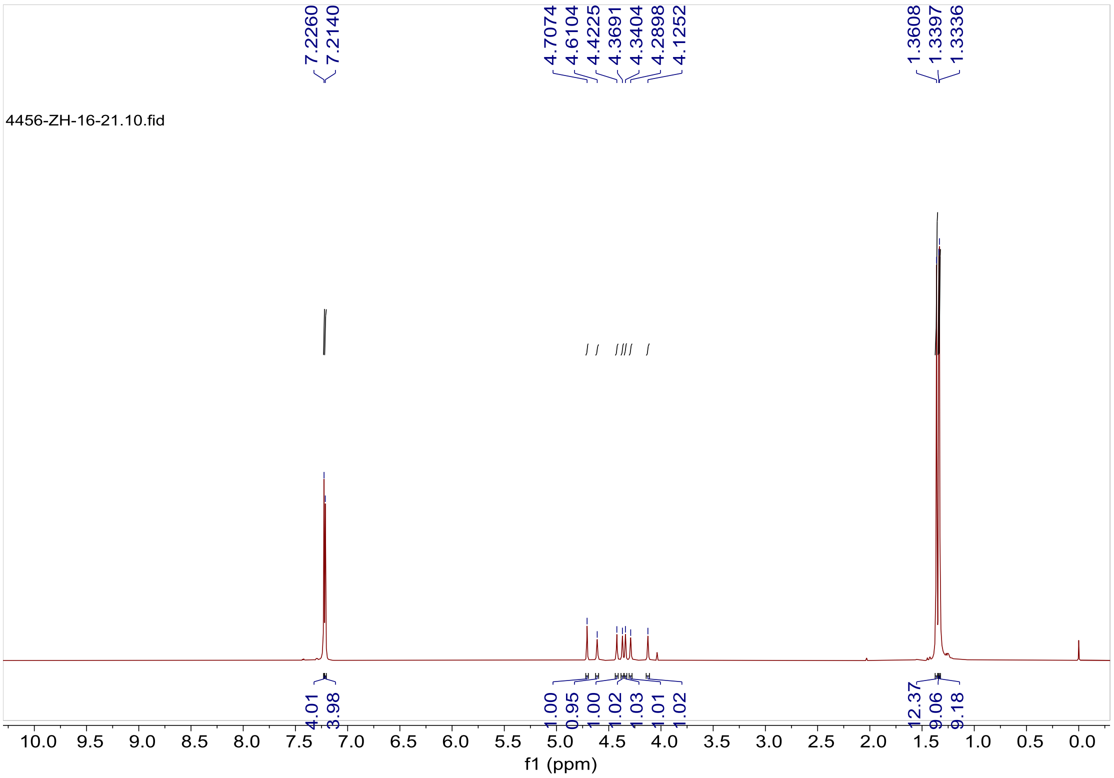

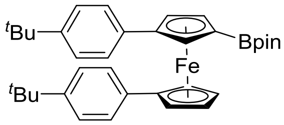

6f

^{1}H NMR (700 MHz, $CDCl_3$)

^{13}C NMR (175 MHz, $CDCl_3$)

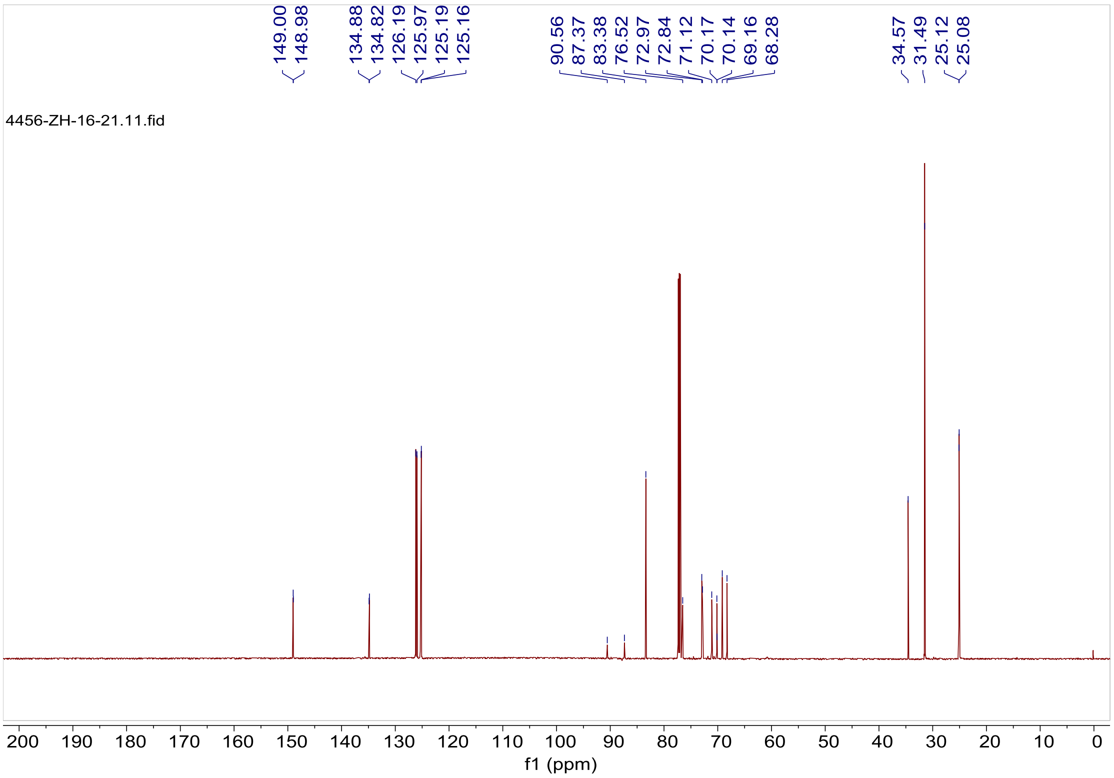

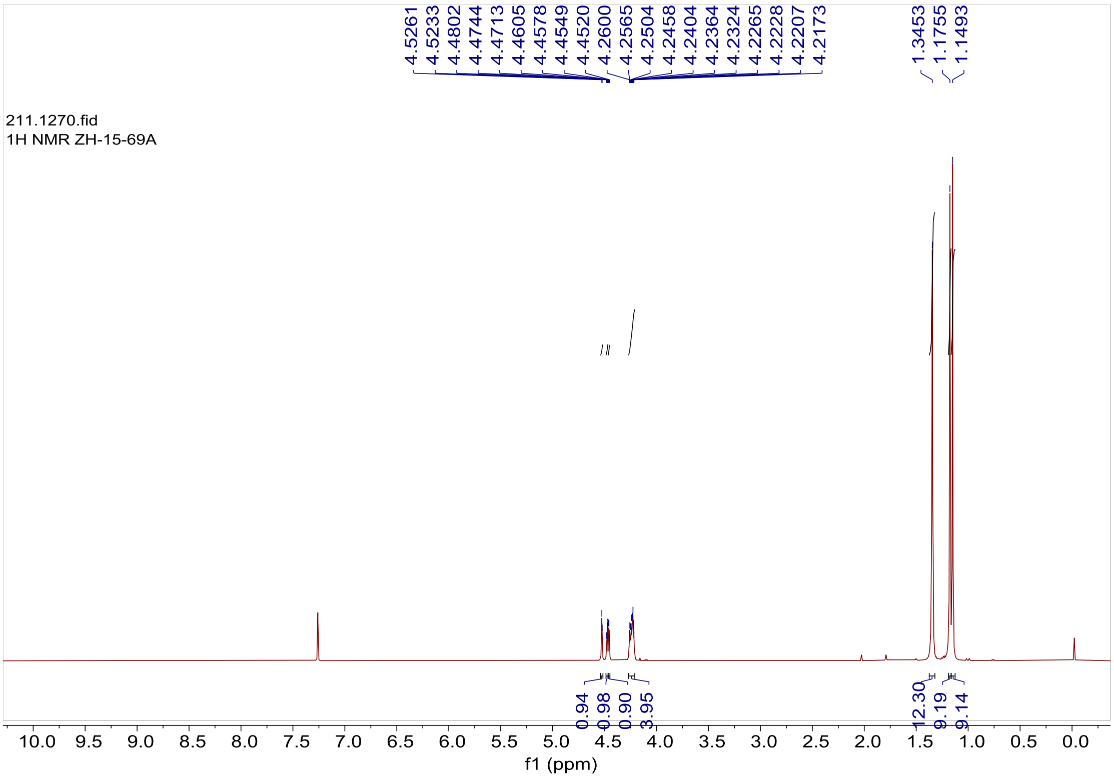

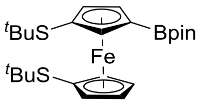

6g

^{1}H NMR (400 MHz, $CDCl_3$)
^{13}C NMR (100 MHz, $CDCl_3$)

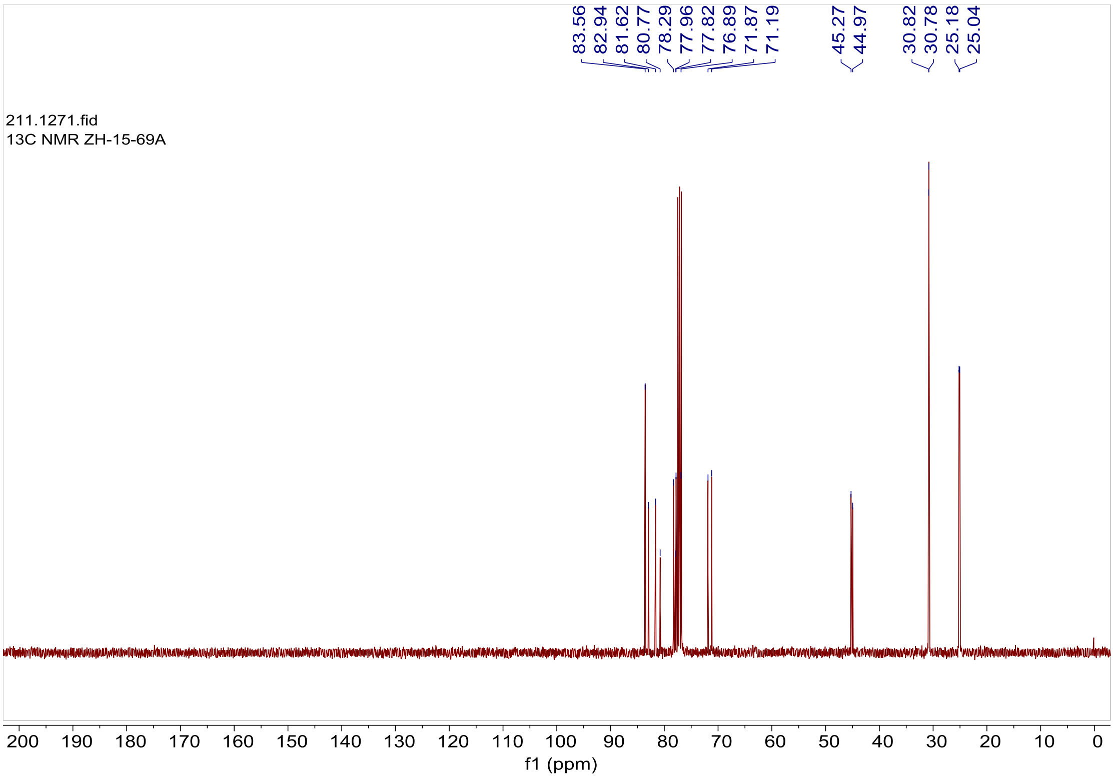

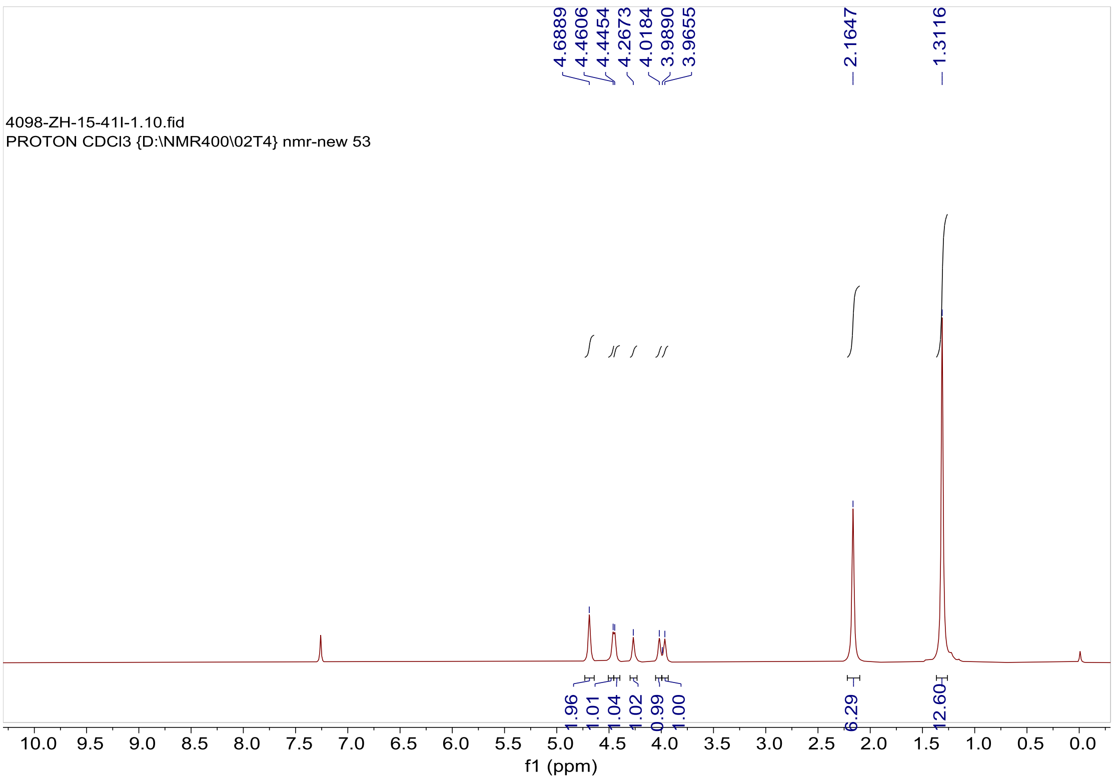

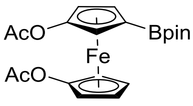

6h

^{1}H NMR (400 MHz, $CDCl_3$)
^{13}C NMR (100 MHz, $CDCl_3$)

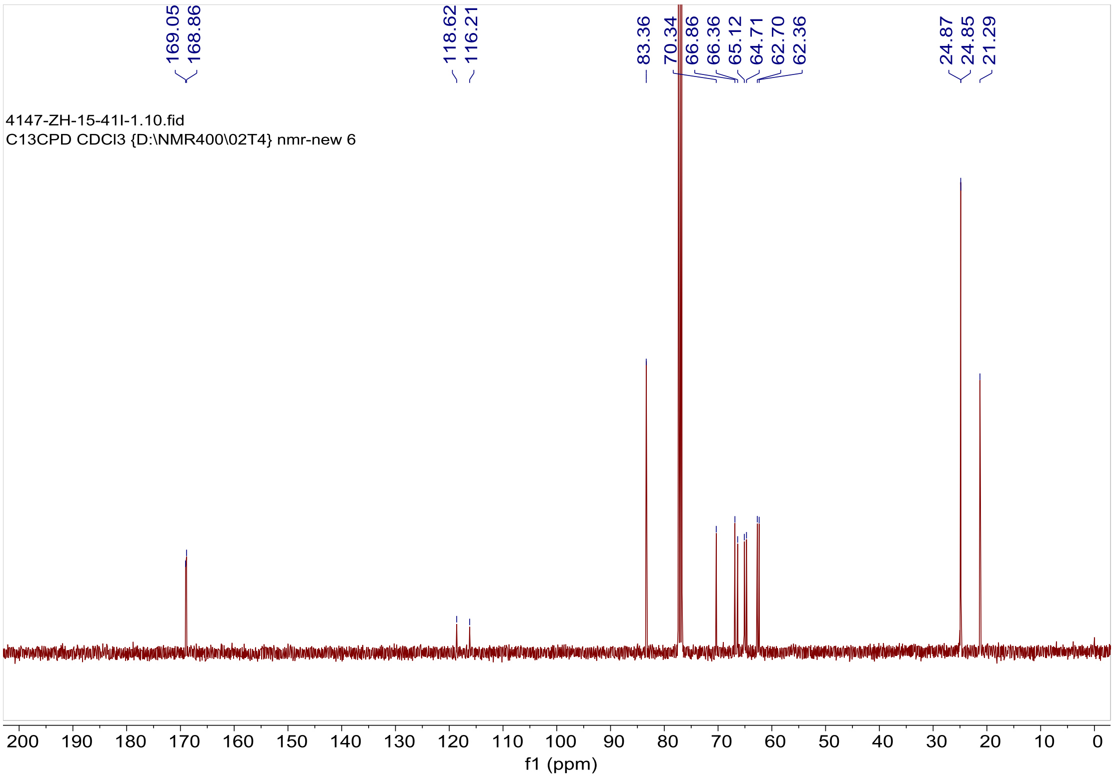

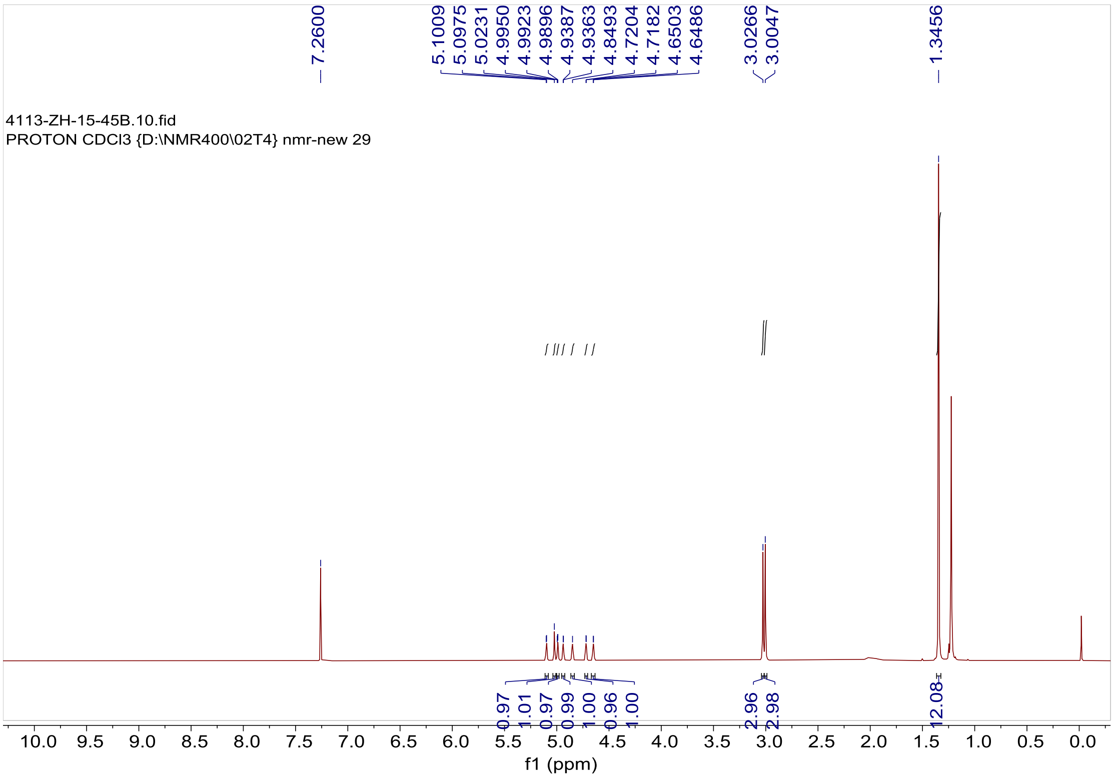

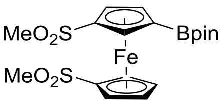

6i

^{1}H NMR (400 MHz, $CDCl_3$)

^{13}C NMR (100 MHz, $CDCl_3$)

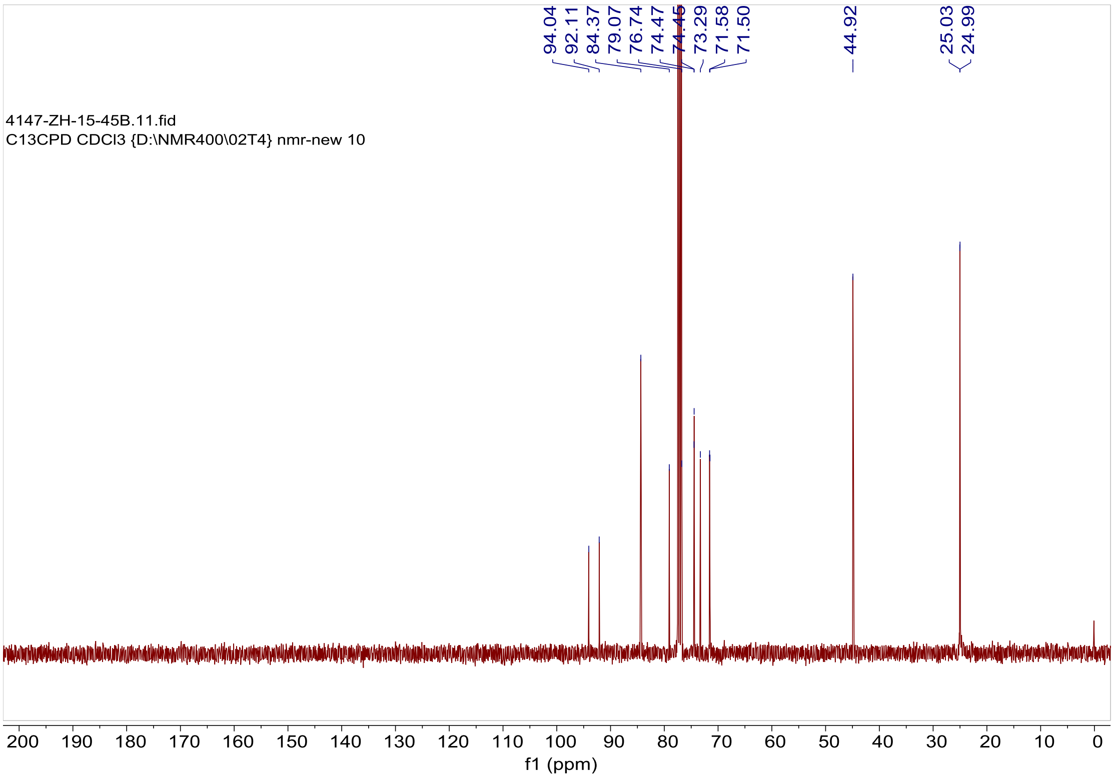

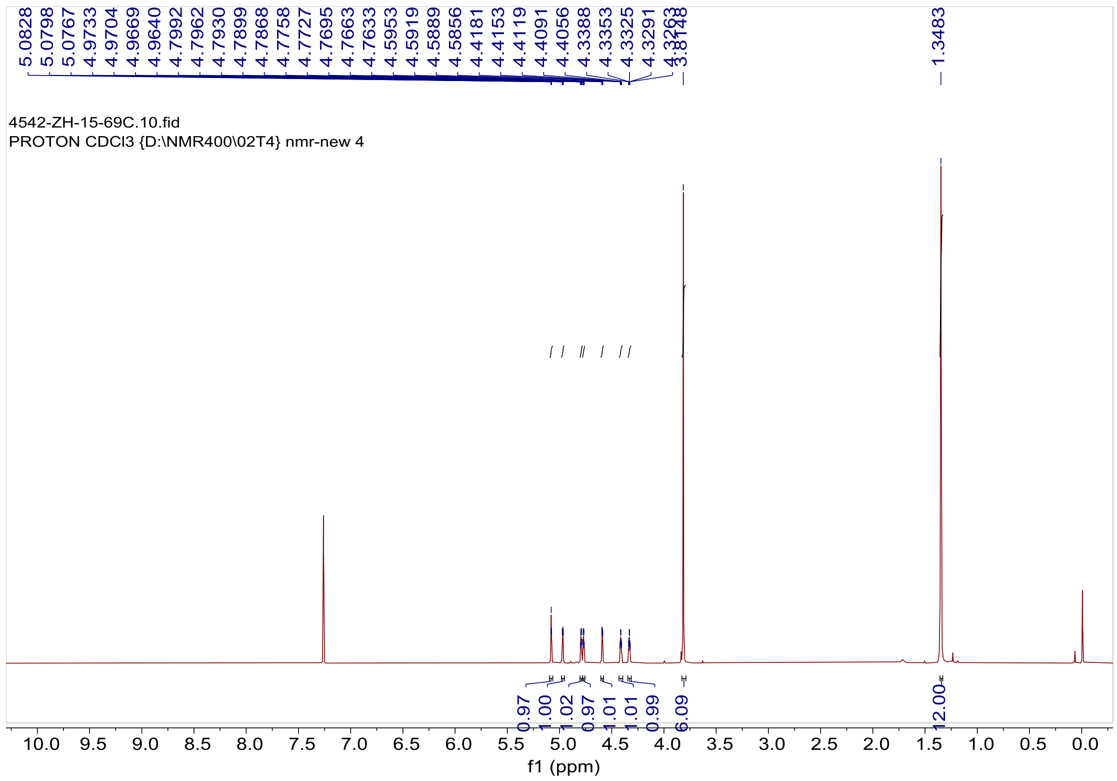

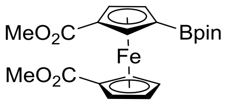

6j

^{1}H NMR (400 MHz, $CDCl_3$)
^{13}C NMR (100 MHz, $CDCl_3$)

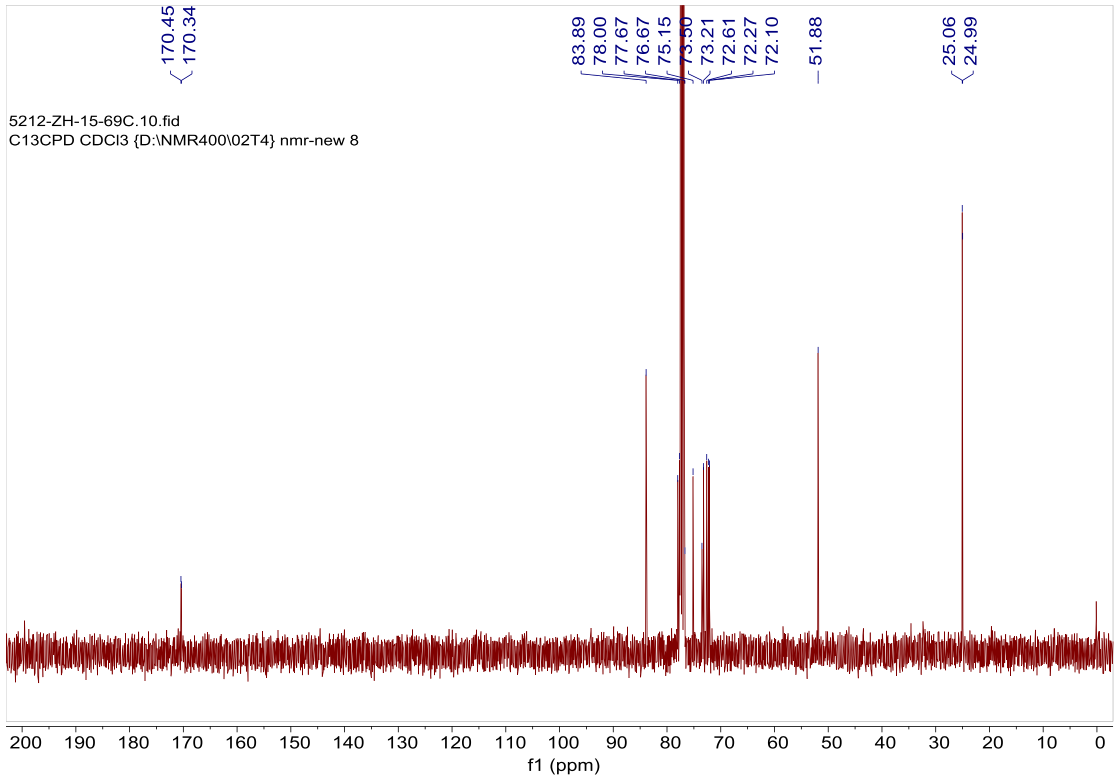

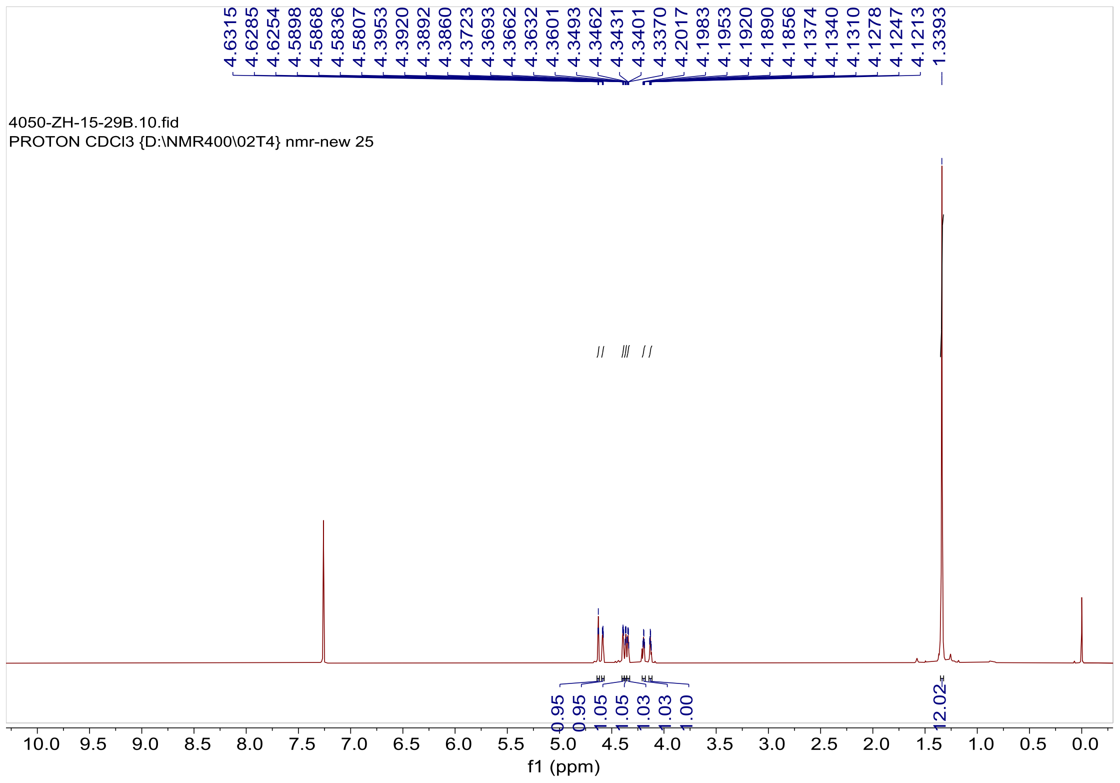

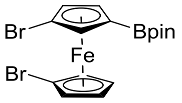

6k

^{1}H NMR (400 MHz, $CDCl_3$)
^{13}C NMR (100 MHz, $CDCl_3$)

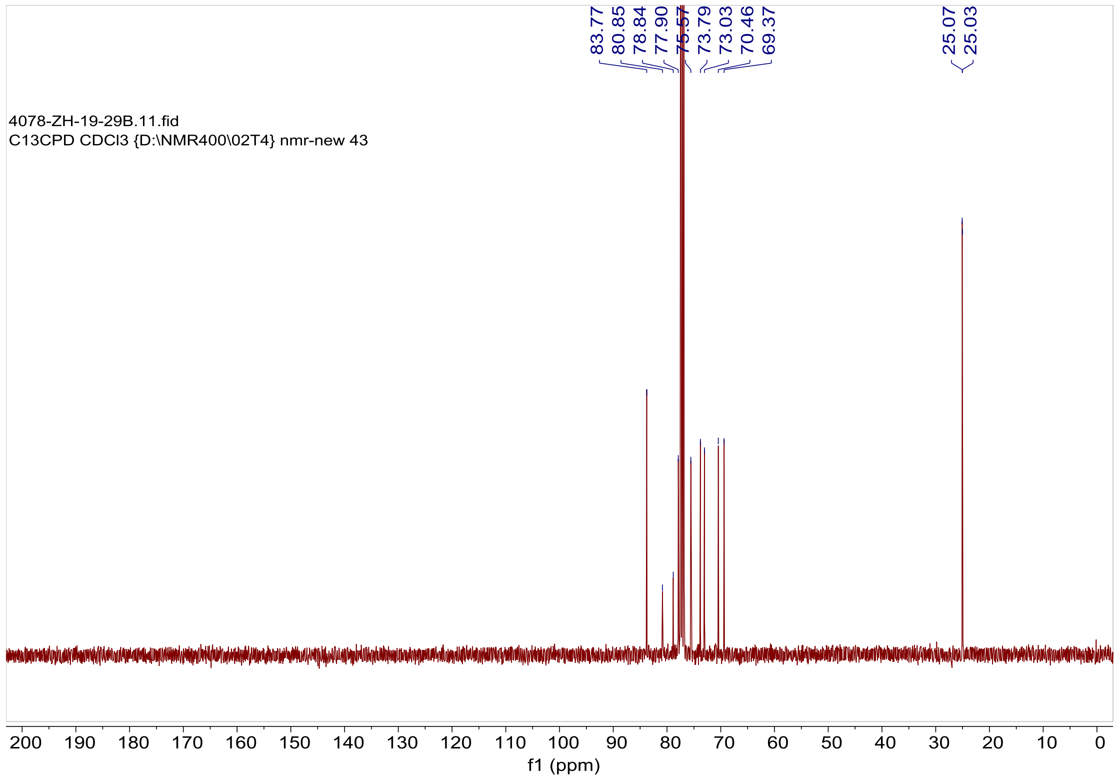

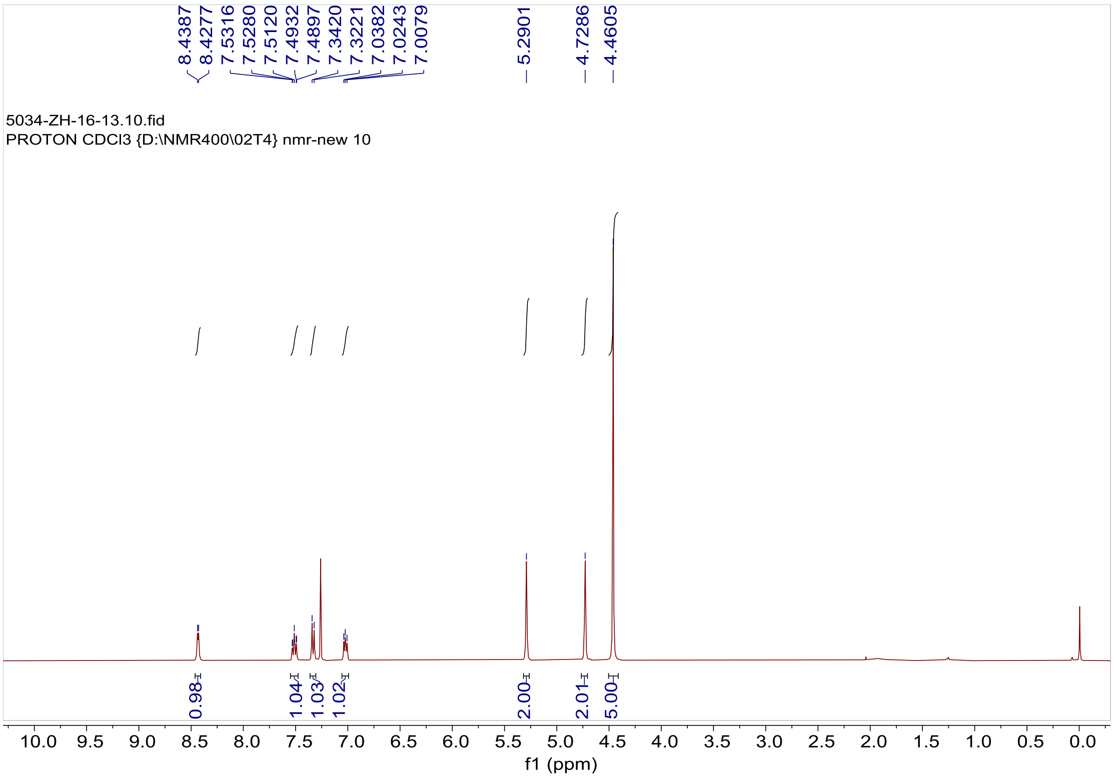

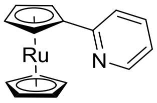

^{1}H NMR (400 MHz, $CDCl_3$)

6m

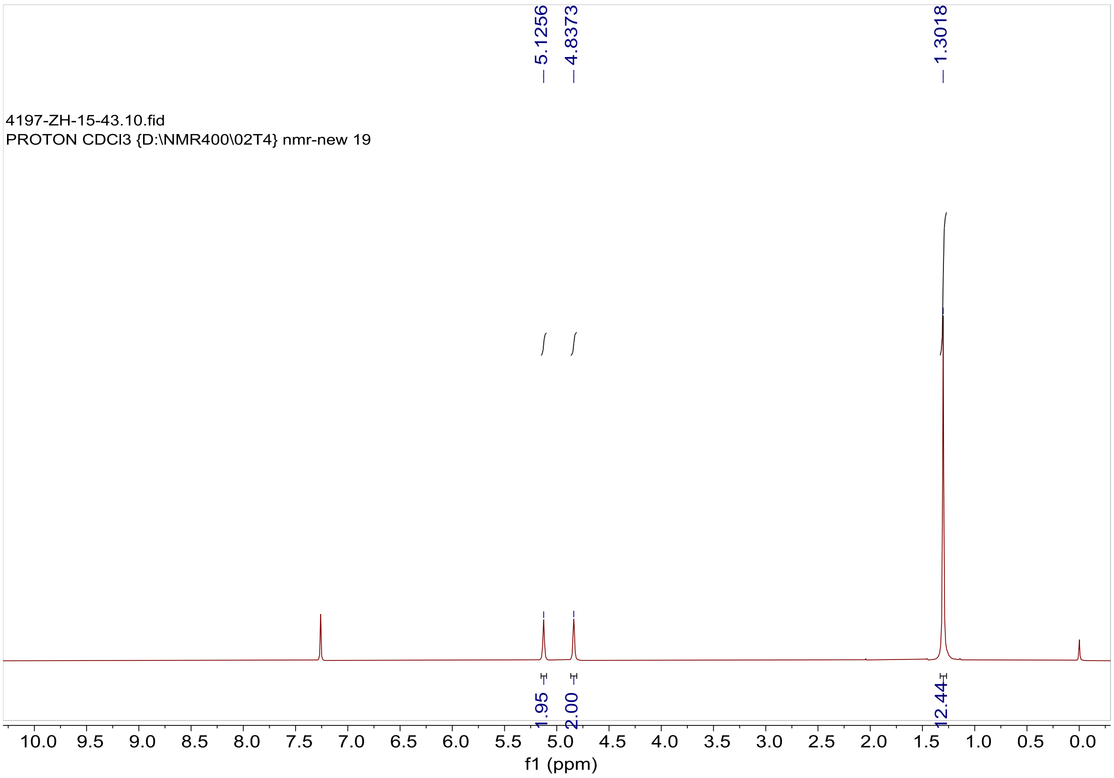

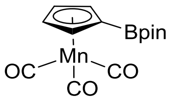

6n

^{1}H NMR (400 MHz, $CDCl_3$)

^{13}C NMR (100 MHz, $CDCl_3$)

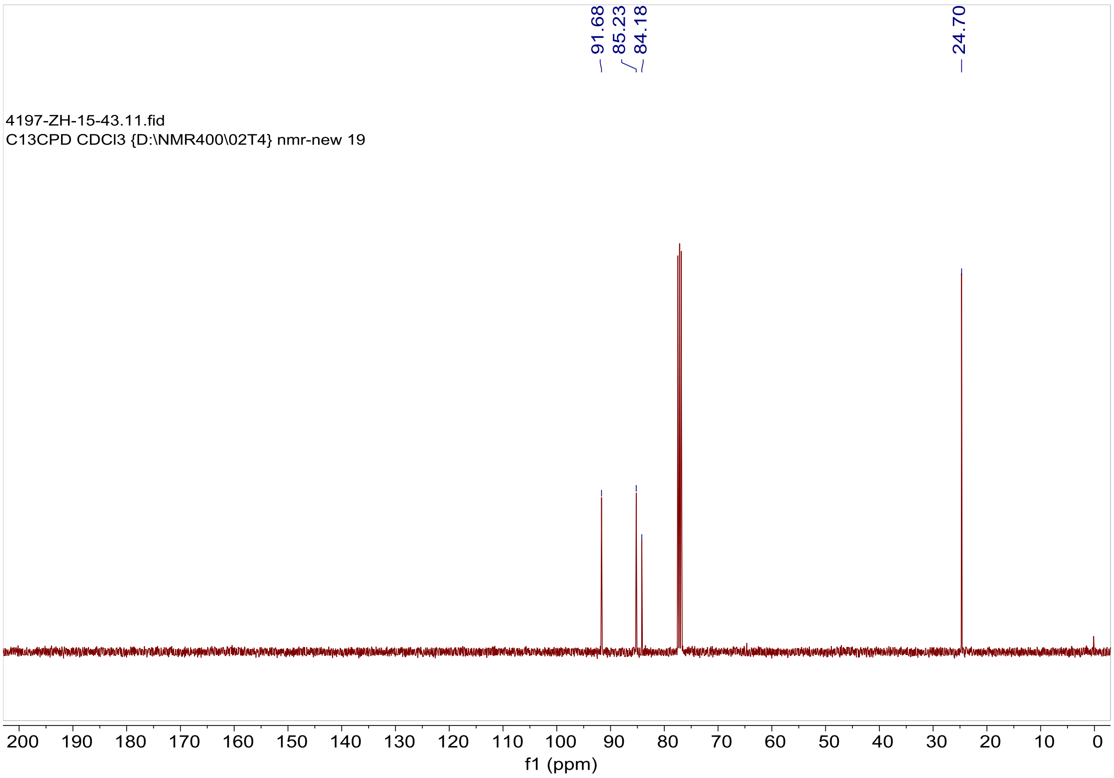

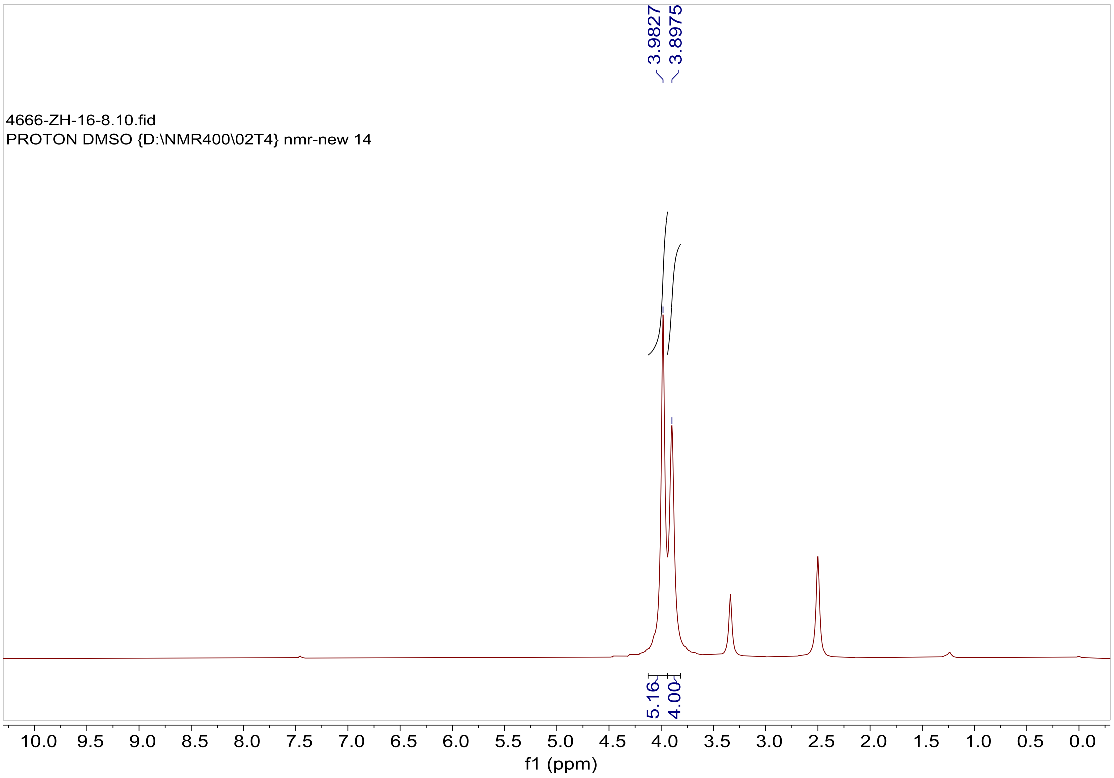

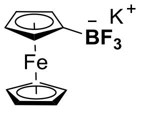

7

^{1}H NMR (400 MHz, DMSO)

^{19}F NMR (376 MHz, DMSO)

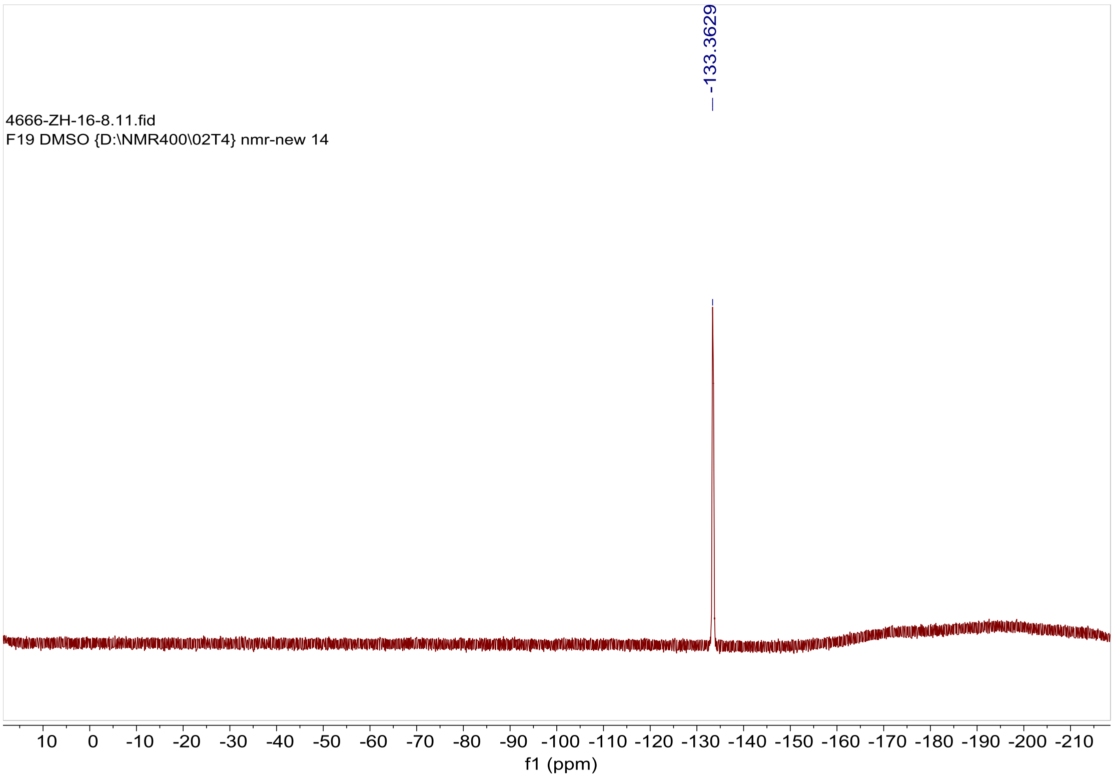

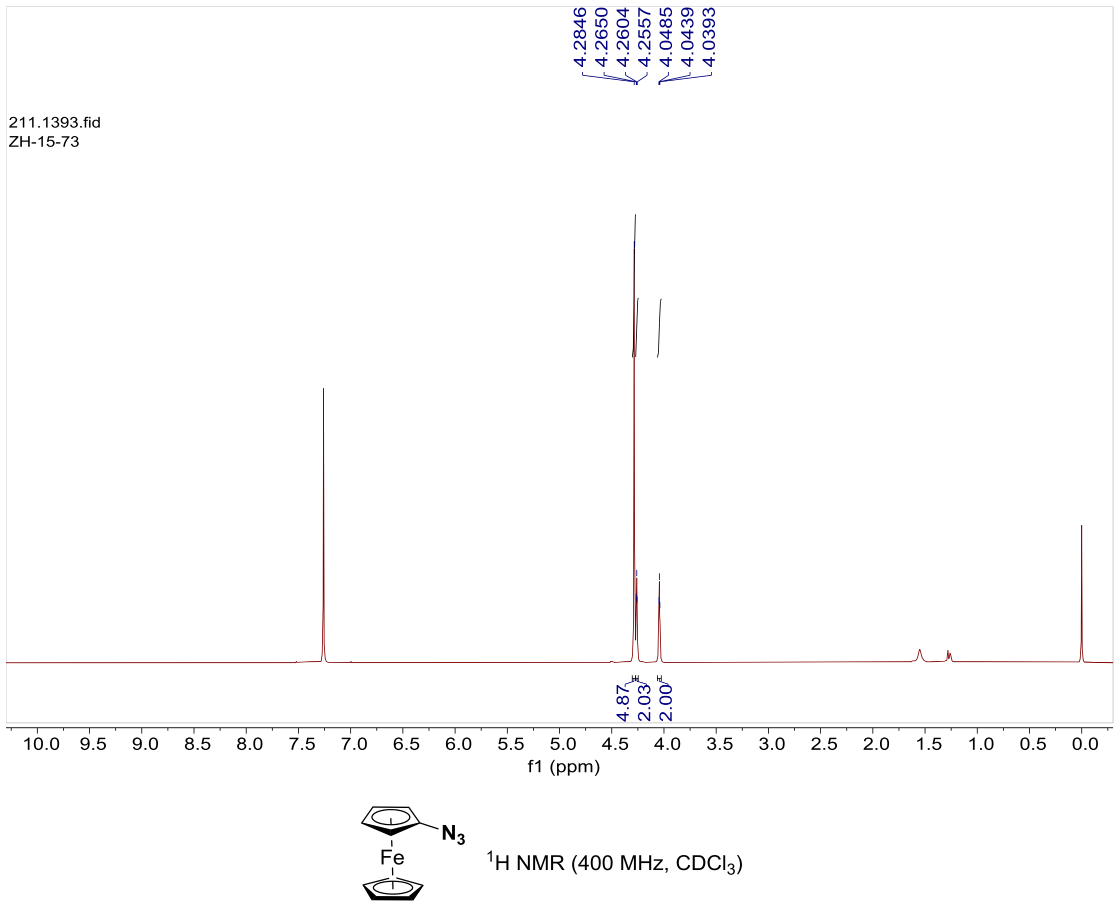

8

^{1}H NMR (400 MHz, $CDCl_3$)

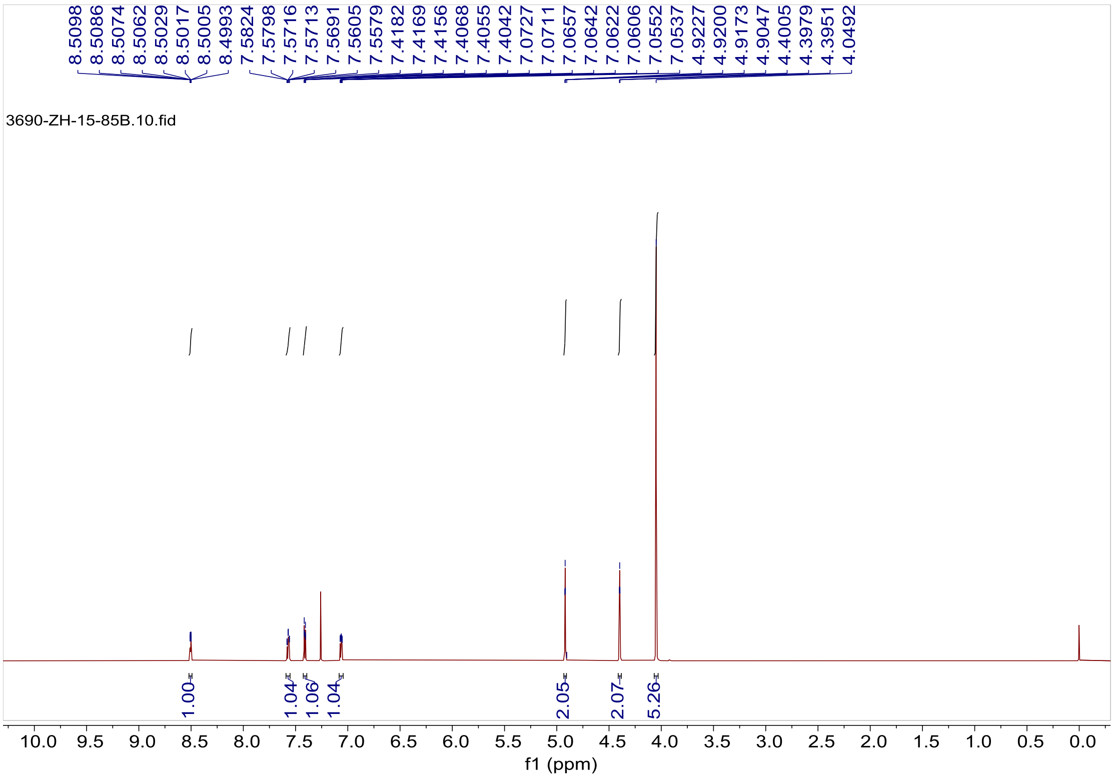

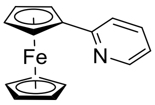

9

^{1}H NMR (400 MHz, $CDCl_3$)

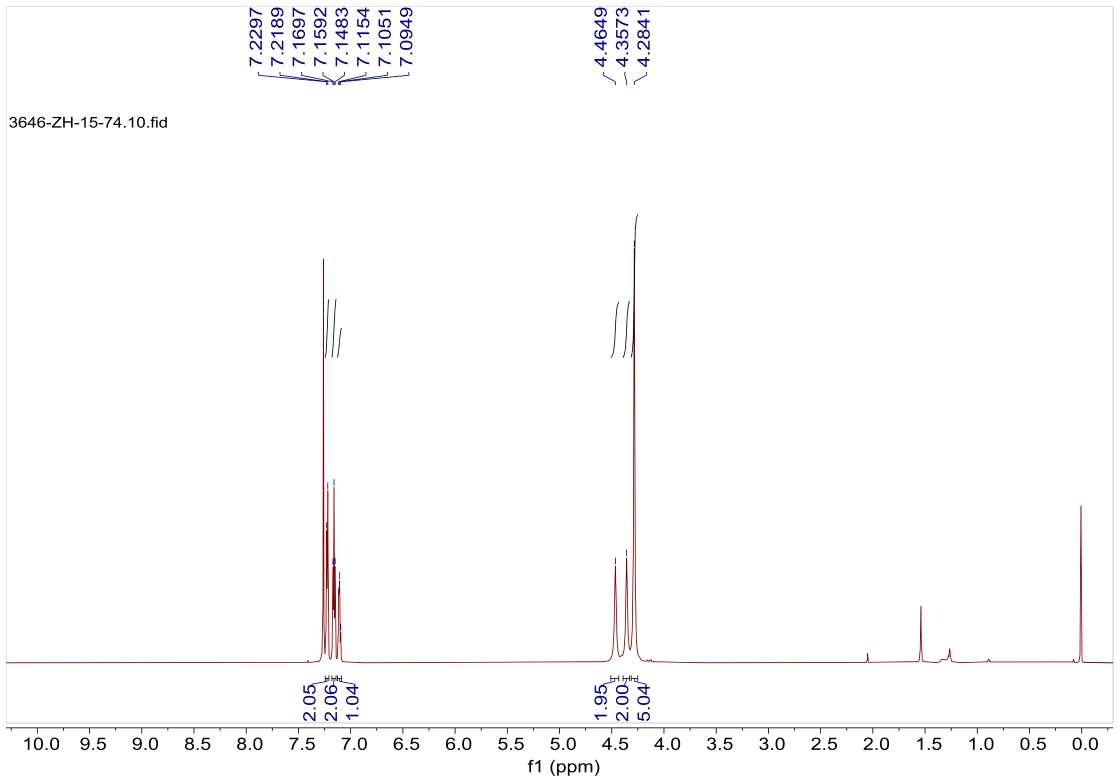

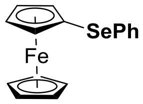

10

^{1}H NMR (400 MHz, $CDCl_3$)

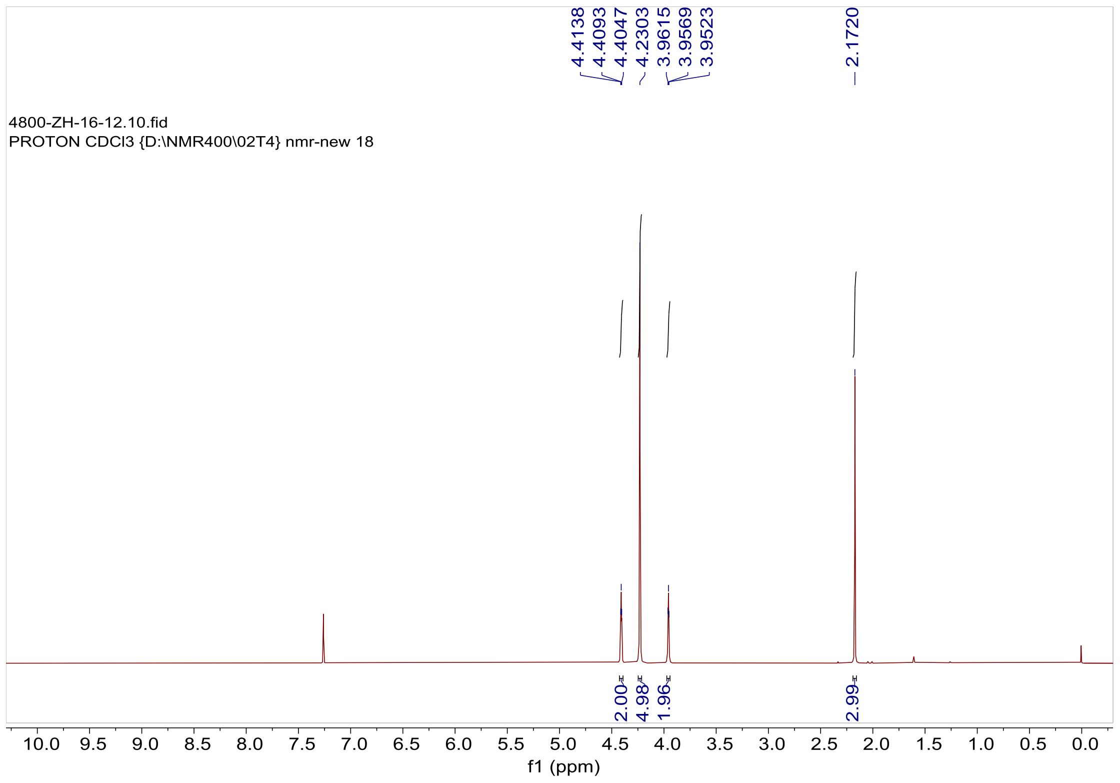

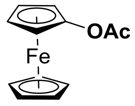

11

^{1}H NMR (400 MHz, $CDCl_3$)
^{13}C NMR (100 MHz, $CDCl_3$)

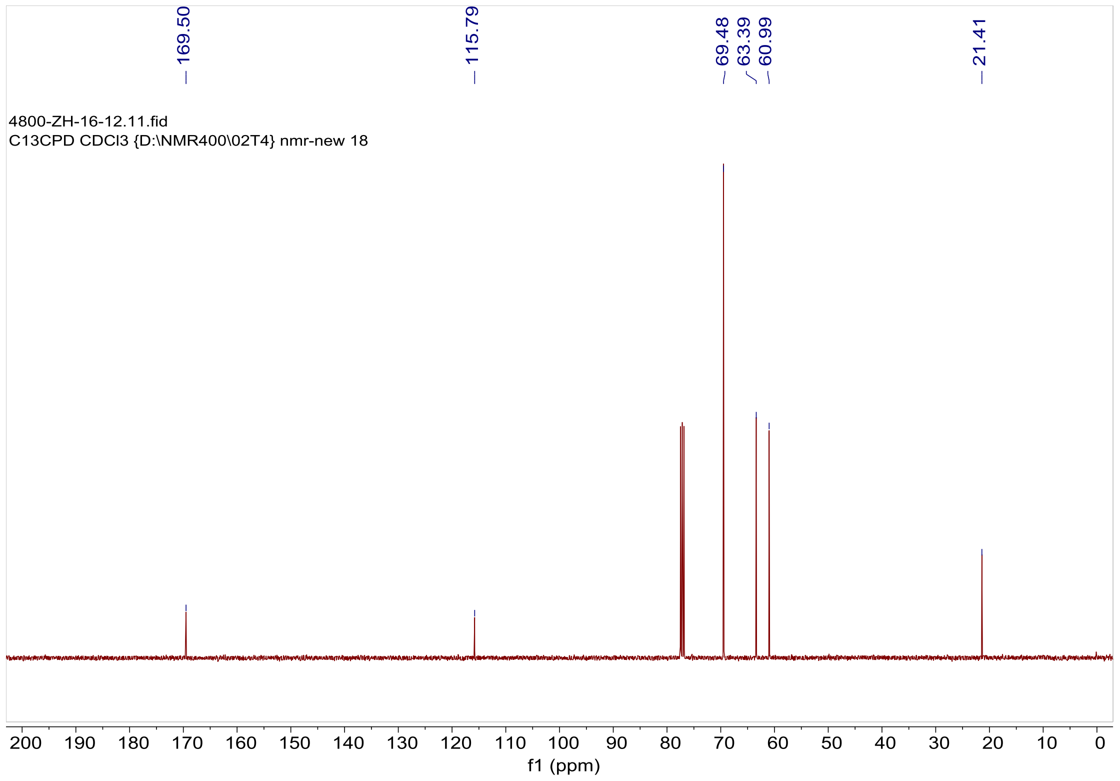

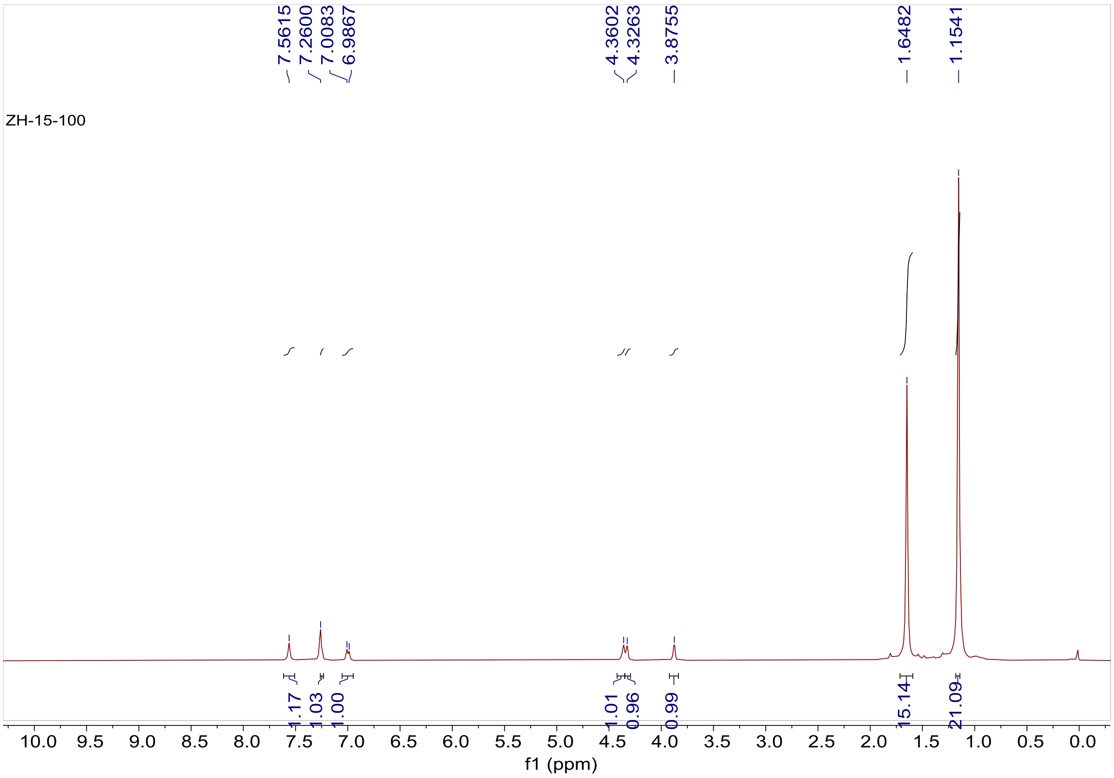

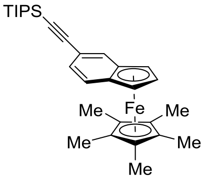

12

^{1}H NMR (400 MHz, $CDCl_3$)

^{13}C NMR (100 MHz, $CDCl_3$)

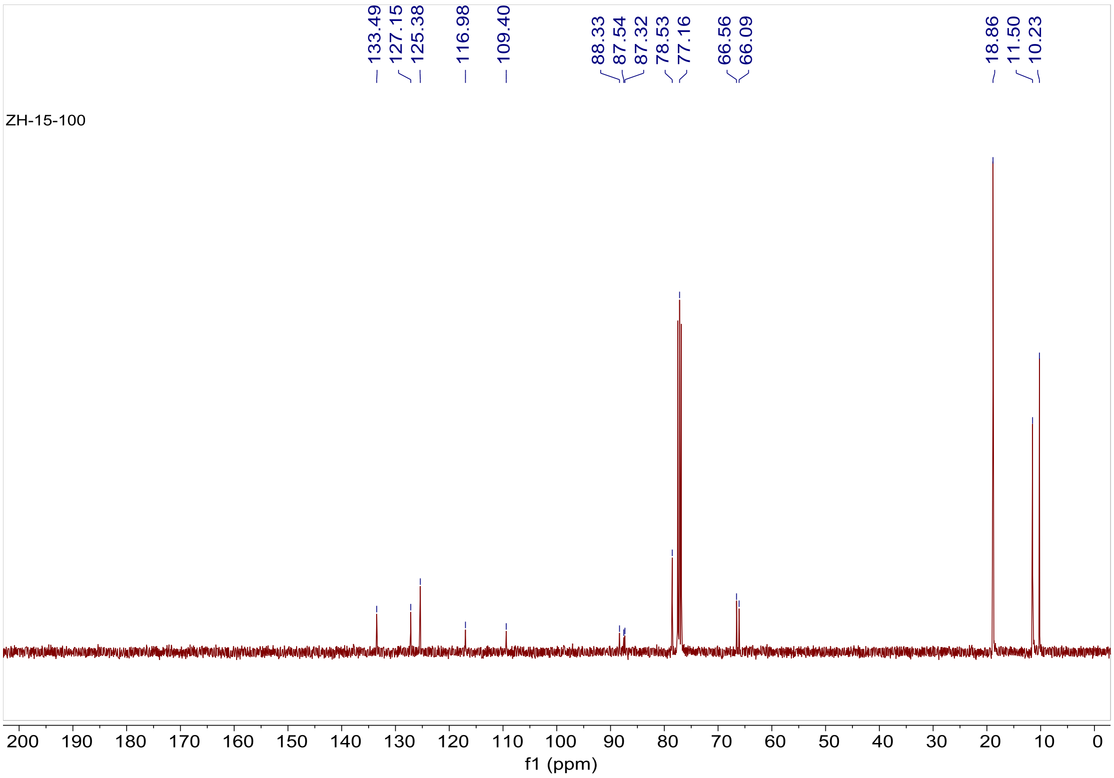

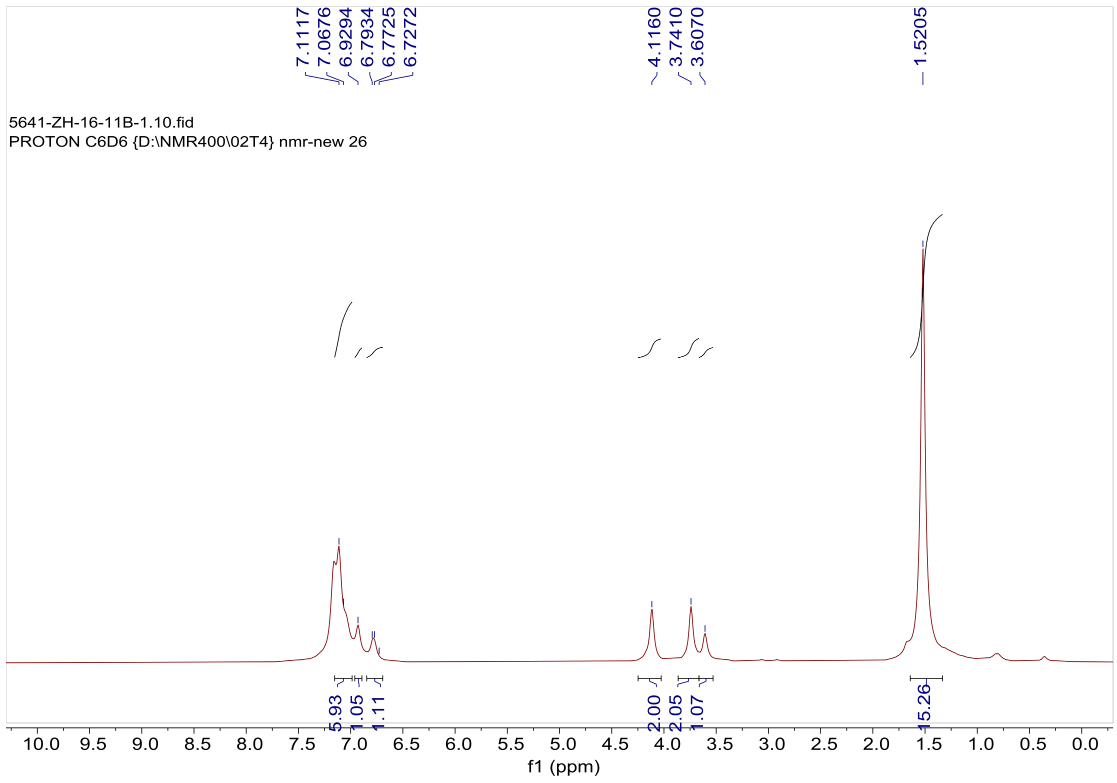

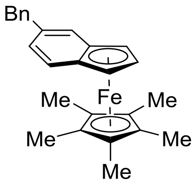

13

^{1}H NMR (400 MHz, C_6D_6)

^{13}C NMR (100 MHz, C_6D_6)

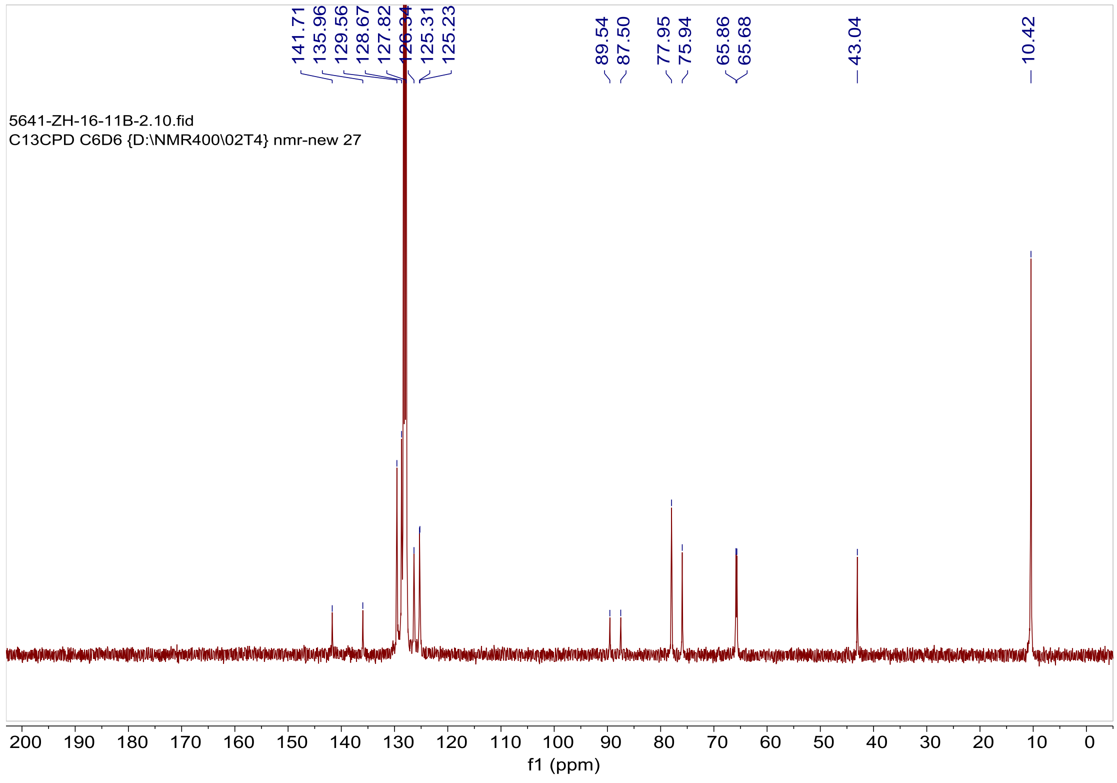

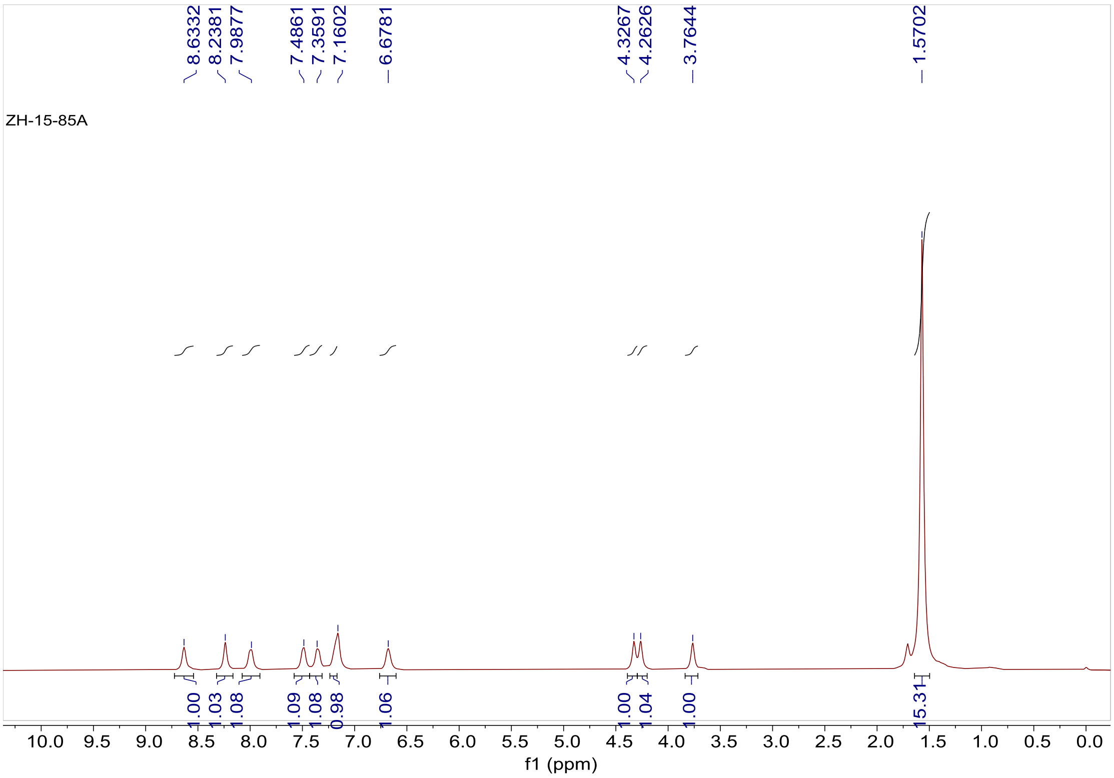

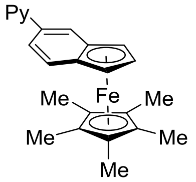

14

^{1}H NMR (400 MHz, $CDCl_3$)

^{13}C NMR (100 MHz, $CDCl_3$)

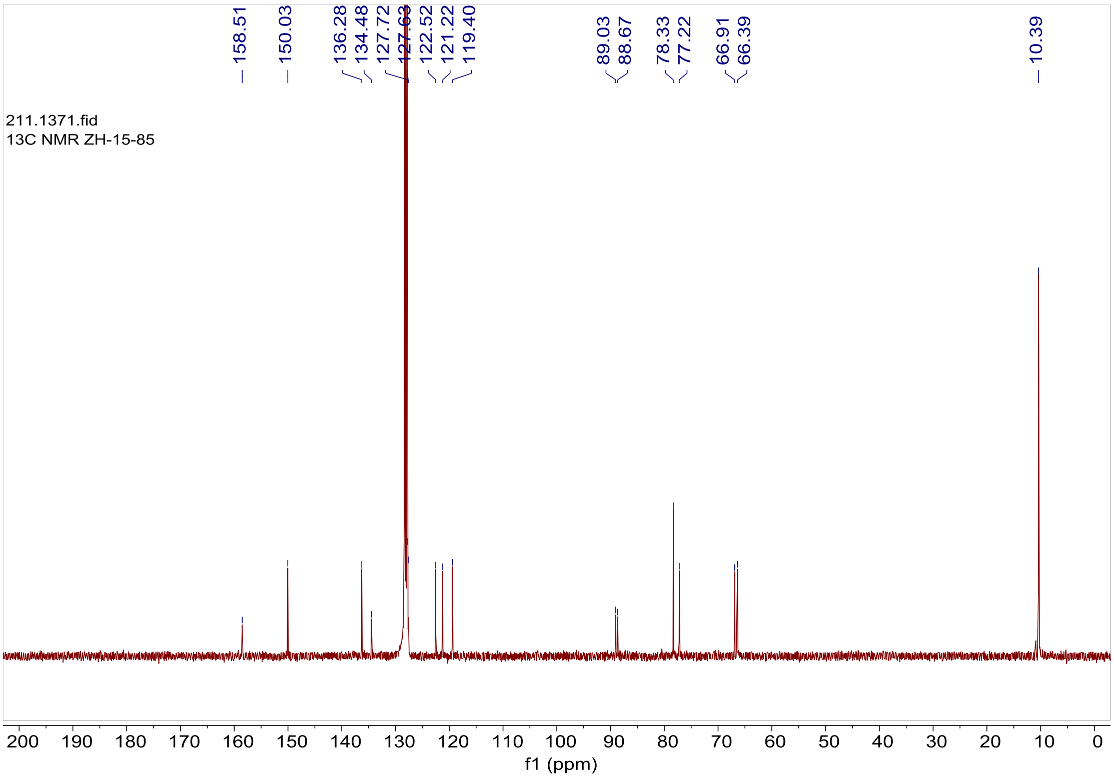

7. References

[1] H. Zheng, C.-H. Liu, S.-Y. Guo, G.-C. He, X.-T. Min, B.-C. Zhou, D.-W. Ji, Y.-C. Hu, Q.-A. Chen, *Nat. Commun.* **2022**, *13*, 3496.

[2] M. R. Jones, C. D. Fast, N. D. Schley, *J. Am. Chem. Soc.* **2020**, *142*, 6488.

[3] L. C. Brown, E. Ressegue, J. S. Merola, *Organometallics* **2016**, *35*, 4014.

[4] R. Fernandez, A. Grirrane, I. Resa, A. Rodriguez, E. Carmona, E. Alvarez, E. Gutierrez-Puebla, A. Monge, J. M. Lopez del Amo, H. H. Limbach, A. Lledos, F. Maseras, D. del Rio, *Chem. Eur. J.* **2009**, *15*, 924.

[5] A. C. Moller, R. H. Heyn, R. Blom, O. Swang, C. H. Gorbitz, J. Kopf, *Dalton Trans.* **2004**, 1578.

[6] C. Zhang, Y. Zhou, J. Huang, C. Tu, X. Zhou, G. Yin, *Org. Biomol. Chem.* **2018**, *16*, 6316.

[7] K. A. Rufanov, I. Y. Titov, D. A. Lemenovskii, D. P. Krut'ko, A. V. Churakov, *New J. Chem.* **2022**, *46*, 1409.

[8] D. M. Tschaen, R. Desmond, A. O. King, M. C. Fortin, B. Pipik, S. King, T. R. Verhoeven, *Synth. Commun.* **1994**, *24*, 887.

[9] J. Mo, S. Liu, J. Xiao, *Tetrahedron* **2005**, *61*, 9902.

[10] J. Ju, M. Jeong, J. Moon, H. Jung, S. Lee, *Org. Lett.* **2007**, *9*, 4615.

[11] D. A. Khobragade, S. G. Mahamulkar, L. Pospisil, I. Cisarova, L. Rulisek, U. Jahn, *Chem. Eur. J.* **2012**, *18*, 12267.

[12] H. Liang, L. Vasamsetty, T. Li, J. Jiang, X. Pang, J. Wang, *Chem. Eur. J.* **2020**, *26*, 14546.

[13] K. Gonsalves, Z. R. Lin, M. D. Rausch, *J. Am. Chem. Soc.* **1984**, *106*, 3862.

[14] A. Datta, A. Kollhofer, H. Plenio, *Chem. Commun.* **2004**, 1508.

[15] K. Sünkel, S. Weigand, *Inorg. Chim. Acta* **2011**, *370*, 224.

[16] W. Erb, J.-P. Hurvois, T. Roisnel, V. Dorcet, *Organometallics* **2018**, *37*, 3780.

[17] S. Vanicek, M. Jochriem, C. Hassenruck, S. Roy, H. Kopacka, K. Wurst, T. Muller, R. F. Winter, E. Reisner, B. Bildstein, *Organometallics* **2019**, *38*, 1361.

[18] G.-C. He, S.-Y. Guo, H. Zheng, C.-H. Liu, Y. Li, X.-T. Min, D.-W. Ji, Q.-A. Chen, *Cell Rep. Phys. Sci.* **2022**, *3*, 100768.

[19] D. Białek, K. Kowalski, J. Ścianowski, Z. Rafiński, A. Wojtczak, *J. Organomet. Chem.* **2012**, *712*, 1.

[20] D. Schaarschmidt, H. Lang, *Eur. J. Inorg. Chem.* **2010**, *2010*, 4811.
